# Organic Four‐Electron Redox Systems Based on Bipyridine and Phenanthroline Carbene Architectures

**DOI:** 10.1002/anie.202203064

**Published:** 2022-04-12

**Authors:** Patrick W. Antoni, Christopher Golz, Max M. Hansmann

**Affiliations:** ^1^ Fakultät für Chemie und Chemische Biologie Technische Universität Dortmund Otto-Hahn-Str.6 44227 Dortmund Germany; ^2^ Georg-August Universität Göttingen Institut für Organische und Biomolekulare Chemie Tammannstr. 2 37077 Göttingen Germany

**Keywords:** Carbenes, Electron Donor, Redox Chemistry, Structure Elucidation

## Abstract

Novel organic redox systems that display multistage redox behaviour are highly sought‐after for a series of applications such as organic batteries or electrochromic materials. Here we describe a simple strategy to transfer well‐known two‐electron redox active bipyridine and phenanthroline architectures into novel strongly reducing four‐electron redox systems featuring fully reversible redox events with up to five stable oxidation states. We give spectroscopic and structural insight into the changes involved in the redox‐events and present characterization data on all isolated oxidation states. The redox‐systems feature strong UV/Vis/NIR polyelectrochromic properties such as distinct strong NIR absorptions in the mixed valence states. Two‐electron charge–discharge cycling studies indicate high electrochemical stability at strongly negative potentials, rendering the new redox architectures promising lead structures for multi‐electron anolyte materials.

## Graphical abstract

Novel four‐electron redox‐systems featuring up to five isolable oxidation states are presented. The synthesis is modular and based on the combination of diquat or phenanthrolinium salts with stable carbenes. The different oxidation states can be isolated and feature diradical and mixed valence states. Charge/discharge experiments confirm that these redox systems are promising new lead structures for two‐electron anolyte materials.

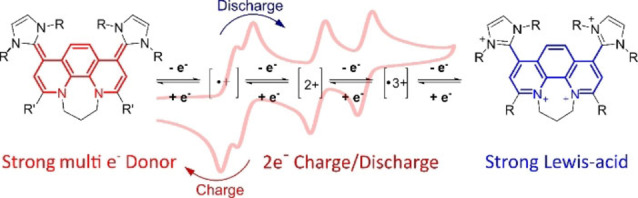

## Introduction

**Figure 1:**
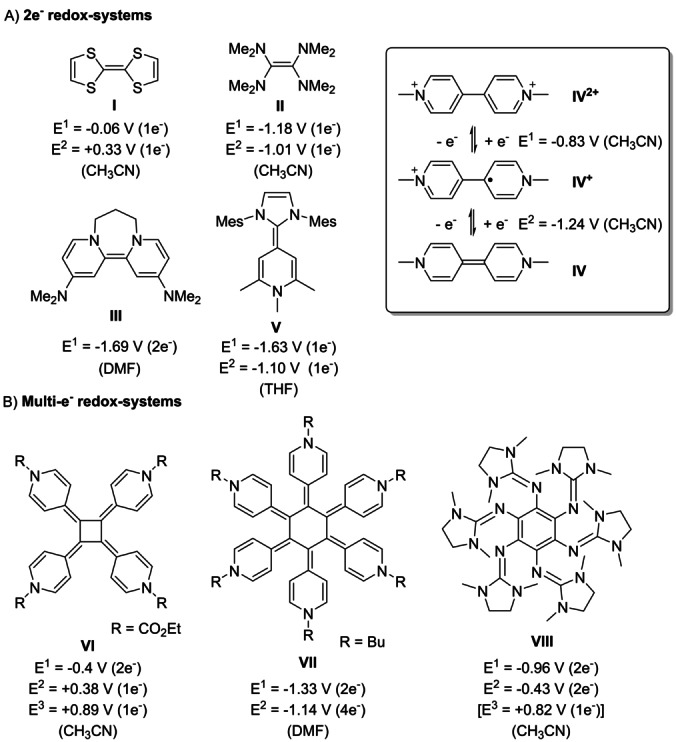
A) Selection of electron rich olefins as two‐electron redox systems (**I**–**V**), the parent paraquat redox‐system (**IV**) and B) multi‐electron redox systems (**VI**–**VIII**). All redox potentials listed against the Fc/Fc^+^ couple for comparison: **I^[^** ^6c]^ and **II^[^** ^6c]^ (corrected by −0.38 V SCE vs. Fc/Fc^+^)^[19]^, **III**,^[7e]^ **IV^[^** ^21]^, **V^[^** ^24]^, **VI^[^** ^20]^ (corrected by −0.50 V Ag/AgCl vs. Fc/Fc^+^), **VII^[^** ^22]^, **VIII^[^** ^23]^.

Organic redox systems are receiving significant attention due to their manifold of applications in advanced materials and organic electronics^[1]^ and are widely applied as electrochromic display materials or memory devices in supramolecular systems.[^2^, ^3^] Typical representatives of organic two‐electron redox systems are electron rich olefins such as tetrathiafulvalene (**I**; TTF)^[4]^ or tetrakis(dimethylamino)ethene (**II**; TDAE)^[5]^ (Figure 1). Due to the negative reduction potential of tetraaminoalkenes, TDAE has also been utilized as organic reductant in organic synthesis. Murphy and co‐workers developed even stronger two‐electron reductants coining the term “organic super‐electron‐donors”,^[6]^ such as bispyridinylene **III**, based on the 2,2′‐bipyridinium core.^[7]^ In fact, presumably the most intensively investigated organic redox systems are “Weitz type”^[8]^ 4,4′‐bispyridinium salts, introduced by Michaelis in 1932 as methyl viologen (**IV** ^2+^),[^9^, ^10^] which feature three stable oxidation states (Figure 1A). The viologen core has been extended,^[11]^ substituted^[12]^ or embedded in heterocyclic^[13]^ or benzannulated systems,^[14]^ with all systems representing two‐electron redox systems. Due to their facile synthetic access and high stability, viologens (4,4′ and 2,2′) found plenty of applications ranging from herbizides (diquat®, paraquat®),^[15]^ reductants,[^6^, ^16^] electrochromic devices to organic flow or polymer batteries.[^17^, ^18^]

We recently described a modular one‐step synthesis of novel two‐electron redox systems **V**, based on the combination of carbenes with pyridinium salts.^[24]^ We could link two pyridinium/carbene hybrids of type **V** with an [N]‐propyl‐[N] spacer, however upon oxidation the two sites acted as independent redox‐systems, with negligible electron/electron interaction.^[24a]^ In fact there are only a limited number of coupled multi‐electron (>two‐electrons) redox‐systems. A common strategy to access such multi‐electron redox systems is to utilize highly symmetrical architectures containing four‐, or six‐fold symmetry (Figure 1B).^[25]^ Beautiful examples are radialenes such as [4]radialene **VI^[^** ^20]^ or the [6]radialene **VII**.^[22]^ The latter only features two redox events with three stable oxidation states (radialene **VII** ^0^, **VII** ^2+^ and **VII** ^6+^). In this line, Himmel and co‐workers reported the strongest neutral four‐electron donor **VIII** with three stable redox states.^[23]^ Note, the few existing organic multi‐electron (>two‐electrons) redox‐systems are typically step‐intense in their synthesis and do not allow for simple structural variation, which however is crucial to tune redox‐properties and stabilities for potential applications. Here we describe a simple single‐step and modular synthetic strategy to transfer well‐established two‐electron redox active bipyridine architectures into novel fully reversible strongly coupled four‐electron redox systems featuring up to five stable oxidation states.

## Results and Discussion

**Scheme 1:**
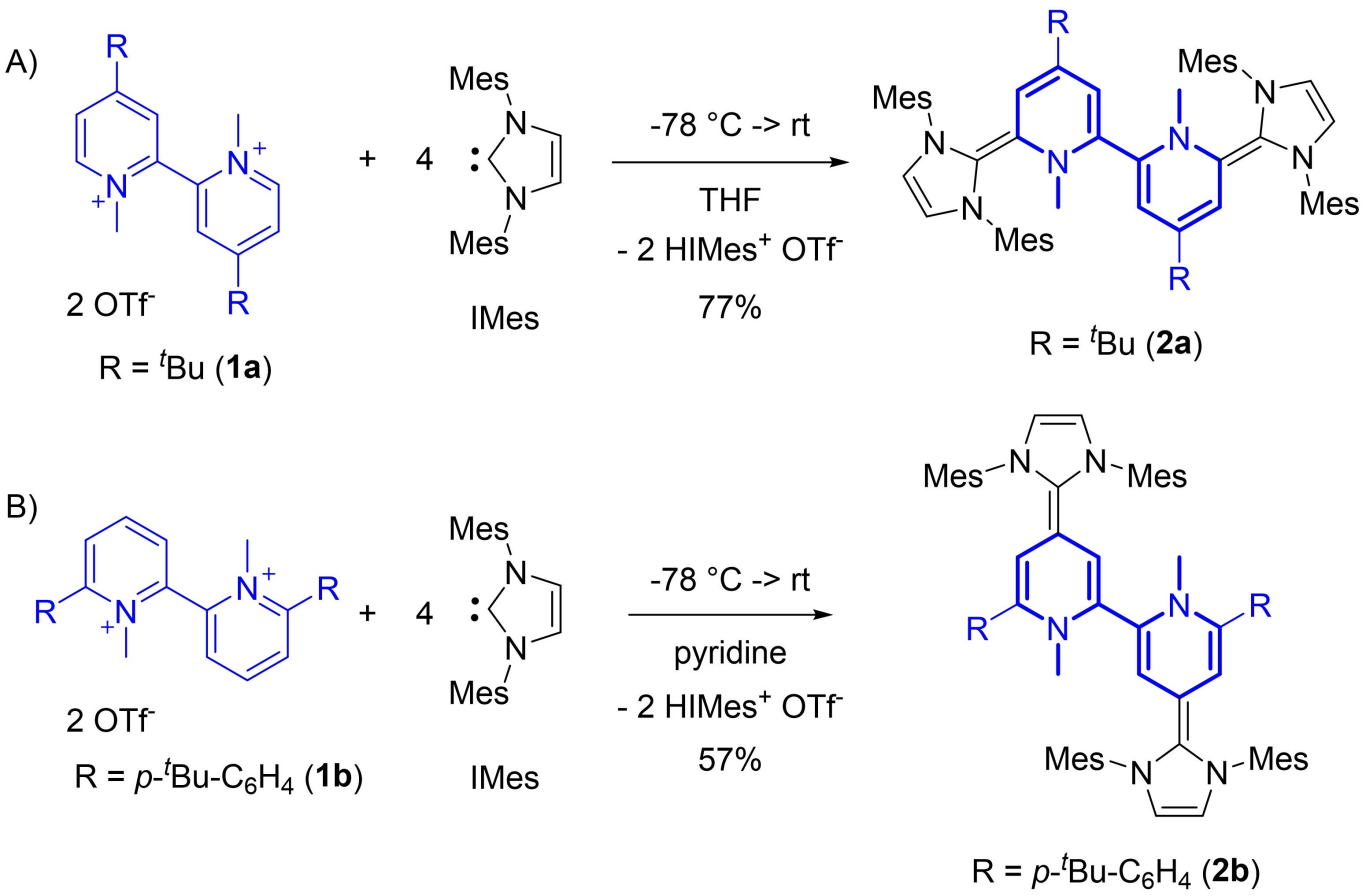
Synthesis of 2,2′‐bipyridine hybrid molecules **2 a** and **2 b**.

We started our investigation by reacting 4,4′‐bis(*tert*butyl)‐2,2′‐bipyridinium salt **1 a** with four equivalents of the stable carbene 1,3‐dimesitylimidazol‐2‐ylidene (IMes) (Scheme 1A). According to the mechanism for the monomeric systems,^[24]^ two equivalents of carbene add to the two electrophilic 6,6′‐positions of the bipyridinium salt, while the additional two equivalents are used as sacrificial base to form the neutral hybrid molecule **2 a**. We observed a clean reaction and could isolate the neutral hybrid molecule **2 a** as highly air sensitive dark violet crystalline solid in 77 % yield. Note, the insoluble protonated carbene byproduct can be reisolated and reused upon deprotonation. In order to demonstrate that this approach is not limited to the 6,6′‐positions of the bipyridine, we also investigated the reactivity of 6,6′‐diarylated‐2,2′‐bipyridinium salt **1 b** (Scheme 1B). In analogy, the reaction of IMes with **1 b** affords **2 b** as dark red solid in 57 % yield.

**Figure 2:**
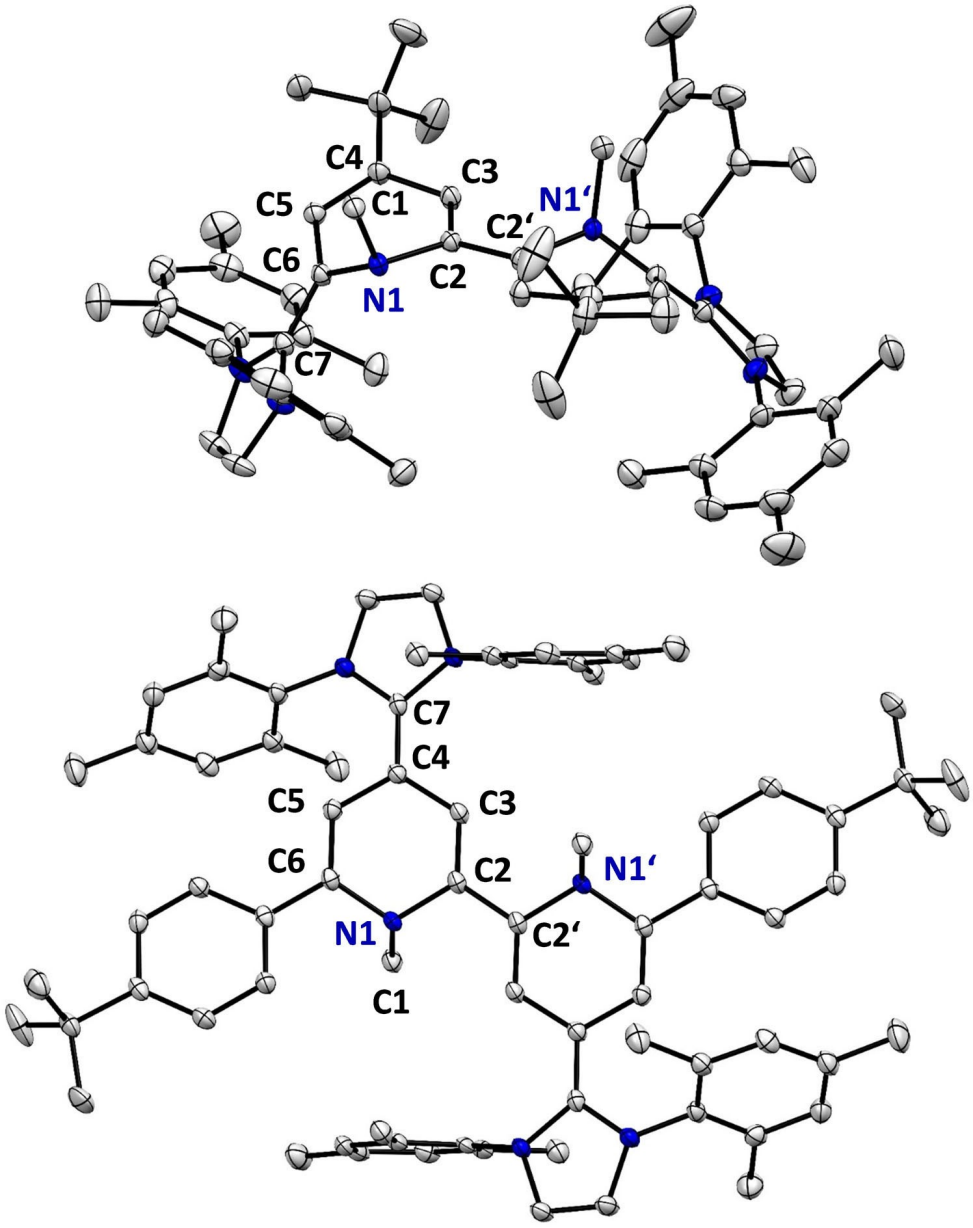
X‐ray solid‐state structures of neutral **2 a** (top) and **2 b** (bottom). Hydrogen atoms and solvent molecules of Et_2_O (**2 a**) and THF (**2 b**) omitted for clarity. Thermal ellipsoids are shown with 50 % probability. Selected bond lengths and angles in [Å]: **2 a**: N1−C2 1.451(2), C2−C3 1.365(2), C3−C4 1.456(2), C4−C5 1.362(2), C5−C6 1.449(2), C6−N1 1.450(2), C2−C2′ 1.463(2), C7−C6 1.369(2). **2 b**: N1−C2 1.451(2), C2−C3 1.354(2), C3−C4 1.445(2), C4−C5 1.443(2), C5−C6 1.346(2), C6−N1 1.436(2), C2−C2′ 1.460(4), C4−C7 1.384(2).

This simple addition/elimination strategy allows to access both types of the 6,6′‐ (**2 a**) and 4,4′‐hybrid molecules (**2 b**) from simple diquat precursors. Due to their sensitivity it is quite common that strongly reducing bispyridinylenes are not spectroscopically nor structurally characterized in their neutral redox state, but are either directly used in situ in organic synthesis^[26]^ or characterized in their oxidized redox state.^[27]^ Indeed, we observed that for **2 a** and **2 b** strictly air‐free handling is necessary to obtain sharp ^1^H and ^13^C NMR signals, which broaden upon slight radical contaminations (see Supporting Information). We could obtain single crystals for both neutral **2 a** and **2 b** and clearly establish their X‐ray solid‐state structures (Figure 2).^[28]^ Both molecular structures are highly distorted,^[29]^ with the N−Me groups being strongly (≈90°) tilted out of the central plane (both up for **2 a** and up and down for **2 b**), clearly indicating a complete loss of aromaticity in both heterocycles. Both **2 a** and **2 b** feature in the central C_5_N cores significant distortions and C−C bond alternations [C2−C3 1.365(2) Å and C3−C4 1.456(2) Å (**2 a**); C2−C3 1.354(2) Å and C3−C4 1.445(2) Å (**2 b**)] confirming a dearomatized core. The NHC‐pyridine C−C bond lengths [C7−C6 1.369(2) Å and C7′−C6′ 1.374(2) Å (**2 a**)] are slightly longer than a regular C=C double bond,^[24]^ while the C2−C2′ bond length [1.463(2) Å (**2 a**)] agrees with a C(sp^2^)−C(sp^2^) single bond [1.47 Å]. The latter should allow free rotation, but is restricted in the N−C−C−N *trans* orientation (170–180°) due to the favorable partial π‐overlap and the sterically demanding N‐mesityl substituents.

**Figure 3:**
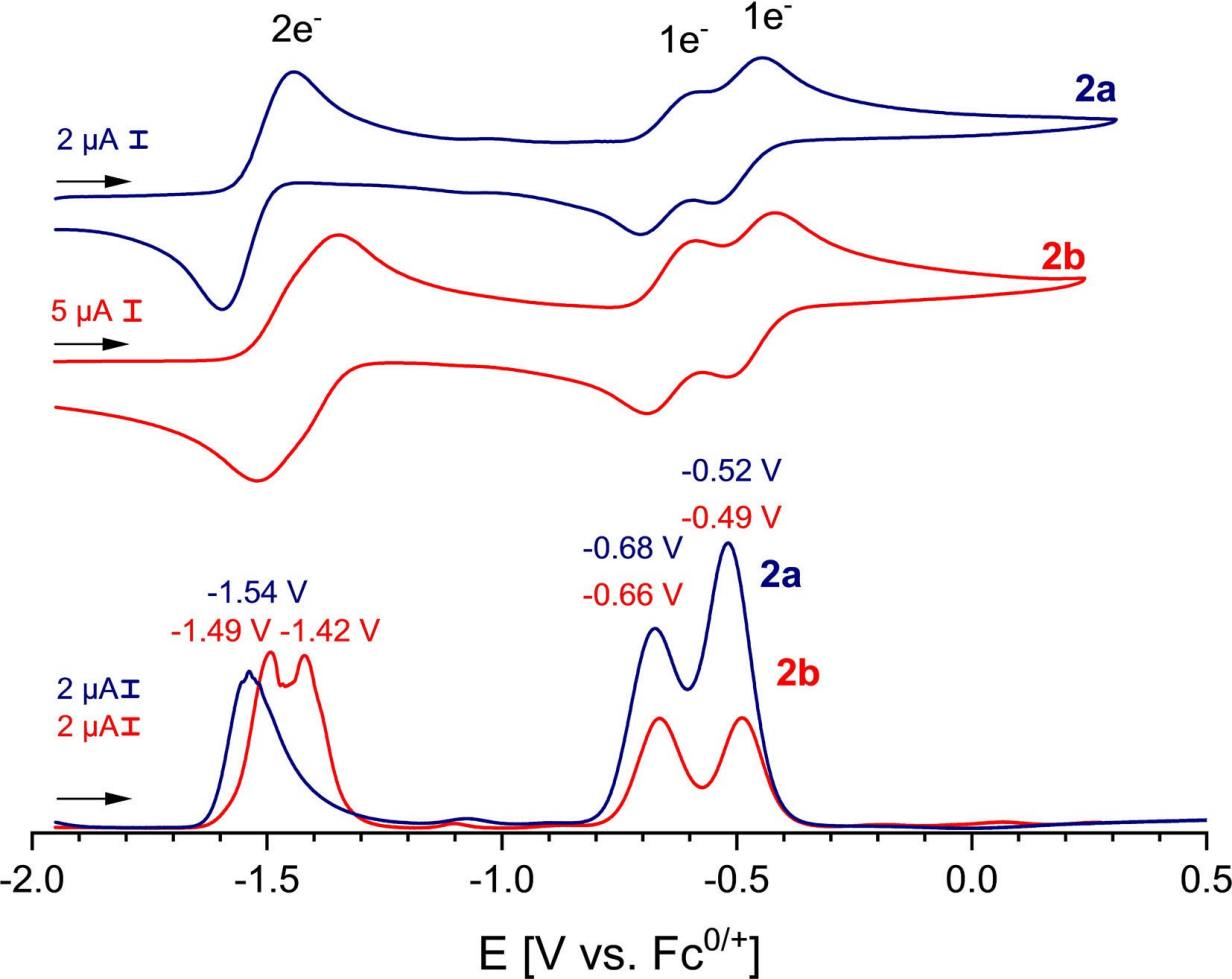
Cyclic voltammograms of **2 a** and **2 b** (0.5±0.1 mg mL^−1^) in THF *n*Bu_4_N^+^PF_6_^−^ (0.1 M), scan rate 200 mV s^−1^ (iR compensation =1800 Ω) referenced against Fc/Fc^+^. Square wave voltammetry of **2 a** and **2 b** (1.6±0.1 mg mL^−1^) in THF (0.1 M *n*Bu_4_N^+^PF_6_^−^). Experimental parameters: iR Compensation=2100 Ω, step size: 1 mV, frequency: 15 Hz, pulse size: 25 mV (arrows indicate scanning direction).

**Scheme 2:**
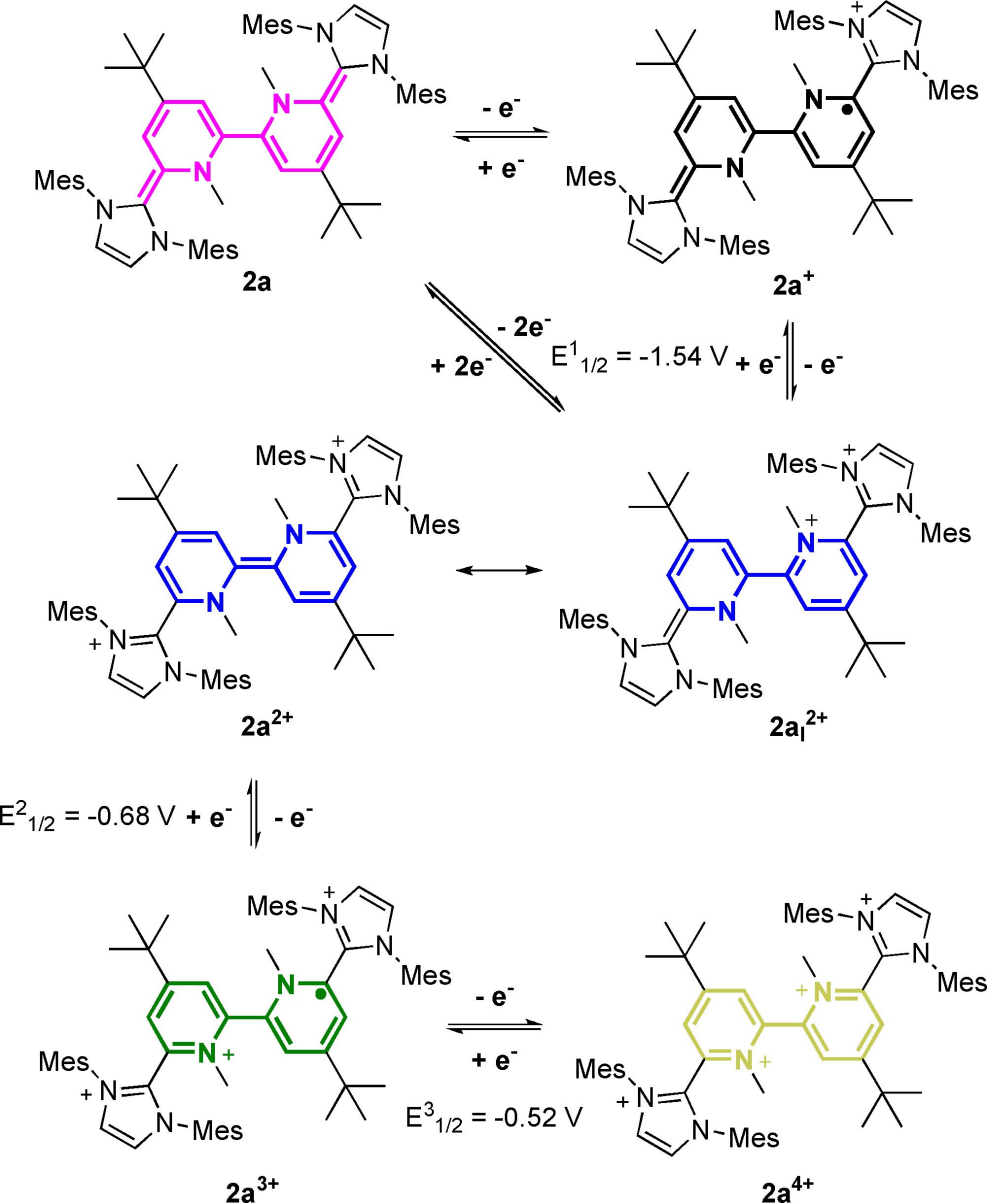
Redox‐states of the 2,2′‐bipyridine hybrid **2 a**. Redox potentials given vs. Fc/Fc^+^.

Next, we investigated the electrochemical properties of **2 a** and **2 b** (Figure 3). The cyclic voltammetry (CV) curve of **2 a** features three quasi‐reversible redox events (four oxidation states): a two‐electron oxidation at a strongly negative redox potential *E*_1/2_=−1.54 V to afford dication **2 a^2+^**, as well as two consecutive one‐electron oxidations at *E*_1/2_=−0.68 V and *E*_1/2_=−0.52 V (vs. Fc/Fc^+^) to give the radical trication **2 a^3+^** and tetracation **2 a^4+^**, respectively (Figure 3; Scheme 2). **2 b** features similar electrochemical properties [*E*_1/2_=−1.44 V (2 e^−^); −0.66 V (1 e^−^) and −0.49 V (1 e^−^)] (Figure 3). The CV curves remain (quasi) reversible also at slow scan‐rates (see Figures S75, S76, S82, S83) and the separation into more than two redox‐events clearly indicates a strong communication between the two redox‐sites. Note, both **2 a** and **2 b** beat Himmel's record of the strongest neutral four‐electron donor in both reduction potentials (see **VIII**; Figure 1).^[23]^

**Figure 4:**
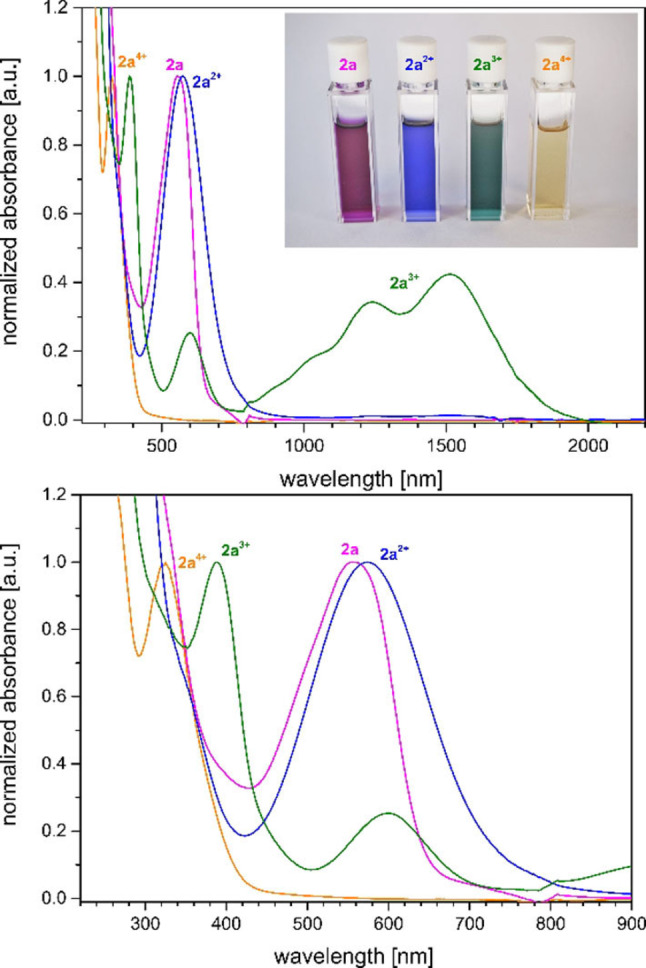
Overlay of normalized UV/Vis‐NIR spectra of the four isolated redox‐states for **2 a** in THF (0.5 mg mL^−1^). Insert: photograph of their optical appearance.

Interestingly, square‐wave voltammetry measurements indicate for **2 b** two consecutive step‐wise one‐electron oxidation steps to give radical **2 b^+^** followed by dication **2 b^2+^** (Δ*E*_1/2_=73 mV), while for **2 a** only a two‐electron oxidation is observed. The normal ordering of redox events for **2 b** in contrast to a potential compression/inversion for **2 a** might be rationalized by the structural changes involved in the oxidation process. Since **2 a** is strongly distorted in a bowl‐shaped fashion an oxidation is likely to feature a larger structural reorganization energy, thereby leading to a potential compression/inversion.^[30]^ Since solvent effects might influence the redox potentials,^[31]^ we also determined for **2 a** and **2 b** CVs in DMF and CH_2_Cl_2_ (Figures S78–S80 and S86–S88), but could not detect a large extrinsic solvent influence. In case of **2 b** the CV data in CH_2_Cl_2_ shows a larger separation of the one vs. two‐electron oxidation peaks (Δ*E*_1/2_=144 mV), however, we also observed the instability of the redox system in CH_2_Cl_2_ in contrast to the high stability in THF. Next, we investigated the stoichiometric oxidation and characterization of all oxidation states. Upon addition of two, three or four equivalents of AgSbF_6_ to **2 a** (and **2 b**) all oxidation states are cleanly accessible and stable, though air‐sensitive solids (vide infra; for the data of the redox‐series of **2 b**, see the Supporting Information). Alternatively, the redox states are accessible in situ via UV/Vis spectroelectrochemistry, which match with the UV/Vis data of the isolated compounds (see Figures S119–S121). As expected **2 a** (and **2 b**) features electrochromic properties with strong UV/Vis absorptions for the neutral (*λ*=553 nm; *ϵ*=28 712 cm^−1^ M^−1^), dication (*λ*=574 nm; *ϵ*=18 620 cm^−1^ M^−1^) and trication (*λ*=602 nm; *ϵ*=5547 cm^−1^ M^−1^), while the tetracation is mostly UV‐active (Figure 4).

**Figure 5:**
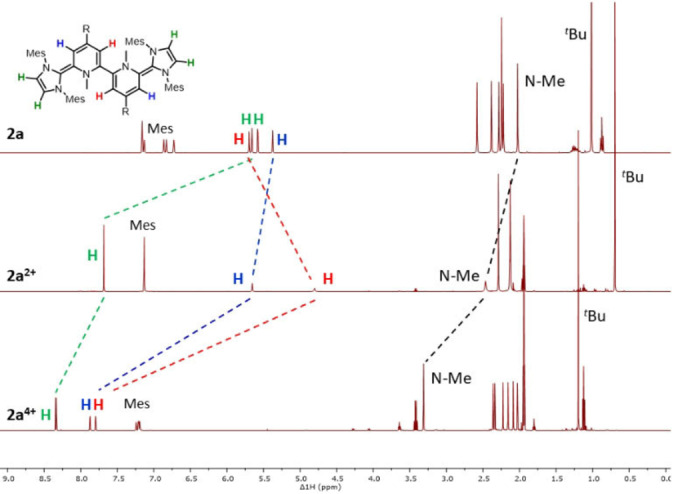
Shift of ^1^H NMR signals from **2** (top; C_6_D_6_), **2^2+^** (middle; CD_3_CN) to **2^4+^** (bottom; CD_3_CN).

The absorption spectra also match very well with the predicted TD‐DFT transitions (see Figures S133–141). Interestingly, the radical trication **2 a^3+^** features two intense NIR absorptions at *λ*=1233 nm and 1513 nm (*ϵ*≈7000–9000 cm^−1^ M^−1^) (vide infra). In case of **2 b** the radical trication **2 b^3+^** also features broad NIR absorptions shifted to even longer wavelengths *λ*=1401 nm and 1698 nm (*ϵ*≈5000 cm^−1^ M^−1^) (Figure S111). Since the CV data indicated the possibility to generate radical monocation **2 b^+^** (Δ*E*_1/2_=73 mV) as compound in equilibrium with its disproportionation products, we also attempted its spectroscopic detection. Note, Δ*E*_1/2_=73 mV relates according to Δ*E*_1/2_=0.059⋅log *K*_rad_ to a rather small radical formation constant of *K*_rad_=17.3.^[32]^ One‐electron oxidation of **2 b** leads to a strong NIR absorption at λ=1367 nm (Figures S72, S73) which we assign to the radical monocation **2 b^+^** and which is additionally supported by a broad X‐band EPR signal (see Figure S71). Note, analogous experiments with **2 a** only resulted in disproportionation into **2 a** and **2 a^2+^** as expected based on the CV/SWV data (see Supporting Information). The formation of the diamagnetic compounds, dication **2 a^2+^** and tetracation **2 a^4+^**, can be monitored by NMR spectroscopy (Figure 5). In the ^1^H NMR spectrum of neutral **2 a** the bipyridine core as well as the imidazole protons appear in an olefinic region (*δ*=5.5–6.0 ppm) (Figure 5 top). Interestingly, upon two‐electron oxidation the imidazole protons are shifted downfield (*δ*=7.68 ppm) while the H‐atoms of the bipyridine core are broadened but remain in the olefinic region (Figure 5 middle). This finding strongly supports a resonance structure with two aromatic imidazolium heterocycles and a dearomatized bispyridinylene core (**2 a^2+^**; Scheme 2).

**Figure 6:**
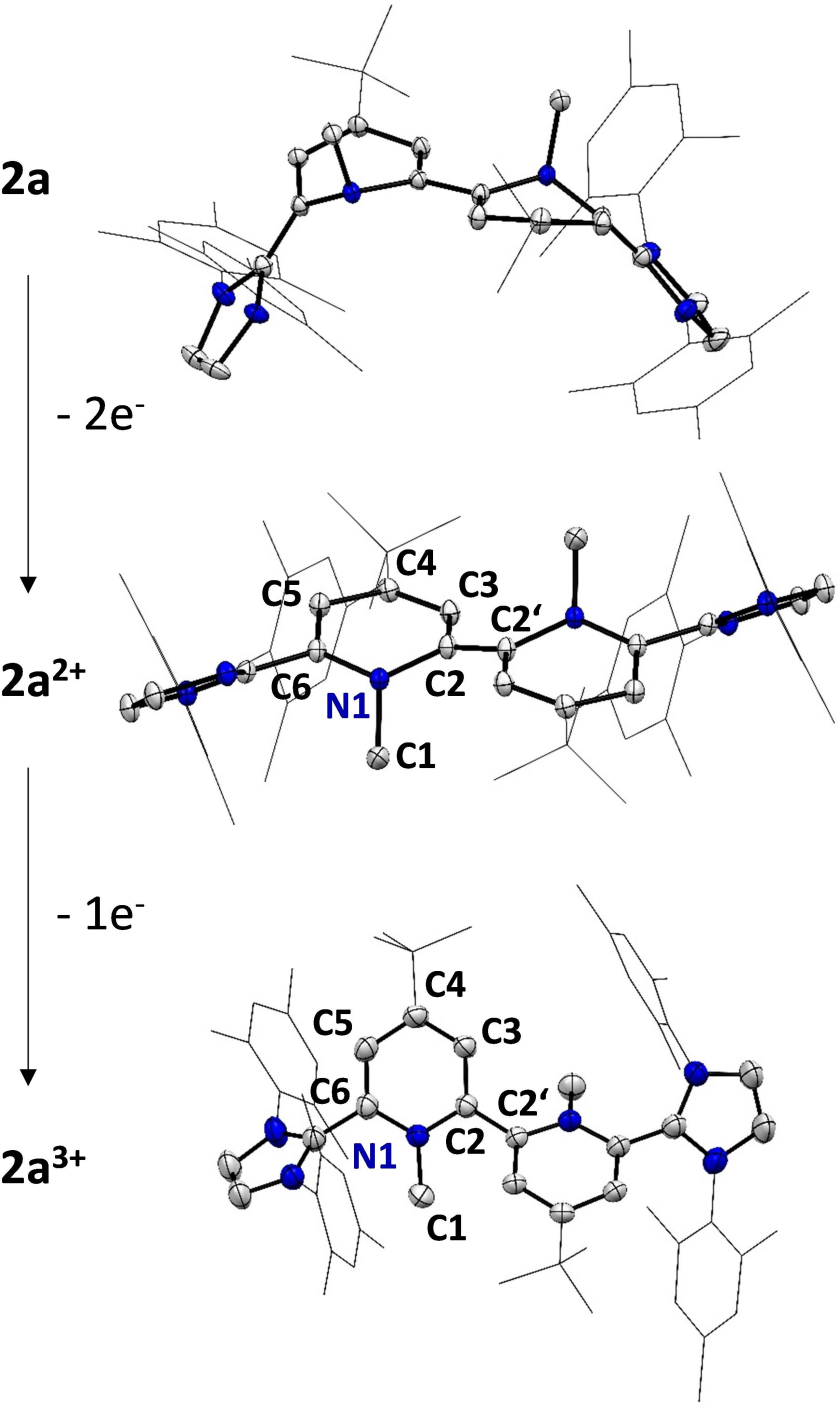
Comparison of X‐ray solid‐state structures of the **2 a** redox series: neutral **2 a** (top), dication **2 a^2+^** (middle) and radical trication **2 a^3+^** (bottom). Hydrogen atoms, solvent molecules (Et_2_O) and counter anions (SbF_6_^−^) omitted for clarity. Thermal ellipsoids are shown with 50 % probability. Selected bond lengths and angles in [Å]: **2 a^2+^**: N1−C2 1.437(2), C2−C3 1.449(2), C3−C4 1.358(2), C4−C5 1.447(2), C5−C6 1.354(2), C6−N1 1.395(1) C2−C2′ 1.373(2). **2 a^3+^**: N1−C2 1.381(4)/1.393(4), C2−C3 1.398(5)/1.397(4), C3−C4 1.374(5)/1.378(4), C4−C5 1.413(5)/1.402(5), C5−C6 1.352(5)/1.363(5), C2−C2′ 1.442(4).

The synthesis of quinoidic, dearomatized bispyridinylene heterocycles (**2 a^2+^**) by oxidation is unprecedented, since bispyridinylenes are typically strong reductants prone to aromatize to the bipyridinium salts.^[7]^ Note, non‐tethered bispyridinylenes exist typically as mixture of *E*/*Z* isomers.^[7]^ NMR data of **2 a^2+^** exclusively shows, in analogy to **2 a**, only one (N−C−C−N) *E*‐isomer due to the steric bulk given by the carbene entities. Upon oxidation of **2 a^2+^** to the tetracation **2 a^4+^** the bispyridinylene protons shift into the typical aromatic bipyridinium range (*δ*≈7.8 ppm) (Figure 5 bottom). Thereby, the NMR data supports a stepwise oxidation process with increasing aromaticity: from an initial neutral redox‐state with no aromaticity, to two aromatic imidazolium heterocycles (**2 a^2+^**), followed by two bipyridinium heterocycles (**2 a^4+^**), compensating the increasing charge. In order to obtain information on the structural changes involved throughout the oxidation process we obtained single crystals suitable for X‐ray diffraction of dication **2 a^2+^** and the radical trication **2 a^3+^** (Figure 6).^[28]^

**Figure 7:**
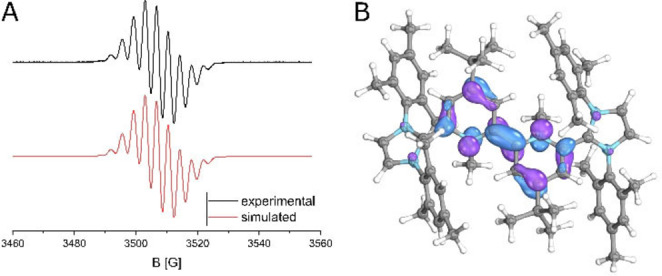
A) X‐band EPR spectrum of **2 a^3+^** in THF. Simulated hfcs: 2×N: 10.52 MHz (calc. 11.4 MHz); 4×N: 1.91 MHz (calc. 1.3 MHz); 6×H: 9.8 MHz (calc. 8.3 MHz); g=2.0030; LW 0.167; for calculations see Figure S122. B) SOMO of **2 a^3+^** (B3LYP‐GD3BJ/def2TZVP) with an isovalue of 50 %.

As suggested based on the CV data, the largest reorganization takes place in the first two‐electron oxidation events. From **2 a** to **2 a^2+^** the central C2−C2′ bond shortens [1.460(4) Å (**2 a**) vs. 1.373(2) Å (**2 a^2+^**)], while the bond alternations [C2−C3 1.365(2) Å, C3−C4 1.456(2) Å (**2 a**); C2−C3 1.449(2) Å, C3−C4 1.358(2) Å (**2 a^2+^**)] remain, but flip positions as indicated by the Lewis‐structures (Scheme 2). The X‐ray data supports the NMR data of a localized dearomatized bispyridinylene core flanked by two cationic imidazolium heterocycles. Interestingly, while **2 a** and **2 a^2+^** are perfectly *trans* (N−C−C−N) aligned, the radical trication **2 a^3+^** features two pyridine planes tilted by 138°. Additionally, the two central C_5_N cores are nearly perfectly planar and the C−C bond alternations are far less pronounced indicating aromatic stabilization in the bipyridine core. The pyramidalization of the N−Me groups out of the heterocyclic core follows the clear order **2 a**>**2 a^2+^** >**2 a^3+^** [sum of angles at N: 320° (**2 a**) vs. 350° (**2 a^2+^**) and 359° (**2 a^3+^**)]. Since **2 a^3+^** and **2 b^3+^** feature an open‐shell ground‐state the question arises whether the radical is localized on one pyridine‐hybrid entity (redox center) with a fast exchange to the other redox center, or is fully delocalized. According to the Robin‐Day classification of mixed valence systems,^[33]^ this would translate to either two weakly interacting redox‐sites (class II) or a strongly coupled fully delocalized system (class III).^[34]^ The intense NIR absorptions suggests an intervalence charge transfer (IV‐CT) band supporting a new class of organic mixed valence system. X‐ray diffraction shows for **2 a^3+^** a rather symmetrical structure, which however might be a result of an averaging due to alternate packing. The X‐band EPR spectrum also indicates a symmetrical electron distribution which agrees very well with the hyperfine coupling constants (hfcs) derived from DFT calculations (Figure 7). Given the short distance between the two redox centers the system is very likely a class III organic mixed valence system, which is also supported by DFT calculations featuring a delocalized single occupied molecular orbital (SOMO) over the central bipyridine core (Figure 7B).

**Scheme 3:**
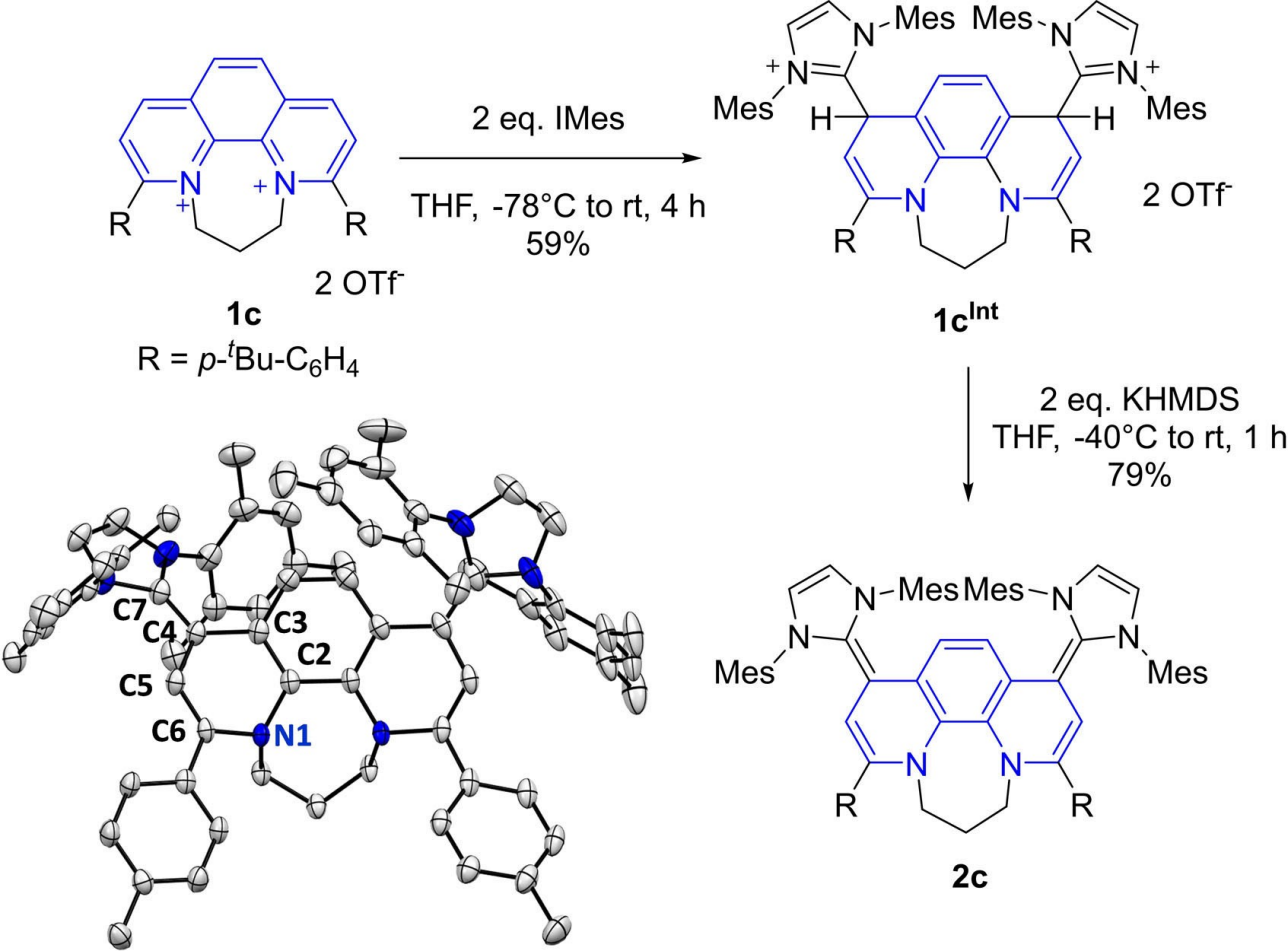
Stepwise synthesis of **2 c**. Left: X‐ray solid‐state structure of neutral **2 c**. Hydrogen atoms are omitted for clarity. ^*t*^Bu groups are simplified by Me groups. Thermal ellipsoids are shown with 50 % probability. Selected bond lengths and angles in [Å]: N1−C2 1.438(4), C2−C3 1.406(4), C3−C4 1.468, C4−C5 1.442(4), C5−C6 1.341(4), C6−N1 1.439(4), C4−C7 1.381(4).

We were intrigued by the large structural reorganization in the 2,2′‐bipyridine systems upon oxidation. In order to investigate the effect of distortion and to increase electronic communication, we analyzed the effect of a rigid central core and selected alkylated phenanthrolinium salt **1 c** (Scheme 3). Initial attempts to add **1 c** to four equivalents carbene generated the desired hybrid molecule **2 c** as main product, however, the clean isolation and characterization was challenging due to contamination with minor radical impurities. As a result, we investigated a stepwise addition/elimination process. Addition of two equivalents of IMes to **1 c** generated the clean double 4,7‐addition product **1 c^Int^** which could be isolated as single diastereomer in 59 % yield. Upon addition of two equivalents KHMDS **2 c** was cleanly formed and isolated in 79 % yield.

Note, this strategy reduces the amount of free carbene, additionally, indicates that the formation of the hybrid molecules proceeds through a double addition followed by a double elimination mechanism. The solid‐state structure of neutral **2 c** indicates a distorted geometry with a strongly tilted (*syn*) N−propyl−N linker with two pyramidalized N‐atoms, C−C bond alternations [C4−C5 1.442(4) Å vs. C5−C6 1.341(4) Å] and a nearly planar C_6_ central ring (Scheme 3).^[28]^ In agreement with the distorted molecular structure the ^1^H NMR data agrees with localized double bonds [^1^H NMR; δ: 5.47 ppm (2*H*) and 5.45 ppm (2*H*)] for the four protons of the central phenanthroline core. Temperature dependent NMR data indicates a strongly dynamic behavior (Figure S55; see Supporting Information).

**Figure 8:**
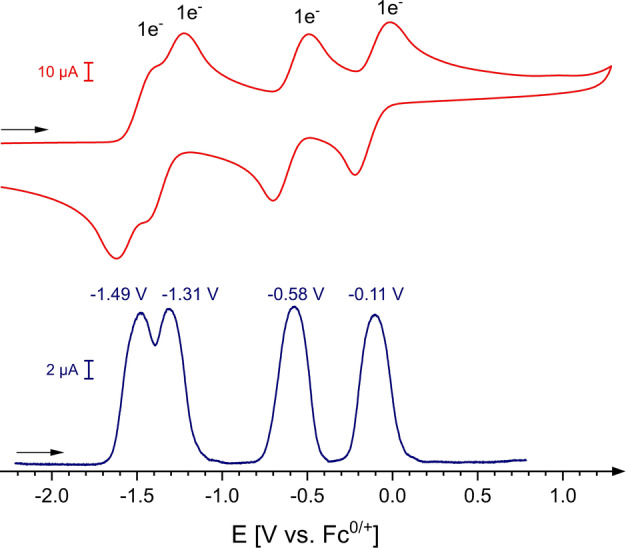
Cyclic voltammograms of **2 c** (0.8±0.1 mg mL^−1^) in THF (0.1 M *n*Bu_4_N^+^PF_6_^−^); scan rate 200 mV s^−1^ (iR compensation=2000 Ω) referenced against Fc/Fc^+^ (top) and square‐wave voltammogram (bottom) in THF (0.1 M *n*Bu_4_N^+^PF_6_^−^); iR Compensation=2170 Ω, step size: 1 mV, frequency: 15 Hz, pulse size: 25 mV.

**Scheme 4:**
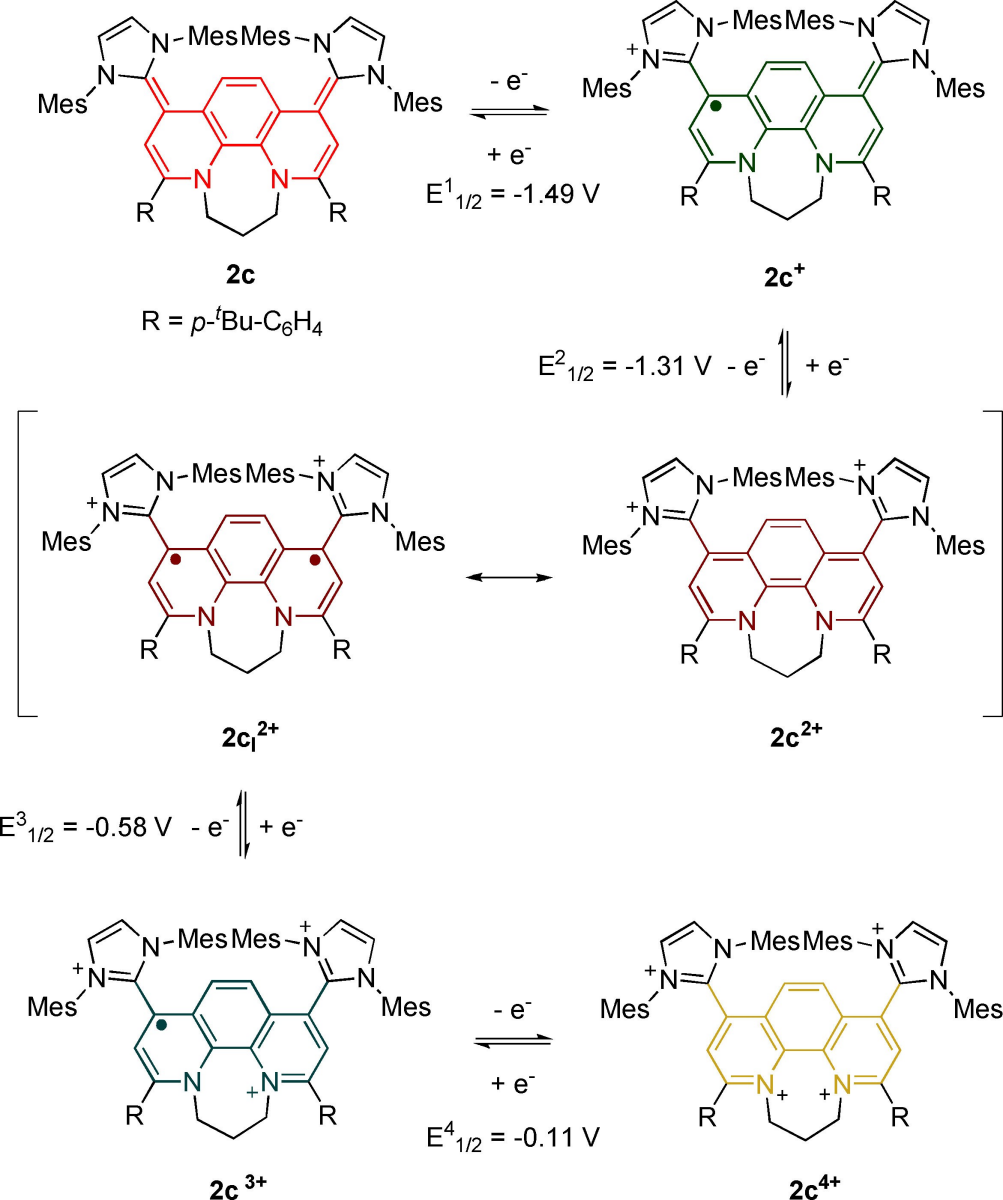
Five stable oxidation states of redox system **2 c**. Redox potentials are given vs. Fc/Fc^+^.

Interestingly, the cyclic voltammogram of **2 c** shows four reversible redox events (five stable oxidation states) at *E* ^1^_1/2_=−1.49 V (**2 c^+^**); *E* ^2^_1/2_=−1.31 V (**2 c^2+^**); *E* ^3^_1/2_=−0.58 V (**2 c^3+^**) and *E* ^4^_1/2_=−0.11 V (**2 c^4+^**) (Figure 8 and Scheme 4), which is supported by the corresponding square‐wave measurement. No large solvent dependency was observed with five well‐defined redox states also detected in CH_2_Cl_2_ and DMF (see Figures S92–S94). Note, simple 1,10‐phenanthrolinium salts typically feature irreversible CVs with only a one‐electron reduction event.^[35]^ Compared with **2 a**/**2 b** the redox potentials for the oxidation to the radical cation (**2 c^+^**) and tetracation (**2 c^4+^**) are shifted towards more positive potentials, indicating a larger degree of communication between the two redox‐sites. In agreement with a rigid central core, the structural changes upon oxidation should be less pronounced leading to a potential expansion confirmed by the CV data.

**Figure 9:**
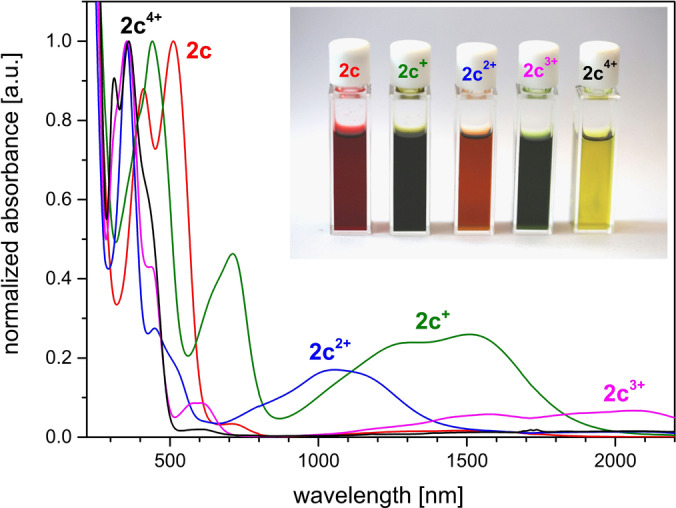
Overlay of the normalized UV/Vis spectra of the isolated redox‐states of **2 c**. Insert: photograph of their optical appearance.

Since the electrochemical data suggests the feasibility to access all five oxidation states we investigated the stoichiometric synthesis by stepwise oxidation with the appropriate amount of oxidant (AgSbF_6_). Again, in situ UV/Vis spectroelectrochemistry is in good agreement with the UV/Vis data of the isolated oxidation states (Figure 9; for UV/Vis SEC see Figure S122) as well as with the TD‐DFT predicted transitions (see Figures S142–146). Most strikingly, the open‐shell systems (**2 c^+^** and **2 c^3+^**) feature intense NIR absorptions (**2 c^+^**: *λ*=1285 nm and 1520 nm; *ϵ*≈4000 cm^−1^ M^−1^; **2 c^3+^**: *λ*=1573 nm and 2071 nm; *ϵ*≈3000 cm^−1^ M^−1^) as expected for IV‐CT transitions in organic mixed valence systems.

**Figure 10:**
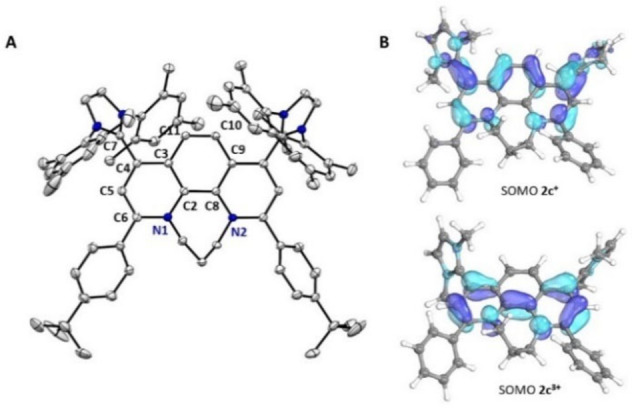
A) X‐ray solid‐state structure of radical trication **2 c^3+^**, hydrogen atoms, three counter‐anions (OTf^−^) and solvent molecules (CH_2_Cl_2_ and Et_2_O) are omitted for clarity. B) Calculated SOMOs (B3LYP‐GD3BJ/def2svp//B3LYP‐GD3BJ/def2TZVP) of the simplified radical cation **2 c^+^** (top) and radical trication **2 c^3+^** shown with isovalues of 50 % (bottom).

We were able to obtain single‐crystals suitable for X‐ray diffraction of the radical trication **2 c^3+^** (Figure 10A). The imidazolium entities are rotated out of a slightly distorted phenanthroline plane (N−C−C−C 55° and 63°). The propyl N−CH_2_CH_2_CH_2_−N linker is positioned *anti* in contrast to the *syn*‐conformation in the neutral redox state (Scheme 3) in good agreement with DFT predictions (see Supporting Information). The X‐band EPR spectra of both radicals **2 c^+^** and **2 c^3+^** are only intense broad waves without resolved nitrogen or proton hyperfine couplings (Figures S97, S98). DFT calculations predict the SOMO of the radical **2 c^+^** to be distributed over the entire π‐system including the imidazolium entities, while the SOMO of radical **2 c^3+^** is primarily centered on the bipyridine fragment similar to **2 a^3+^** (Figure 10B).

**Figure 11:**
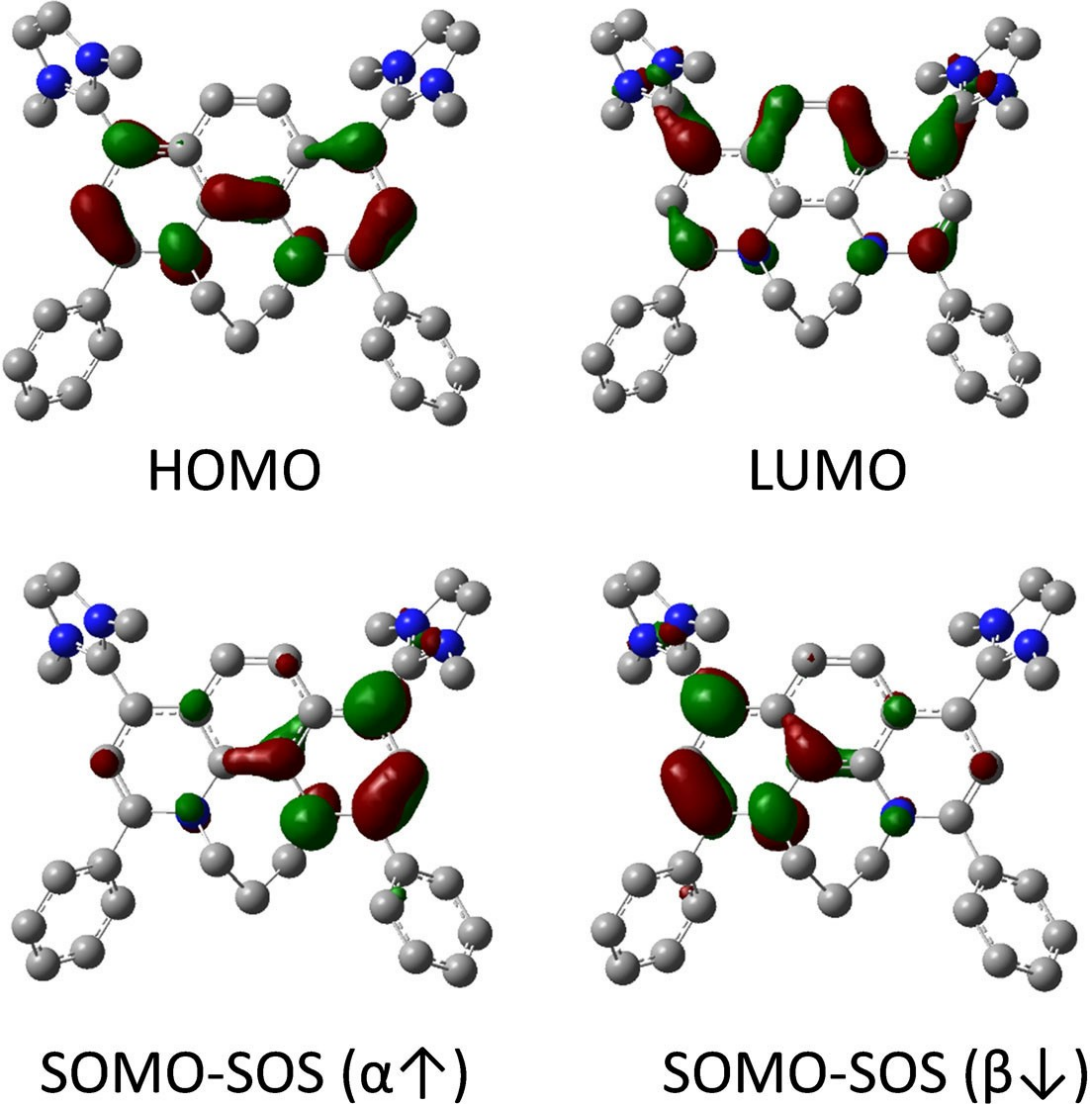
Frontier molecular orbitals (HOMO and LUMO) of the closed shell state of **2 c^2+^** (top) and SOMOs of the singlet open shell state (SOS) (bottom) at the (U)CAM‐B3LYP(BS)/6‐31G* level of theory. Isovalues shown with 0.05.

The electronic structure of **2 c^2+^** appears particularly intriguing since a quinoidic structure inhibits aromatization of the central C_6_ core. Additionally, 16 π‐electrons would predict antiaromatic character.^[14]^ In fact, **2 c^2+^** can be represented as quinoid (**2 c^2+^**) or as Kekulé‐diradical (**2 c_I_** ^**2+**^) (Scheme 4). ^1^H NMR measurements at room temperature show broad signals for the central core [*δ*(d_8_‐thf): 5.28 ppm (middle ring) and 4.03 ppm (left/right rings)], which give sharp ^1^H NMR signals upon cooling to −60 °C (see Figures S58–S59), supporting a singlet ground state.^[36]^ Interestingly, **2 c^2+^** features a NIR absorption [*λ*=1041 nm (*ϵ*=5325 cm^−1^ M^−1^), Figure S115] consistent with a partial diradical character. In fact, calculations at the UCAM‐B3LYP(BS)/6‐31G* and B3LYP(BS)/6‐31G** level of theory employing the broken‐symmetry (BS) formalism indicate for the simplified system a singlet open shell ground state with a small S/T gap of ca. 1–2 kcal mol^−1^ with the singlet closed shell being the highest in energy (ca. 2–5 kcal mol^−1^). SOMOs (α and β) for the open shell singlet state are centered on the two outer rings of the phenanthroline system (Figure 11).

Note, BS‐DFT calculations might overestimate the stabilization of the open‐shell state and disfavor the closed shell state. Nevertheless, the singlet closed and open shell state as well as the triplet state appear close in energy rendering **2 c^2+^** potentially interesting for novel open‐shell diradical applications.^[37]^ We also conducted nuclear independent shifts (NICS)^[38]^ calculations on a simplified phenanthroline system as a function of the oxidation state (see Table S5). The most positive (least aromatic) NICS values are obtained in the dicationic oxidation state (**2 cS^2+^**). Upon oxidation to the tetracation the NICS values strongly indicate aromatic stabilization compensating the highly positive charge.

Finally, we investigated the stabilities of the redox‐systems for potential applications as novel anolyte materials for redox‐flow batteries. In contrast to one‐electron cycling, multiple electron transfers are often problematic due to instabilities or a large undesired separation of the two redox potentials.[^17c^, ^39^] Note, viologen anolyte materials are typically only cycled by one electron over the dication/radical cation oxidation states.^[40]^ More recently there has been interest in harnessing two electrons from the viologen system,[^41^, ^42^] or finding other multi‐electron redox systems at highly negative or positive redox potentials.[^43^, ^44^, ^45^] Note, simple pyridinium/carbene hybrid molecules such as **V** (Figure 1) show significant decomposition after 25 cycles for two‐electron cycling in the symmetrical H‐cell.^[24a]^

**Figure 12:**
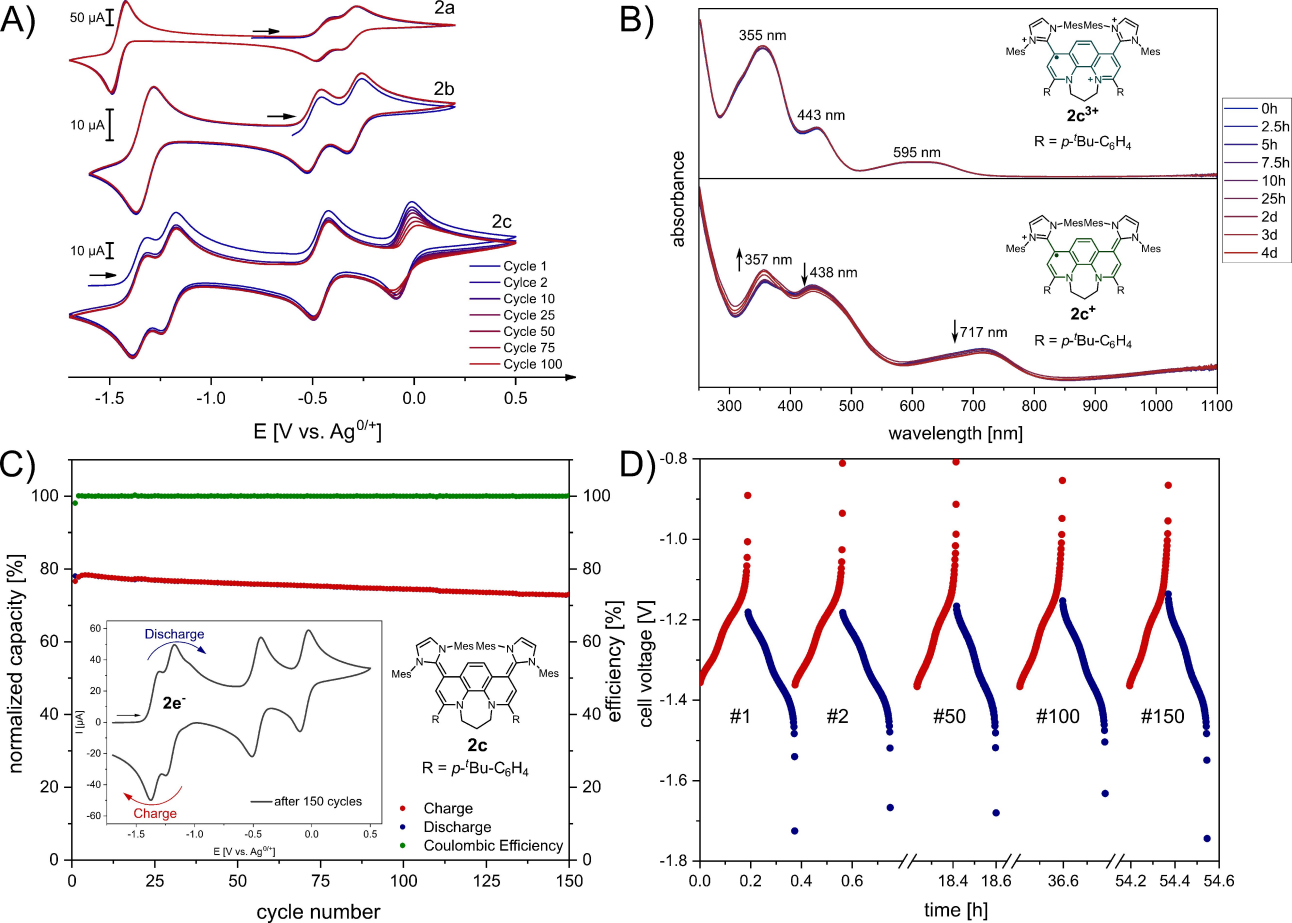
A) Stability tests of the three redox‐systems **2 a**–**2 c** determined by repetitive CV cycling in acetonitrile (200 mV s^−1^; **2 a^2+^** 3.5±0.2 mM; **2 b^2+^** 1.3±0.2 mM and **2 c** 1.8±0.2 mM); B) Time‐dependent UV/Vis spectroscopy of radicals **2 a^+^** and **2 a^3+^** in acetonitrile. C) Capacity versus cycle number and Coulombic efficiency for two‐electron cycling of **2 c/2 c^2+^** (2.5 mM in 0.5 M TBAPF_6_/acetonitrile) in a symmetrical H‐cell for 150 cycles; inset CV after 150 cycles. D) Voltage profile for the charge/discharge process.

To assess the electrochemical stability, we performed repetitive CV measurements for **2 a**–**2 c** (Figure 12A). Over 100 cycles no significant change in the current of the cyclic voltammogram was observed indicating promising electrochemical stabilities for redox systems **2 a** and **2 b**. In case of **2 c** the first three redox states (**2 c**–**2 c^3+^**) show high stability but the current of the tetracation slowly decays with increasing cycle number (Figure 12A). We initially attributed this decrease to the high Lewis‐acidity of **2 c^4+^** leading to a potential fluoride abstraction of the PF_6_^−^ anion of the *n‐*Bu_4_NPF_6_ electrolyte.^[46]^ Since isolated tetracation **2 c^4+^** in acetonitrile shows high stability over several months (Figure S129) as well as over several hours in the presence of *n*‐Bu_4_NPF_6_ electrolyte (Figure S130) the fading seems more likely attributed to a decomposition on the electrode surface resulting in an electrode fouling process.^[47]^ Indeed, cleaning the working electrode surface after repetitive cycling restores the initial cyclic voltammogram. We then determined the solubilities of selected redox‐states (see Supporting Information): Redox system **2 c^2+^** features good solubility in acetonitrile (>15 mM) and rather low (≈3 mM) solubility in the neutral redox state (see Supporting Information). In general, all oxidation states for **2 a**–**2 c** show high stabilities exceeding days and even months if kept under inert conditions (see Supporting Information). For instance, **2 c** and **2 c^2+^** are stable in solution with no change in the NMR spectra over several months (Figure S127, S128), while the radicals (**2 c^+^** and **2 c^3+^**) are stable over several days under inert conditions (Figure 12B). Radical **2 c^3+^** is even stable open to air over an extended period of time (>6 h).

In order to test if this high persistence translates to stable electrochemical cycling, **2 c** was selected due to its increased solubility over **2 a** and **2 b** as two‐electron storage material for charge–discharge studies. We performed two‐electron charge–discharge cycling of a 2.5 mM solution (**2 c** vs. **2 c^2+^**) in 0.5 M TBAPF_6_/acetonitrile in a symmetrical H‐cell under galvanostatic charging (2*C*) with reticulated vitreous carbon (RVC) electrodes and voltaic cutoffs of −0.8 V and −1.8 V. The utilization of a symmetrical H‐cell is an established procedure to determine long‐term cycling stabilities.^[48]^ The first charge was performed with a high capacity of ca. 80 % of the theoretical capacity and proved to show very high stability with only ca. 5 % capacity loss over 150 cycles while maintaining very high >99 % Coulombic efficiency (Figure 12C; for C‐rate dependency see Figure S132). The voltage profile is consistent with the CV data and indicates a nearly constant plateau for the two‐electron transfer with no new redox active compounds appearing over 150 cycles (Figure 12D). Additionally, we observed no significant change in the CV of the solution after the 150 charge/discharge cycles (ca. 55 h) (inset Figure 12C). In conclusion, **2 c** shows very high electrochemical stability for two‐electron cycling at a stable highly negative redox plateau, even more negative than the second reduction potential of viologen. Future work will address the increase of solubilities and to decrease the molecular weight, which is currently under investigation.

## Conclusion

In summary, we report a simple synthetic strategy to transfer well‐known diquat and phenanthroline two‐electron redox systems into novel strongly coupled four‐electron redox systems. While the few reported organic multi‐electron (>two‐electrons) redox systems are typically quite tedious in their preparation and do not allow straightforward electronic manipulation, the here described synthesis is highly modular. Since there are plenty of well characterized 2,2′‐bipyridinium and phenanthrolinium salts with various linker elements as well as plenty of carbenes in various sizes available, the outlined concept should allow a rapid synthesis of a large library of (super) multi‐electron donors. This should enable fine‐tuning of the redox‐, and optical properties of the four‐electron redox system for various applications. In particular the open‐shell diradical as well as mixed valence compounds could be altered and diradical properties enhanced by choosing appropriate carbene entities. Additionally, we analyzed in detail the structural changes involved in the oxidation process and relate them to potential compression or expansion processes. It should be pointed out that these systems beat the previous record^[23]^ of the strongest organic four‐electron donor. The increase in charge density by utilizing multi‐electron processes renders the systems also as attractive candidates for battery applications. Indeed, we could demonstrate by galvanostatic charge/discharge studies that such redox systems show very high stability for the two‐electron storage at highly negative redox potentials. These findings pave the way for new multi‐redox lead structures for energy storage applications such as non‐aqueous redox flow batteries.

## Conflict of interest

The authors declare no conflict of interest.

1

## Supporting information

As a service to our authors and readers, this journal provides supporting information supplied by the authors. Such materials are peer reviewed and may be re‐organized for online delivery, but are not copy‐edited or typeset. Technical support issues arising from supporting information (other than missing files) should be addressed to the authors.

Supporting Information

## Data Availability

The data that support the findings of this study are available in the Supporting Information of this article.
