# Supplementary material for: Organic Four‐Electron Redox Systems Based on Bipyridine and Phenanthroline Carbene Architectures
Source: Angew Chem Int Ed Engl. 2022 Apr 12;61(24):e202203064. doi: 10.1002/anie.202203064 (PMC9325510; doi:10.1002/anie.202203064)
Supplement: Supplementary file 1 — Supporting Information [file ANIE-61-0-s001.pdf]

Angewandte Chemie
Eine Zeitschrift der Gesellschaft Deutscher Chemiker

Supporting Information

Organic Four-Electron Redox Systems Based on Bipyridine and Phenanthroline Carbene Architectures

*P. W. Antoni, C. Golz, M. M. Hansmann**

Author Contributions

P.A. performed the synthesis and characterization, C.G. the crystallographic investigation. Calculations were performed by P.A. and M.M.H. The project was designed and the manuscript was written by M.M.H.

Table of Contents

1. Materials and methods S-3
2. Synthetic procedures S-4
3. NMR Spectra S-20
4. Attempted preparation of radicals **2a**$^+$ and **2b**$^+$ S-56
5. X-ray characterization data S-59
6. Electrochemical measurements S-62
7. EPR Spectroscopy S-75
8. UV-vis-NIR spectroscopy S-77
9. UV-vis-NIR spectroelectrochemistry S-90
10. Solubility and stability measurements S-94
11. H-cell charge/discharge experiments S-99
12. Computational data S-101
13. References S-135

1. Materials and methods

All solvents were purified with a MBraun SPS – 800 over molecular sieves and degassed with argon. Pyridine was distilled over CaH_2 and degassed with three freeze-pump-thaw-cycles. Reactions were carried out either under N_2 or Ar atmosphere. Solids were handled and NMR samples were prepared in a nitrogen filled glovebox. High resolution MS (EI): Finnigan MAT 8200 (70 eV), ESIMS: Finnigan MAT 95, accurate mass determinations: Bruker APEX III FT-MS (7 T magnet) and LTQ-Orbitrap-XL (Thermo Scientific) equipped with a heated electrospray ionization source (HESI). NMR: NMR spectra were measured on the spectrometers Bruker AV 500 Avance NEO, Bruker AV 400 Avance III HD NanoBay, AV 600 Avance III HD and AV 700 Avance III HD and chemical shifts (δ) are referenced to their solvent signals [C_6D_6, 7.16 (^{1}H-NMR) 128.06 (^{13}C-NMR); CD_3CN, 1.94 (^{1}H NMR) 118.26 (^{13}C NMR), $CDCl_3$ 7.26 (^{1}H NMR) 77.16 (^{13}C NMR), CD_2Cl_2, 5.32 (^{1}H NMR) 54.00(^{13}C NMR), d_8-thf, 3.58 (^{1}H NMR) 67.57(^{13}C NMR)], coupling constants (J) in Hz. All spectra were recorded in 5mm NMR tubes at the temperatures indicated. X-Band EPR experiments were performed using a Bruker EMX-Nano Benchtop spectrometer or a Bruker EMX Mikro with an EMX premium bridge. Simulations were performed with the EasySpin program.[1] UV-vis spectra were recorded on an Agilent Cary5000 or Cary60. Flash chromatography was performed with Merck 60 silica gel (40-63 µm). Thin-layer chromatography (TLC) analysis was performed using Merck silica gel 60 F254 TLC plates and visualized by UV irradiation and/or ceric ammonium molybdate, $KMnO_4$ or p-anisaldehyde. All commercially available compounds (Acros, abcr, Alfa Aesar, Sigma Aldrich, Fluorochem) were used as received. IR-ATR measurements (diamond) were performed in reflection mode on a Bruker Alpha II inside a glovebox, wavenumbers in cm^{-1}. Melting points were measured with a Büchi M-560 apparatus. Free IMes was synthesized by deprotonation of the imidazolium-BF_4 salt in thf employing NaH as base, in combination with a catalytic amount KO^tBu.[2]

2. Synthetic procedures

Synthesis of **1a**

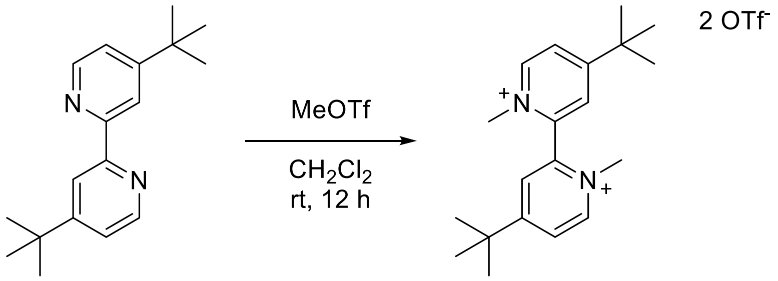

To a solution of 4,4'-di-*tert*-butyl-2,2'-bipyridine (1.00 g, 3.73 mmol, 1.0 eq.) in CH_2Cl_2 (40 mL) was added MeOTf (0.9 mL, 1.35 g, 8.20 mmol, 2.2 eq.) in one portion. The mixture was stirred for 12 h at room temperature, leading to the formation of a colorless precipitate. The amount of solvent was reduced to ~5 mL under reduced pressure, and the slightly cloudy solution poured into Et_2O (100 mL) under vigorous stirring, furnishing a voluminous colorless precipitate. The crude product was filtered off and washed twice with Et_2O (3 x 50 mL). Drying under reduced pressure yielded **1a** (2.16 g, 3.62 mmol, 97%) as colorless solid.

Note: MeOTf is highly cancerogenic and toxic upon inhalation or skin contact, the use of appropriate personal safety equipment is strongly advised!

m.p. 194 °C; **^{1}H NMR** (500 MHz, CD_3CN): δ [ppm] = 8.89 (d, *J* = 7.5 Hz, 2H, py-C*H*), 8.30 – 8.25 (m, 4H, py-C*H*), 4.04 (s, 6H, N-CH_3), 1.43 (s, 18H, C(CH_3)$_3$); **^{13}C NMR** (126 MHz, CD_3CN): δ [ppm] = 174.0 (py-C_q), 149.1 (py-*C*H), 143.9 (py-C_q), 129.4 (py-*C*H), 128.4 (py-*C*H), 123.2 (t, $^1J_{C,F}$ = 320 MHz, -CF_3), 47.7 (N-*C*H_3), 37.9 (*C*(CH_3)$_3$), 29.9 (C(*C*H_3)$_3$); **IR** [cm^{-1}]: ṽ = 2976, 1621, 1507, 1273, 1251, 1222, 1155, 1119, 1028, 940, 836, 754, 633, 573, 515; **HR-MS-ESI(+)** calc. $[M]^{2+}$ $C_{20}H_{30}N_2^{2+}$ 149.1199; found 149.1198.

Synthesis of **2a**

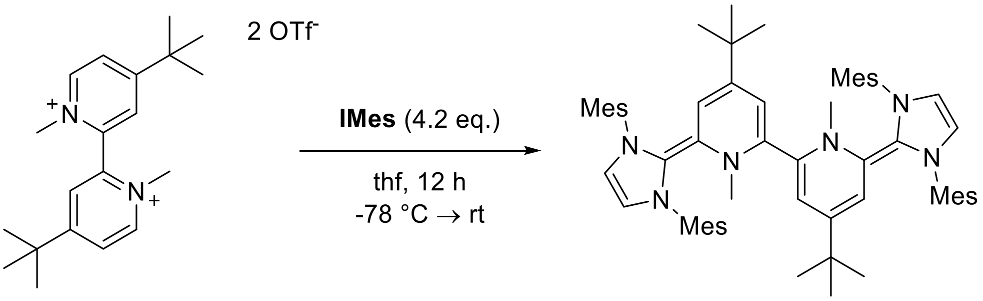

To a solution of IMes (627 mg, 2.06 mmol, 4.10 eq.) in thf (20 mL) was added dropwise a solution of **1a** (300 mg, 0.502 mmol, 1.0 eq.) at -78 °C leading to an instantaneous change in color from colorless to dark violet. The mixture was slowly warmed to room temperature

overnight. The solvent was removed under reduced pressure and the remaining solid redissolved in pentane (40 mL) under vigorous stirring, furnishing a dark purple solution. The solution was filtered over a short Celite plug (inside a glovebox) and the remaining solid extracted four times with pentane (4 x 20 mL). The solvent of the combined extracts was removed under reduced pressure and the crude product recrystallized from a pentane:Et_2O (10:1) at -40 °C, furnishing **2a** (350 mg, 386 mmol, 77%) as dark violet crystals.

m.p. 192 °C; **^{1}H NMR** (400 MHz, C_6D_6): δ [ppm] = 7.13 (s, 2H, Mes-C*H*), 6.86 (s, 2H, Mes-C*H*), 6.83 (s, 2H, Mes-C*H*), 6.73 (s, 2H, Mes-C*H*), 5.70 (s, 2H, Py-C*H*), 5.66 (d, ^3J = 2.4 Hz, 2H, ImCH), 5.58 (d, ^3J = 2.4 Hz, 2H, ImCH), 5.37 (s, 2H, Py-C*H*), 2.58 (s, 6H, Mes-CH_3), 2.38 (s, 6H, Mes-CH_3), 2.28 (s, 6H, Mes-CH_3), 2.25 (s, 12H, Mes-CH_3), 2.22 (s, 6H), 2.03 (s, 6H, N-*C*H_3), 1.02 (s, 18H, C(CH_3)$_3$); **^{13}C NMR** (101 MHz, C_6D_6): δ [ppm] = 144.3 (PyC_q-C_qPy), 138.5 (NCN), 138.2 (Mes-*C*q), 138.0 (Mes-*C*q), 137.9 (Mes-*C*q), 137.5 (Mes-*C*q), 136.7 (Mes-*C*q), 136.7 (Mes-*C*q), 136.4 (Mes-*C*q), 135.3 (Mes-*C*q), 134.2 (C_q-Py), 130.0 (Mes-*C*H), 129.5 (Mes-*C*H), 129.2 (Mes-*C*H), 128.7 (Mes-*C*H), 117.0 (Im-*C*H), 116.8 (Im-*C*H), 115.5 (Py-*C*H), 113.7(Py-*C*H), 93.6 (IMes=C_q-Py), 40.1 (N-*C*H_3), 33.2 (*C*(CH_3)$_3$), 30.4 (C(*C*H_3)$_3$), 21.2 (Mes-CH_3), 21.0 (Mes-CH_3), 19.2 (Mes-CH_3), 18.5 (Mes-CH_3), 18.3 (Mes-CH_3), 18.2 (Mes-CH_3); **IR** [cm^{-1}]: ṽ = 2912, 2851, 1619, 1583, 1481, 1437, 1394, 1376, 1361, 1328, 1286, 1242, 1197, 1155, 1083, 1027, 1009, 959, 932, 913, 882, 852, 834, 824, 815, 754, 720, 680, 655, 624, 608, 588, 576, 554, 509, 493, 415; **HR-MS-ESI(+)** calc. $[M]^{2+}$ $C_{62}H_{76}N_6^{2+}$ 452.3060; found 452.3056; **UV-vis-NIR:** λ_{max}: 553 nm (ε = 28712 cm^{-1} M^{-1}).

Synthesis of **2a^{2+}**

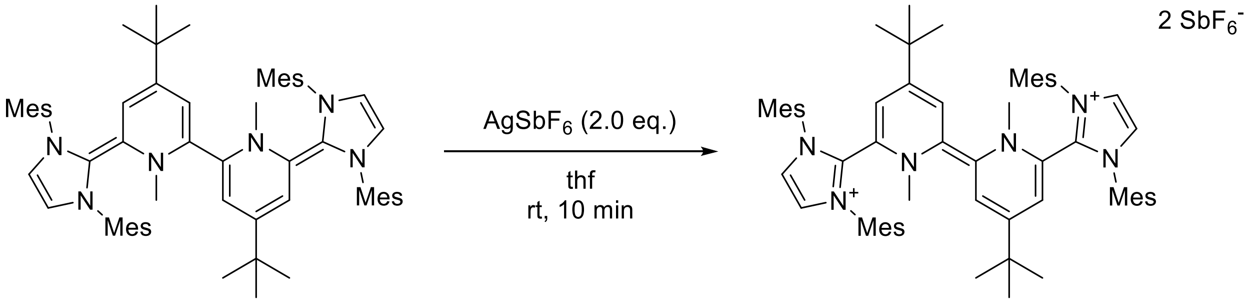

To a solution of **2a** (50 mg, 55 µmol, 1.0 eq.) in thf (5 mL) was added a solution of $AgSbF_6$ (38 mg, 110 µmol, 2.0 eq.) in THF (5 mL). After 10 min a black precipitate was collected by filtration and washed with THF (10 mL). The remaining black solid was extracted with MeCN (5 mL), furnishing a deep purple solution, from which the crude product was precipitated by addition of Et_2O (15 mL). Filtration followed by drying under reduced pressure afforded **2a^{2+}** as a deep purple solid (46 mg, 33 µmol, 61%). Note: While **2a** and **2a^{3+}** are soluble in thf, **2a^{2+}** is only soluble in MeCN and CH_2Cl_2.

^{1}H NMR (500 MHz, CD_3CN): δ [ppm] = 7.68 (s, 4H, Im-C*H*), 7.13 (s, 8H, Mes-C*H*), 5.65 (s, 2H, Py-C*H*), 4.80 (s, 2H, Py-C*H*), 2.46 (s, 6H, N-CH_3), 2.29 (s, 12H, Mes-CH_3), 2.13 (s, 24H, Mes-CH_3), 0.70 (s, 18H, C(CH_3)$_3$); **^{13}C NMR** (126 MHz, CD_3CN): δ [ppm] = 142.6 (Py-C_q), 142.4 (N*C*N), 142.2 (Mes-C_q), 134.9 (Mes-C_q), 132.1 (Mes-C_q), 131.4 (Mes-CH), 130.4 (Py-C_q), 129.9 (Py-C_q), 126.8 (Im-*C*H), 123.8 (py-CH), 119.2 (py-*C*H), 46.3 (N-CH_3), 34.1 (*C*(CH_3)$_3$), 29.2 (C(*C*H_3)$_3$), 21.1 (Mes-*C*H_3), 18.5 (Mes-CH_3); **IR** [cm^{-1}]: ṽ = 3169, 2960, 2920, 2851, 1631, 1607, 1554, 1483, 1462, 1387, 1258, 1231, 1173, 1092, 1030, 1020, 929, 856, 799, 784, 730, 653; **HR-MS-ESI(+)** calc. $[M]^{2+}$ $C_{62}H_{76}N_6^{2+}$ 452.3060; found 452.3043; **UV-vis-NIR:** λ_{max}: 574 nm (ε = 18620 cm^{-1} M^{-1}).

Synthesis of **$2a^{3+}$**

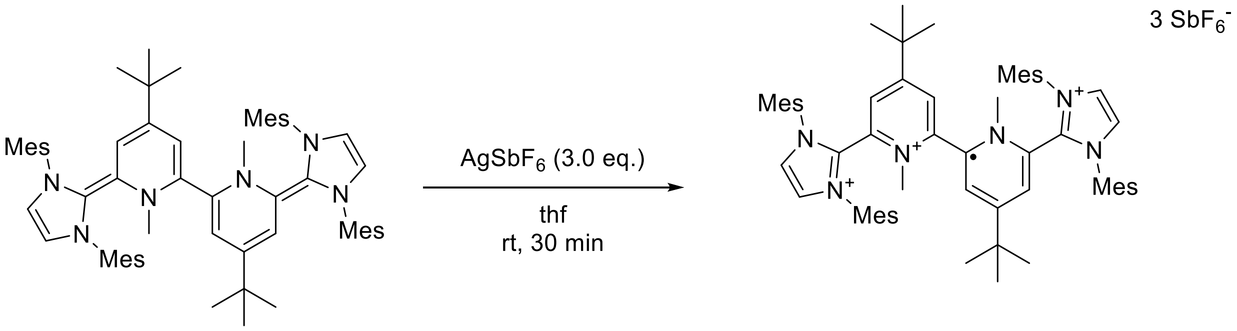

To a solution of **2a** (50 mg, 55 µmol, 1.0 eq.) in thf (5 mL) was added a solution of $AgSbF_6$ (57 mg, 165 µmol, 3.0 eq.) in thf (5 mL). After 30 minutes the black suspension was filtered over a short celite pad to obtain a green solution. The crude product was precipitated by addition of Et_2O (50 mL) and collected by filtration. The resulting precipitate was washed twice with Et_2O (2 x 10 mL). Drying under reduced pressure afforded **$2a^{3+}$** (55 mg, 34 µmol, 62 %) as an intensely green solid.

EPR: g = 2.0030 (2xN: 10.52 MHz; 4xN: 1.91 MHz; 6xH: 9.79 MHz); **IR** [cm^{-1}]: ṽ = 3173, 2965, 2922, 1606, 1551, 1484, 1349, 1301, 1263, 1228, 1158, 1111, 1028, 984, 942, 930, 861, 779, 725, 651, 574; **HR-MS-ESI(+)** calc. $[M]^{2+}$ $C_{62}H_{76}N_6^{2+}$ 452.3060; found 452.3043; **UV-vis-NIR:** λ_{max}: 602 nm (ε = 5547 cm^{-1} M^{-1}), 1233 nm (ε = 7143 cm^{-1} M^{-1}), 1513 nm (ε = 8854 cm^{-1} M^{-1}).

Synthesis of **$2a^{4+}$**

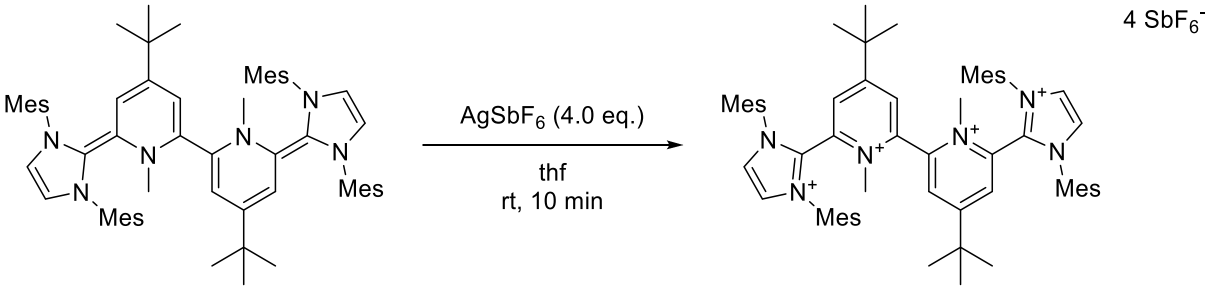

To a solution of **2a** (50 mg, 55 µmol, 1.0 eq.) in thf (5 mL) was added a solution of $AgSbF_6$ (76 mg, 220 µmol, 4.0 eq.) in thf (5 mL). After 10 min the black suspension was filtered over a short celite pad to obtain a colorless solution. The crude product was precipitated by addition of Et_2O (25 mL) and collected by filtration. Drying under reduced pressure afforded **2a^{4+}** (70 mg, 38 µmol, 69%) as an off-white solid.

^{1}H NMR (500 MHz, CD_3CN): δ [ppm] = 8.34 (d, *J* = 2.0 Hz, 2H, im-C*H*), 8.33 (d, *J* = 2.0 Hz, 2H, im-CH), 7.87 (d, *J* = 2.2 Hz, 2H, py-C*H*), 7.80 (d, *J* = 2.2 Hz, 2H, py-C*H*), 7.26 – 7.22 (m, 2H, Mes-C*H*), 7.23 – 7.18 (m, 4H, Mes-C*H*), 7.20 – 7.18 (m, 2H, Mes-C*H*), 3.31 (s, 6H, N-C*H*$_3$), 2.36 (s, 6H, Mes-CH_3), 2.34 (s, 6H, Mes-CH_3), 2.23 (s, 6H, Mes-CH_3), 2.16 (s, 6H, Mes-CH_3), 2.09 (s, 6H, Mes-CH_3), 2.03 (s, 6H, Mes-CH_3), 1.20 (s, 18H, C(C*H*$_3$)$_3$); **^{13}C NMR** (126 MHz, CD_3CN): δ [ppm] = 177.9 (py-*C*$_q$), 146.1 (py-C_q), 144.4 (Mes-*C*$_q$), 144.3 (Mes-*C*$_q$), 138.3 (py-*C*$_q$), 135.6 (Mes-*C*$_q$), 135.5 (Mes-*C*$_q$), 135.1 (Mes-*C*$_q$), 134.6 (py-*C*H), 133.7 (py-*C*H), 133.5 (NCN), 132.2 (Mes-*C*H), 132.1 (Mes-*C*H), 132.0 (Mes-*C*H), 131.2 (im-*C*H), 131.2 (im-*C*H), 129.4 (Mes-*C*$_q$), 46.3 (N-*C*H$_3$), 39.3 (*C*(CH$_3$)$_3$), 29.2 (C(*C*H$_3$)$_3$), 21.2 (Mes-*C*H$_3$), 21.1 (Mes-*C*H$_3$), 19.1 (Mes-*C*H$_3$), 19.0 (Mes-*C*H$_3$), 18.9 (Mes-*C*H$_3$); **IR** [cm^{-1}]: ṽ = 2969, 2929, 2877, 1605, 1544, 1485, 1449, 1385, 1293, 1229, 1115, 1032, 931, 896, 858, 786, 738, 653, 639, 574; **HR-MS-ESI(+)** calc. $[M+SbF_6]^{3+}$ $C_{62}H_{76}N_6SbF_6^{3+}$ 379.8352; found 379.8320.

Synthesis of **6,6'-dibromo-2,2'-bipyridine**

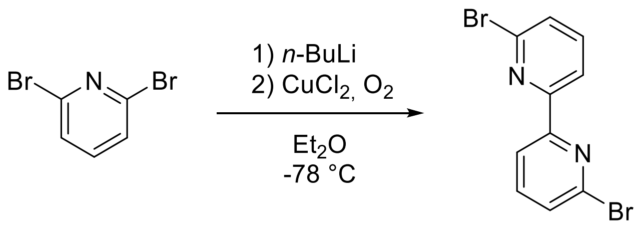

6,6'-dibromo-2,2'-bipyridine was synthesized according to a modified literature procedure.[3] 2,6-dibromopyridine (1.00 g, 4.22 mmol, 1.00 eq) was suspended in Et_2O (40 mL) and cooled to -78 °C. *n*-BuLi (2.5 M in hexane, 2 mL, 5.07 mmol, 1.20 eq.) was added dropwise. The reaction mixture was stirred for 30 min before $CuCl_2$ (284 mg, 2.11 mmol, 0.50 eq.) was added as solid in one portion. After stirring for 5 minutes oxygen was bubbled trough the solution utilizing a balloon and long cannula. The now dark brown reaction mixture was stirred additional 1.5 h at -78 °C, before 6 M HCl (100 mL) was added to quench the reaction. The phases were separated and the aqueous phase extracted twice with CH_2Cl_2 (2 x 50 mL), the combined organic phases were washed with water (2 x 50 mL) and dried over Na_2SO_4. The solvent was removed under reduced pressure and the resulting brownish crude product purified by recrystallisation from a saturated thf:pentane (1:10) solution at -24 °C over night, furnishing 6,6'-dibromo-2,2'-bipyridine (0.50 g, 1.59 mmol, 37%) as colorless crystalline needles. The spectroscopic data are in good agreement with previous reports.[4]

Note: The combination of *n*-BuLi with Et_2O and oxygen followed by addition of HCl might lead to the formation of explosive hydroperoxides. Even though no incidents occurred in our lab it is not advisable to scale this reaction up, as previous reports highlight the potential of explosions during the addition of oxygen and during the workup![5]

^{1}H NMR (400 MHz, $CDCl_3$): δ [ppm] = 8.38 (dd, *J* = 7.7, 0.9 Hz, 2H, py-C*H*), 7.67 (t, *J* = 7.8 Hz, 2H, py-C*H*), 7.50 (dd, *J* = 7.8, 0.9 Hz, 2H, Py-C*H*); **^{13}C NMR** (101 MHz, $CDCl_3$): δ [ppm] = 155.8 (py-C_q), 141.7 (py-C_q), 139.5 (py-*C*H), 128.7 (py-*C*H), 120.3 (py-*C*H).

Synthesis of **6,6'-bis(4-(tert-butyl)phenyl)-2,2'-bipyridine**

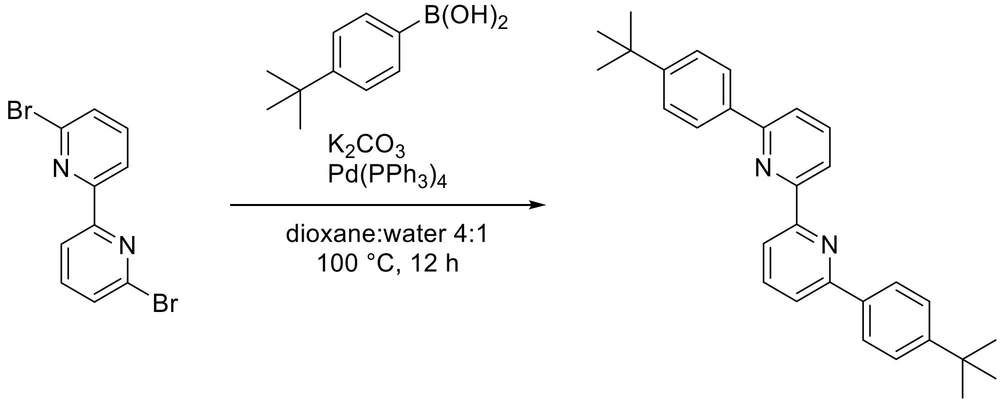

To a solution of 6,6'-dibromo-2,2'-bipyridine (1.00 g, 3.18 mmol 1.00 eq.), (4-(*tert*-butyl)phenyl)boronic acid (1.70 g, 9.55 mmol, 3.00 eq.) and K_2CO_3 (1.76 g, 12.74 mmol, 4.00 eq.) in a degassed mixture of 1,4-dioxane (40 mL) and water (10 mL) was added $Pd(PPh_3)_4$ (184 mg, 0.154 mmol, 5 mol%). The reaction mixture was stirred 12 h at 100 °C. The reaction was cooled to room temperature, water (50 mL) added and the aqueous phase extracted with CH_2Cl_2 (3 x 50 mL). The combined organic phases were dried over Na_2SO_4 and the solvent removed under reduced pressure. The crude product was purified by column chromatography (silica, pentane:ethylacetate 100:1 to 10:1 to 5:1, rf: 0.5 in 1:10 with blue fluorescence under 254 nm irradiation), furnishing the desired 6,6'-bis(4-(*tert*-butyl)phenyl)-2,2'-bipyridine (1.13 g, 2.69 mmol, 84%) as a colorless, microcrystalline solid.

m.p. 245 °C; **^{1}H NMR** (500 MHz, $CDCl_3$): δ [ppm] = δ 8.60 (dd, *J* = 7.8, 1.0 Hz, 2H, py-C*H*), 8.12 (d, *J* = 8.5 Hz, 4H, Ar-C*H*), 7.90 (t, *J* = 7.8 Hz, 2H, py-C*H*), 7.77 (dd, *J* = 7.9, 1.0 Hz, 2H, py-C*H*), 7.56 (d, *J* = 8.5 Hz, 4H, Ar-C*H*), 1.41 (s, 18H, C(C*H*$_3$)$_3$); **^{13}C NMR** (126 MHz, $CDCl_3$): δ [ppm] = 156.5 (py-C_q), 156.1 (py-C_q), 152.2 (Ar-C_q), 137.6 (py-*C*H), 136.9 (Ar-C_q), 126.8 (Ar-*C*H), 125.8 (Ar-*C*H), 120.2 (py-*C*H), 119.4 (py-*C*H), 34.8 (*C*(CH$_3$)$_3$), 31.5 (C(*C*H$_3$)$_3$); **IR** [cm^{-1}]: ṽ = 2959, 2902, 2862, 1608, 1567, 1514, 1463, 1437, 1360, 1297, 1266, 1230, 1198, 1156, 1113, 1084, 1013, 988, 844, 820, 795, 750, 739, 686, 644, 627, 598, 569, 542, 520; **HR-MS-ESI(+)** calc. $[M+H]^+$ $C_{30}H_{33}N_2^+$ 421.2638; found 421.2636.

Synthesis of **1b**

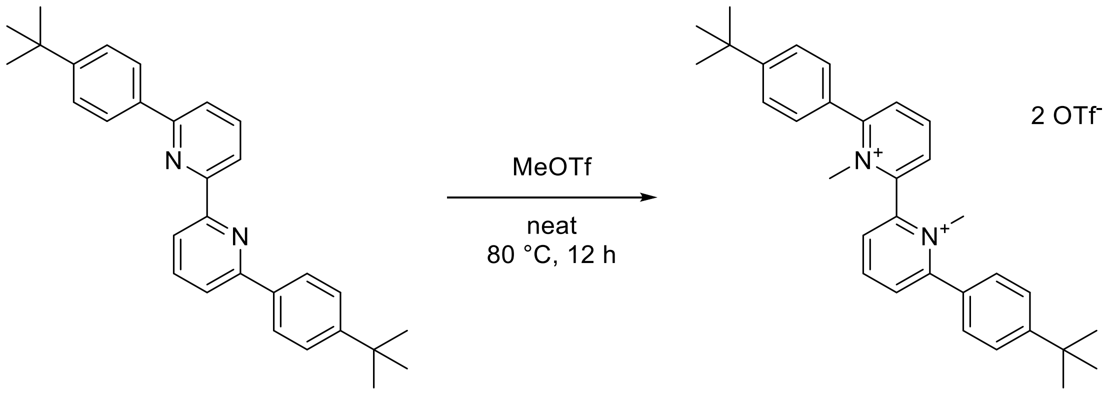

In a 100 mL teflon capped pressure schlenk flask MeOTf (2.5 mL, 22.9 mmol, 20 eq.) was added to 6,6'-bis(4-(*tert*-butyl)phenyl)-2,2'-bipyridine (1.00 g, 1.19 mmol, 1.00 eq.) at room temperature. The flask was closed and heated to 80 °C for 24 h, leading to a slightly brown solution. Under argon atmosphere dry Et_2O (50 mL) was added, leading to the formation of a voluminous colorless precipitate. The supernatant was filtered off using a filter cannula, directly quenching the filtrate with an aqueous solution of NH_3 (25%, 250 mL, vigorously stirred). The resulting colorless solid was washed twice with Et_2O (2 x 25 mL) and dried under reduced pressure, furnishing **1b** (680 mg, 0.908 mmol, 76%) as a colorless solid.

Note: MeOTf is highly cancerogenic and toxic upon inhalation or skin contact, the use of appropriate personal safety equipment is strongly advised!

m.p. 268 °C; **^{1}H NMR** (700 MHz, CD_3CN): δ [ppm] = 8.75 (t, *J* = 8.0 Hz, 2H, py-CH), 8.38 (dd, *J* = 7.9, 1.5 Hz, 2H, py-CH), 8.25 (dd, *J* = 8.1, 1.5 Hz, 2H, py-CH), 7.75 (d, *J* = 8.6 Hz, 4H, Ar-CH), 7.70 (d, *J* = 8.5 Hz, 4H, Ar-CH), 3.97 (s, 6H, N-CH_3), 1.40 (s, 18H, C(CH_3)$_3$); **^{13}C NMR** (176 MHz, CD_3CN): δ [ppm] = 160.8 (py-C_q), 156.6 (Ar-C_q), 147.2 (py-*C*H), 145.8 (py-C_q), 134.1 (py-*C*H), 130.8 (py-*C*H), 130.2 (Ar-*C*H), 129.7 (Ar-C_q), 127.4 (Ar-*C*H), 47.1 (N-*C*H_3), 35.8 (*C*(CH_3)$_3$), 31.3 (C(*C*H_3)$_3$); **IR** [cm^{-1}]: ṽ = 2965, 1611, 1575, 1476, 1366, 1253, 1223, 1162, 1112, 1029, 843, 818, 756, 680, 637, 590, 573; **HR-MS-ESI(+)** $[M]^{2+}$ $C_{32}H_{38}N_2^{2+}$ 225.1512; found 225.1515.

Synthesis of **2b**

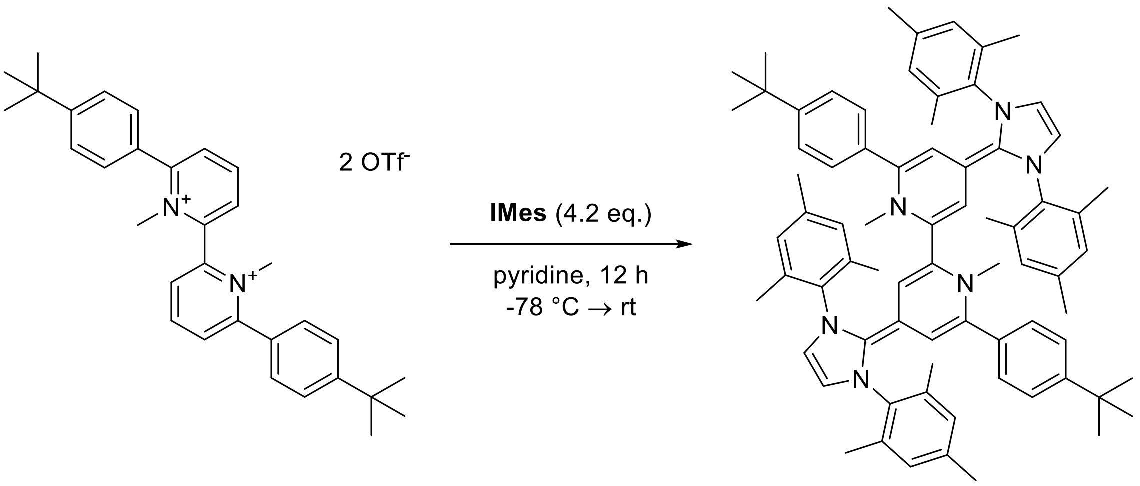

To a solution of IMes (1.33 g, 4.38µmol, 4.10 eq.) in dry and thoroughly degassed pyridine (25 mL) is added dropwise a solution of **1b** (800 mg, 1.07 mmol, 1.00 eq.) in pyridine (40 mL) at -78 °C, leading to a color change from slightly yellow to intense dark red. The reaction mixture is slowly warmed up to room temperature over 12 h. The solvent is removed under reduced pressure, the resulting dark brown solid is washed with pentane (25 mL) extracted with toluene (4 x 25 mL) and the solvent of the filtrate removed under reduced pressure. The crude product is washed with cold pentane (2 x 5 mL, -40 °C) and dried under reduced pressure, furnishing **2b** (900 mg, 851 µmol, 80 %) as a dark red solid. The product can be further purified by crystallization (saturated pentane:thf 10:1 solution, -40 °C), furnishing **2b** as dark red crystalline blocks (650 mg, 614 µmol, 57 %).

Note: To obtain sharp NMR spectra it proved necessary to add a small amount of KHMDS (~0.5 mg - 1 mg) to the freshly prepared NMR sample in C_6D_6 or d_8-THF, otherwise only broad spectra were observed.

m.p. 220 °C; **^{1}H NMR** (600 MHz, d_8-thf, 298K): δ [ppm] = 7.10 (d, *J* = 8.5 Hz, 4H, CH-Ar), 7.02 (s, 4H, Mes-CH), 6.90 (s, 4H, Mes-CH), 6.64 (d, *J* = 8.5 Hz, 4H, CH-Ar), 6.28 (d, *J* = 2.4 Hz, 2H, Im-CH), 6.22 (d, *J* = 2.4 Hz, 2H, Im-CH), 5.57 (d, *J* = 1.6 Hz, 2H, Py-CH), 5.50 (d, *J* = 1.6 Hz, 2H, Py-CH), 2.36 (s, 6H, Mes-CH_3), 2.33 (s, 12H, Mes-CH_3), 2.24 (s, 12H, Mes-CH_3), 2.02 (s, 6H, Mes-CH_3), 1.70 (s, 6H, N-CH_3), 1.32 (s, 18H, C(CH_3)$_3$); **^{13}C NMR** (151 MHz, d_8-thf, 298K): δ [ppm] = 146.9 (Ar-C_q), 138.8 (Mes-C_q), 138.8 (Mes-C_q), 137.8 (Ar-C_q), 137.8 (Mes-C_q), 137.7 (NCN), 137.6 (Mes-C_q), 136.2 (Mes-C_q), 135.9 (Py-C_q), 135.6 (Py-C_q), 130.5 (Mes-CH), 130.2 (Mes-CH), 124.9 (Ar-CH), 124.8 (Ar-CH), 118.4 (Im-CH), 118.2 (Im-CH), 113.9 (Py-CH), 110.1 (Py-CH), 85.9 (Py-C_q), 42.6 (N-*C*H_3), 34.9 (*C*(CH_3)$_3$), 32.1 (C(*C*H_3)$_3$), 21.3 (Mes-CH_3), 21.2 (Mes-CH_3), 18.9 (Mes-CH_3), 18.6 (Mes-CH_3); **IR** [cm^{-1}]: ṽ = 2911, 2860, 1624, 1599, 1558, 1544, 1529, 1505, 1480, 1400, 1376, 1326, 1307, 1269, 1252, 1201, 1178, 1159, 1099, 1082, 1030, 1018, 1004, 961, 914, 850, 826, 770, 752, 733, 696, 664, 637, 615, 589, 574, 540,

503, 467, 424; **HR-MS-ESI(+)** calc. $[M]^{2+}$ $C_{74}H_{84}N_6^{2+}$ 528.3373; found 528.3377; **UV-vis-NIR:** λ_{max}: 259 nm (ε = 26231 cm^{-1} M^{-1}), 435 nm (ε = 27669 cm^{-1} M^{-1}), 486 nm (ε = 31897 cm^{-1} M^{-1}).

Synthesis of $\mathbf{2b^{2+}}$

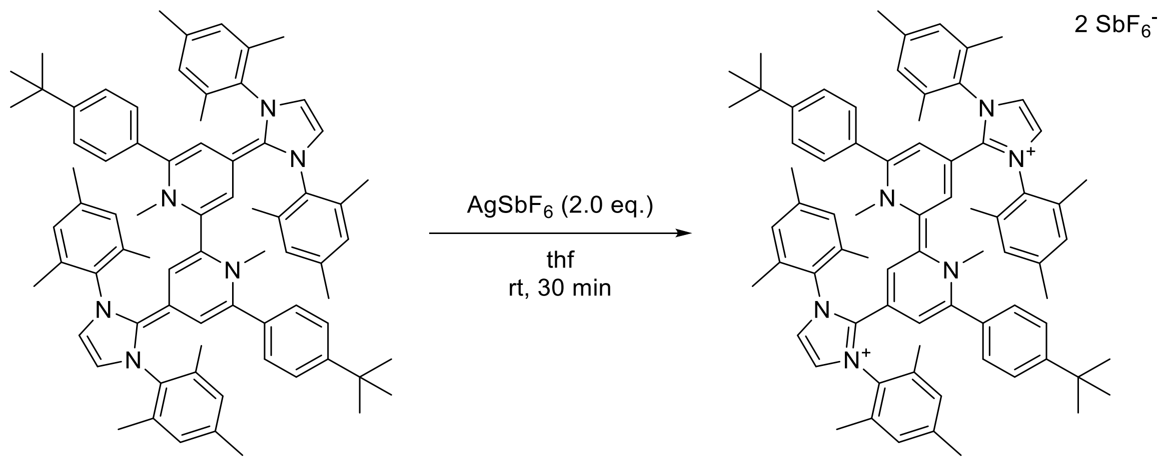

To a solution of **2b** (50 mg, 47.3 µmol, 1.00 eq.) in thf (5 mL) was added a solution of $AgSbF_6$ (32.5 mg, 94.6 µmol, 2.00 eq.) in thf (2 mL, rinsed with 2 mL to ensure complete addition), resulting in a change of color from dark red to dark blue. After stirring the reaction mixture for 30 minutes, the precipitated silver is filtered off using a short celite pad. The celite pad was rinsed with additional thf (2 mL). The combined solvent filtrate was removed under reduced pressure and the resulting brown solid was washed first with Et_2O (10 mL) and twice with thf (3x 10 mL). (Note: While the crude product, which is likely contaminated with minor amounts of the respective mono- or trication, is soluble in thf the solubility decreases significantly during the purification process). The blue crude product was recrystallized from CH_2Cl_2:Et_2O 1:5 at -40 °C, furnishing $\mathbf{2b^{2+}}$ (45 mg, 29.4 µmol, 63 %) as a dark blue solid.

^{1}H NMR (600 MHz, CD_2Cl_2): δ [ppm] = 7.49 (s, 4H, Im-C*H*), 7.39 (d, *J* = 8.4 Hz, 4H, Ar-C*H*), 7.14 (s, 8H, Mes-C*H*), 6.59 (d, *J* = 8.3 Hz, 4H, Ar-C*H*), 6.02 (d, *J* = 1.8 Hz, 2H, py-C*H*), 5.09 (s, 2H, py-C*H*), 2.29 (s, 12H, Mes-CH_3), 2.16 (s, 24H, Mes-CH_3), 1.87 (bs, 6H, N-CH_3) 1.38 (s, 18H, C(CH_3)$_3$); **^{13}C NMR** (151 MHz, CD_2Cl_2): δ [ppm] = 154.1 (C_q), 152.6 (C_q), 142.6 (C_q), 134.8 (C_q), 133.5 (C_q), 132.6 (C_q), 131.6 (C_q), 130.8 (Mes-CH), 127.3 (Ar-*C*H), 126.4 (py-*C*H), 126.1 (Ar-*C*H), 125.2 (Im-*C*H), 106.2 (py-*C*H), 46.1 (N-CH_3), 35.2 (*C*(CH_3)$_3$), 31.4 (C(*C*H_3)$_3$), 21.2 (Mes-CH_3), 18.0 (Mes-CH_3); two quaternary C-atoms not visible due to broadening; **IR** [cm^{-1}]: $\tilde{v}$ = 3178, 3148, 2966, 2865, 1595, 1562, 1459, 1365, 1256, 1229, 1166, 1110, 1058, 1031, 861, 844, 807, 762, 724, 701, 652, 607, 571; **HR-MS-ESI(+)** calc. $[M]^{2+}$ $C_{74}H_{84}N_6^{2+}$ 528.3373; found 528.3367; **UV-vis-NIR:** λ_{max}: 656 nm (ε = 15348 cm^{-1} M^{-1}).

Synthesis of **2b^{3+}**

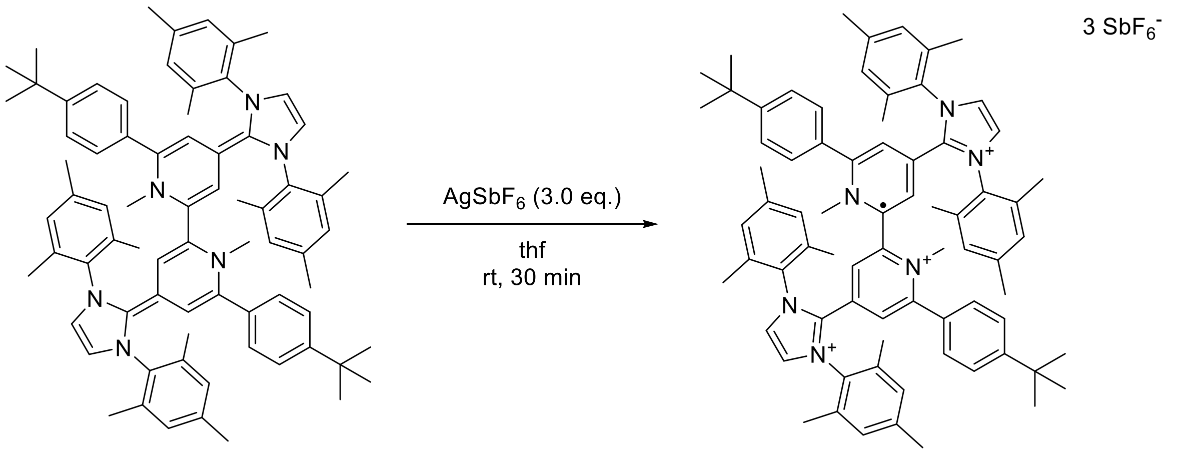

To a solution of **2b** (50 mg, 47.3 µmol, 1.00 eq.) in thf (5 mL) was added a solution of $AgSbF_6$ (49 mg, 141 µmol, 3.00 eq.) in thf (2 mL, rinsed with 2 mL to ensure complete addition), resulting in a change of color from dark red to orange. After stirring the reaction mixture for 30 minutes, the precipitated silver is filtered off using a short celite pad. The celite pad was rinsed with additional thf (2 mL). The solvent of the combined filtrate was removed under reduced pressure and the crude product recrystallized from CH_2Cl_2:Et_2O 1:5 at -40 °C, furnishing **2b^{3+}** (51 mg, 28.9 µmol, 61 %) as an olive green solid.

EPR: g = 2.0031 (hfc could not be resolved); **IR** [cm^{-1}]: $\tilde{\nu}$ = 3169, 2964, 2869, 1607, 1556, 1483, 1432, 1322, 1254, 1227, 1193, 1149, 1111, 1061, 1013, 901, 842, 732, 654; **HR-MS-ESI(+)** calc. $[M]^{3+}$ $C_{74}H_{84}N_6^{3+}$ 352.2247; found 352.2226; **UV-vis-NIR:** λ_{max}: 287 nm (ε = 24152 cm^{-1} M^{-1}), 427 nm (ε = 8645 cm^{-1} M^{-1}), 559 nm (ε = 2573 cm^{-1} M^{-1}), 1401 nm (ε = 4741 cm^{-1} M^{-1}), 1698 nm (ε = 5339 cm^{-1} M^{-1}).

Synthesis of **2b^{4+}**

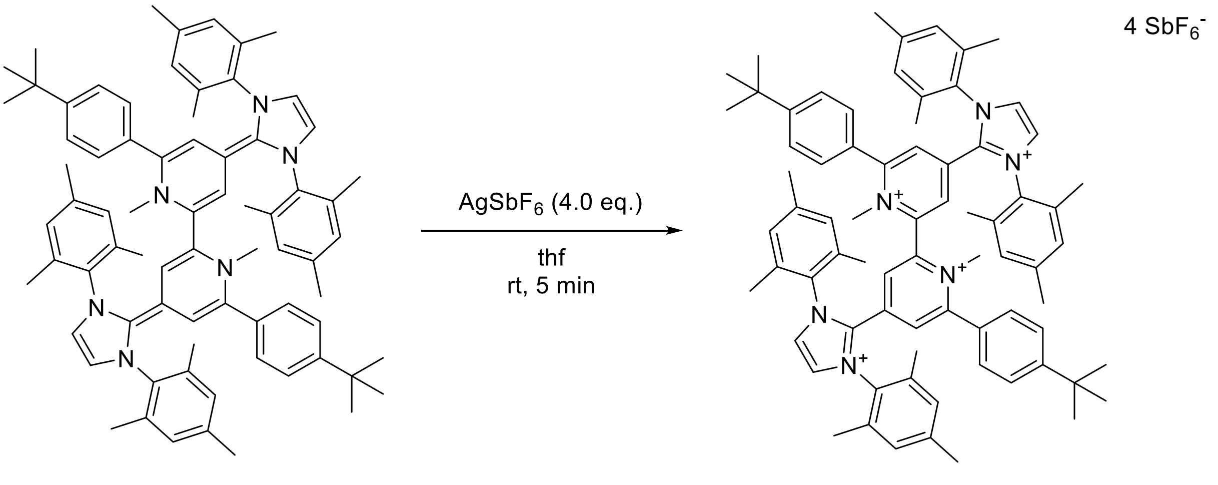

To a solution of **2b** (50 mg, 47.3 µmol, 1.00 eq.) in thf (5 mL) was added a solution of $AgSbF_6$ (65 mg, 189 µmol, 4.00 eq.) in thf (2 mL, rinsed with 2 mL to ensure complete addition),

resulting in a change of color from dark red to light yellow / grey. After stirring the reaction mixture for 30 minutes, the precipitated silver is filtered off using a short celite pad. The celite pad was rinsed with additional thf (3 x 2 mL). The combined solvent filtrate was removed under reduced pressure and the crude product recrystallized from CH_2Cl_2:Et_2O 1:5 at -40 °C, furnishing **2b**$^{4+}$ (64 mg, 31.9 µmol, 68 %) as yellow solid.

Note: The NMR-spectra of **2b**$^{4+}$point to the coordination of 1 equivalent of Et_2O, which can not be removed by drying the solid for 6 h at 10^{-3} mbar and 40 °C.

^{1}H NMR (500 MHz, CD_3CN): δ [ppm] = 8.17 (s, 4H, Im-C*H*), 7.78 (d, *J* = 8.6 Hz, 4H, Ar-CH), 7.60 (d, *J* = 2.3 Hz, 2H, py-C*H*), 7.36 (d, *J* = 2.2 Hz, 2H, py-C*H*), 7.27 (s, 4H; Mes-C*H*), 7.17 (d, *J* = 8.6 Hz, 4H, Ar-C*H*), 7.14 (s, 4H, Mes-C*H*), 3.19 (s, 6H, N-CH_3), 2.33 (s, 12H, Mes-C*H*$_3$), 2.19 (s, 12H, Mes-C*H*$_3$), 1.97 (s, 12H, Mes-C*H*$_3$), 1.42 (s, 18H, C(C*H*$_3$)$_3$); **^{13}C NMR** (151 MHz, CD_3CN): δ [ppm] = 163.5 (py-C_q), 158.7 (Ar-C_q), 145.4 (py-C_q), 144.2 (Mes-C_q), 136.7 (py-C_q), 135.9 (Mes-C_q), 135.6 (Mes-C_q), 134.3 (py-*C*H), 131.6 (Mes-*C*H), 131.5 (Mes-*C*H), 130.3 (Ar-*C*H), 130.0, 129.7 (Im-*C*H), 128.0 (Ar-CH), 127.7 (Ar-C_q), 127.6 (py-*C*H), 48.3 (N-*C*H$_3$), 36.1 (*C*(CH$_3$)$_3$), 31.1 (C(*C*H$_3$)$_3$), 21.2, 18.6 (Mes-*C*H$_3$), 18.1 (Mes-*C*H$_3$); **IR** [cm^{-1}]: ṽ = 2971, 1604, 1554, 1487, 1254, 1229, 1111, 1014, 900, 842, 733, 657, 563; **HR-MS-ESI(+)** calc. $[M+SbF_6]^{3+}$ $C_{74}H_{84}N_6SbF_6^{3+}$ 430.5228; found 430.5203; calc. $[M+(SbF_6)_2]^{2+}$ $C_{74}H_{84}N_6Sb_2F_{12}^{2+}$ 763.2316; found 763.2294;**UV-vis-NIR:** λ_{max}: 297 nm (ε = 24218 cm^{-1} M^{-1}).

Synthesis of **bis(4-(tert-butyl)phenyl)-1,10-phenanthroline**

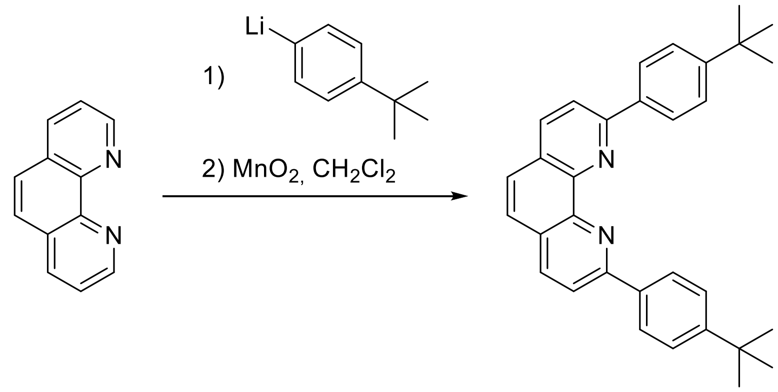

1-bromo-4-*tert*-butylbenzene (1.54 mL, 8.88 mmol, 4.0 equiv.) was added to elemental lithium (154 mg, 22.2 mmol, 10.0 equiv) in Et_2O (10 mL) at 0 °C and stirred for another 3 h at ambient temperature. The reaction mixture was added to a solution of phenanthroline (400 mg, 2.22 mmol, 1.0 equiv.) in Et_2O (10 mL) at -78 °C. Afterwards, the reaction mixture was allowed to warm up to ambient temperature and stirred over night. The solution was poured onto ice and the aqueous phase extracted with EtOAc (1x) and CH_2Cl_2 (2x). The combined organic phases were dried over Na_2SO_4 and all volatiles removed under reduced pressure. CH_2Cl_2 (50 mL) and MnO_2 (3.9 g, 44.8 mmol, 20 equiv.) were added and the suspension stirred over

night at ambient temperature. The reaction mixture was filtrated over a short silica plug and all volatiles were removed under reduced pressure. After purification by column chromatography (*n*-hexane/EtOAc/NEt_3 5:1:1% -> *n*-hexane/EtOAc/NEt_3 1:1:1%) the desired 2,9-bis(4-(*tert*-butyl)phenyl)-1,10-phenanthroline was obtained as crystalline colorless solid (660 mg, 1.48 mmol, 67%).

m.p. 248 °C; **^{1}H NMR** ($CDCl_3$, 400 MHz, 298 K): δ [ppm] = 8.46 (d, *J* = 8.4 Hz, 2H), 8.36 (d, *J* = 8.5 Hz, 4H), 8.22 (d, *J* = 8.5 Hz, 2H), 7.87 (s, 2H), 7.64 (d, *J* = 8.4 Hz, 4H), 1.42 (s, 18H).; **^{13}C NMR** ($CDCl_3$ 100 MHz, 289 K): 156.7 (Ar-C_q), 153.5 (Ar-C_q), 144.4 (Ar-C_q), 138.2 (Ar-*C*H), 135.7 (Ar-C_q), 128.1 (Ar-C_q), 127.72 (Ar-*C*H), 126.23 (Ar-*C*H), 126.2 (Ar-*C*H), 120.9 (Ar-*C*H), 35.0 (*C*(CH_3)$_3$), 31.5 (C(*C*H_3)$_3$); **IR** [cm^{-1}]: ṽ = 2901, 2863, 1604, 1587, 1562, 1543, 1486, 1420, 1399, 1365, 1313, 1268, 1192, 1154, 1120, 1092, 1012, 975, 891, 855, 837, 794, 747, 732, 643, 627, 601, 543, 420; **HR-MS-ESI(+)** calc. $[M+H]^+$ $C_{32}H_{33}N_2^{2+}$ 445.2638; found 445.2641.

Synthesis of **1c**

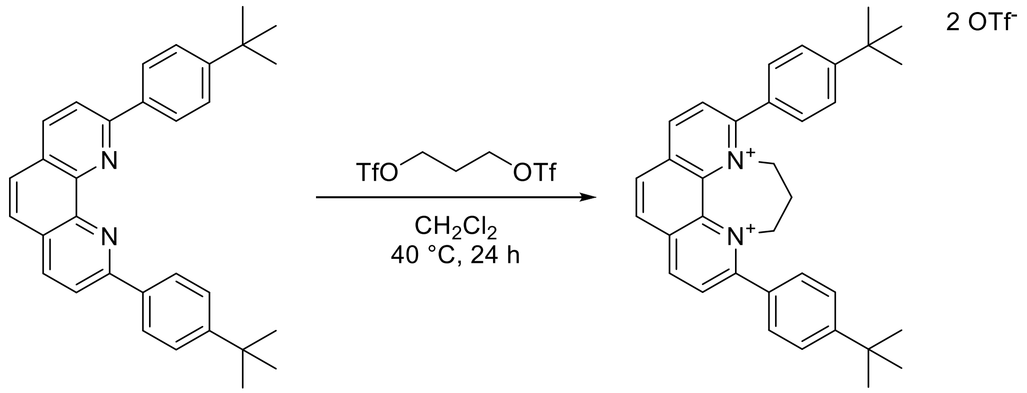

To a solution of 2,9-bis(4-(*tert*-butyl)phenyl)-1,10-phenanthroline (500 mg, 1.12 mmol, 1.00 eq.) in CH_2Cl_2 (25 mL) was added prop(OTf)$_2$ (497 mg, 1.46 mmol, 1.3 eq.). The reaction mixture was heated to 40 °C in a closed Schlenk flask and stirred over night. Under inert atmosphere Et_2O (~150 mL) was added (Note: the product is not particularly air sensitive however its hygroscopicity prevents the filtration under air, as the resulting yellow sticky substance complicates further purification) and the resulting suspension was vigorously stirred for 30 minutes before the supernatant was filtered off and the resulting yellow solid washed twice with Et_2O (2 x 40 mL). Drying under reduced pressure furnishes **1c** (650 mg,0.828 mmol, 74 %) as an off-white solid.

m.p. 207.1 °C; **^{1}H NMR** (500 MHz, CD_3CN): δ [ppm] = 9.38 (d, *J* = 8.6 Hz, 2H, phen-CH), 8.66 (s, 2H, phen-CH), 8.42 (d, *J* = 8.5 Hz, 2H, phen-CH), 7.83 - 7.79 (m, 8H, Ar-CH), 4.83 (bs, 4H, -C*H*$_2$-CH$_2$-C*H*$_2$), 3.13 (pentet, *J* = 6.7 Hz, 2H, -CH$_2$-C*H*$_2$-CH$_2$), 1.43 (s, 18H, C(C*H*$_3$)$_3$); **^{13}C NMR** (126 MHz, CD_3CN): δ [ppm] = 164.5 (phen-C_q), 157.6 (Ar-C_q), 147.4 (phen-*C*H), 136.2 (phen-C_q), 134.7 (phen-C_q), 131.4 (phen-*C*H), 131.2 (phen-*C*H), 130.3 (Ar-C_q), 130.1 (Ar-*C*H), 127.7

(Ar-*C*H), 59.5 (-*C*H_2-CH_2-*C*H_2), 35.8 (*C*(CH_3)$_3$), 35.7 (-CH_2-*C*H_2-CH_2), 31.2 (C(*C*H_3)$_3$); **IR** [cm^{-1}]: ṽ = 2959, 2901, 2863, 1604, 1587, 1562, 1543, 1486, 1420, 1399, 1365, 1313, 1268, 1192, 1154, 1120, 1092, 1012, 975, 891, 855, 837, 794, 747, 732, 643, 627, 601, 543; **HR-MS-ESI(+)** calc. $[M]^{2+}$ $C_{35}H_{38}N_2^{2+}$ 243.1512; found 243.1510.

Synthesis of **1c^{Int}**

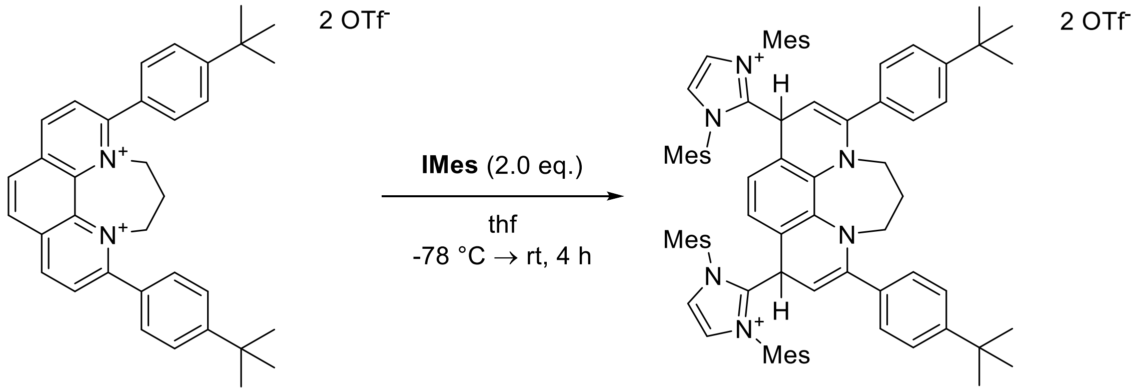

To a solution of **1c** (300 mg, 382 µmol, 1.00 eq.) in thf (20 mL) was added dropwise a solution of IMes (232 mg, 764 µmol, 2.00 eq.) in thf (20 mL) at -78 °C over 30 minutes, leading to a change in color from light yellow to light orange. The solution was allowed to warm up to room temperature and stirred additional 30 minutes, before the solvent was removed under reduced pressure. The resulting reddish solid was washed twice with Et_2O (2 x 20 mL) and pentane (2x 20 mL). Drying under reduced pressure furnished **1c^{Int}** (316 mg, 226 µmol, 59%) as a slightly violet solid.

m.p. >250 °C; **^{1}H NMR** (600 MHz, CD_2Cl_2): δ [ppm] = 7.55 (s, 4H, Im-C*H*), 7.38 (d, *J* = 8.2 Hz, 4H, Ar-C*H*), 7.16 (s, 4H, Mes-C*H*), 7.00 (d, *J* = 8.3 Hz, 4H, Ar-C*H*), 6.92 (s, 4H, Mes-C*H*), 6.39 (s, 2H, Phen-C*H*), 5.18 (d, *J* = 6.0 Hz, 2H, Phen(Im)-C*H*), 4.39 (d, *J* = 6.0 Hz, 2H, Phen-C*H*), 2.83 (dt, *J* = 14.7, 8.6 Hz, 2H, prop-C*H*$_2$), 2.61 (dt, *J* = 14.7, 4.1 Hz, 2H, prop-C*H*$_2$), 2.33 (s, 12H, Mes-C*H*$_3$), 2.29 (s, 12H, Mes-C*H*$_3$), 1.87 (s, 12H, Mes-C*H*$_3$), 1.30 (s, 18H, C(C*H*$_3$)$_3$), 1.11 – 1.05 (m, 2H, prop-C*H*$_2$); **^{13}C NMR** (151 MHz, CD_2Cl_2): δ [ppm] = 153.4 (Ar-C_q), 149.0 (Phen-C_q), 147.5 (NCN), 142.3 (Mes-C_q), 135.4 (Ar-C_q) 135.2 (Mes-C_q), 134.8 (Mes-C_q), 133.1 (Ar-C_q), 130.6 (Mes-C_q), 130.6 (Mes-*C*H), 130.3 (Mes-CH), 127.9 (Ar-CH), 126.0 (Ar-*C*H), 125.5 (Im-*C*H), 124.4 (Phen-*C*H), 119.8 (Phen-C_q), 95.2 (Phen-*C*H), 49.4 (2 x prop-*C*H_2), 37.7 (Phen(Im)-*C*H), 35.0 (*C*(CH_3)$_3$), 31.3 (C(*C*H_3)$_3$), 28.1 (Prop-*C*H_2), 21.3 (Mes-*C*H_3), 18.6 (Mes-*C*H_3), 18.1 (Mes-*C*H_3); **IR** [cm^{-1}]: ṽ = 2964, 1631, 1601, 1550, 1523, 1500, 1465, 1407, 1367, 1330, 1259, 1221, 1151, 1112, 1086, 1028, 950, 882, 837, 809, 779, 754, 633, 608, 587, 572, 542, 516; **HR-MS-ESI(+)** calc. $[M]^{2+}$ $C_{77}H_{86}N_6^{2+}$ 547.3451; found 547.3452.

Synthesis of **2c**

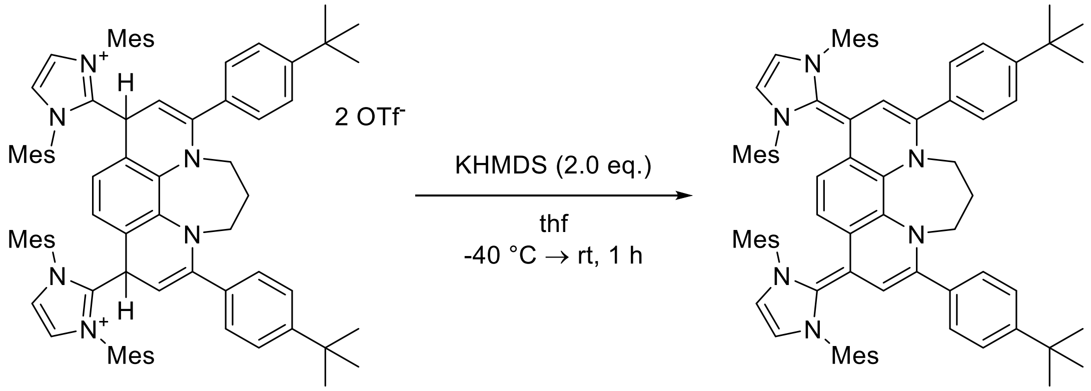

To a solution of **$1c^{Int}$** (200 mg, 143 µmol, 1.00) in thf (10 mL) was added a solution of KHMDS (57 mg, 287 µmol, 2.00 eq.) in thf (10 mL) at -40 °C. The reaction mixture was warmed to rt and stirred additional 30 minutes, leading to a color change to dark red. The solvent was removed under reduced pressure and the resulting solid extracted four times with Et_2O (4x 20 mL). The combined extracts were filtered over a short celite pad and the solvent removed under reduced pressure. The crude product was washed with pentane (15 mL) and dried under reduced pressure, yielding **2c** (125 mg, 114 µmol, 79%) as a dark red solid.

Note: To obtain NMR spectra it proved necessary to add a small amount of KHMDS (~0.5 mg -1 mg) to the freshly prepared NMR sample in C_6D_6 or d_8-thf, otherwise only broad spectra were observed (see Fig. S54).

m.p. 240 °C; **^{1}H NMR** (500 MHz, d_8-THF, 298 K): δ [ppm] = 7.10 (d, *J* = 8.7 Hz, 4H, Ar-CH), 7.03 (d, *J* = 8.5 Hz, 4H, Ar-CH), 7.00 (bs, 4H, Mes-CH), 6.78 (bs, 4H, Mes-CH), 6.42 (s, 4H, Im-CH), 5.47 (s, 2, Phen-CH), 5.45 (s, 2H, Phen-CH), 2.73 – 2.54 (m, 4H, 2x prop-CH_2), 2.26 (s, 24H, Mes-CH_3), 2.21 (bs, 12H, Mes-CH_3), 1.54 – 1.43 (m, 2H, prop-CH_2), 1.26 (s, 18H, C(CH_3)$_3$); **^{13}C NMR** (125 MHz, d_8-THF): δ [ppm] = 147.3 (Ar-C_q), 142.9 (NCN), 139.4 (Phen-C_q), 137.6 (Mes-C_q), 137.5 (broad, Mes-C_q), 137.2 (Ar-C_q), 137.0 (Phen-C_q), 135.4 (Mes-C_q), 130.4 (Mes-CH), 128.6 (Phen-C_q), 125.4 (Ar-CH), 125.3 (Ar-CH), 119.0 (Im-CH), 116.4 (Phen-*C*H), 115.8 (Phen-*C*H), 83.9 (Phen-C_q-Im), 55.5 (2x prop-CH_2), 35.0 (*C*(CH_3)$_3$), 32.0 (C(*C*H_3)$_3$), 30.8 (prop-CH_2), 21.2 (Mes-CH_3), 19.4 (Mes-CH_3), 18.9 (Mes-CH_3); **IR** [cm^{-1}]: ṽ = 2948, 1582, 1539, 1508, 1481, 1450, 1397, 1322, 1303, 1262, 1194, 1160, 1110, 1078, 1049, 1030, 1012, 965, 914, 852, 840, 818, 733, 697, 669, 659, 610, 599, 576, 546, 521; **HR-MS-ESI(+)** calc. $[M+H]^+$ $C_{77}H_{85}N_6^{2+}$ 1093.6830 found 1093.6921 (-8.3 ppm derivation) and calc. $[M+H]^+$ $C_{77}H_{86}N_6^+$ 547.3451; found 547.3441; **UV-vis-NIR:** λ_{max}: 412 nm (ε = 17159 cm^{-1} M^{-1}), 508 nm (ε = 19742 cm^{-1} M^{-1}).

Synthesis of **2c⁺**

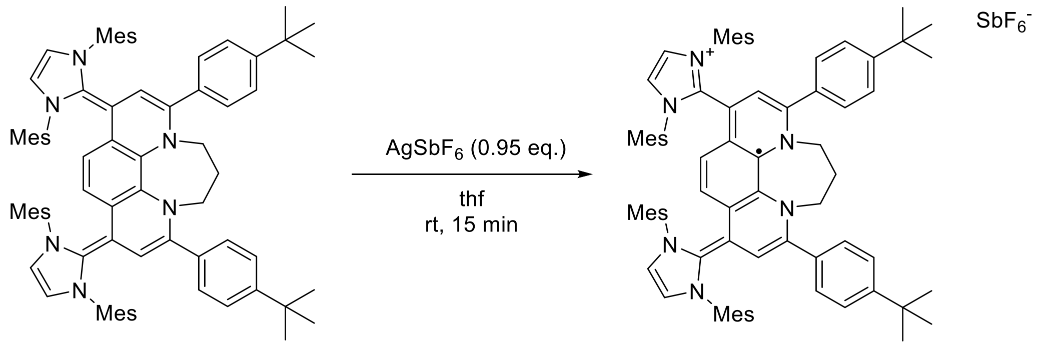

To a solution of **2c** (50 mg, 45.7 µmol, 1.00 eq.), in thf (5 mL) was added a solution of $AgSbF_6$ (14.9 mg, 43.4µmol, 0.95 eq.) in thf (2 mL, rinsed with 2 mL to ensure complete addition), resulting in a change of color from dark red to bright green. After stirring the reaction mixture for 15 minutes, the precipitated silver was filtered off using a short celite pad. The celite pad was rinsed with additional thf (2 mL). The solvent of the filtrate was removed under reduced pressure and the crude product recrystallized from thf:Et_2O 1:10 at -40 °C, furnishing **2c⁺** (36 mg, 27.1 µmol, 59%) as a green microcrystalline solid.

EPR: g = 2.0032 (hfcs could not be resolved); **IR** [cm^{-1}]: ṽ = 2861, 1560, 1465, 1436, 1415, 1323, 1285, 1253, 1195, 1097, 1083, 1051, 1031, 990, 925, 856, 841, 831, 819, 795, 777, 748, 731, 655, 597, 574, 550, 520, 469, 421; **HR-MS-ESI(+)** only $[M]^{2+}$ was observed: calc. $[M]^{2+}$ $C_{77}H_{84}N_6^{2+}$ 546.3373; found 546.3369. **UV-vis-NIR:** λ_{max}: 441 nm (ε = 17612 cm^{-1} M^{-1}), 711 nm (ε = 7869 cm^{-1} M^{-1}), 1285 nm (ε = 3976 cm^{-1} M^{-1}), 1520 nm (ε = 4385 cm^{-1} M^{-1}).

Synthesis of **2c²⁺**

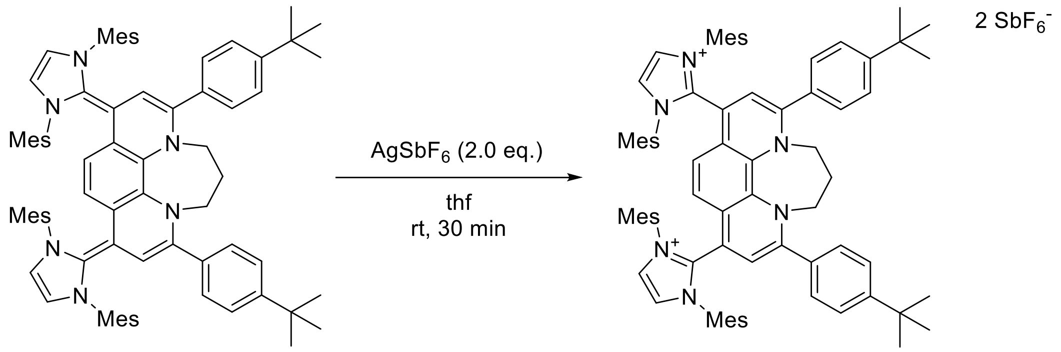

A solution of **2c** (50 mg, 45.7 µmol, 1.00 eq.), in thf (5 mL) was titrated with a solution of $AgSbF_6$ (31 mg, 91.4 µmol, 2.00 eq.) in thf (5 mL). The addition was stopped as soon as the color changed from dark red to green and back to an intense orange/red (~ 4.8 mL). After stirring the reaction mixture for 30 minutes, the precipitated silver was filtered off using a short celite pad. The celite pad was rinsed with additional thf (2 mL). The solvent of the filtrate was removed under reduced pressure. The resulting solid was redissolved and filtrated twice over

a short celite pads to ensure complete removal of precipitated silver. The crude product was twice recrystallized from THF:Et_2O 1:5 at -40 °C (crystals were collected by filtration, rinsed with Et_2O (5 ml), redissolved in a minimal amount of the indicated THF:Et_2O mixture, filtrated and stored overnight at -40 °C), furnishing **2c**$^{2+}$ (18 mg, 12.9 µmol, 29%) as an orange microcrystalline solid.

^{1}H NMR (400 MHz, d_8-THF, 224 K): δ [ppm] = 8.07 (s, 4H, Im-CH), 7.36 – 7.25 (m, 12H, Ar-CH & Mes-CH), 6.65 (d, J = 8.1 Hz, 4H, Ar-CH), 5.28 (s, 2H, phen-CH), 4.03 (s, 2H, phen-CH), 3.09 – 2.95 (m, 4H, 2 x prop-CH_2), 2.51 (s, 12H, Mes-CH_3), 2.20 (s, 12H, Mes-CH_3), 2.17 (s, 12H, Mes-CH_3), 1.22 (s, 18H, C(C*H*$_3$)$_3$), 0.72 – 0.58 (m, 2H, prop-CH_2); **^{13}C NMR** (101 MHz, d_8-THF, 224 K): δ [ppm] = 153.3 (Ar-C_q), 153.2 (Ar-C_q), 144.3 (NCN), 141.9 (Mes-C_q), 138.7 (phen-C_q), 135.5 (Mes-C_q), 135.0 (Mes-C_q), 133.3 (phen-C_q), 132.0 (Mes-C_q), 131.5 (Mes-CH), 131.0 (Mes-CH), 129.2 (phen-C_q), 128.4 (Ar-CH), 126.5 (Ar-CH), 126.5 (Im-CH), 124.1 (phen-CH), 110.7 (phen-C_q), 110.2 (phen-CH), 50.0 (2 x prop-CH_2), 35.5 (*C*(CH_3)$_3$), 31.5 (C(*C*H$_3$)$_3$), 21.5 (Mes-CH_3), 18.7 (Mes-CH_3), 18.5 (Mes-CH_3), 17.0 (prop-CH_2); **IR** [cm^{-1}]: ṽ = 2960, 2864, 1608, 1555, 1478, 1453, 1381, 1329, 1261, 1230, 1198, 1172, 1141, 1108, 1083, 1028, 928, 853, 803, 753, 726, 652, 592, 573, 552, 523, 422; **HR-MS-ESI(+)** calc. $[M]^{2+}$ $C_{77}H_{84}N_6^{2+}$ 546.3373; found 546.3369; **UV-vis:** λ_{max}: 354 nm (ε = 26869 cm^{-1} M^{-1}), 452 nm (ε = 7182 cm^{-1} M^{-1}), 1041 nm (ε = 5232 cm^{-1} M^{-1}).

Synthesis of **2c**$^{3+}$

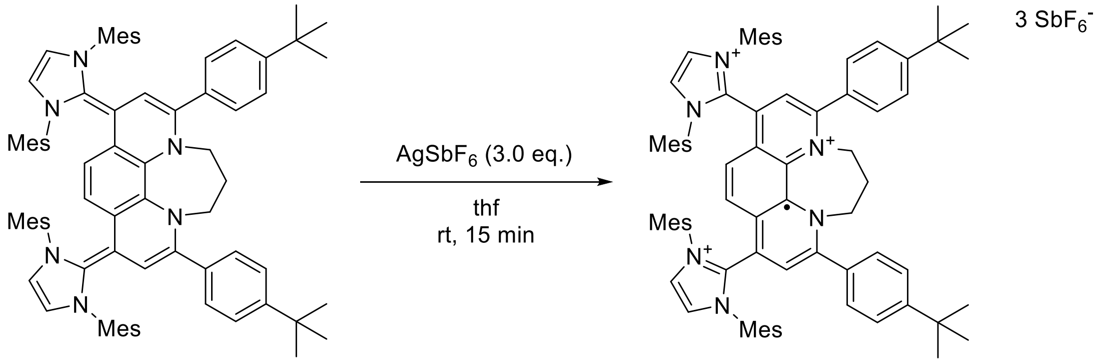

To a solution of **2c** (45 mg, 41.2 µmol, 1.00 eq.), in thf (5 mL) was added a solution of $AgSbF_6$ (42.4 mg, 123.5 µmol, 3.00 eq.) in thf (2 mL, rinsed with 2 mL to ensure complete addition), resulting in a change of color from dark red to orange. After stirring the reaction mixture for 15 minutes, the precipitated silver was filtered off using a short celite pad. The celite pad was rinsed with additional thf (2 mL). The solvent of the filtrate was removed under reduced pressure and the crude product recrystallized from CH_2Cl_2:Et_2O 1:10 at -40 °C, furnishing **2c**$^{3+}$ (28 mg, 15.6 µmol, 38%) as a dark green solid.

EPR: g = 2.0033 (hfcs could not be resolved); **IR** [cm^{-1}]: ṽ = 2963.99, 2869.79, 1604.70, 1533.30, 1483.49, 1461.07, 1367.42, 1301.98, 1249.35, 1230.47, 1204.47, 1136.95, 1110.13,

1082.96, 1044.98, 1004.51, 929.79, 899.55, 839.24, 803.09, 756.62, 731.70, 651.20, 589.95, 573.49, 552.85, 524.84, 423.12; **HR-MS-ESI(+)** calc. $[M]^{3+}$ $C_{77}H_{84}N_6^{3+}$ 364.2247; found 364.2232; **UV-vis:** λ_{max}: 353 nm (ε = 33953 cm^{-1} M^{-1}), 585 nm (ε = 3269 cm^{-1} M^{-1}), 1573 nm (ε = 2230 cm^{-1} M^{-1}), 2071 nm (ε = 2590 cm^{-1} M^{-1}).

Synthesis of **$2c^{4+}$**

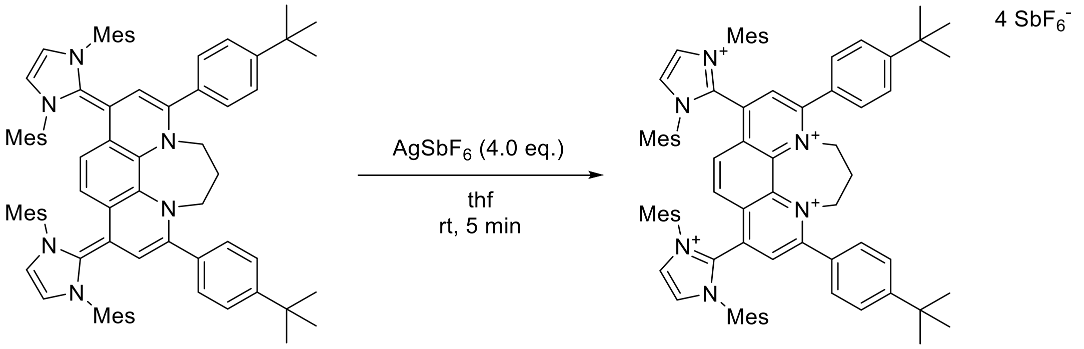

To a solution of **2c** (50 mg, 45.7 µmol, 1.00 eq.), in thf (5 mL) was added a solution of $AgSbF_6$ (62.8 mg, 182.9 µmol, 4.00 eq.) in thf (2 mL, rinsed with 2 mL to ensure complete addition), resulting in a change of color from dark red to bright yellow / grey. After stirring the reaction mixture for 5 minutes, the precipitated silver was filtered off using a short celite pad. The celite pad was rinsed with additional thf (2 mL). The solvent of the filtrate was removed under reduced pressure and the crude product precipitated from CH_2Cl_2:Et_2O 1:10 at -40 °C, furnishing **$2c^{4+}$** (53 mg, 26.0 µmol, 57%) as an orange solid.

^{1}H NMR (600 MHz, CD_3CN): δ [ppm] = 8.37 (s, 2H, Ar-CH), 8.30 (s, 2H, Ar-CH), 7.97 (s, 2H, Ar-CH), 7.78 (s, 2H, Ar-CH), 7.75 (d, *J* = 8.3 Hz, 4H, Ar-CH), 7.46 (s, 2H, Ar-CH), 7.09 (s, 2H, Ar-CH), 7.00 (s, 2H, Ar-CH), 6.75 (s, 2H, Ar-CH), 4.77 – 4.69 (m, 2H, prop-CH_2), 4.64 – 4.56 (m, 2H, prop-CH_2), 3.28 – 3.11 (m, 2H, prop-CH_2), 2.62 (s, 6H, Mes-CH_3), 2.51 (s, 6H, Mes-CH_3), 2.33 (s, 6H, Mes-CH_3), 2.17 (s, 6H, Mes-CH_3), 1.78 (s, 6H, Mes-CH_3), 1.72 (s, 6H, Mes-CH_3), 1.42 (s, 18H, $C(CH_3)_3$). Four aromatic Mes-CH protons are broad signals ~7.0 ppm. **^{13}C NMR** (151 MHz, CD_3CN, 298 K): δ [ppm] = 165.1 (C_q), 159.5 (C_q), 143.9 (C_q), 143.5 (C_q), 138.0 (C_q), 137.1 (C_q), 136.8 (C_q), 136.0 (C_q), 135.5 (CH), 135.2 (C_q), 134.5 (C_q), 131.8 (CH), 131.5 (CH), 131.4 (CH), 131.1 (C_q), 130.7 (C_q), 130.2 (C_q), 129.9 (CH), 129.8 (CH), 129.8 (CH), 128.7 (C_q), 128.1 (CH), 126.4 (CH), 60.4 (2x prop-CH_2), 36.1 ($C(CH_3)_3$), 35.9 (prop-CH_2), 31.1 ($C(CH_3)_3$), 21.2 (Mes-CH_3), 19.0 (Mes-CH_3), 18.9 (Mes-CH_3), 18.8 (Mes-CH_3), 18.3 (Mes-CH_3); **IR** [cm^{-1}]: $\tilde{v}$ = 2966.04, 2871.49, 1602.59, 1555.33, 1535.88, 1482.89, 1461.96, 1366.17, 1287.82, 1229.50, 1111.29, 1014.11, 900.82, 845.98, 802.04, 732.09, 652.61, 589.35, 574.58; **HR-MS-ESI(+)** calc. $[M+CH_3O+SbF_6]^{2+}$ $C_{77}H_{84}N_6SbF_6CH_3O^{2+}$ 679.2936; found 679.2937; **UV-vis:** λ_{max}: 316 nm (ε = 30714 cm^{-1} M^{-1}), 359 nm (ε = 34903 cm^{-1} M^{-1}).

3. NMR Spectra

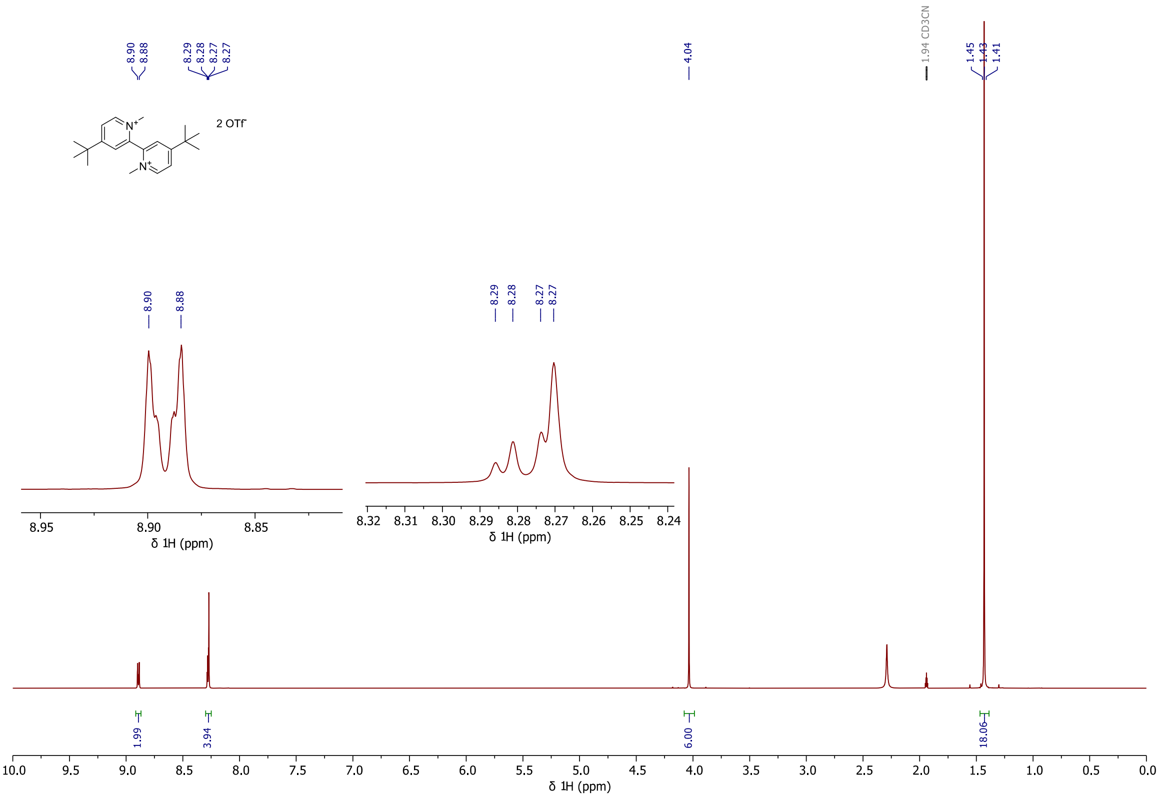

Figure S1. **^{1}H NMR** (500 MHz, CD_3CN, 298K) of **1a**.

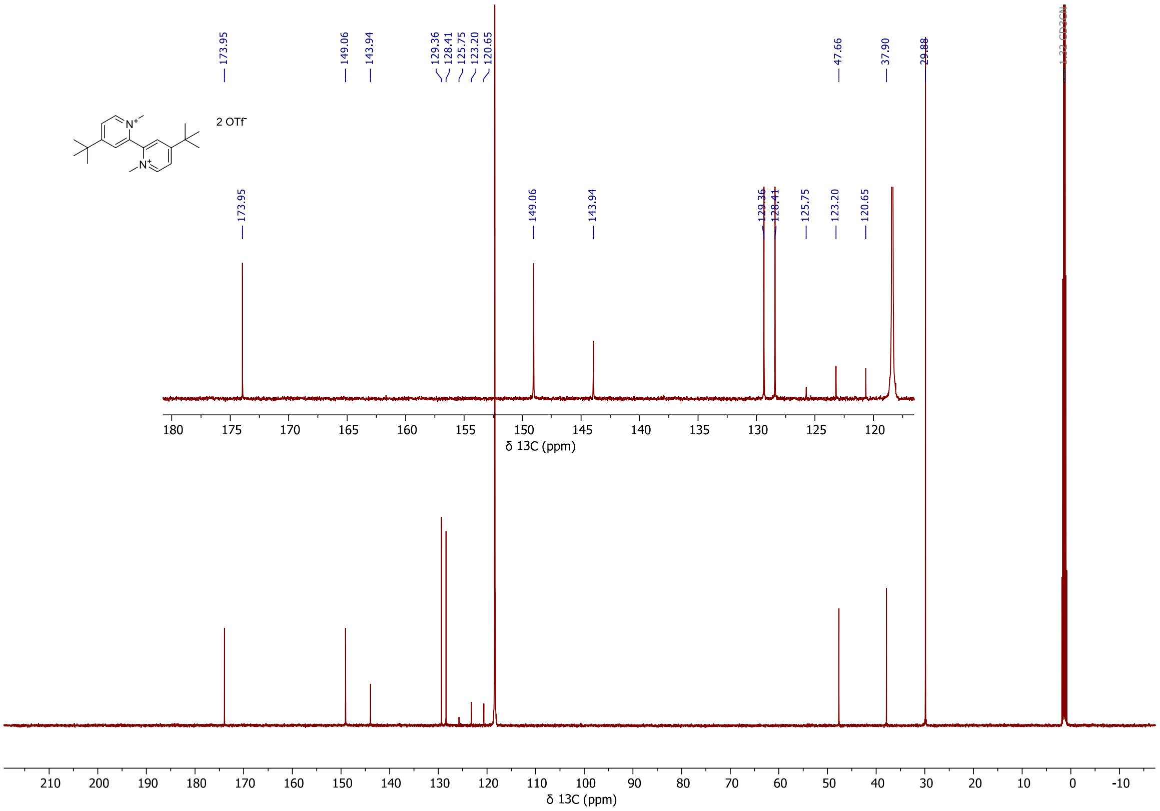

Figure S2. **^{13}C {^{1}H} NMR** (126 MHz, CD_3CN, 298K) of **1a**.

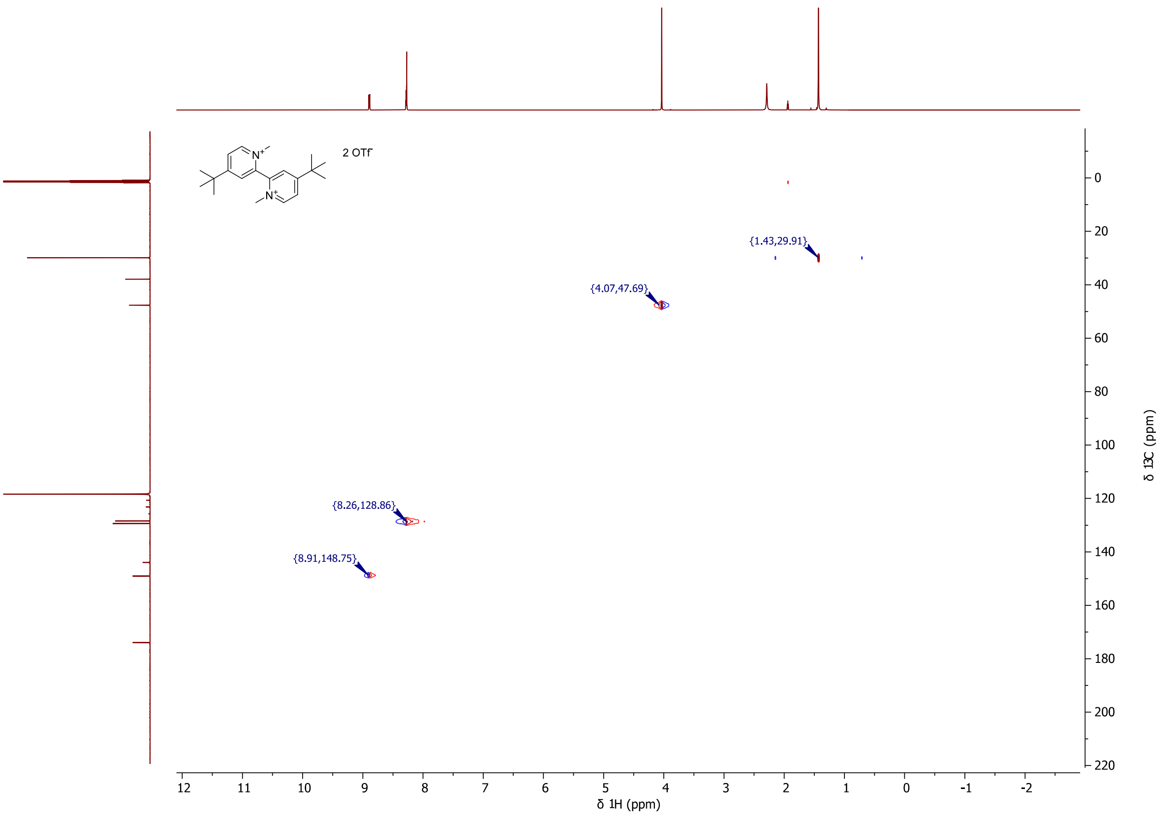

Figure S3. **^{1}H/^{13}C HSQC** (500/126 MHz, CD_3CN, 298K) of **1a**.

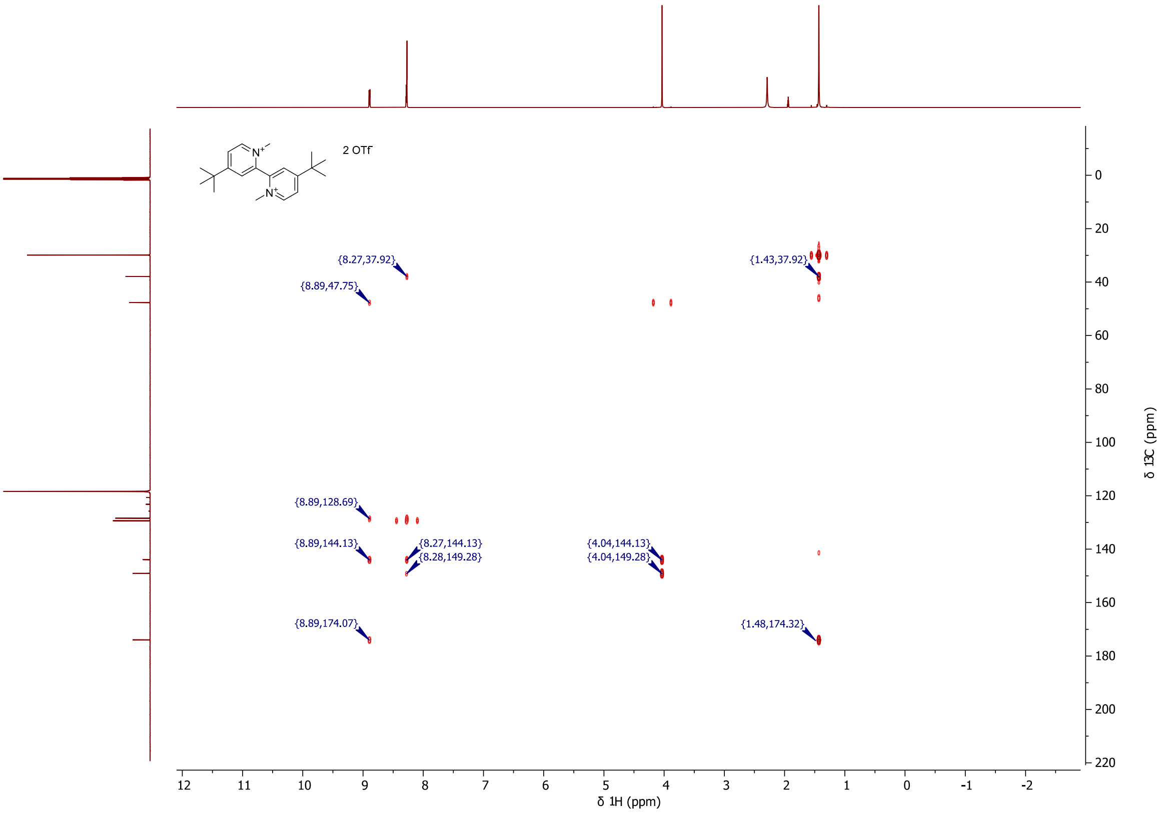

Figure S4. **^{1}H/^{13}C HMBC** (500/126 MHz, CD_3CN, 298K) of **1a**.

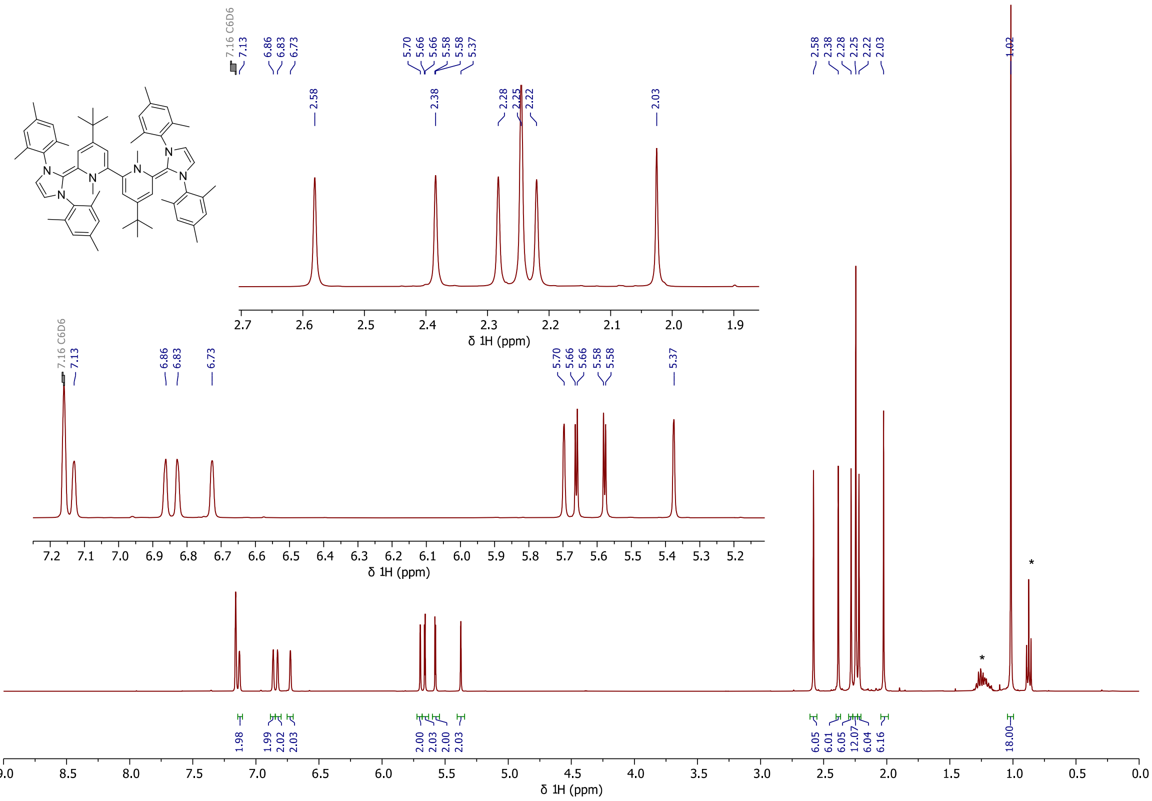

Figure S5. [1]**H NMR** (400 MHz, C_6D_6, 298K) of **2a**, residual pentane marked with *.

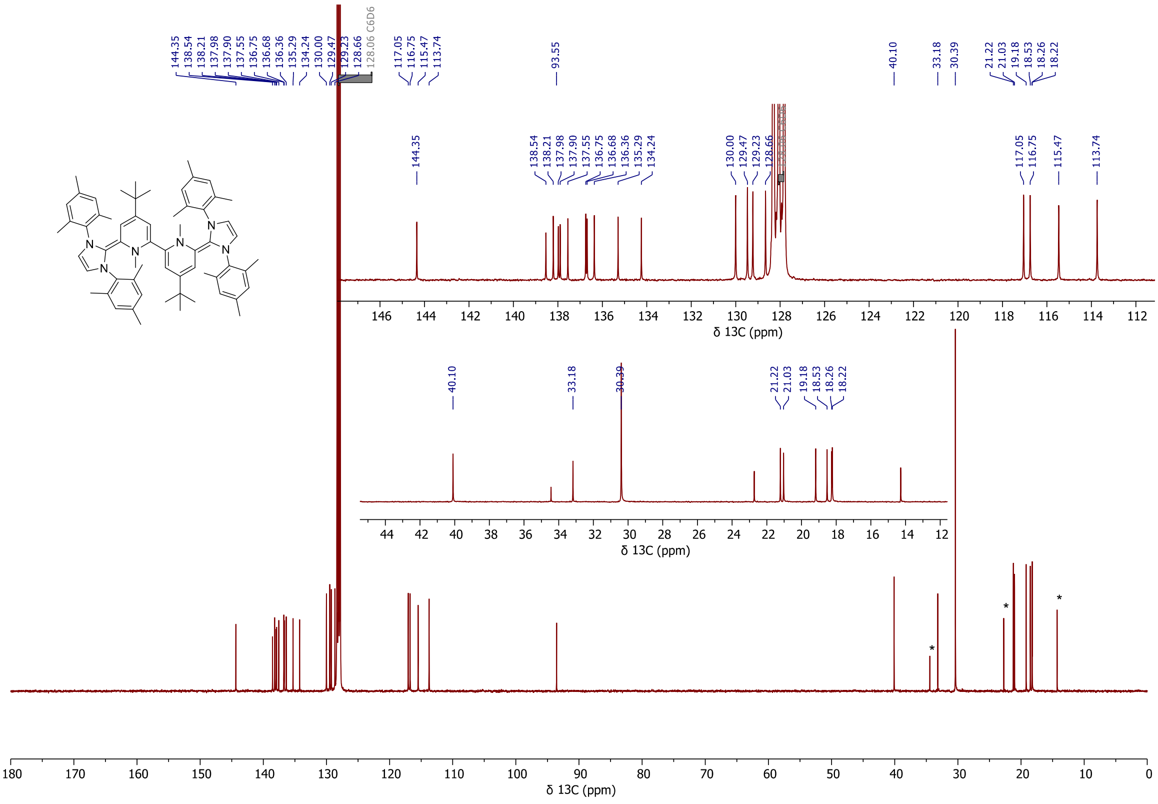

Figure S6. **^{13}C {^{1}H} NMR** (100 MHz, C_6D_6, 298K) of **2a**, residual pentane marked with *.

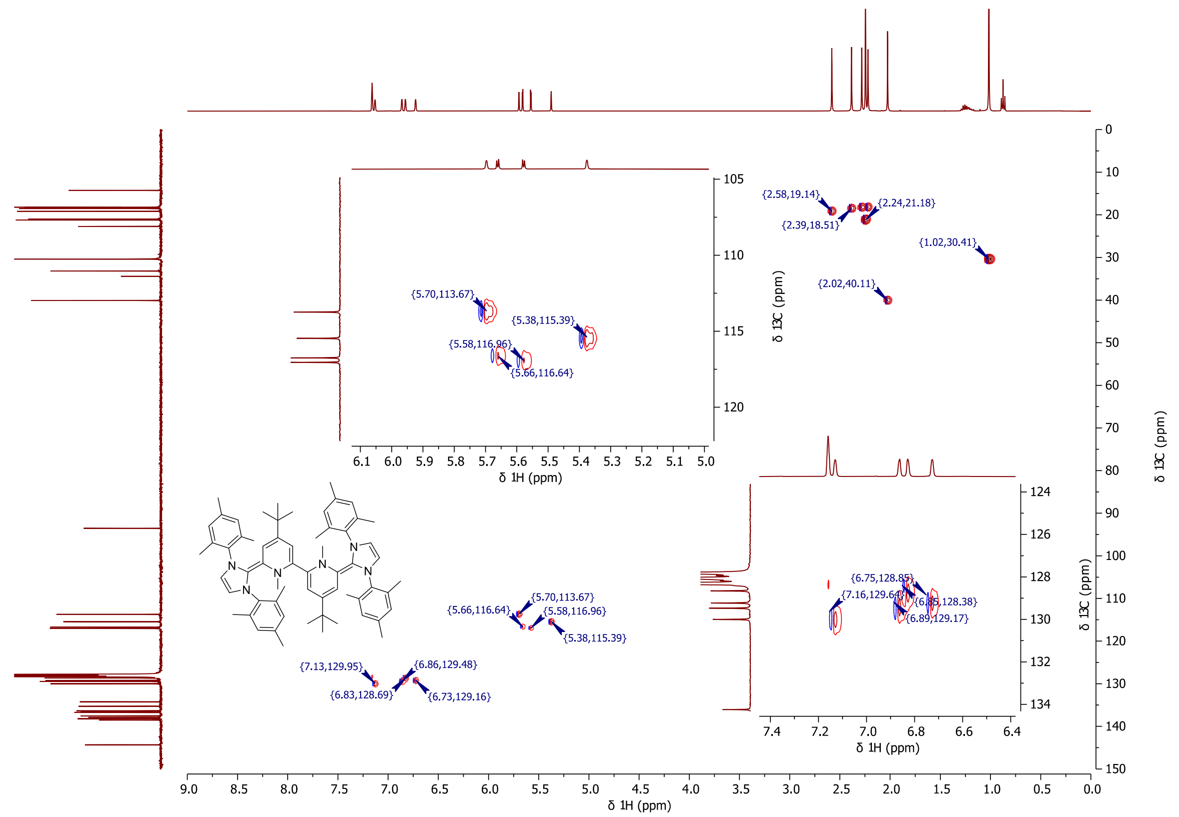

Figure S7. **^{1}H/^{13}C HSQC** (400/100 MHz, C_6D_6, 298K) of **2a**.

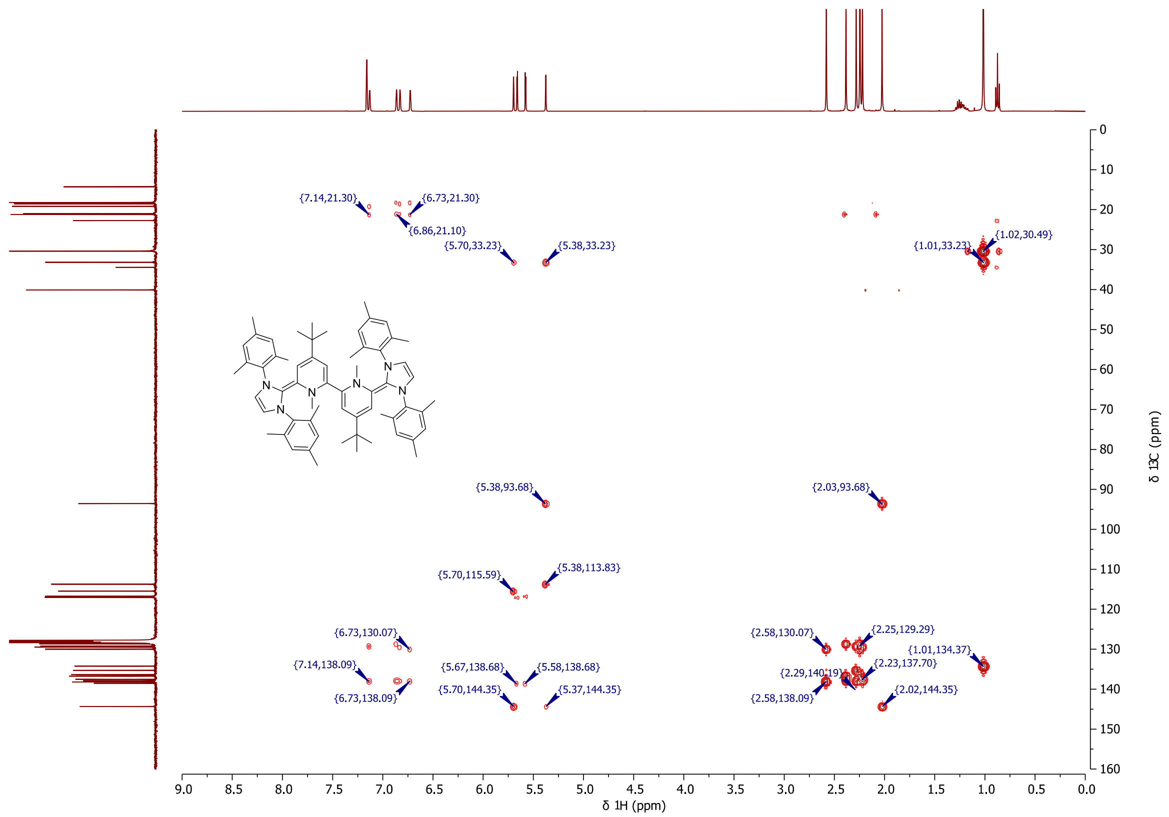

Figure S8. **^{1}H/^{13}C HMBC** (400/100 MHz, C_6D_6, 298K) of **2a**.

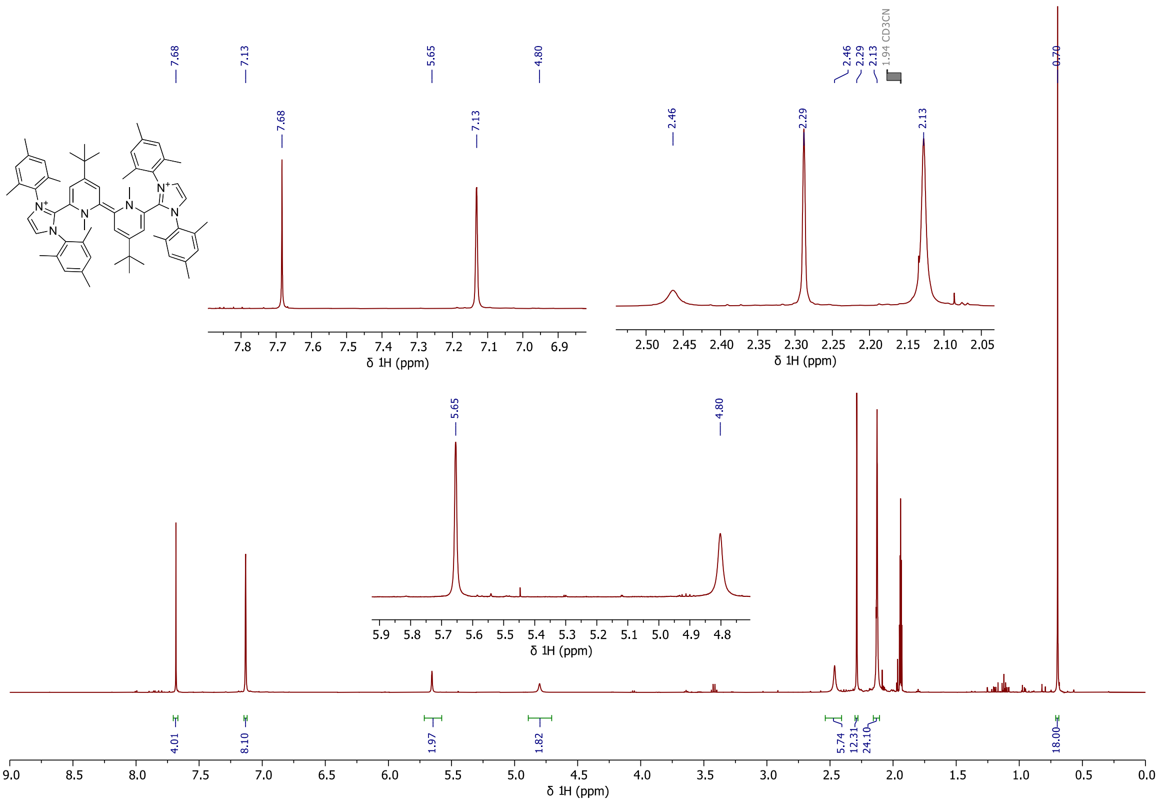

Figure S9. **^{1}H NMR** (500 MHz, CD_3CN, 298K) of **2a^{2+}**.

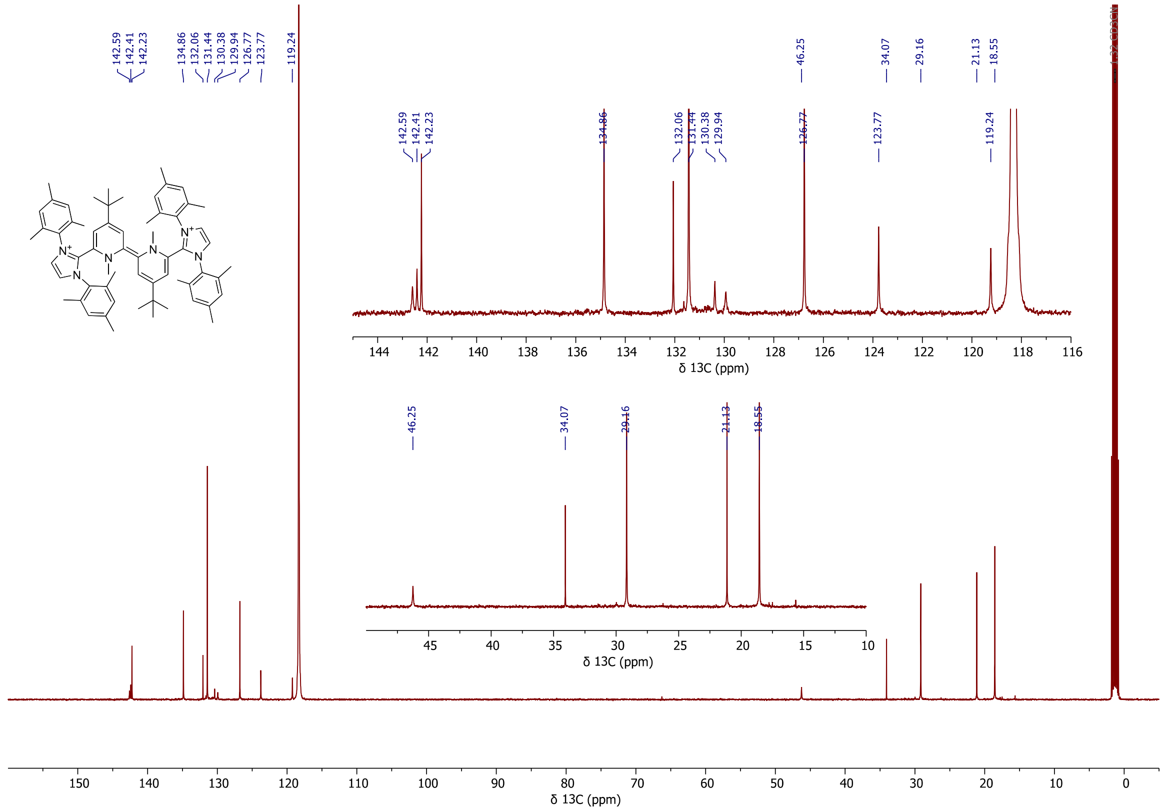

Figure S10. **^{13}C {^{1}H} NMR** (126 MHz, CD_3CN, 298K) of **2a^{2+}**.

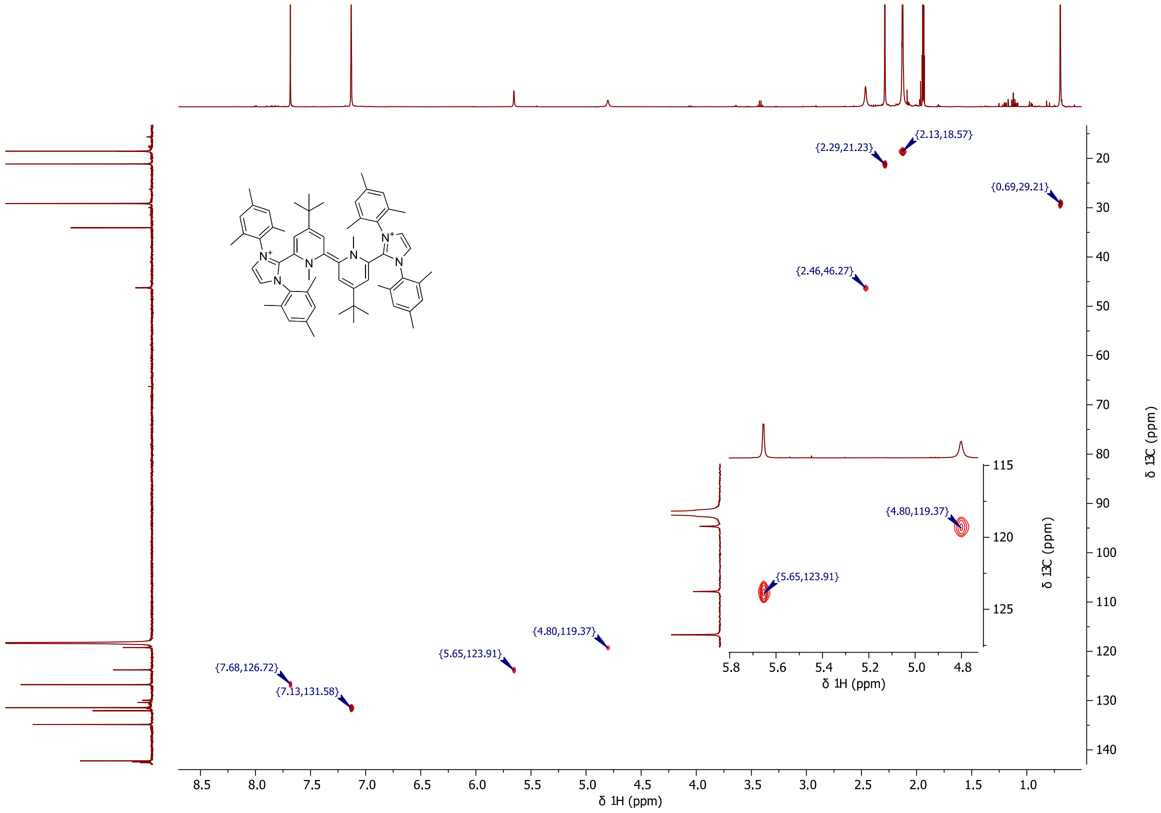

Figure S11. **$^1H/^{13}C$ HSQC** (500/126 MHz, CD_3CN, 298K) of **$2a^{2+}$**.

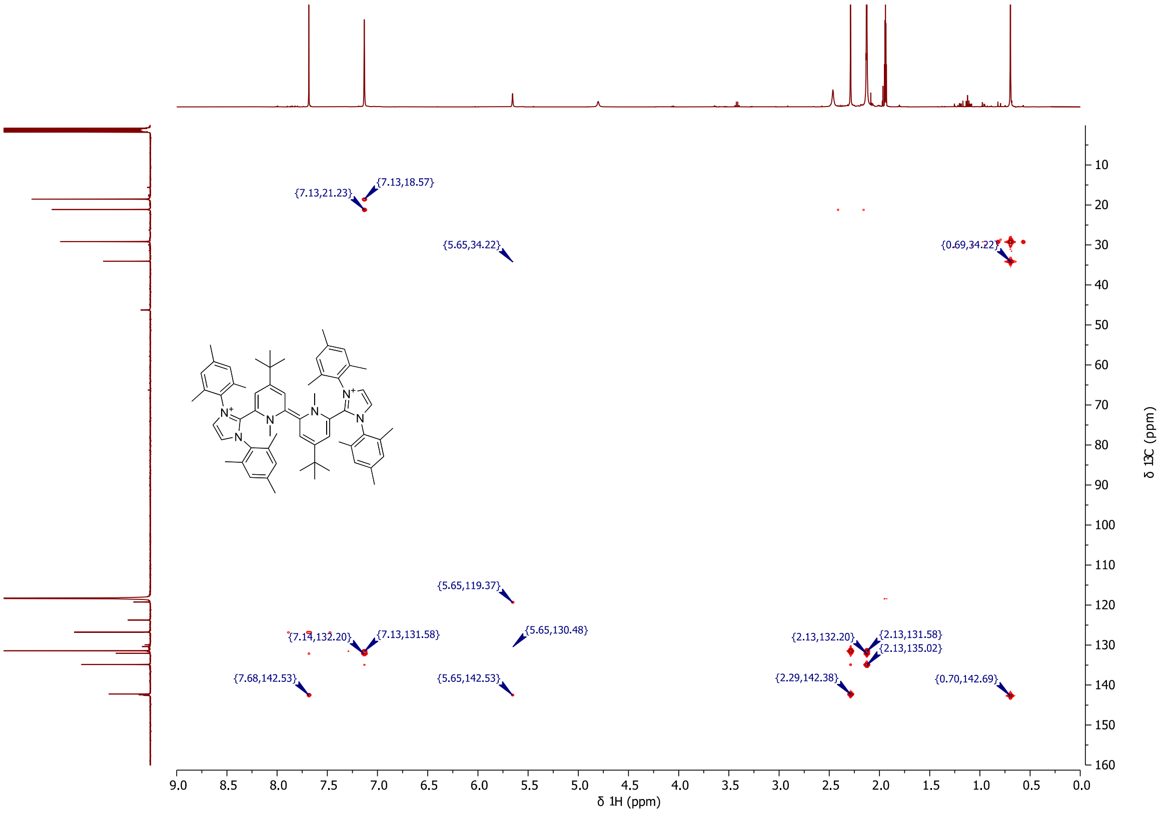

Figure S12. **$^1H/^{13}C$ HMBC** (500/126 MHz, CD_3CN, 298K) of **$2a^{2+}$**.

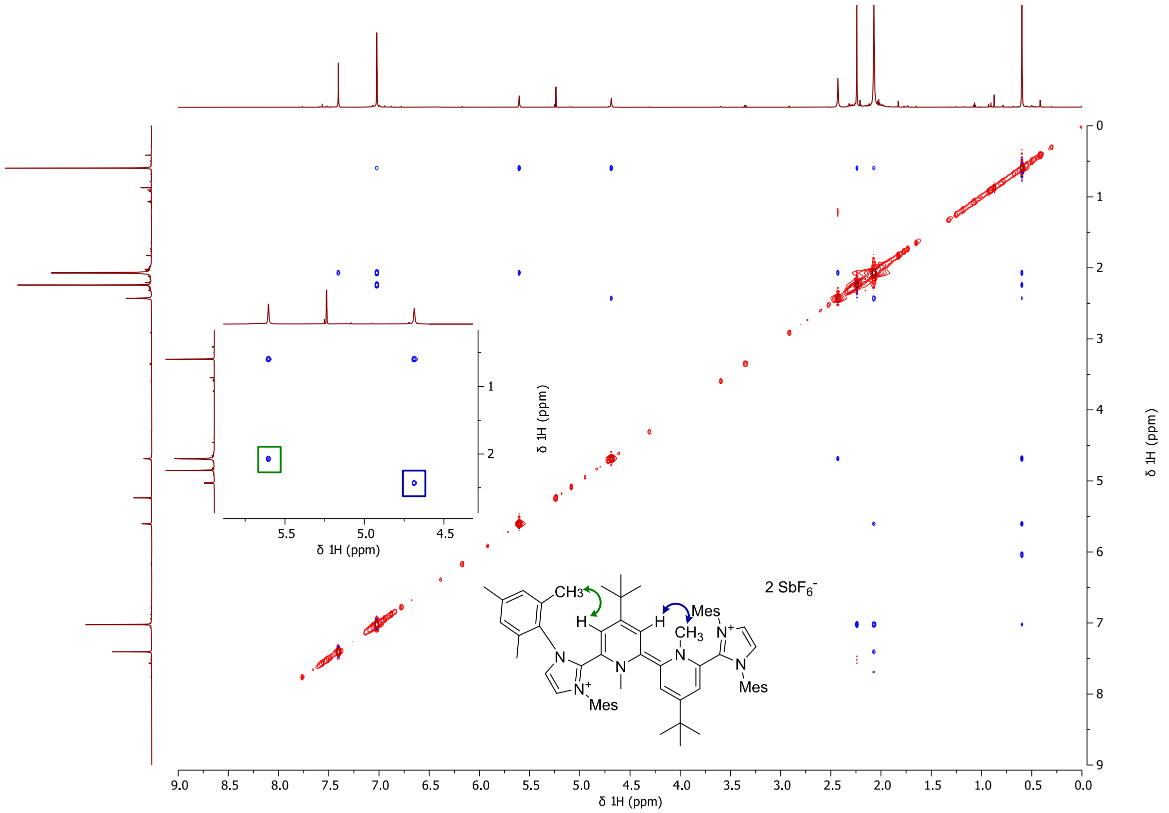

Figure S13. **^{1}H/^{1}H NOESY** (700/700 MHz, CD_2Cl_2, 298K) of **$2a^{2+}$**.

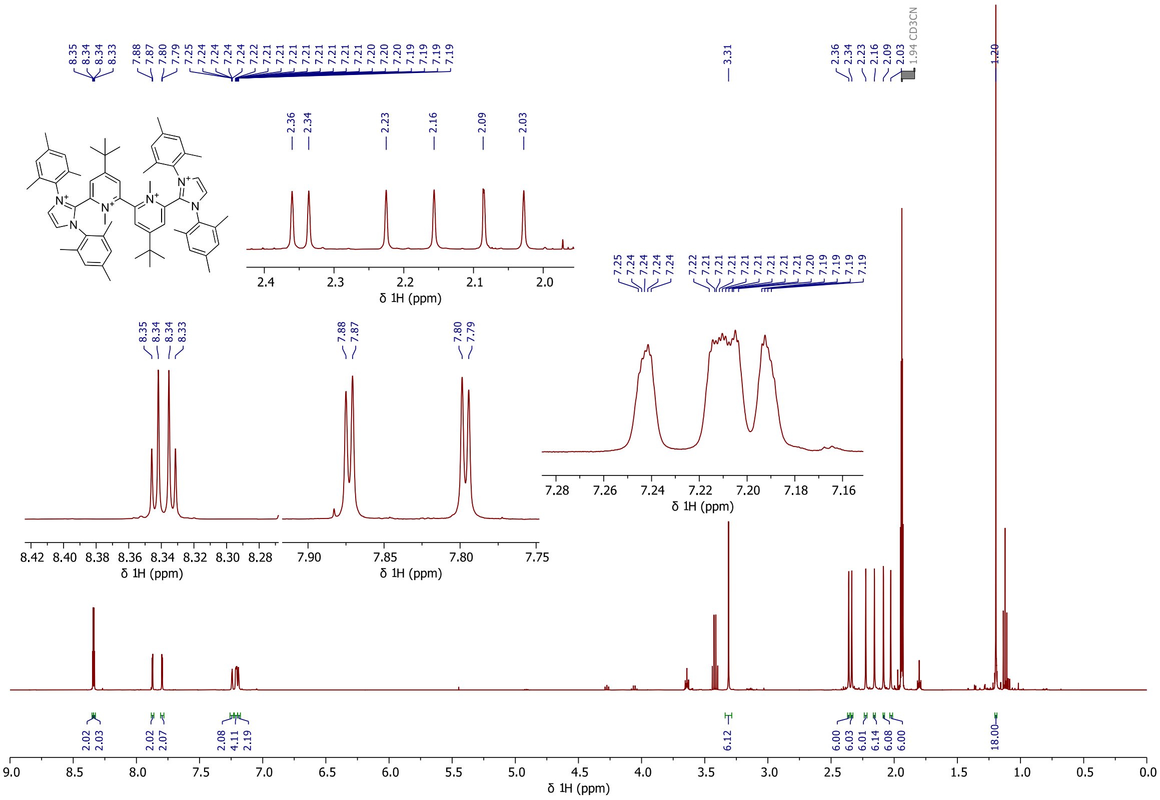

Figure S14. **^{1}H NMR** (500 MHz, CD_3CN, 298K) of **$2a^{4+}$**.

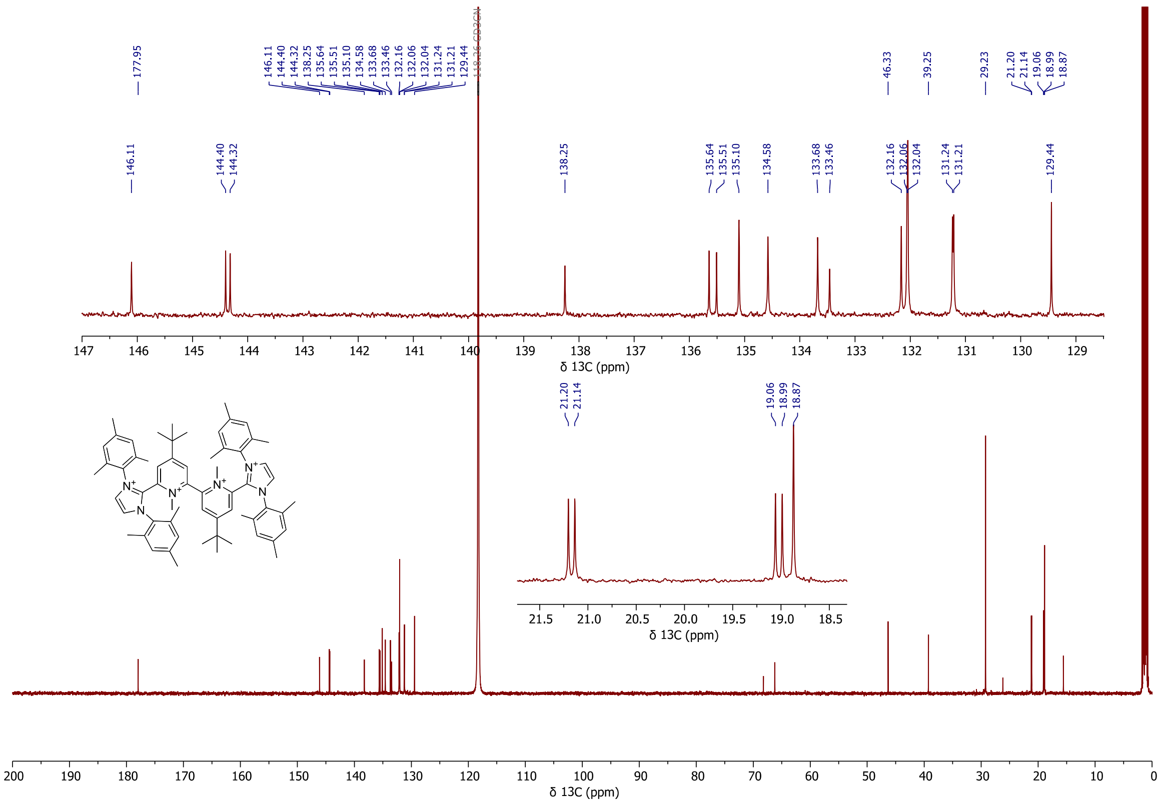

Figure S15. ^{13}C {^{1}H} **NMR** (126 MHz, CD_3CN, 298K) of **2a**$^{4+}$.

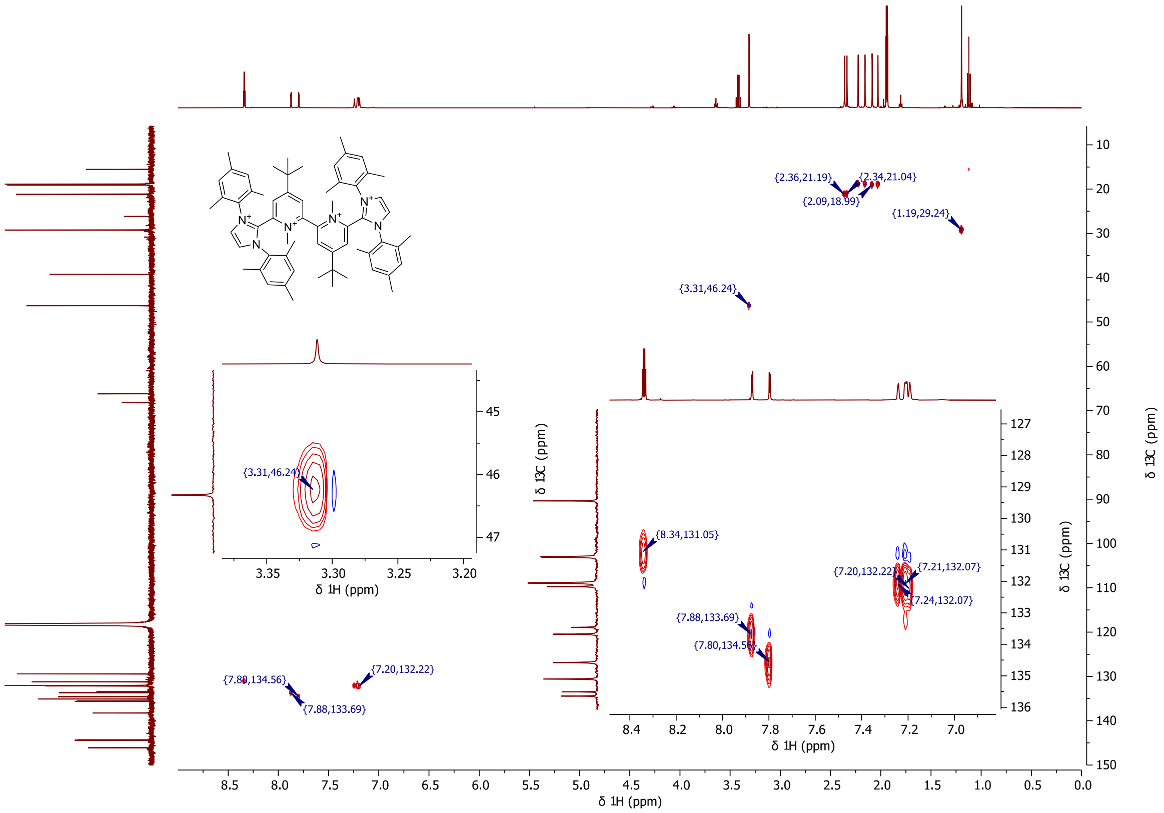

Figure S16. ^{1}H/^{13}C **HSQC** (500/126 MHz, CD_3CN, 298K) of **2a**$^{4+}$.

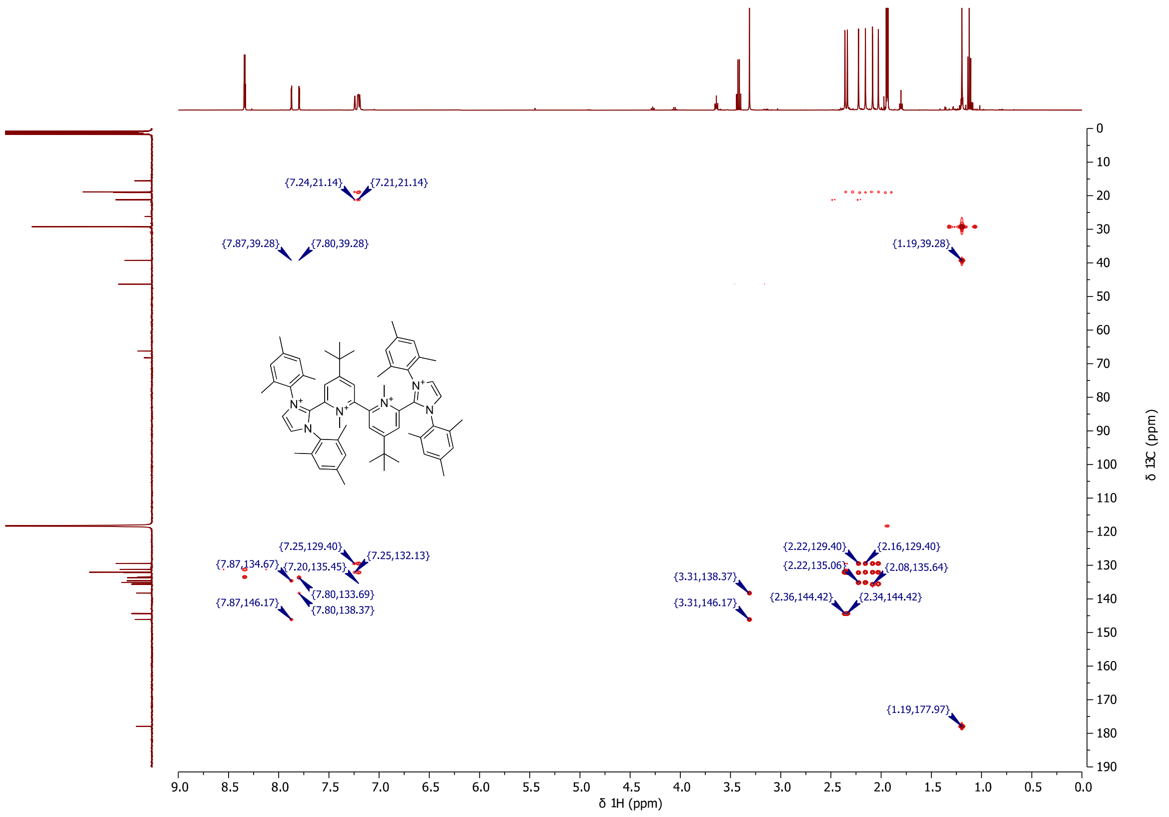

Figure S17. **^{1}H/^{13}C HMBC** (500/126 MHz, CD_3CN, 298K) of **$2a^{4+}$**.

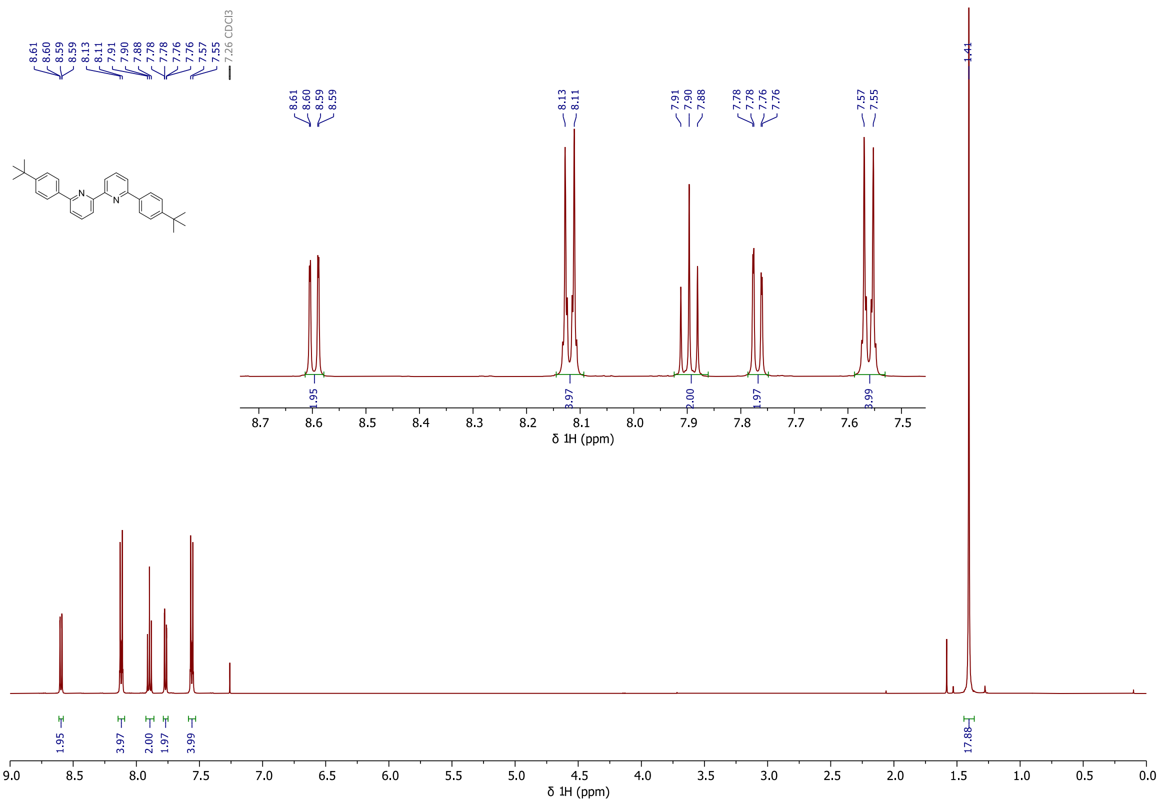

Figure S18. **^{1}H NMR** (500 MHz, $CDCl_3$, 298K) of **6,6'-bis(4-(tert-butyl)phenyl)-2,2'-bipyridine.**

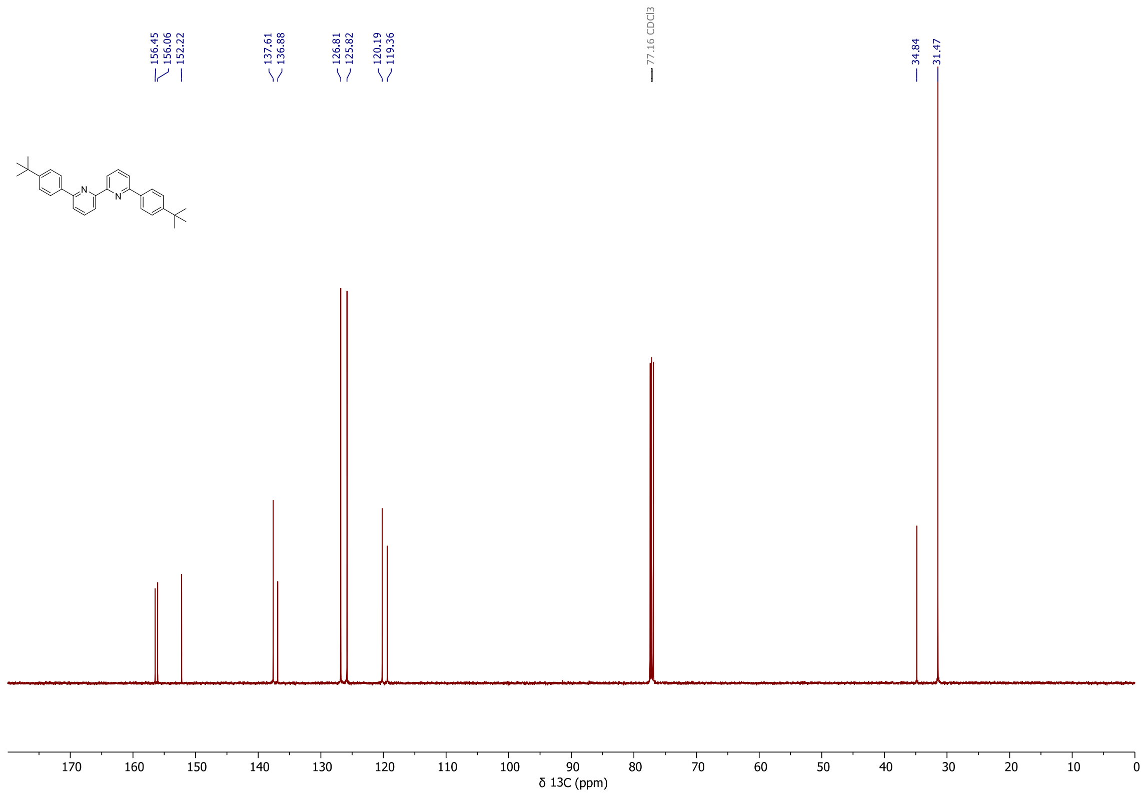

Figure S19. **^{13}C {^{1}H} NMR** (126 MHz, $CDCl_3$, 298K) of **6,6'-bis(4-(tert-butyl)phenyl)-2,2'-bipyridine**.

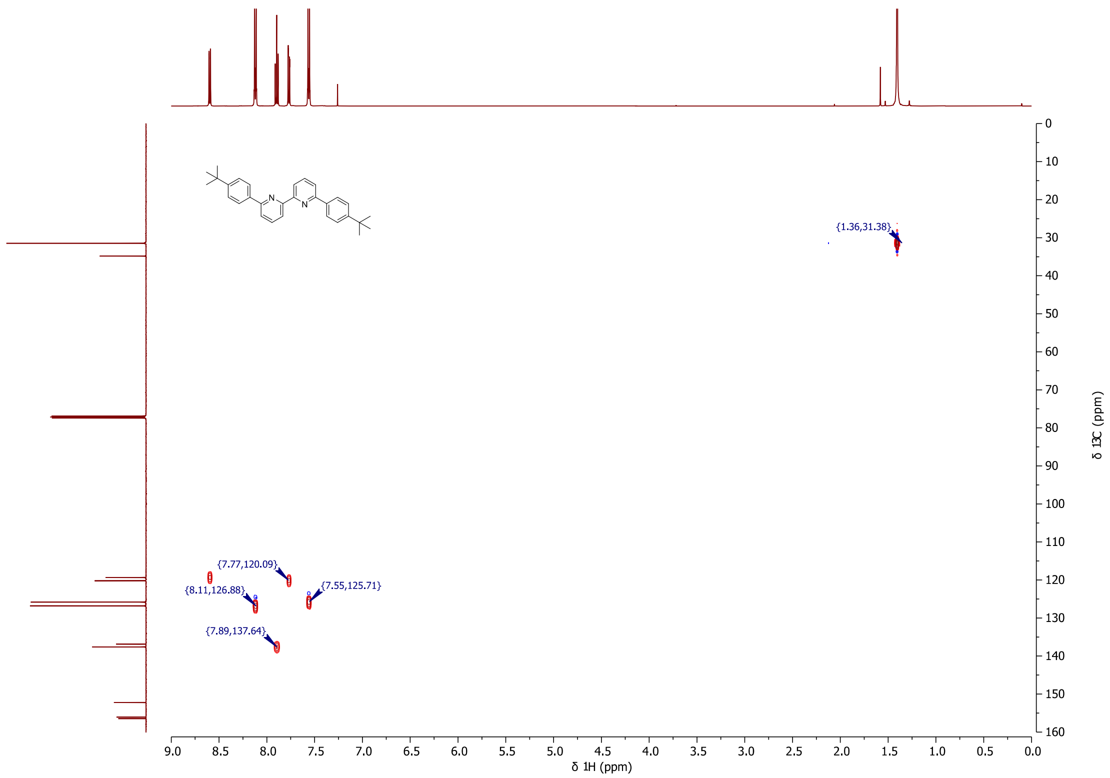

Figure S20. **^{1}H/^{13}C HSQC** (500/126 MHz, $CDCl_3$, 298K) of **6,6'-bis(4-(tert-butyl)phenyl)-2,2'-bipyridine**.

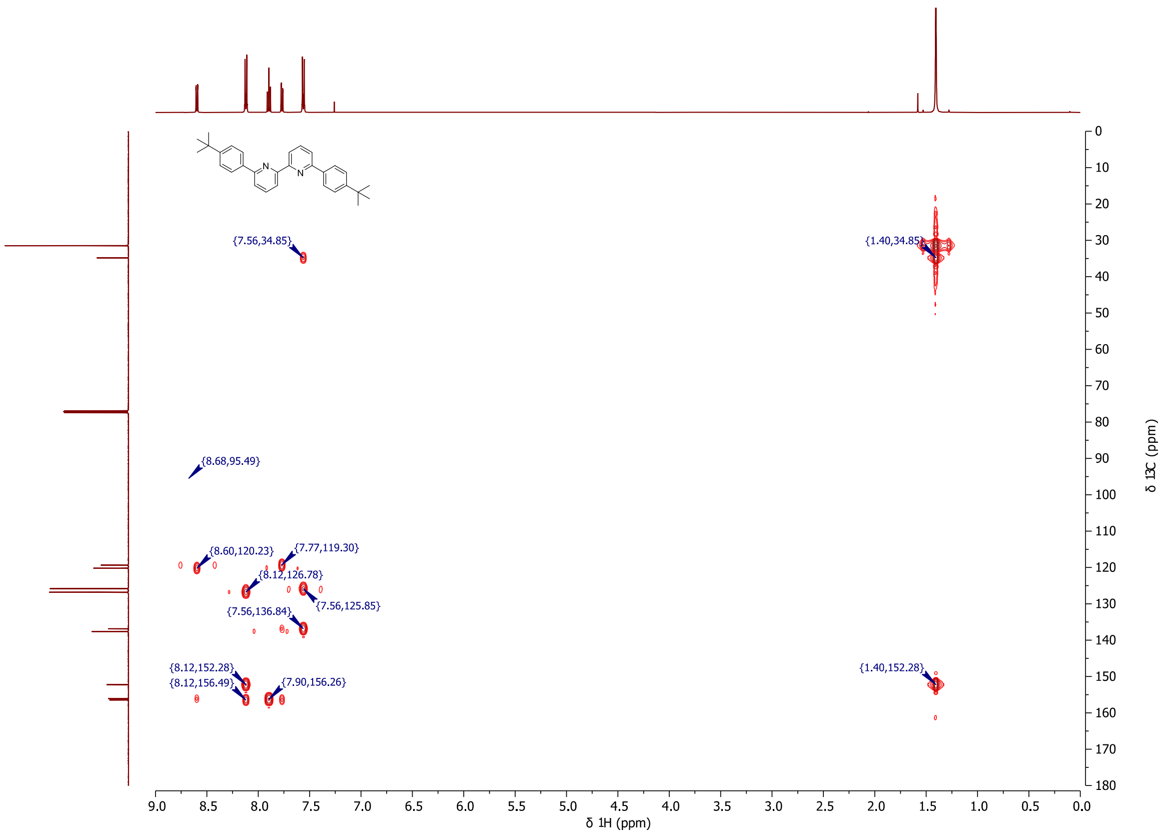

Figure S21. **^{1}H/^{13}C HMBC** (500/126 MHz, $CDCl_3$, 298K) of **6,6'-bis(4-(tert-butyl)phenyl)-2,2'-bipyridine**.

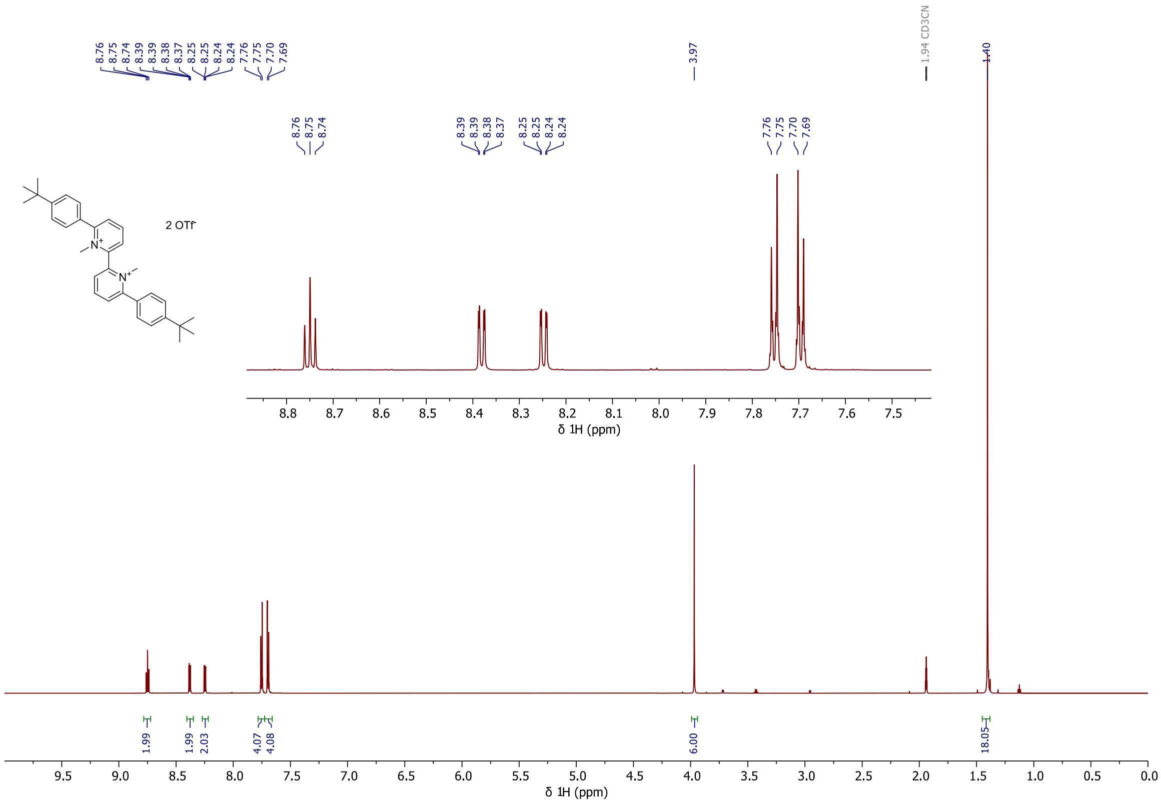

Figure S22. **^{1}H NMR** (700 MHz, CD_3CN, 298K) of **1b**.

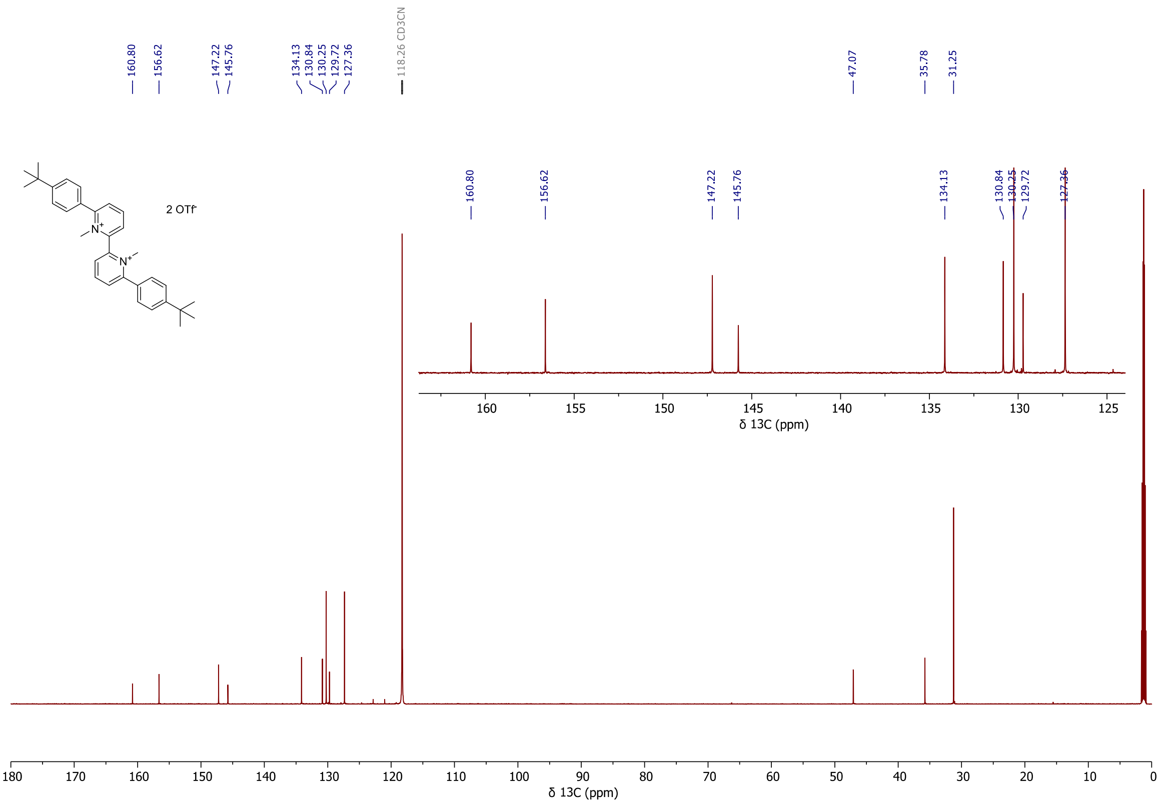

Figure S23. ^{13}C {^{1}H} **NMR** (176 MHz, CD_3CN, 298K) of **1b**.

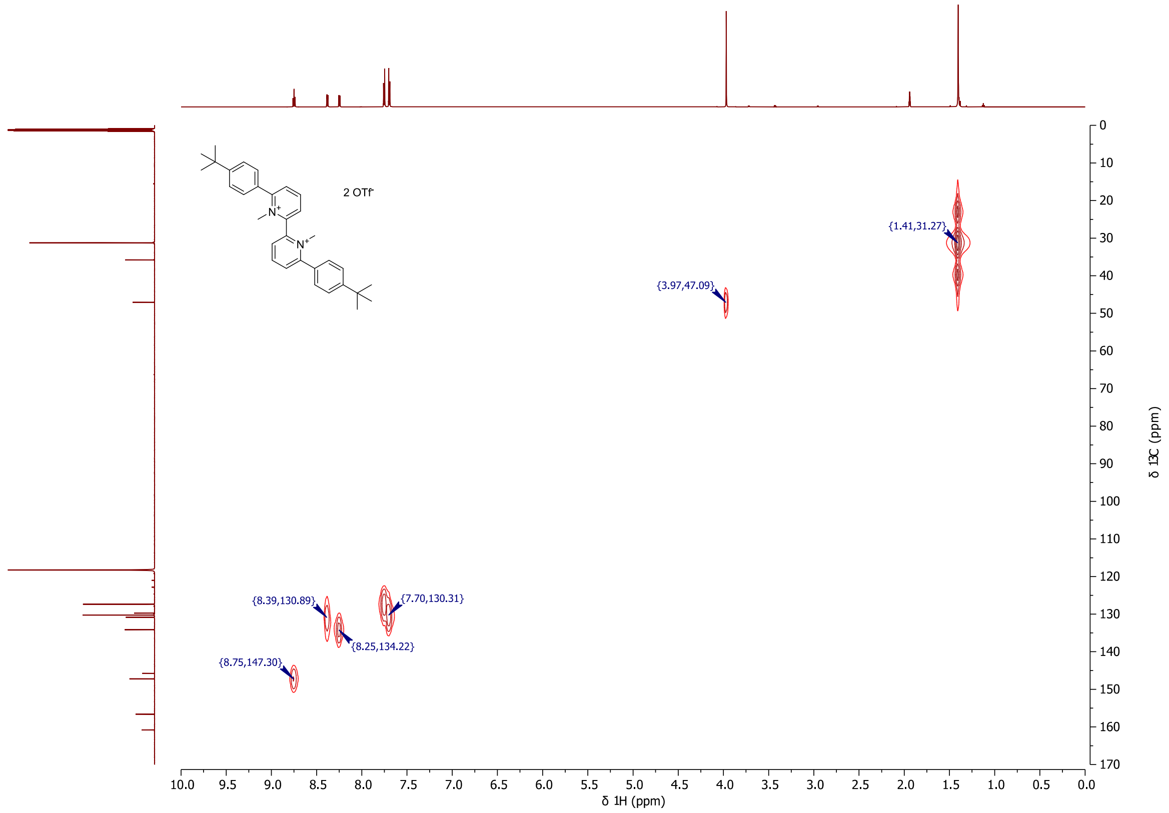

Figure S24. ^{1}H/^{13}C **HSQC** (700/176 MHz, CD_3CN, 298K) of **1b**.

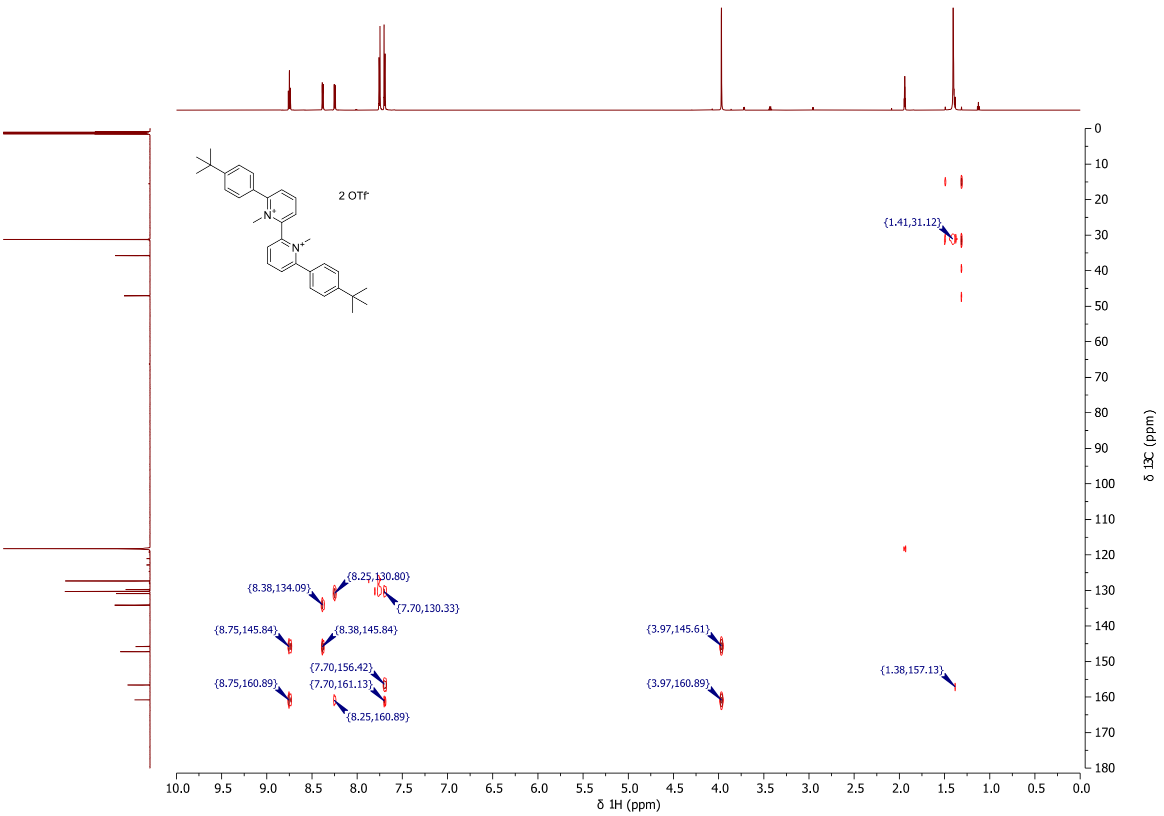

Figure S25. **^{1}H/^{13}C HMBC** (700/176 MHz, CD_3CN, 298K) of **1b**.

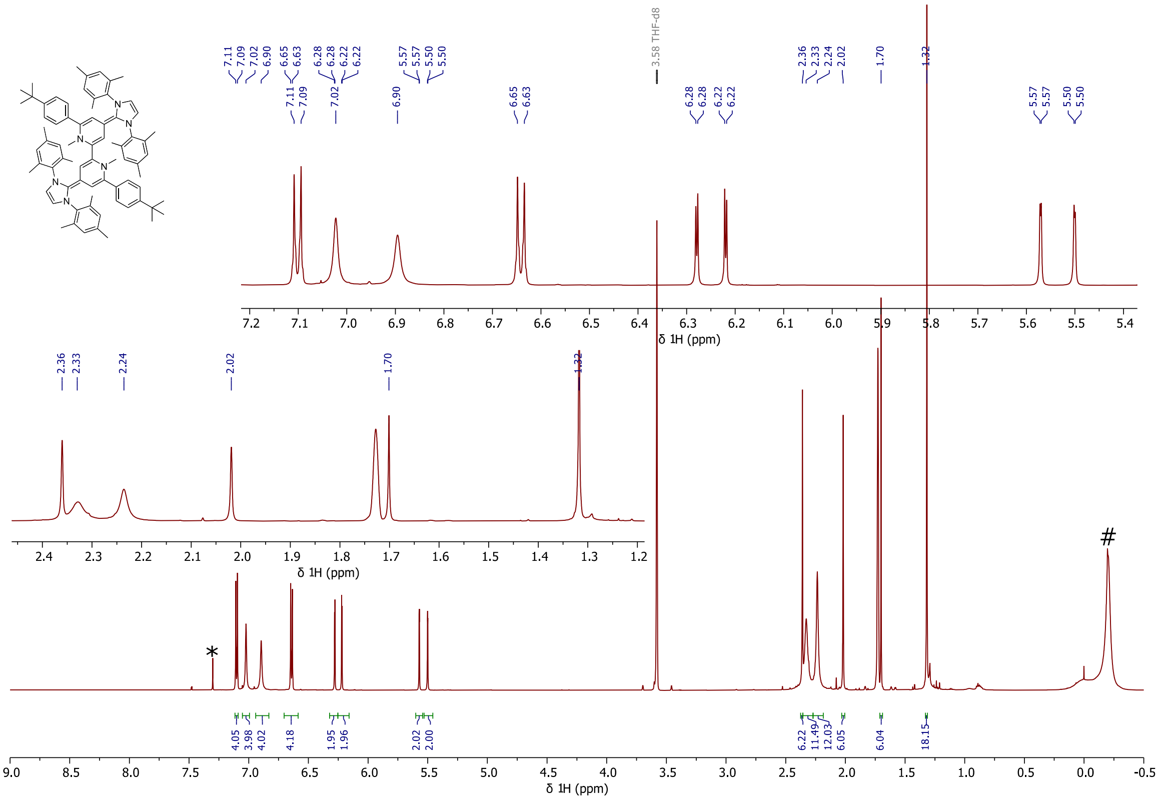

Figure S26. **^{1}H NMR** (600 MHz, d_8-THF, 298K) of **2b**. C_6D_6/C_6H_6 marked with *, KHMDS/HMDS marked with #.

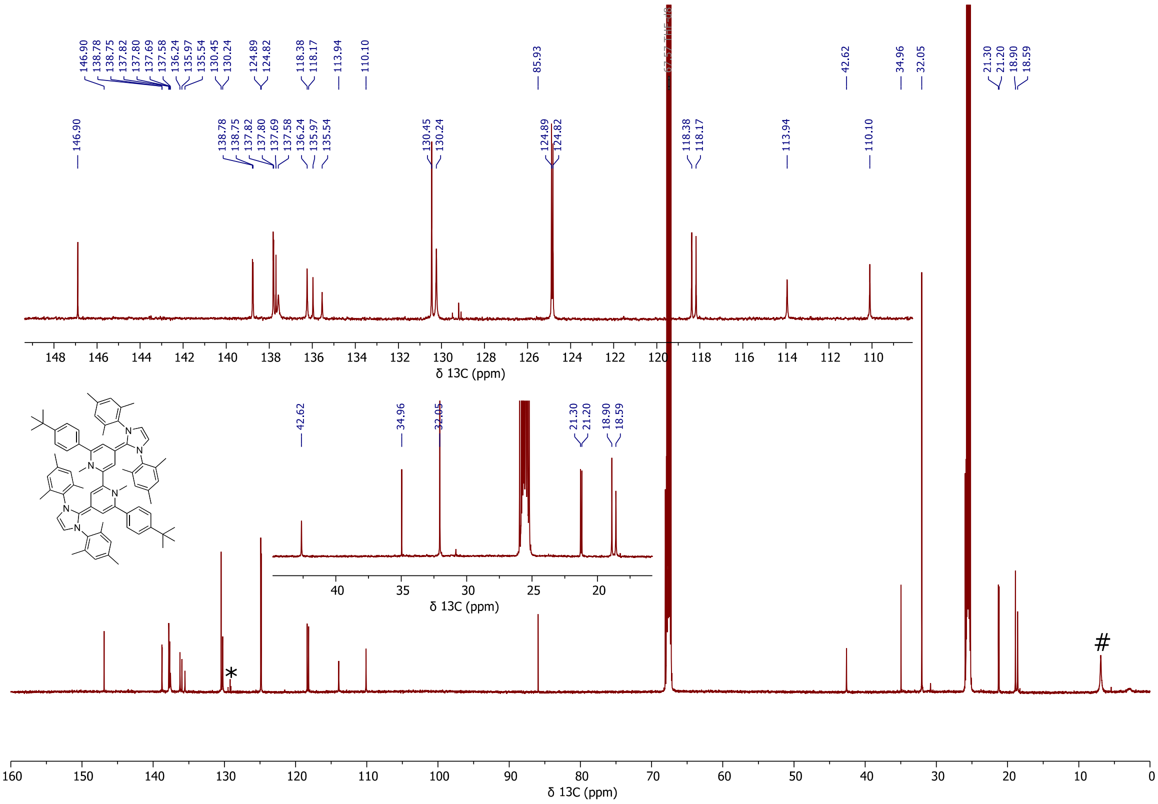

Figure S27. **^{13}C {^{1}H} NMR** (151 MHz, d$_8$-THF, 298K) of **2b**. C_6D_6/C_6H_6 marked with *, KHMDS/HMDS marked with #.

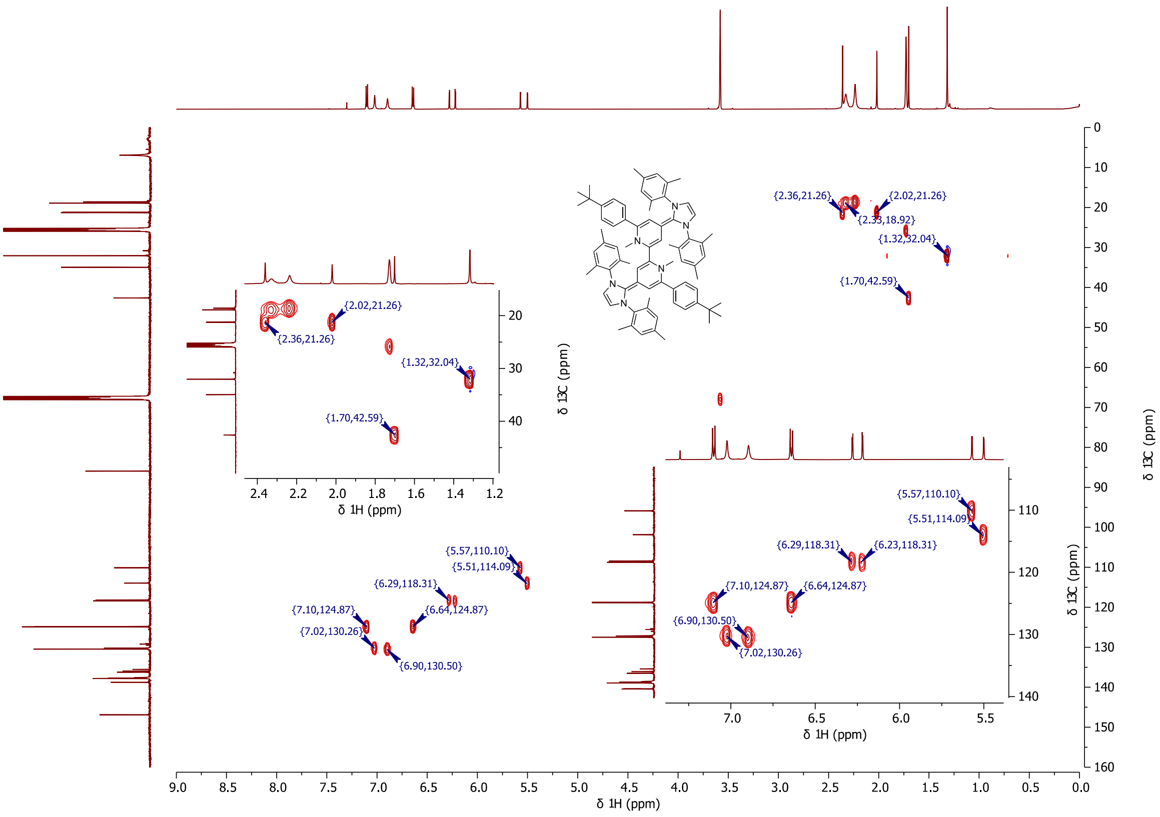

Figure S28. **^{1}H/^{13}C HSQC** (600/151 MHz, d$_8$-THF, 298K) of **2b**.

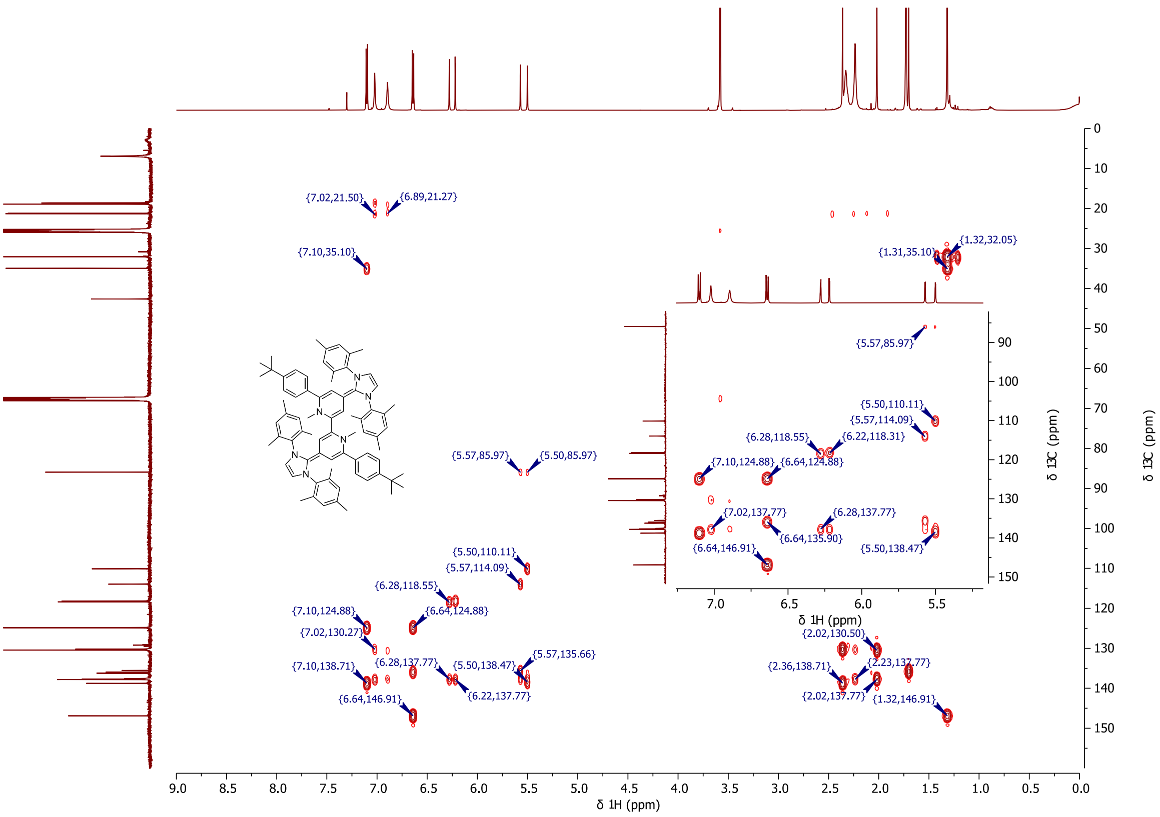

Figure S29. **$^{1}H/^{13}C$ HMBC** (600/151 MHz, d_8-THF, 298K) of **2b**.

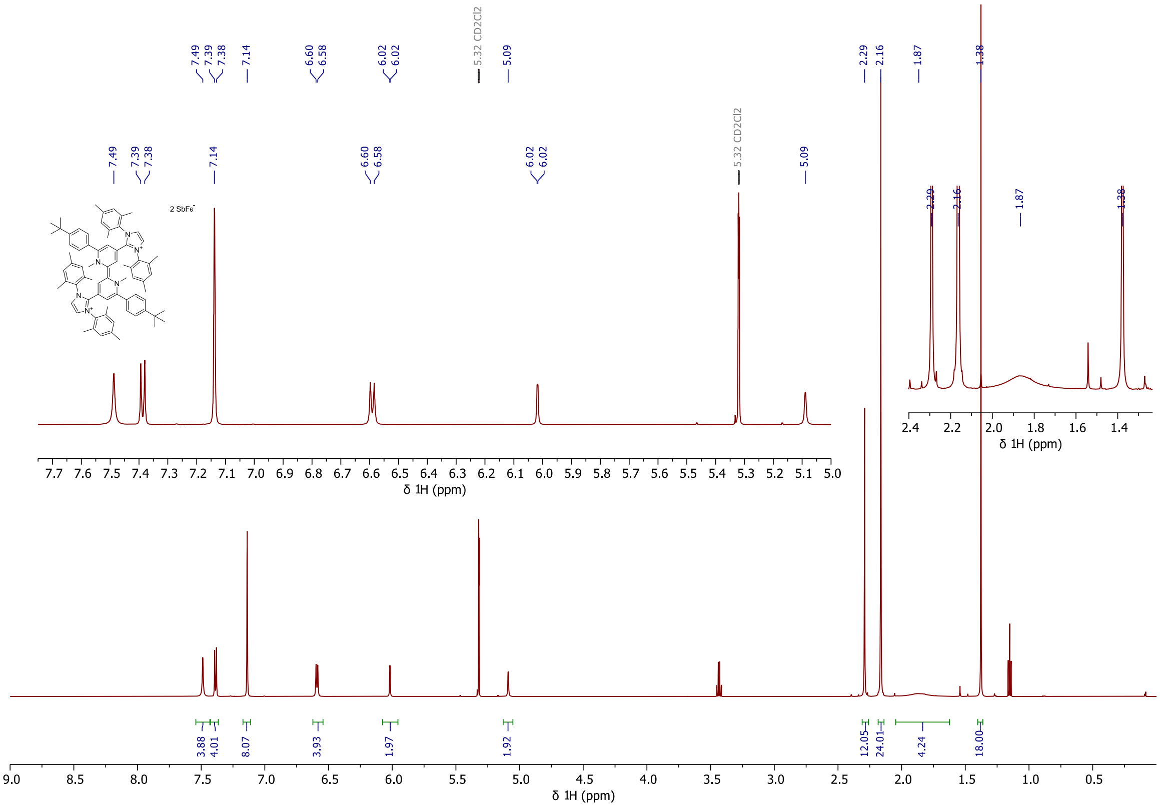

Figure S30. **^{1}H NMR** (600 MHz, CD_2Cl_2, 298K) of **$2b^{2+}$**.

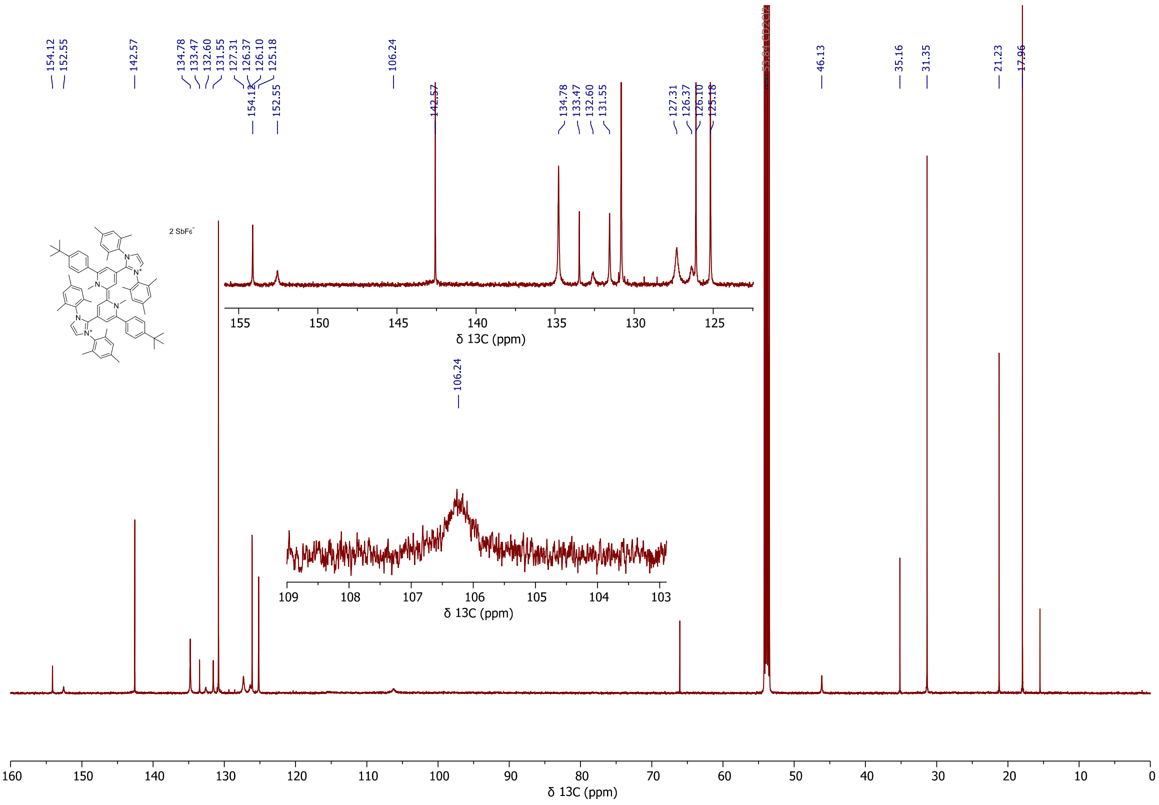

Figure S31. **^{13}C {^{1}H} NMR** (151 MHz, CD_2Cl_2, 298K) of **2b^{2+}**.

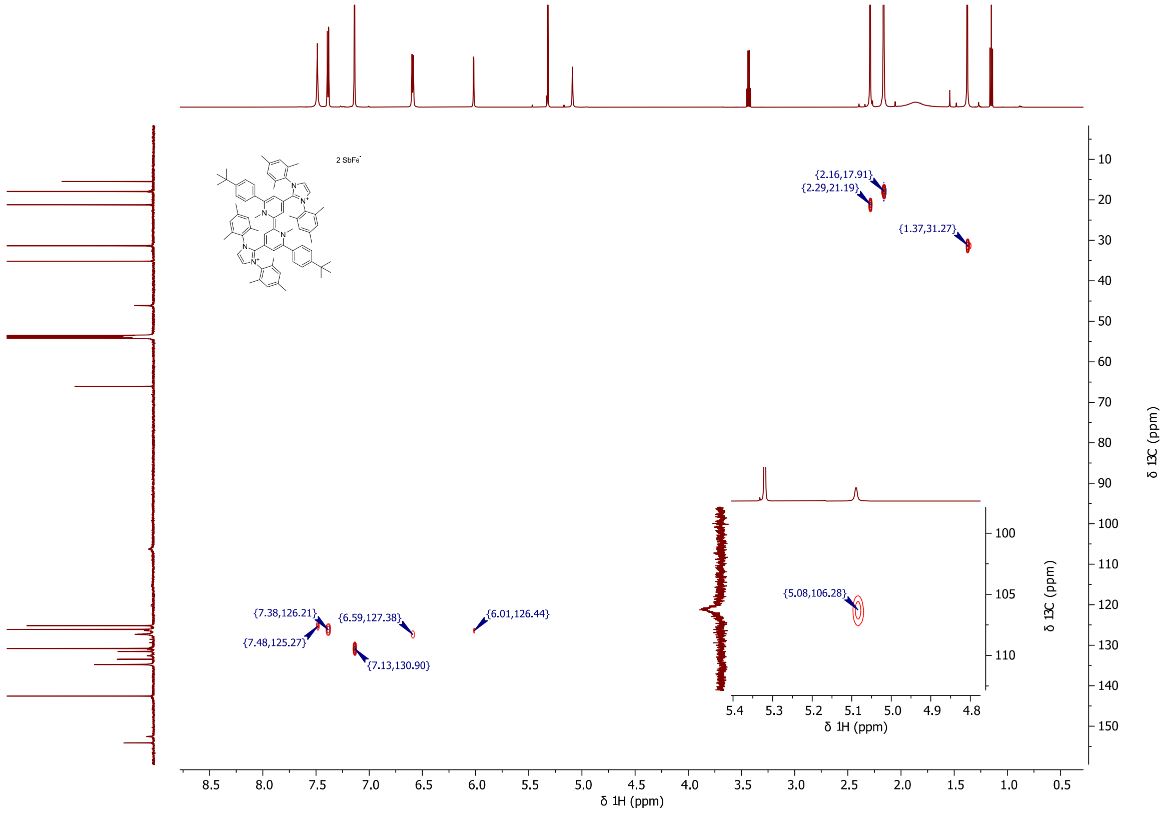

Figure S32. **^{1}H/^{13}C HSQC** (600/151 MHz, CD_2Cl_2, 298K) of **2b^{2+}**.

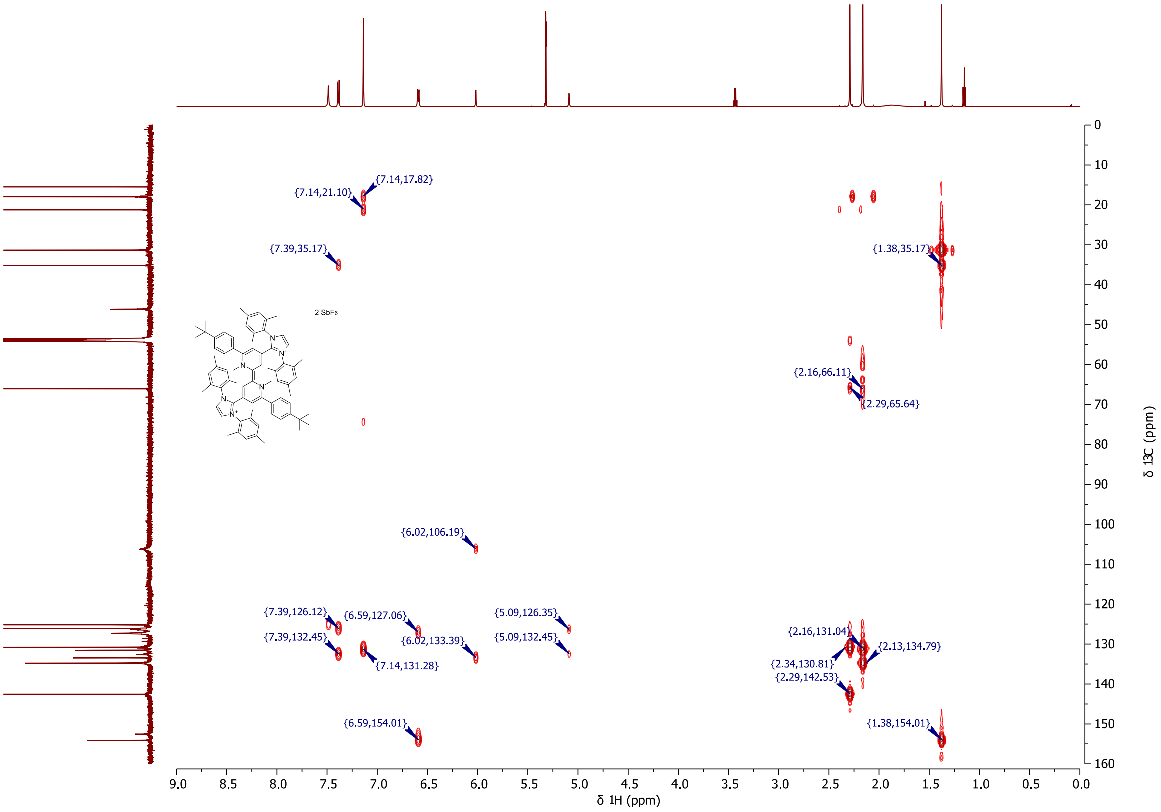

Figure S33. **^{1}H/^{13}C HMBC** (600/151 MHz, CD_2Cl_2, 298K) of **$2b^{2+}$**.

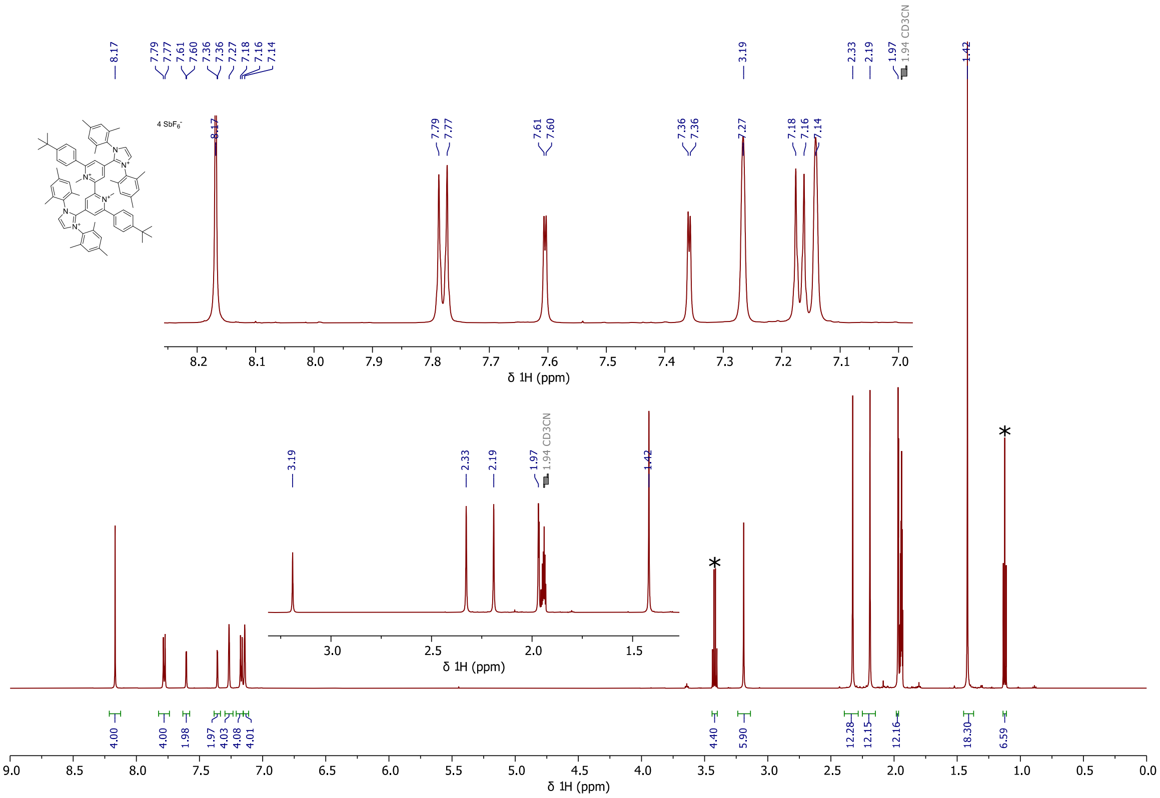

Figure S34. **^{1}H NMR** (600 MHz, CD_3CN, 298K) of **$2b^{4+}$**; 1 eq. of Et_2O is marked with *.

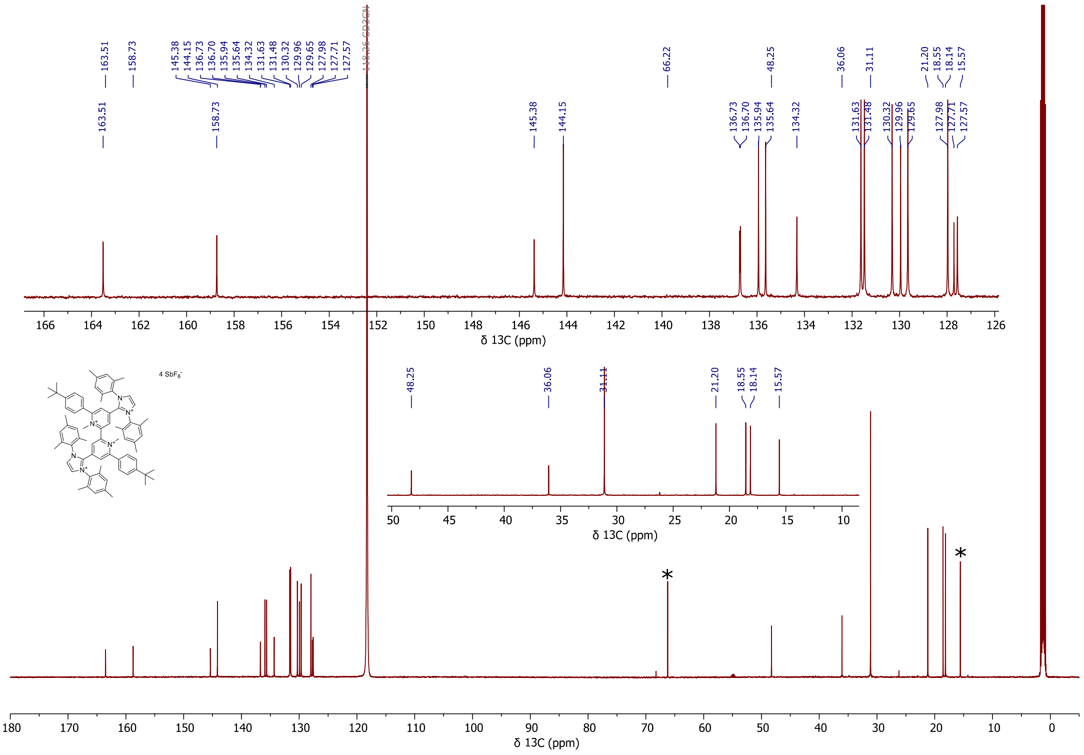

Figure S35. **^{13}C {^{1}H} NMR** (151 MHz, CD_3CN, 298K) of **$2b^{4+}$**; 1 eq. of Et_2O is marked with *

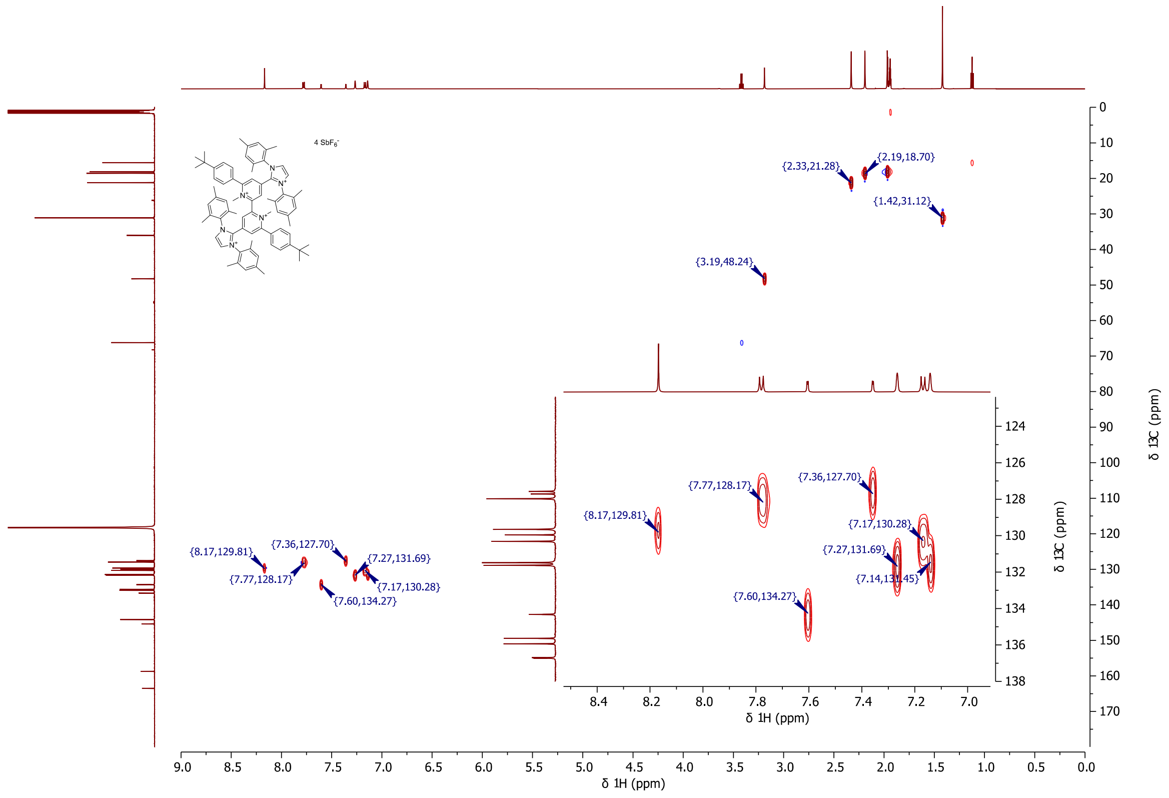

Figure S36. **$^{1}H/^{13}C$ HSQC** (600/151 MHz, CD_3CN, 298K) of **$2b^{4+}$**.

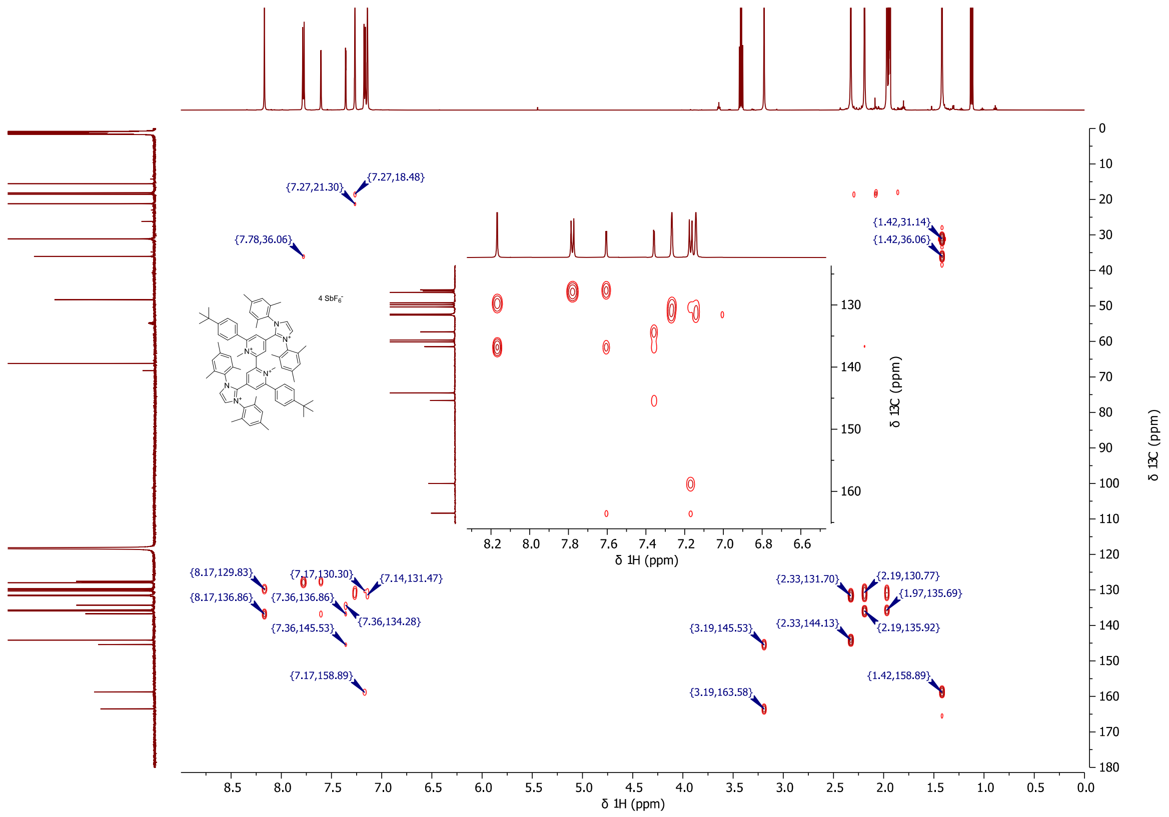

Figure S37. **^{1}H/^{13}C HMBC** (600/151 MHz, CD_3CN, 298K) of **$2b^{4+}$**.

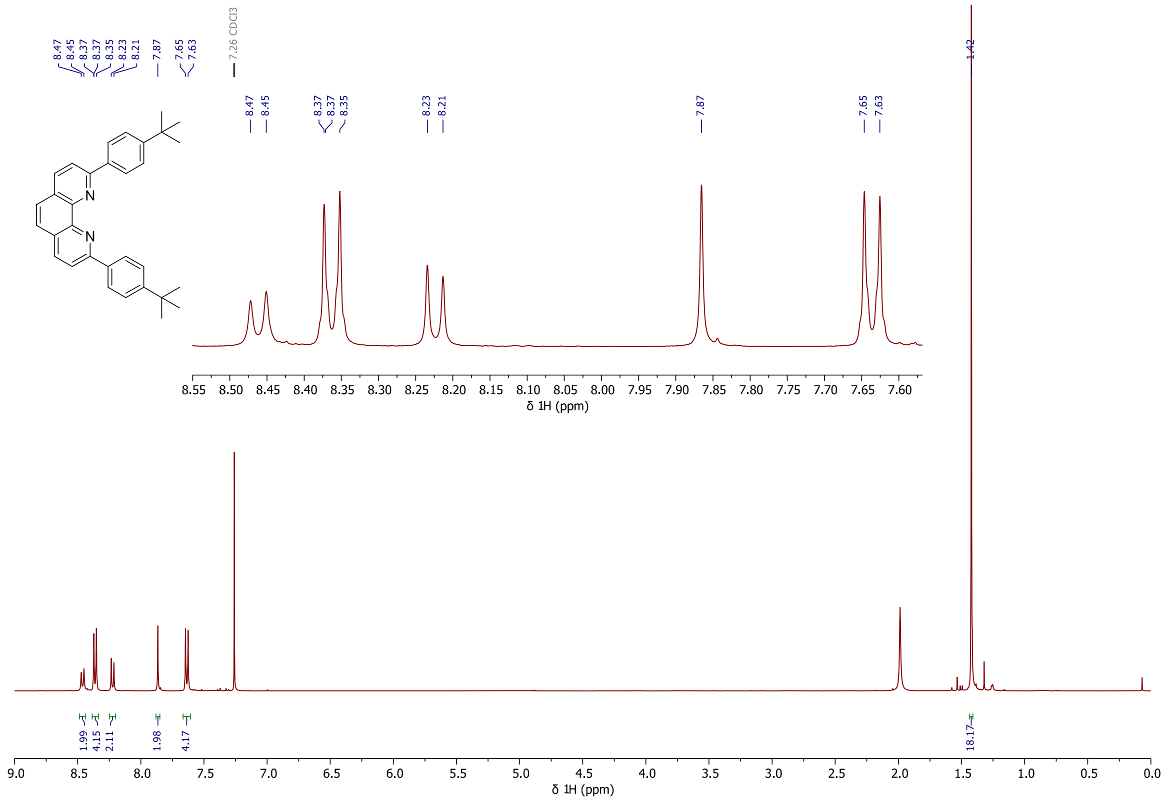

Figure S38. **^{1}H NMR** (400 MHz, $CDCl_3$, 298K) of **bis(4-(tert-butyl)phenyl)-1,10-phenanthroline**.

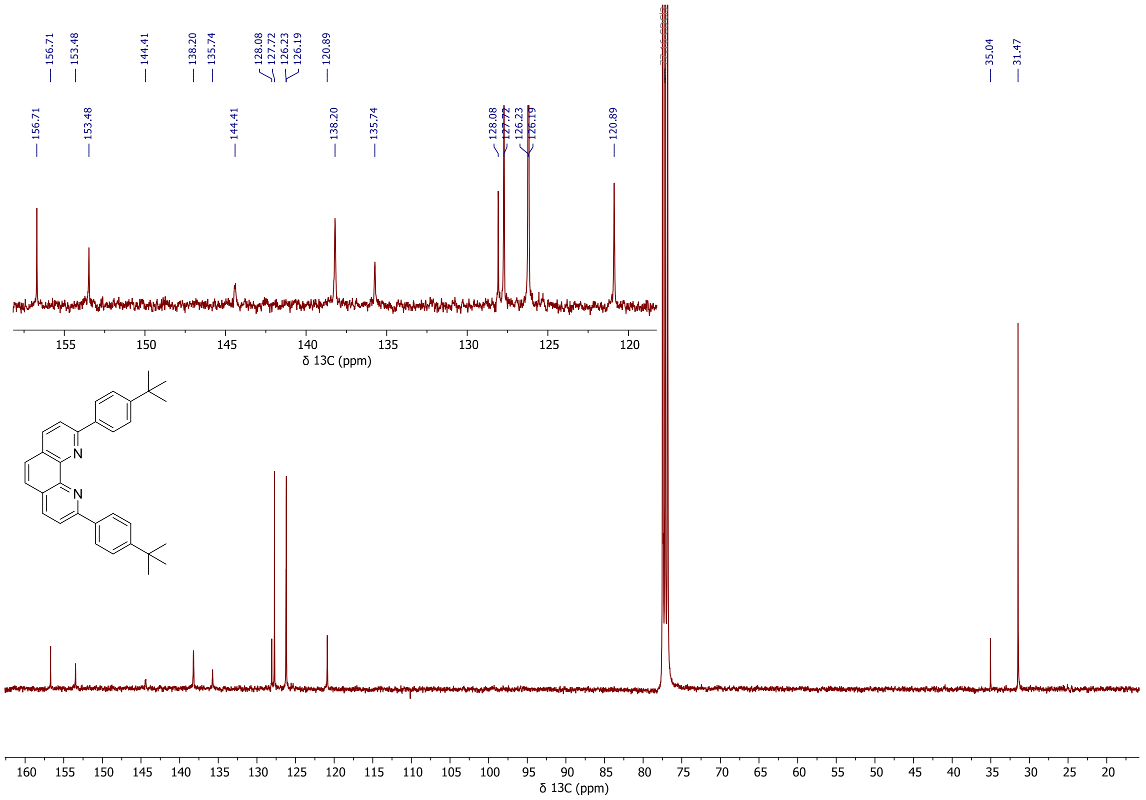

Figure S39. ^{13}C {^{1}H} **NMR** (100 MHz, $CDCl_3$, 298K) of **bis(4-(tert-butyl)phenyl)-1,10-phenanthroline**.

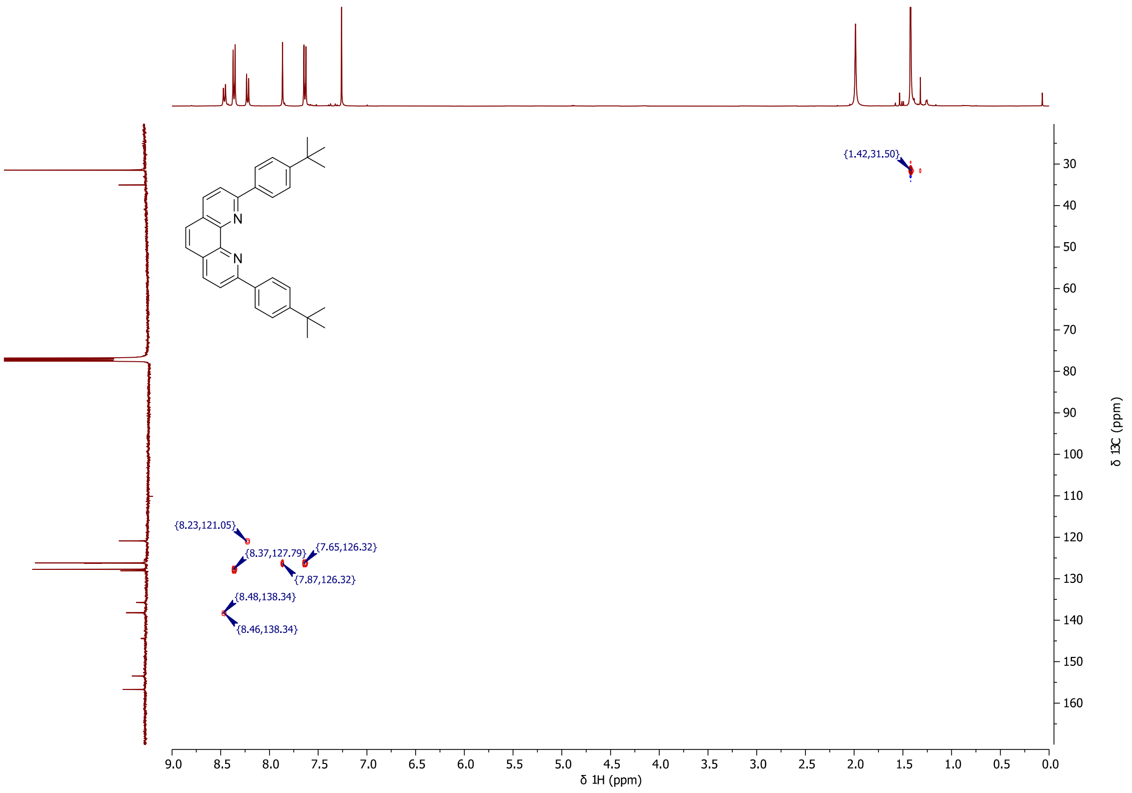

Figure S40. ^{1}H/^{13}C **HSQC** (400/100 MHz, $CDCl_3$, 298K) of **bis(4-(tert-butyl)phenyl)-1,10-phenanthroline**.

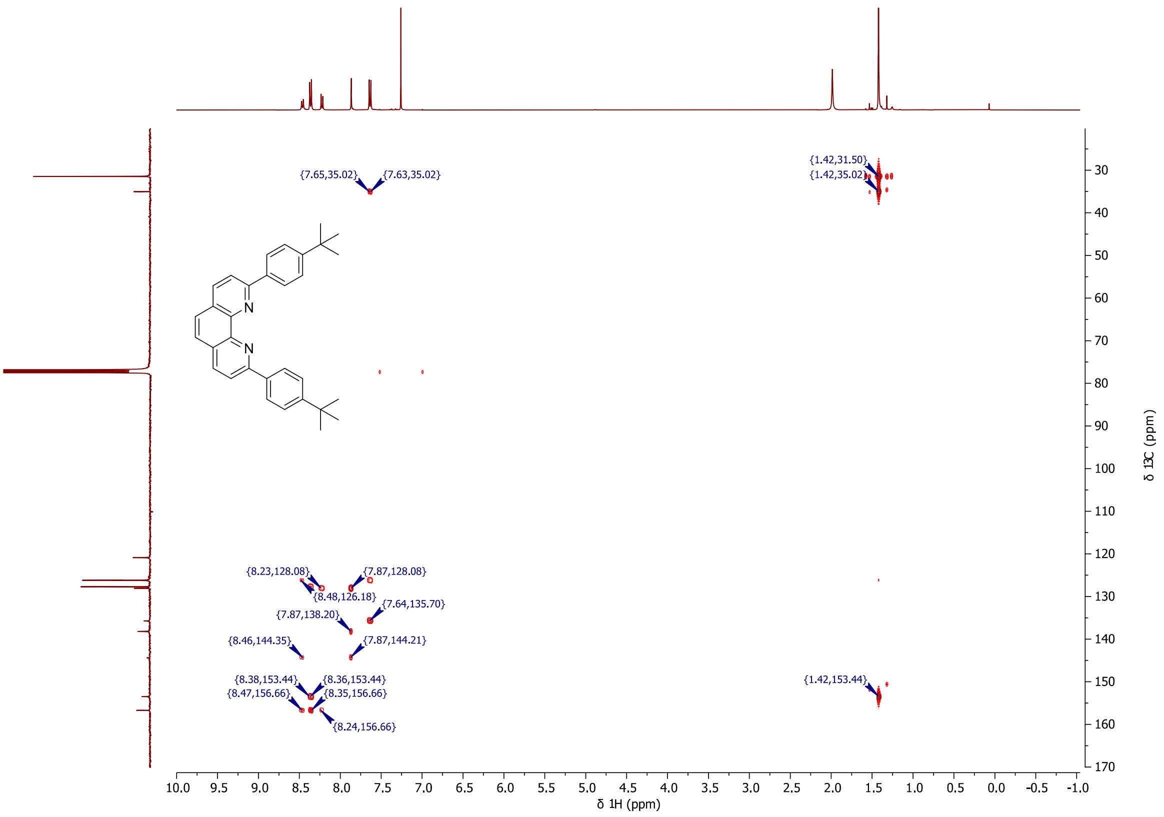

Figure S41. **^{1}H/^{13}C HMBC** (400/100 MHz, $CDCl_3$, 298K) of **bis(4-(tert-butyl)phenyl)-1,10-phenanthroline**.

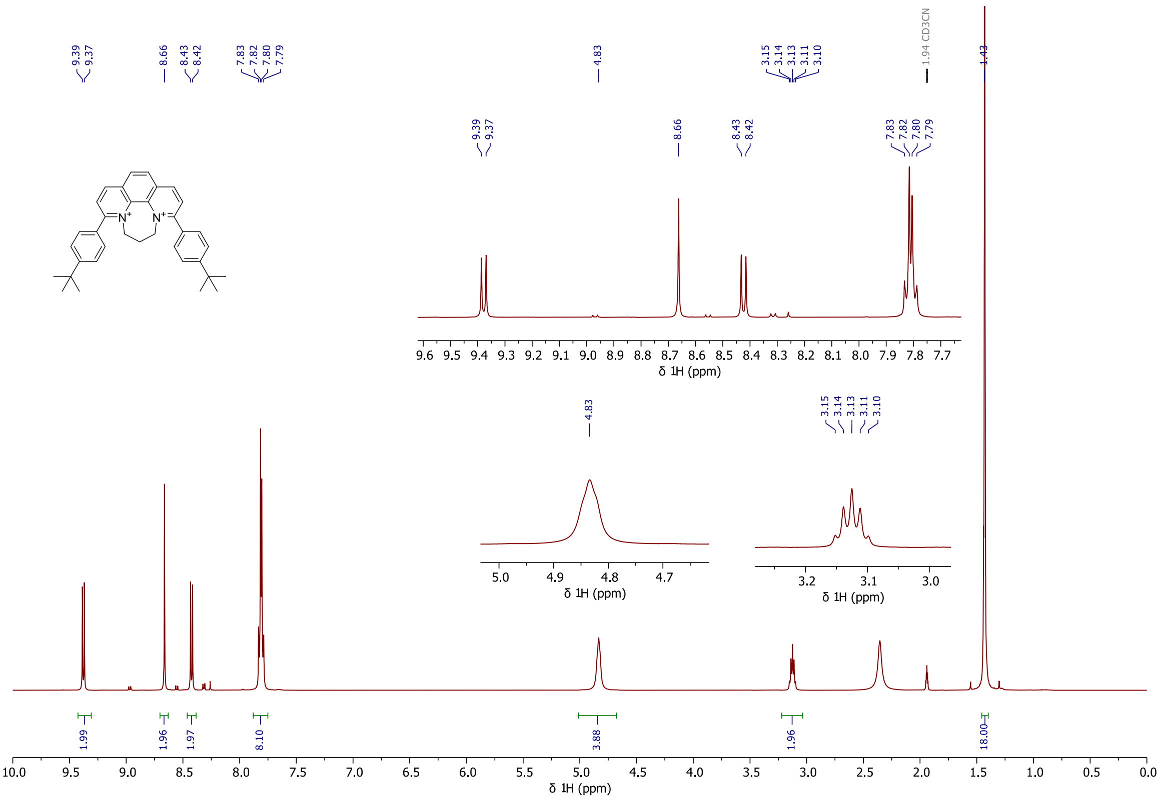

Figure S42. **^{1}H NMR** (500 MHz, CD_3CN, 298K) of **1c**.

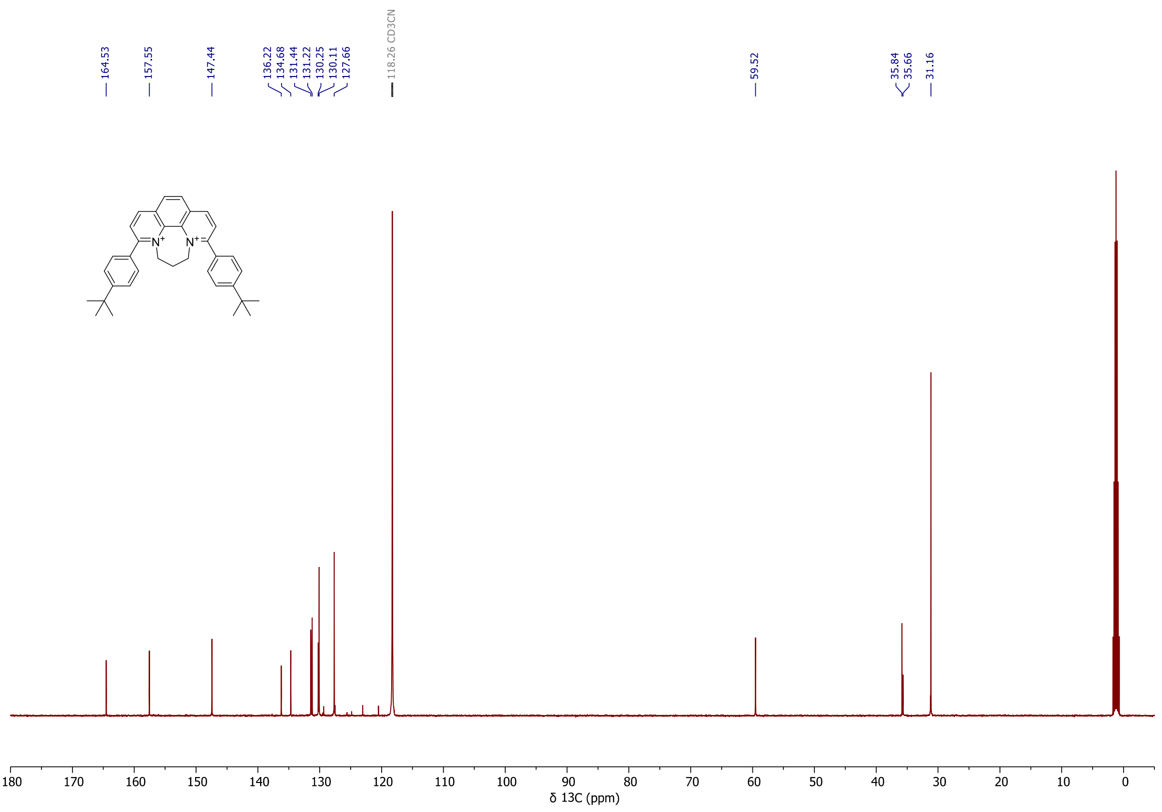

Figure S43. **^{13}C {^{1}H} NMR** (126 MHz, CD_3CN, 298K) of **1c**.

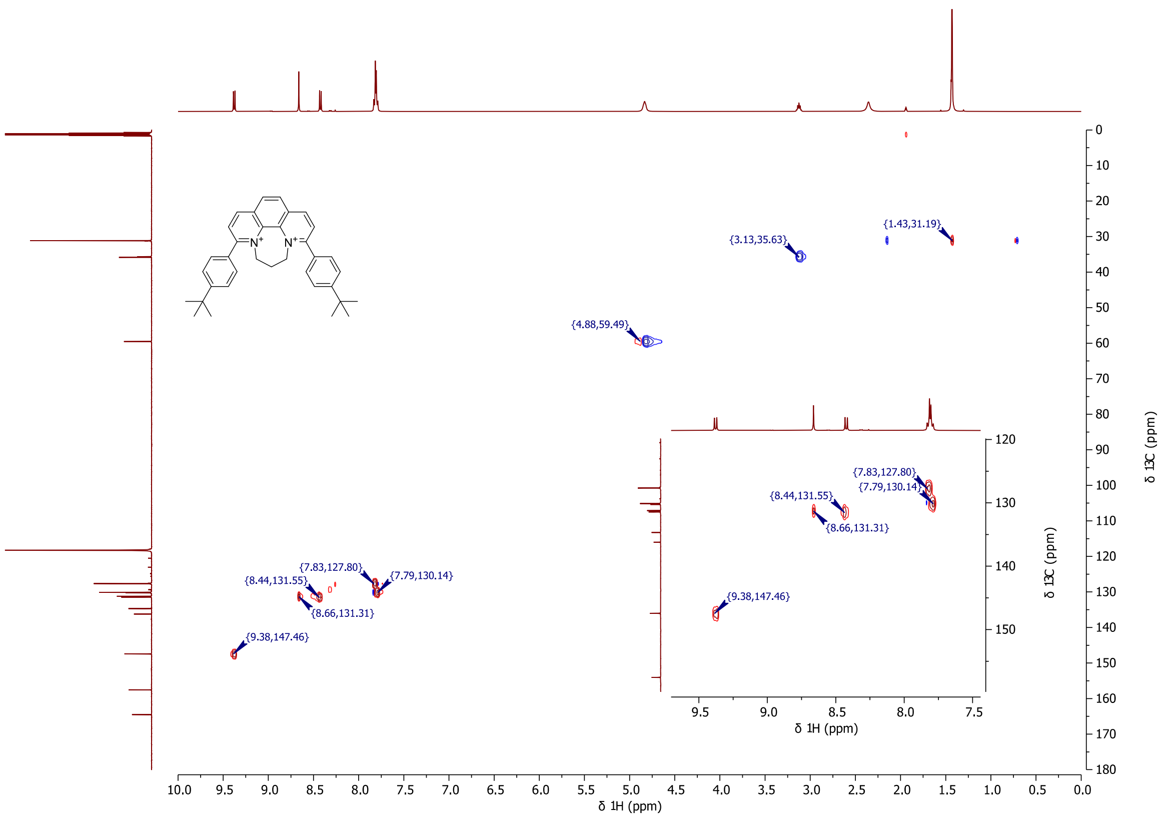

Figure S44. **$^{1}H/^{13}C$ HSQC** (500/126 MHz, CD_3CN, 298K) of **1c**.

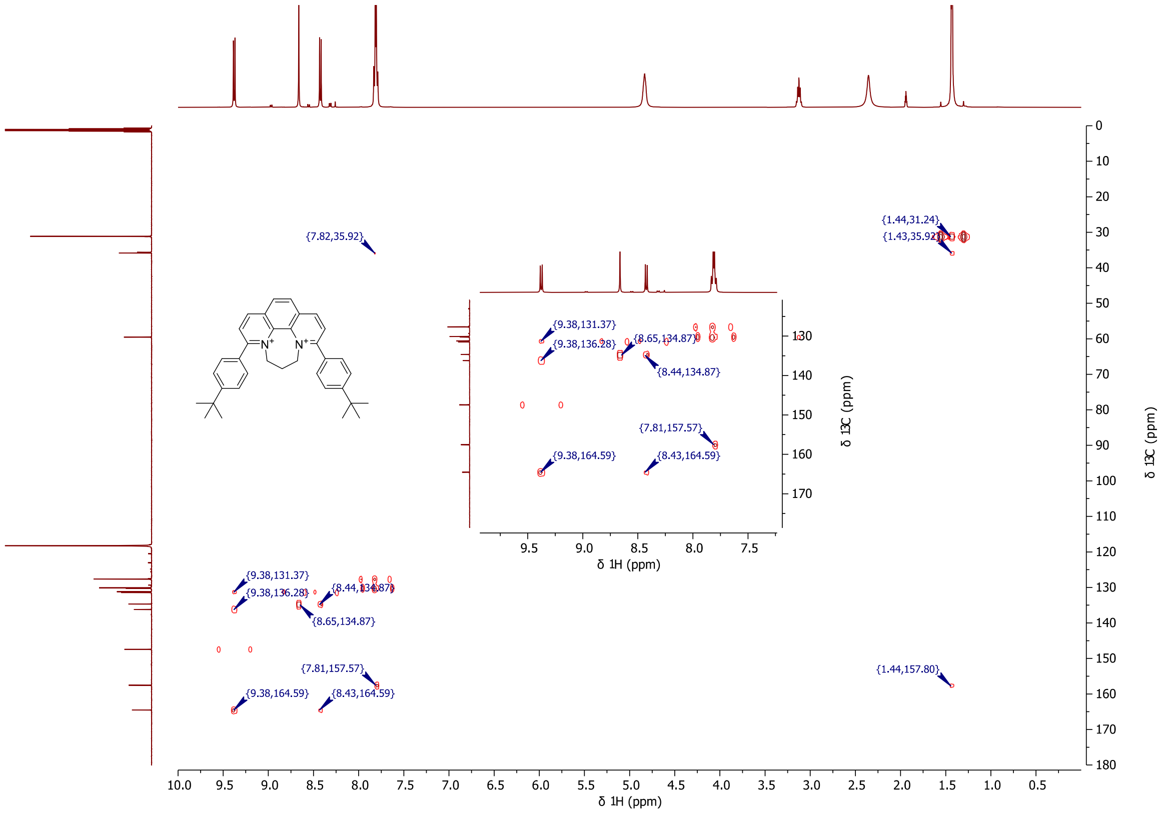

Figure S45. **^{1}H/^{13}C HMBC** (500/126 MHz, CD_3CN, 298K) of **1c**.

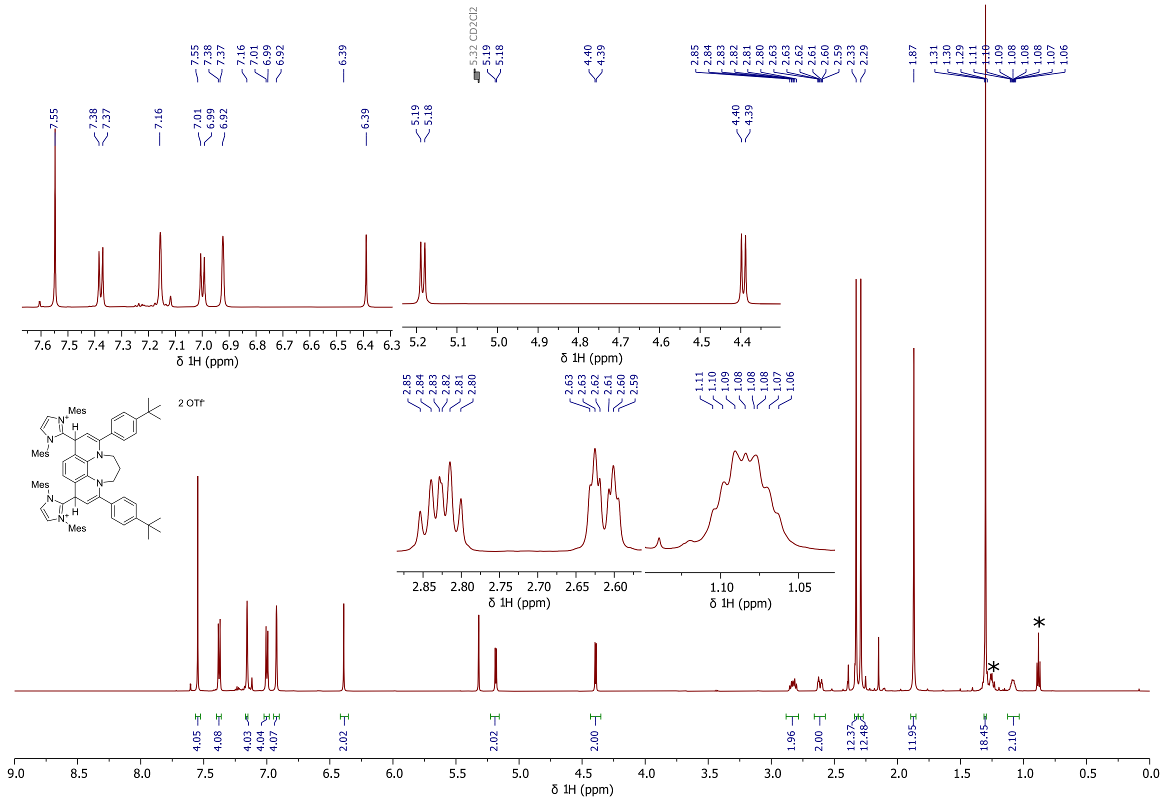

Figure S46. **^{1}H NMR** (600 MHz, CD_2Cl_2, 298K) of **1c^{Int}**. Pentane marked with an *.

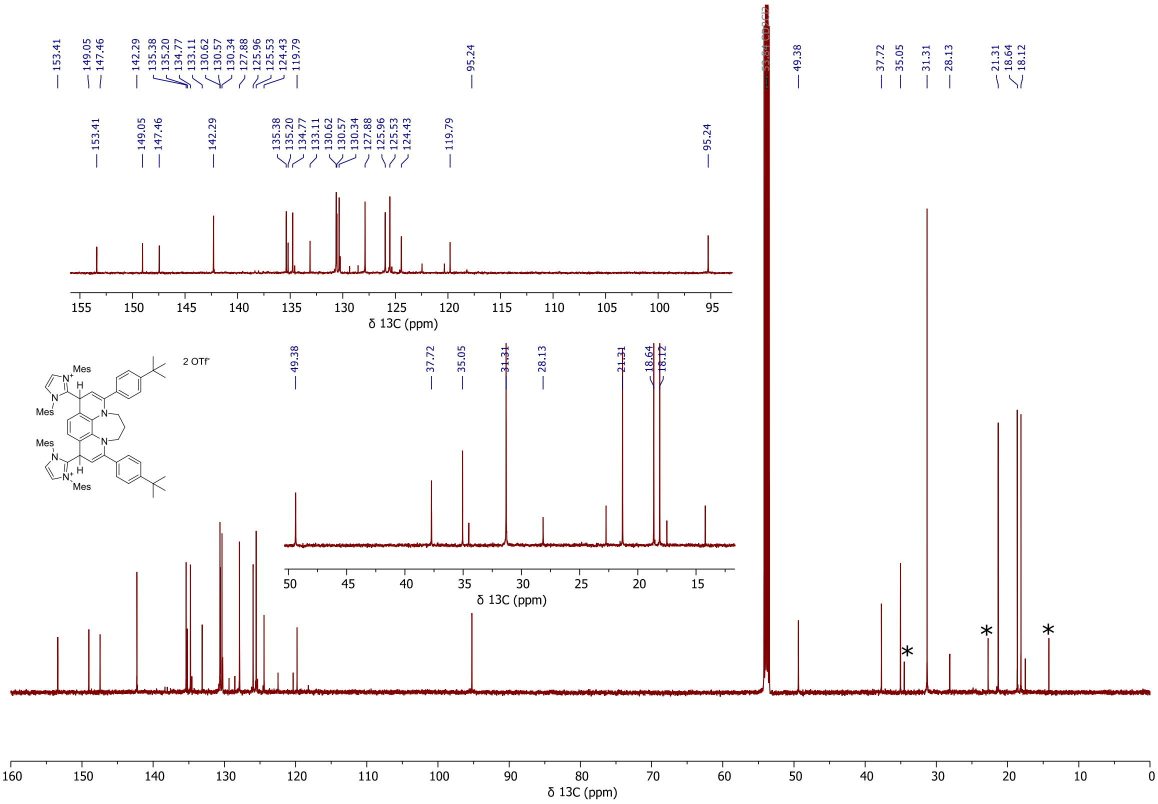

Figure S47. ^{13}**C NMR** (151 MHz, CD_2Cl_2, 298K) of **1c**^{Int}. Pentane marked with an *.

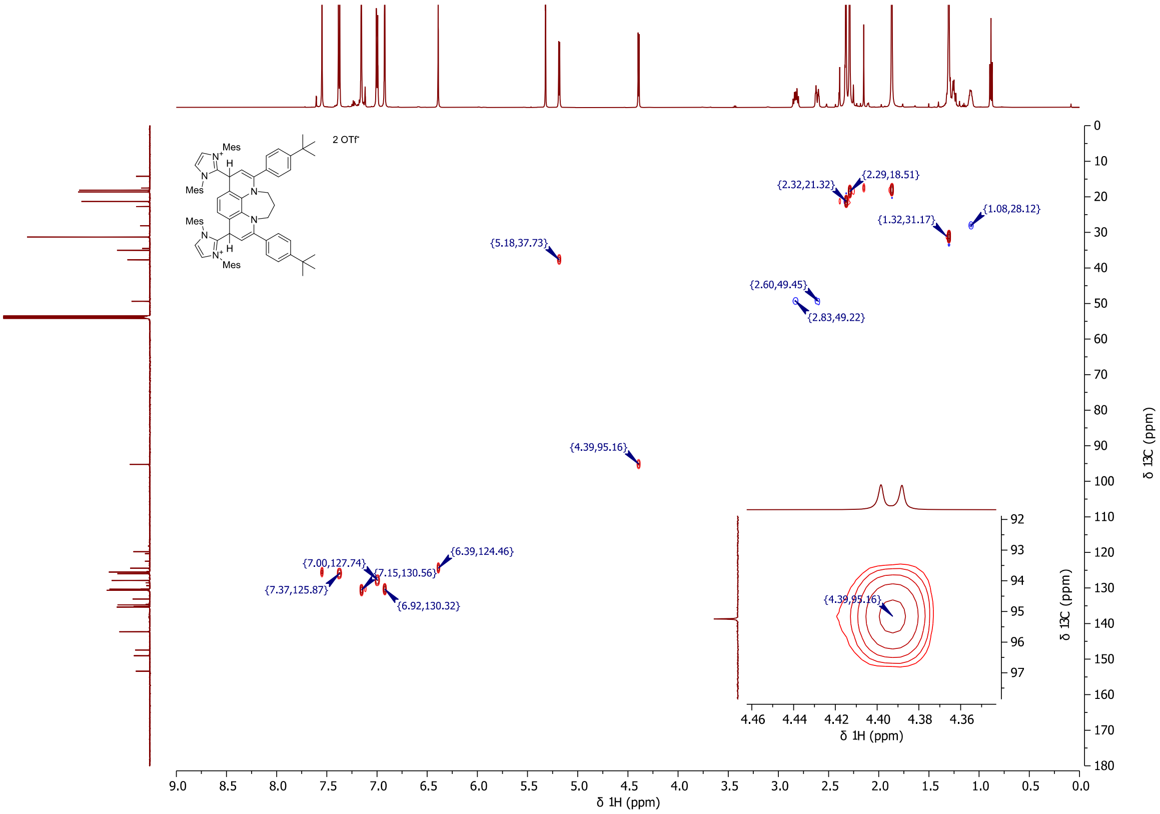

Figure S48. ^{1}**H/**^{13}**C HSQC** (600/151 MHz, CD_2Cl_2, 298K) of **1c**^{Int}.

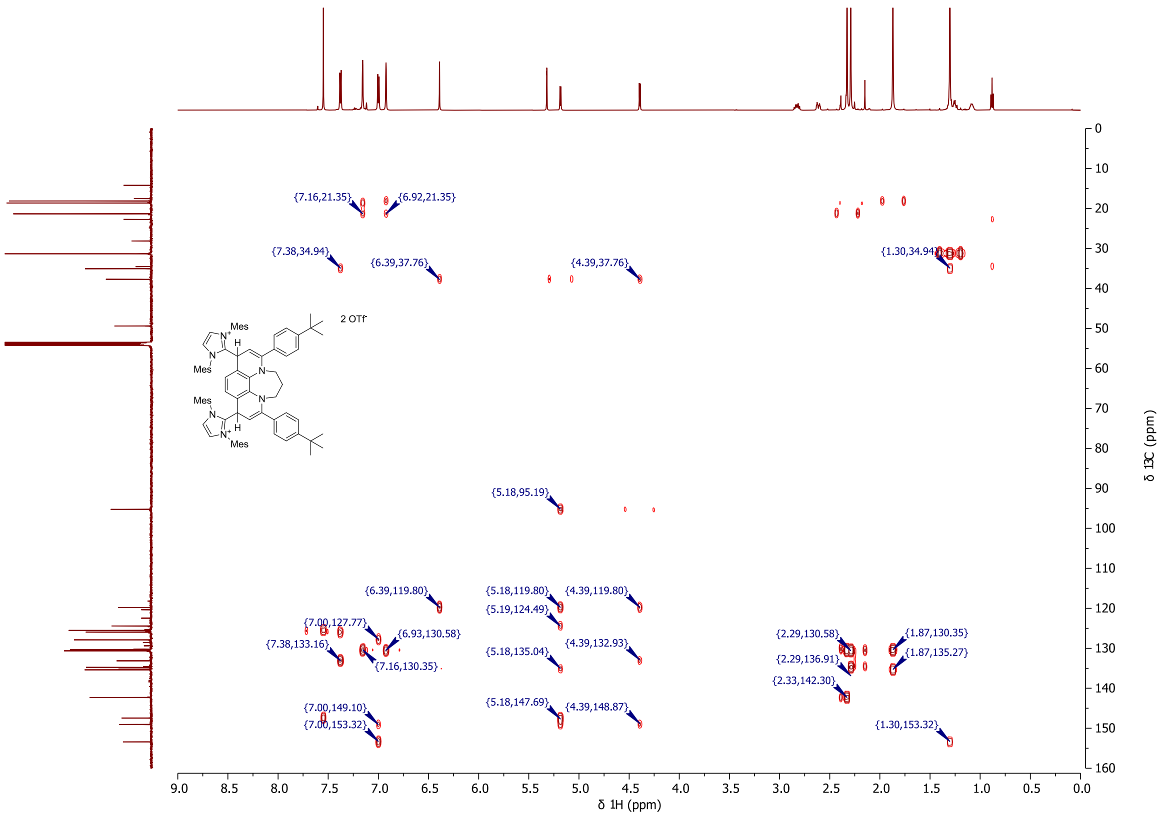

Figure S49. **$^{1}H/^{13}C$ HMBC** (600/151 MHz, CD_2Cl_2, 298K) of **$1c^{Int}$**.

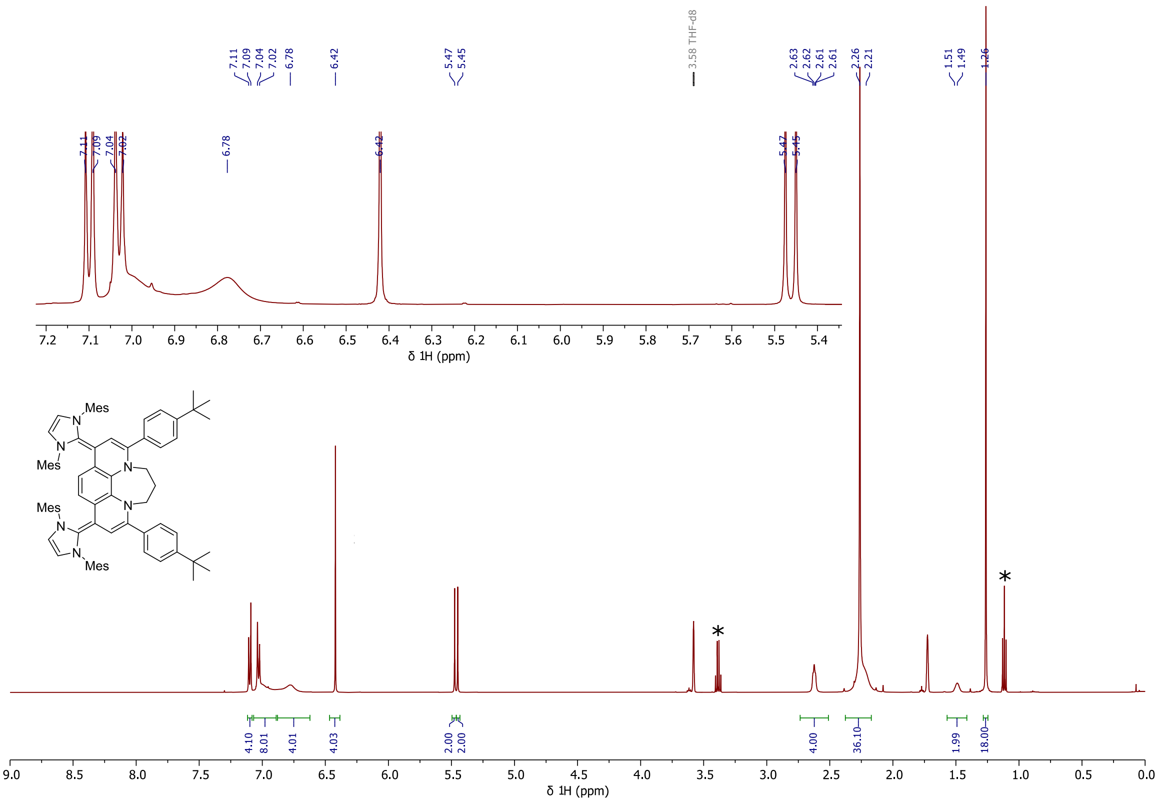

Figure S50. **^{1}H NMR** (500 MHz, d_8-thf, 298K) of **2c**. Et_2O marked with an *, after the addition of <1mg of KHMDS**.**

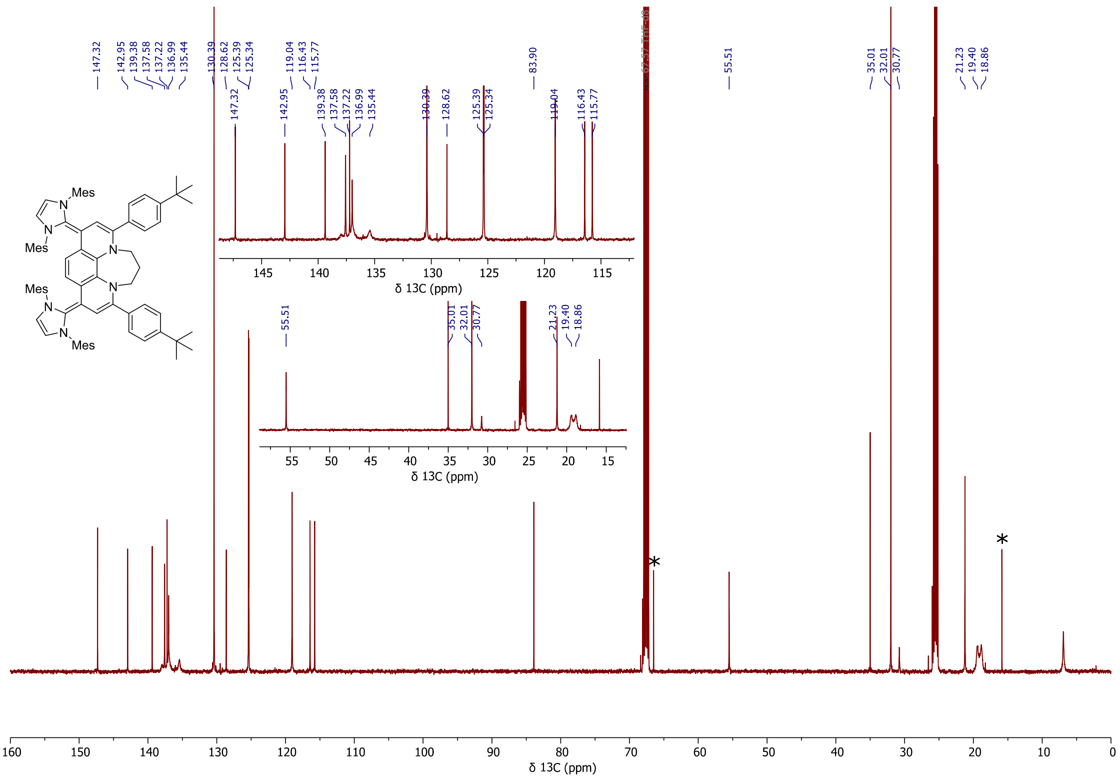

Figure S51. **^{13}C NMR** (125 MHz, d_8-thf, 298K) of **2c**. Et_2O marked with an *, after the addition of <1mg of KHMDS**.**

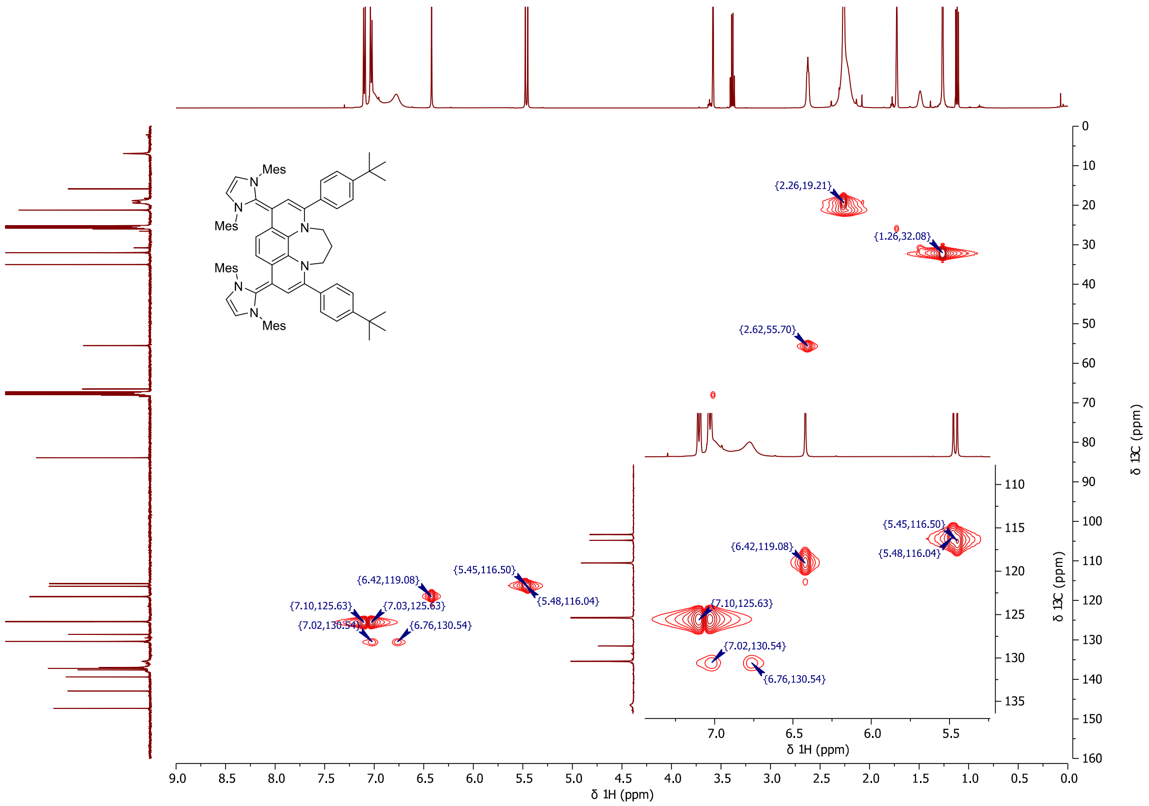

Figure S52. **^{1}H/^{13}C HSQC** (500/125 MHz, d_8-thf, 298K) of **2c** after the addition of <1mg of KHMDS**.**

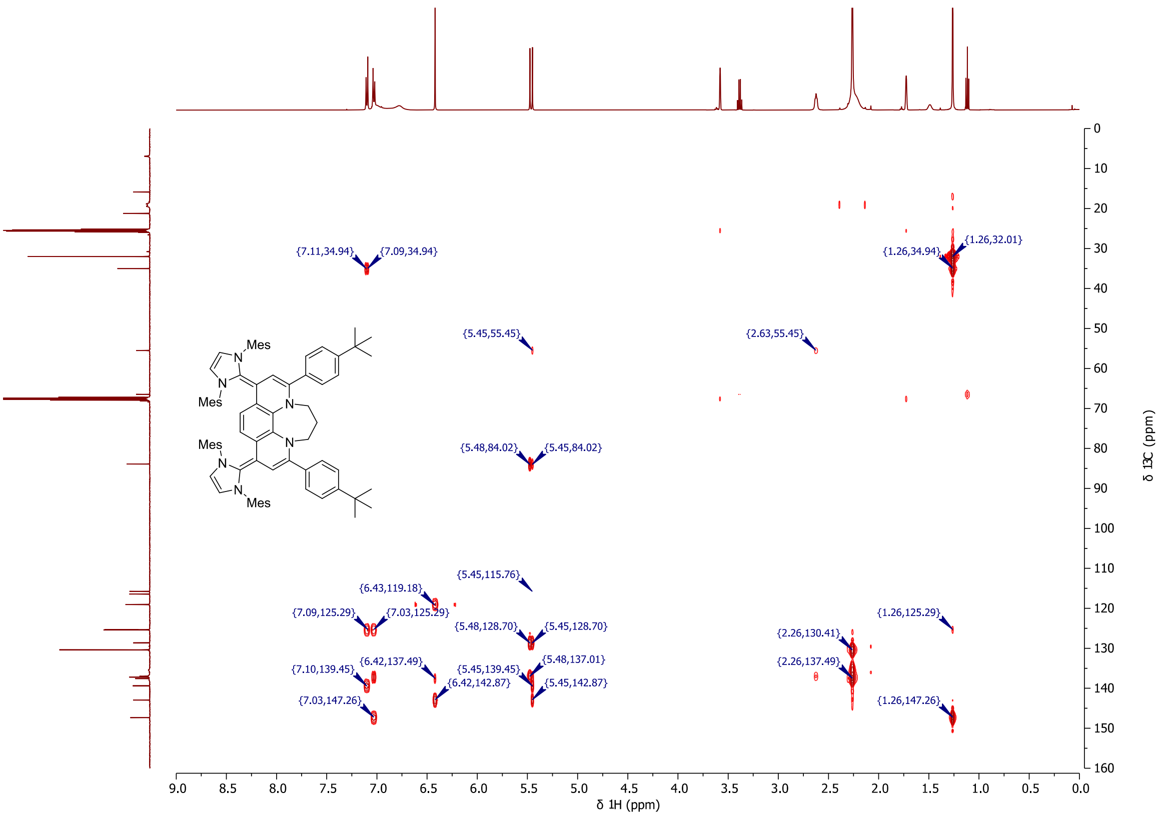

Figure S53. **^{1}H/^{13}C HMBC** (500/125 MHz, d_8-thf, 298K) of **2c** after the addition of <1mg of KHMDS.

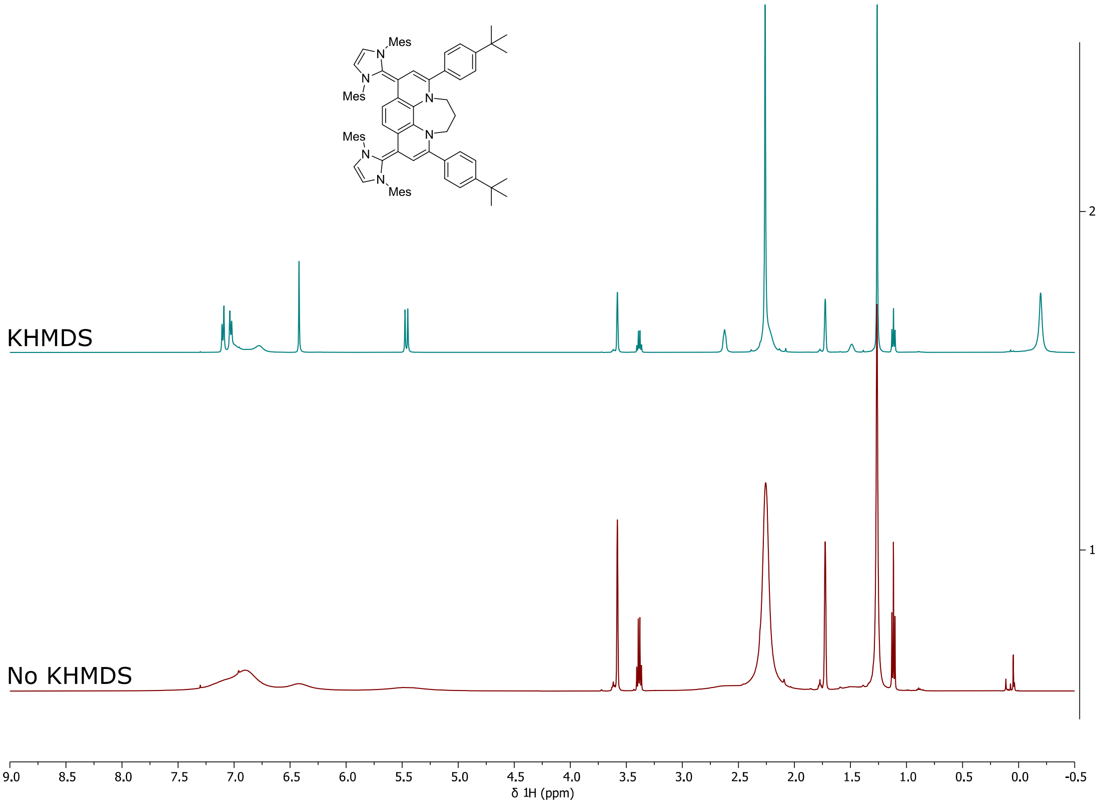

Figure S54. Comparison of **^{1}H NMR** (400 MHz, d_8-thf, 298 K) of **2c** before (bottom) after (top) the addition of <1mg of KHMDS**.**

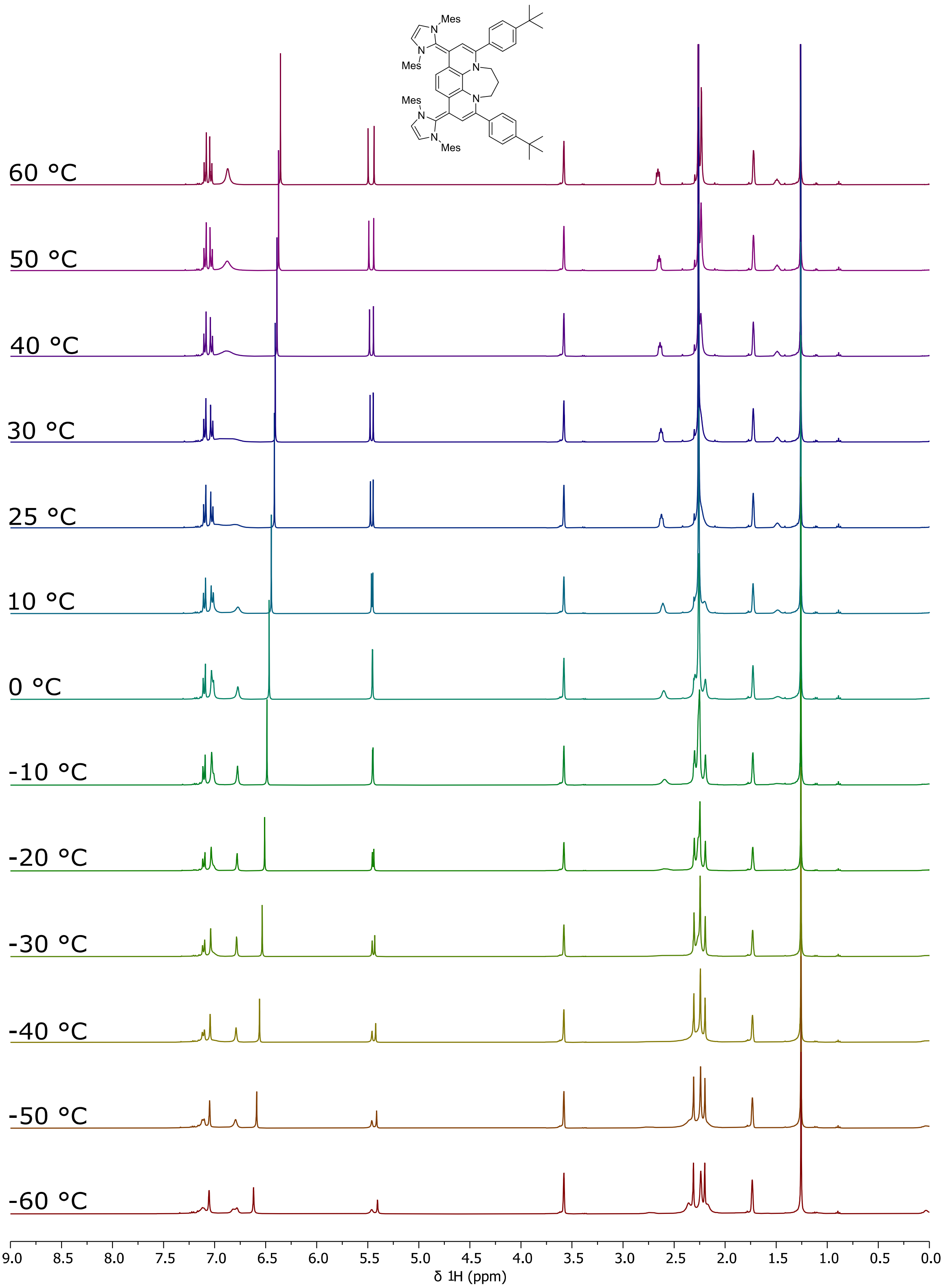

Figure S55. **^{1}H NMR** (400 MHz, d_8-thf, variable temperature) of **2c** after the addition of <1 mg of KHMDS**.**

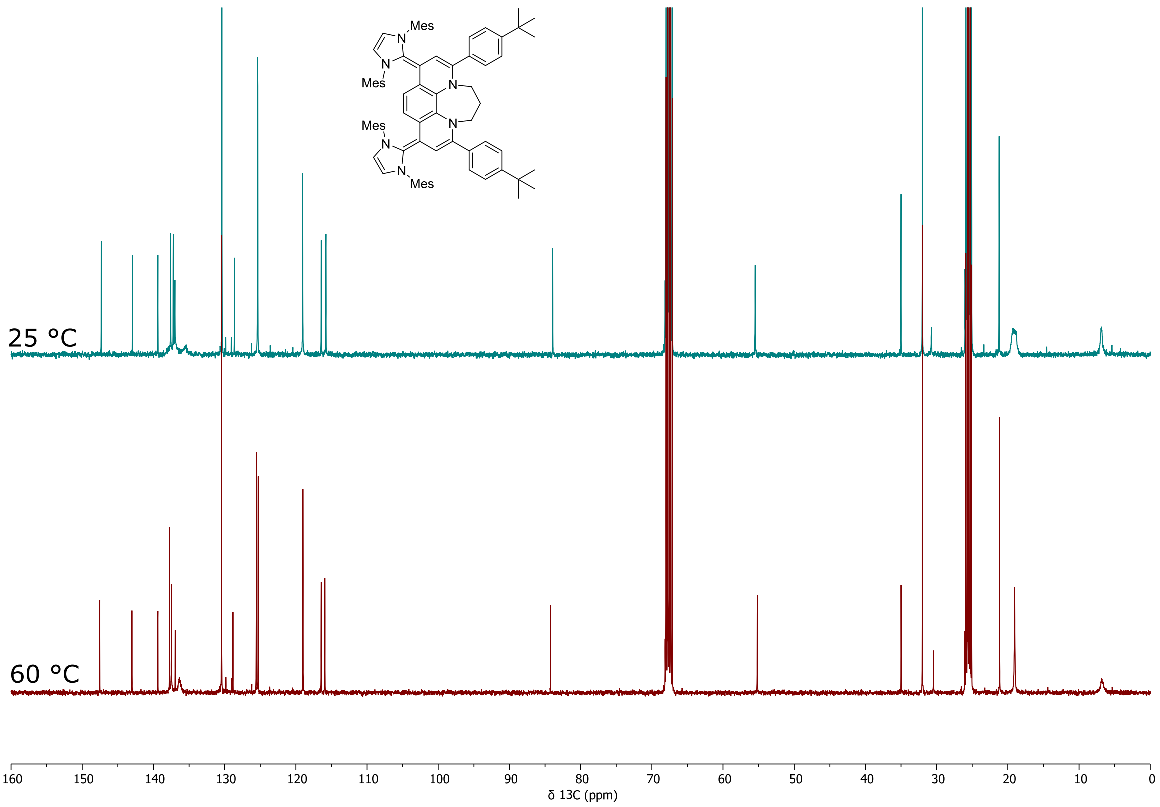

Figure S56. Comparison of **^{13}C NMR** (125 MHz, d_8-thf) of **2c** 25 °C and 60 °C. After the addition of <1 mg of KHMDS**.**

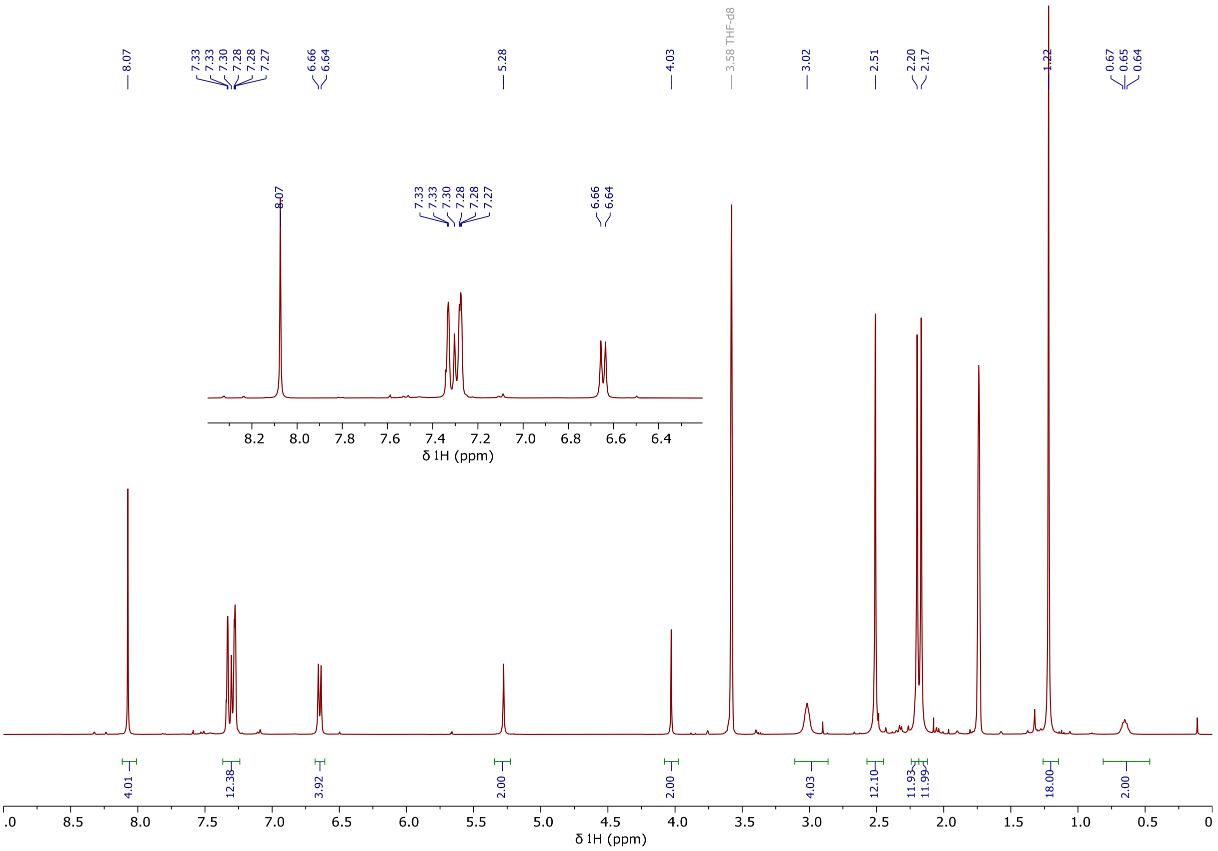

Figure S57. **^{1}H NMR** (400 MHz, d_8-thf, 225 K) of **$2c^{2+}$**.

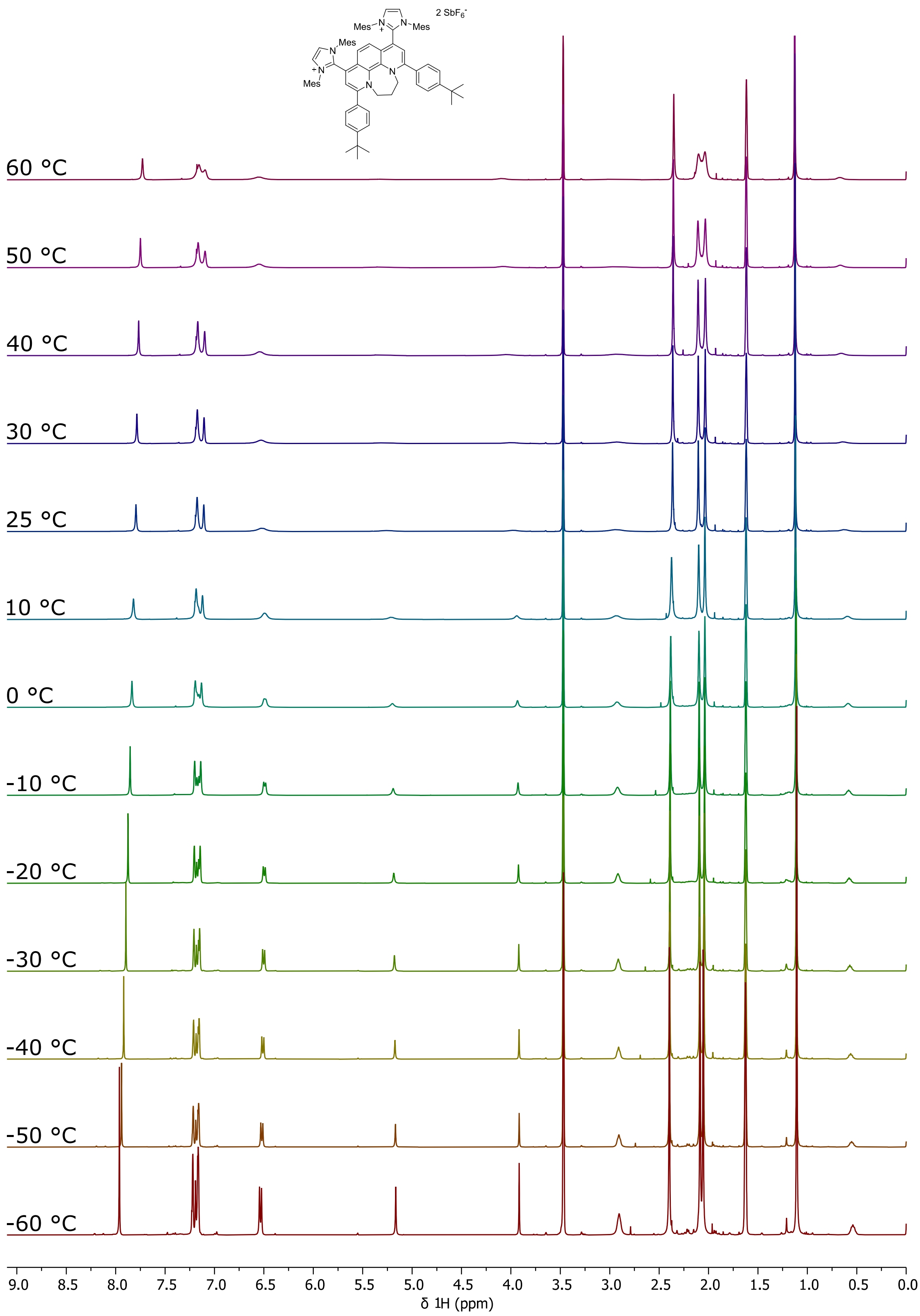

Figure S58. **^{1}H NMR** (400 MHz, d_8-thf, variable temperature) of **$2c^{2+}$**.

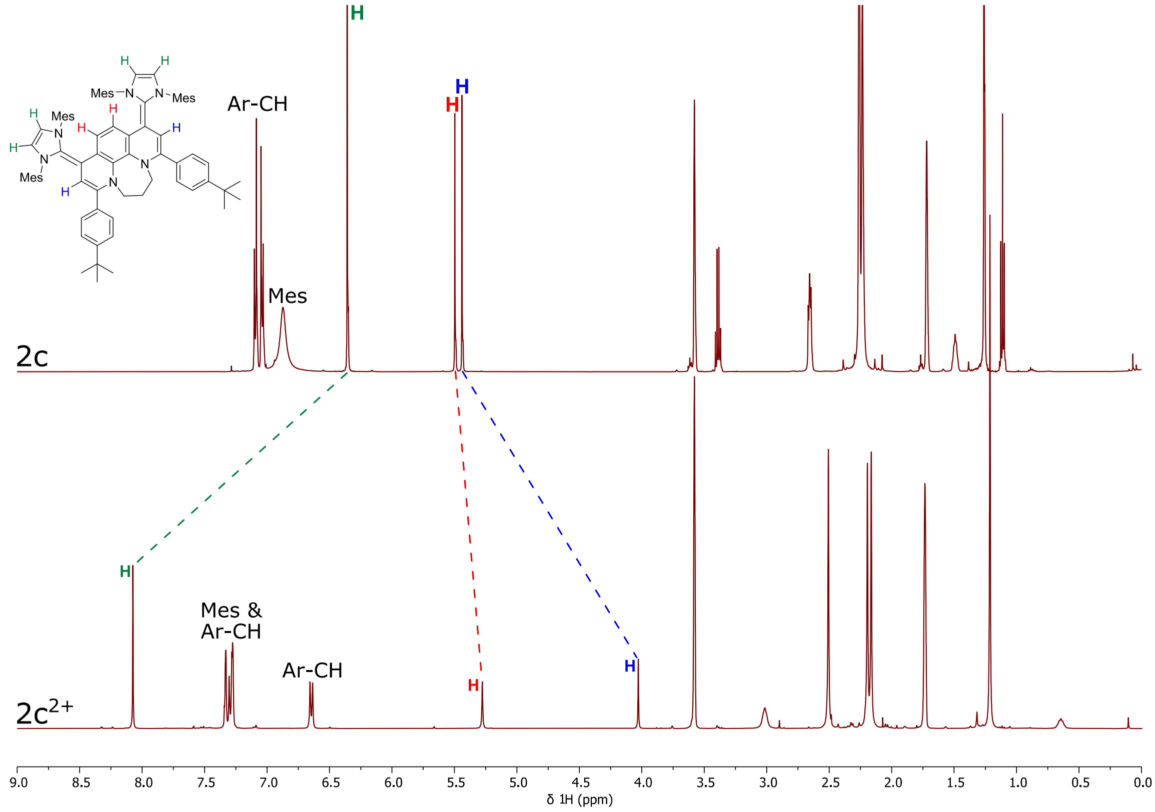

Figure S59. Comparison of ^{1}**H NMR** (400 MHz, d_8-thf, 225 K) of **2c** and **2c^{2+}**.

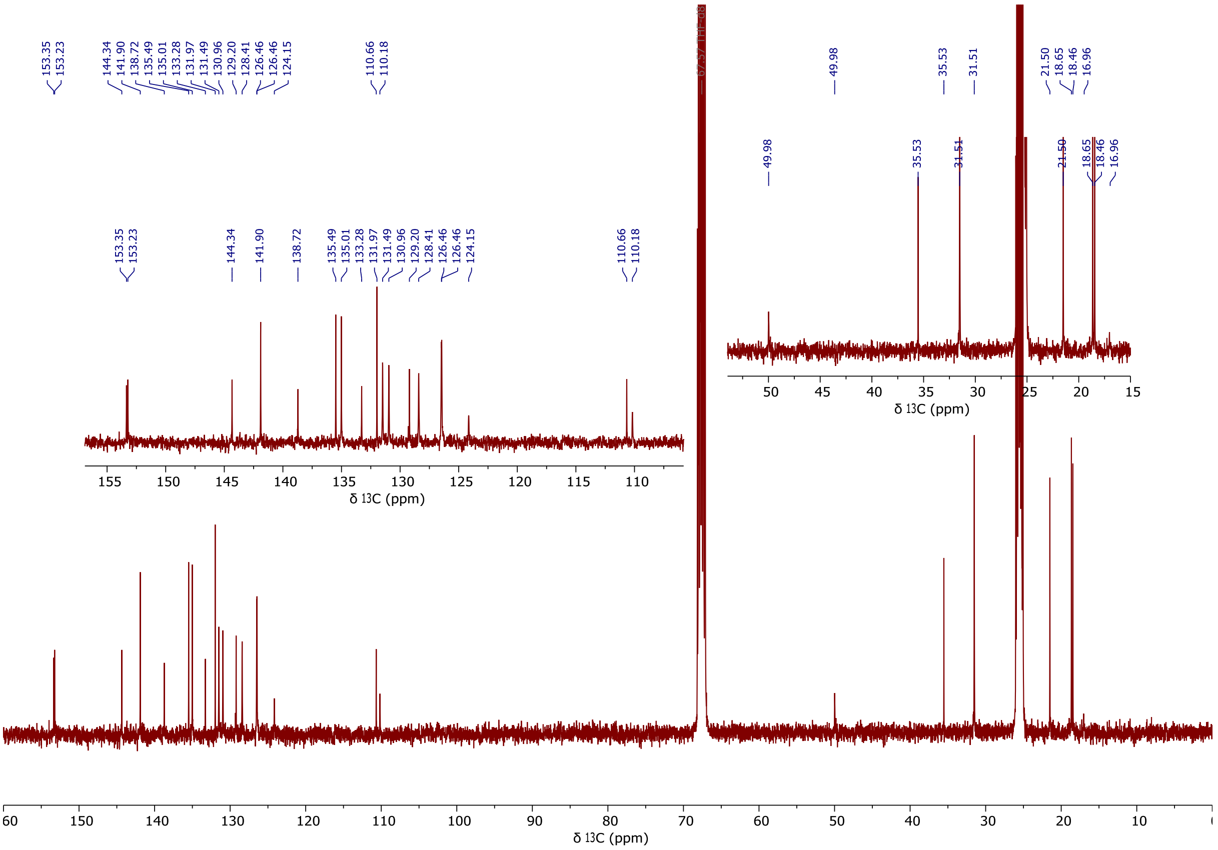

Figure S60. ^{13}**C NMR** (101 MHz, d_8-thf, 225 K) of **2c^{2+}**.

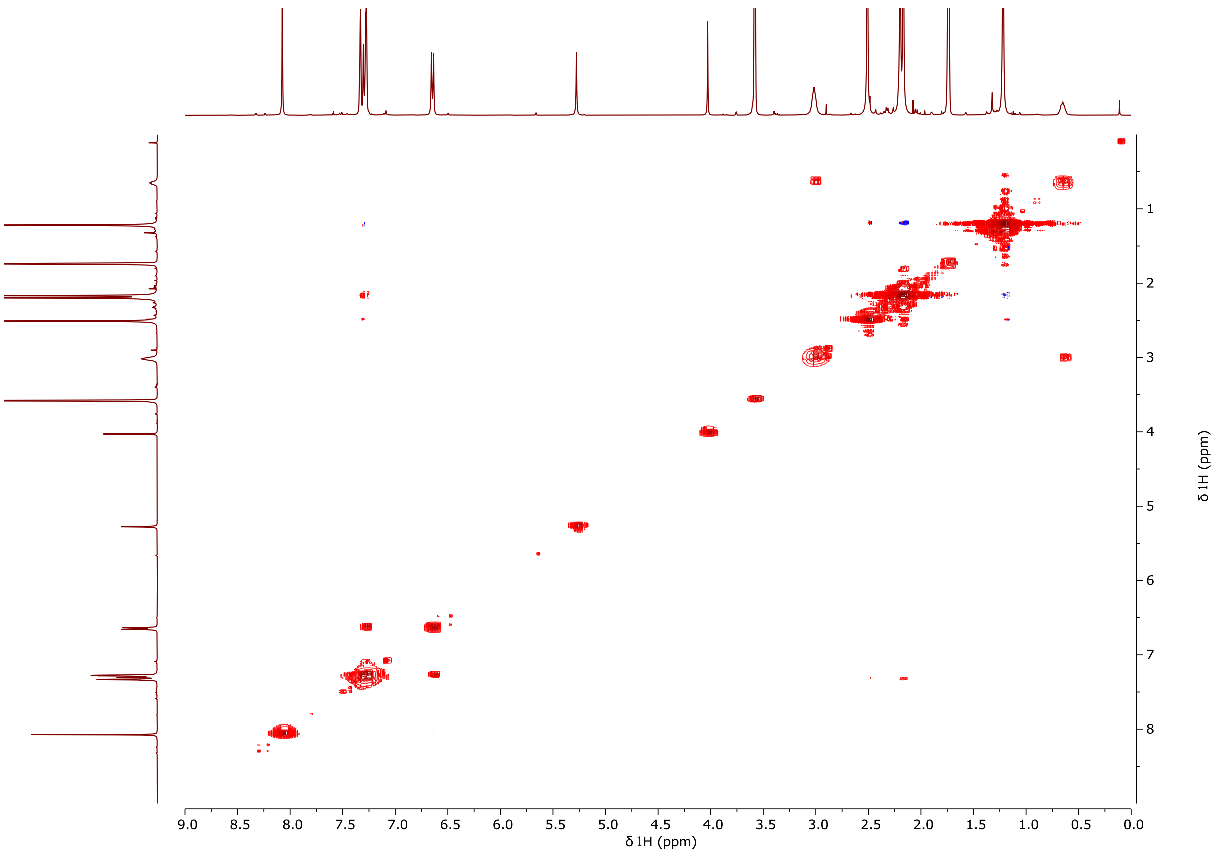

Figure S61. **$^1H/^1H$ COSY** (400/400 MHz, d_8-thf, 225 K) of **$2c^{2+}$**.

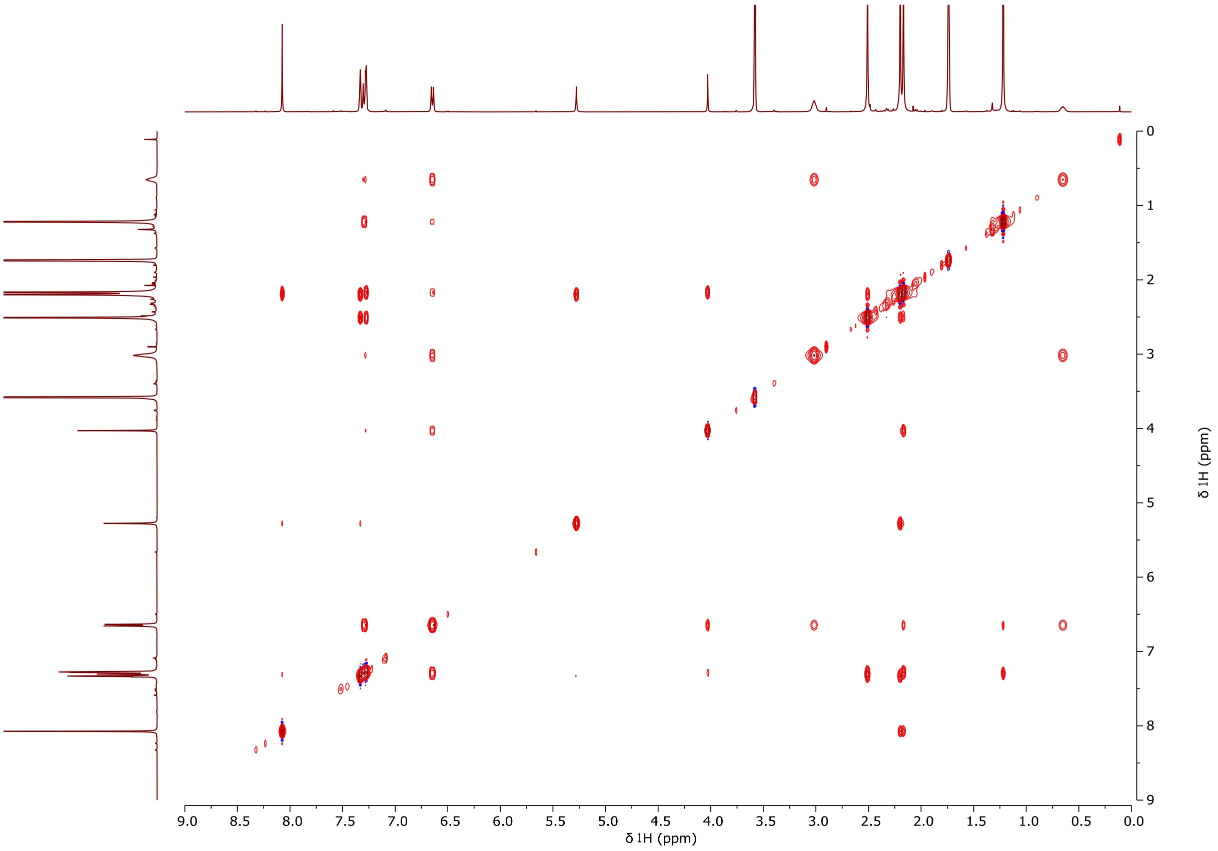

Figure S62. **$^1H/^1H$ NOESY** (400/400 MHz, d_8-thf, 225 K) of **$2c^{2+}$**.

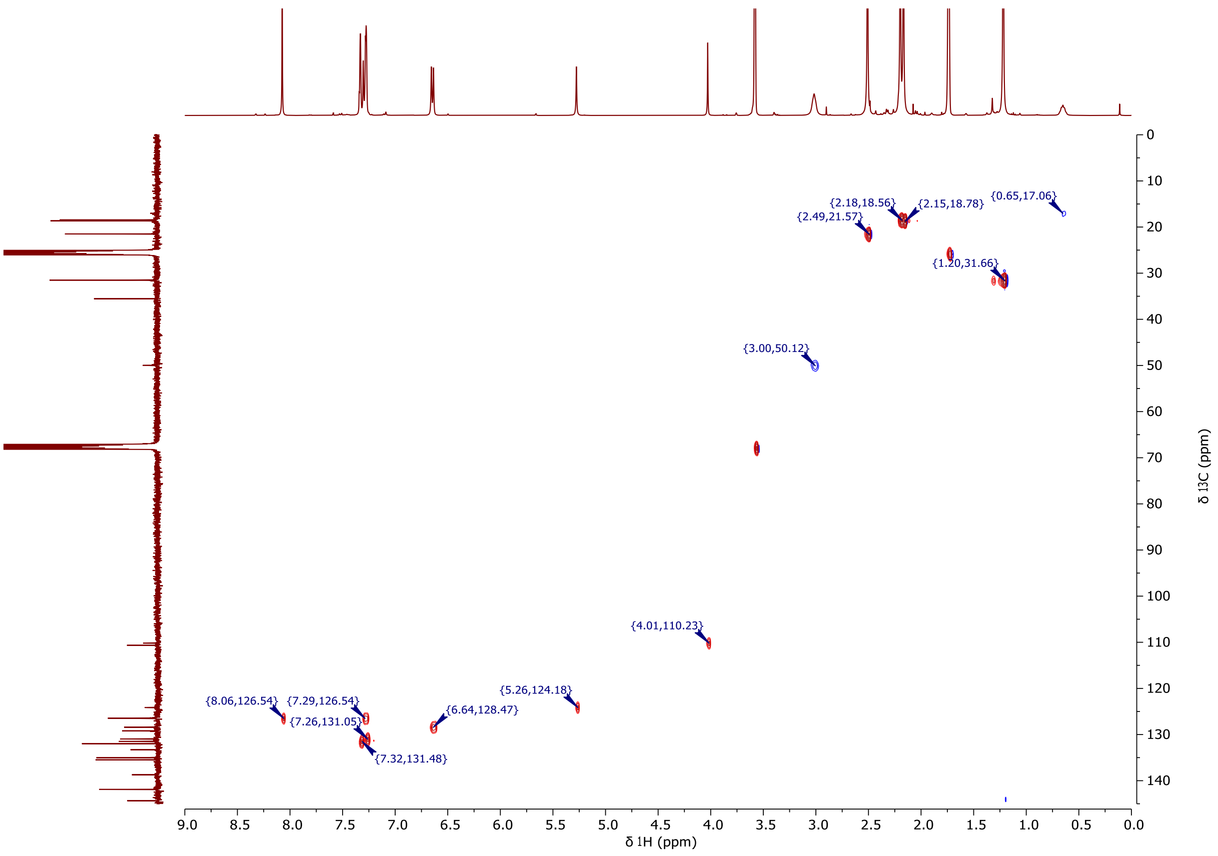

Figure S63. **^{1}H/^{13}C HSQC** (400/101 MHz, d_8-thf, 225 K) of **2c^{2+}**.

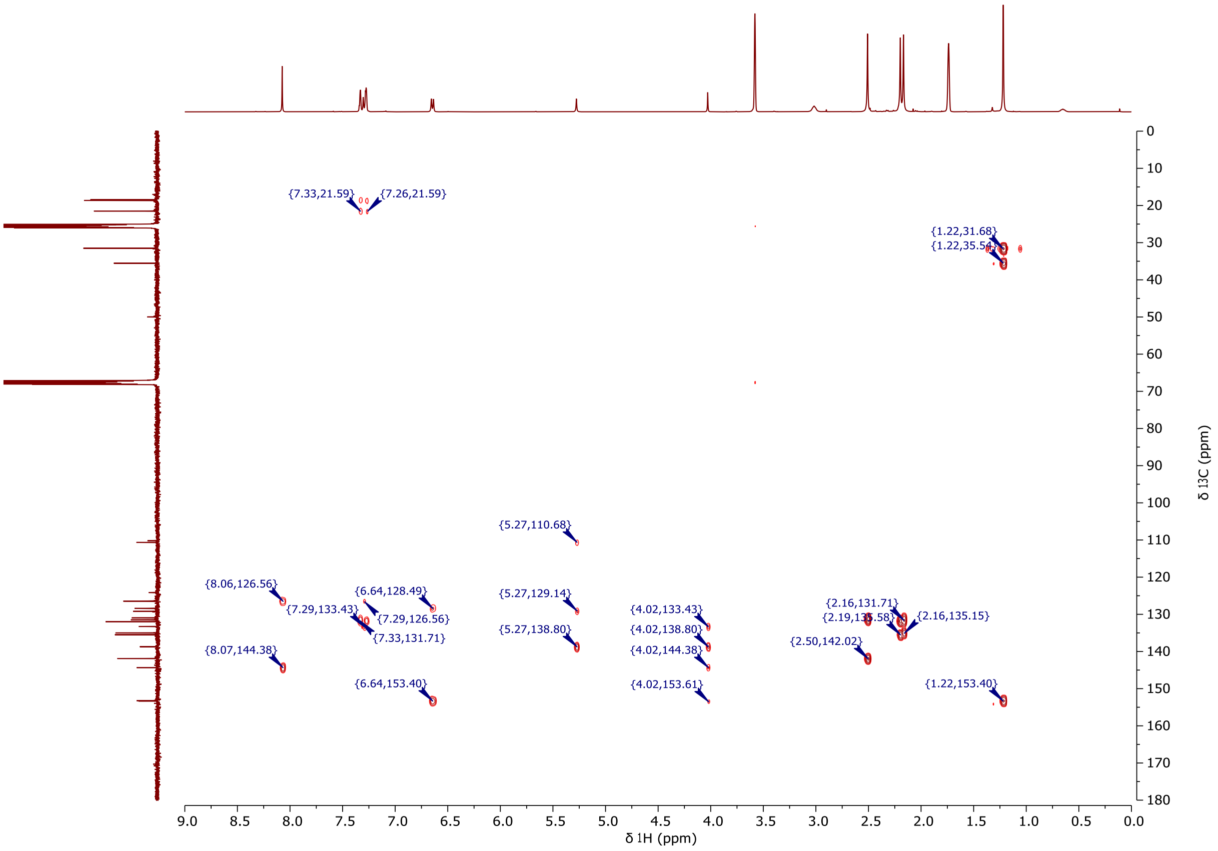

Figure S64. **^{1}H/^{13}C HMBC** (400/101 MHz, d_8-thf, 225 K) of **2c^{2+}**.

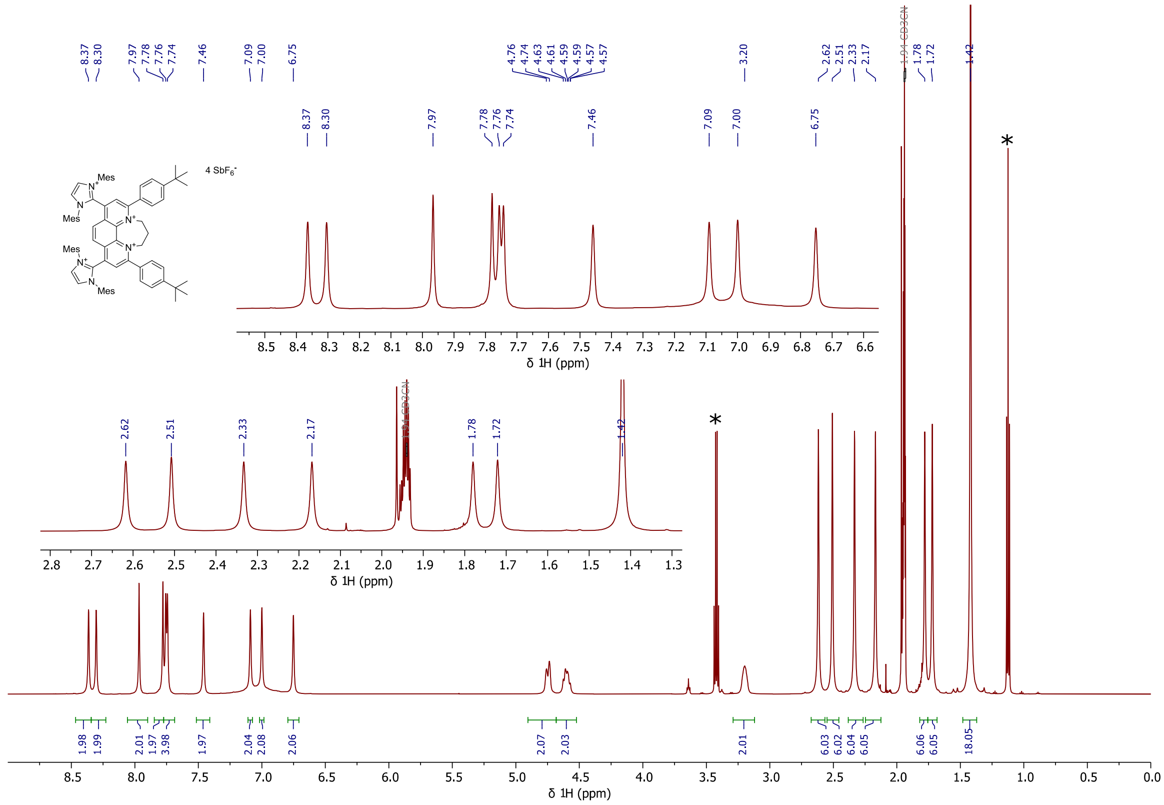

Figure S65. **^{1}H NMR** (600 MHz, CD_3CN, 298K) of **2c^{4+}**. Coordinated Et_2O marked with *.

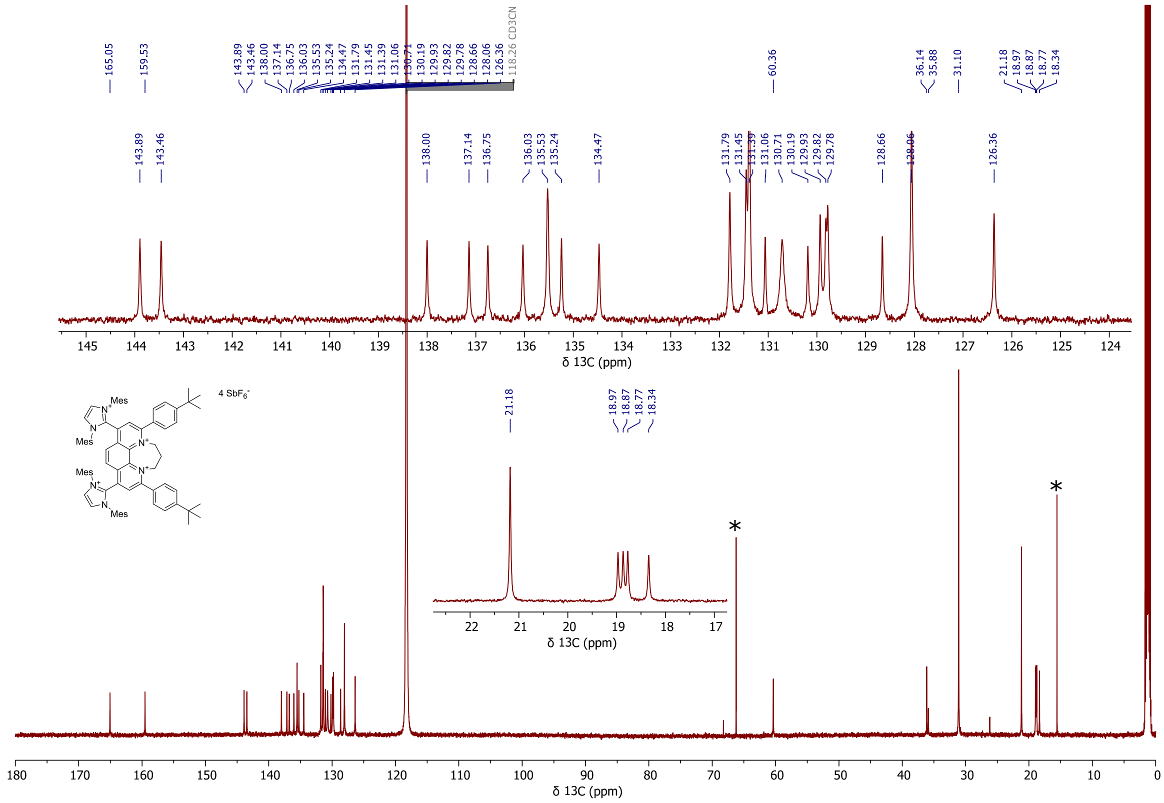

Figure S66. **^{13}C NMR** (150 MHz, CD_3CN, 298K) of **2c^{4+}**. Coordinated Et_2O marked with *.

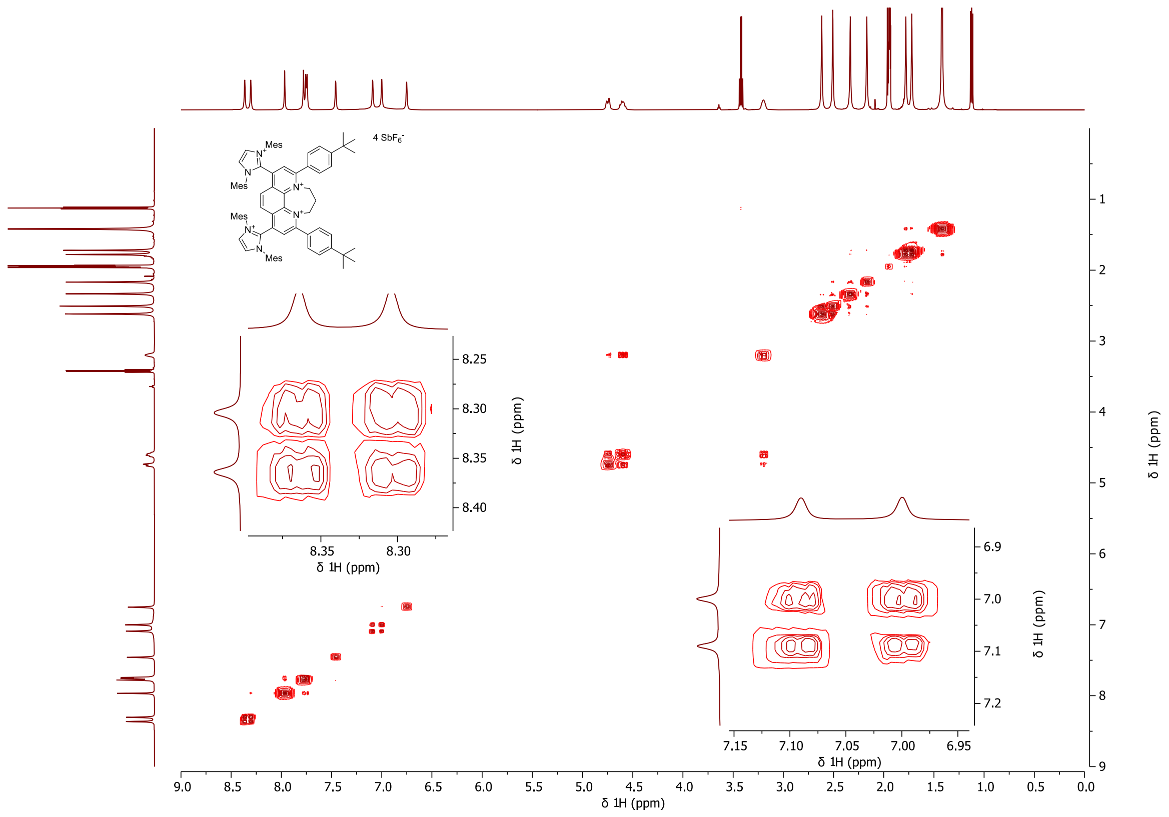

Figure S67. **^{1}H/^{1}H COSY** (600/600 MHz, CD_3CN, 298K, symmetrized) of **$2c^{4+}$**.

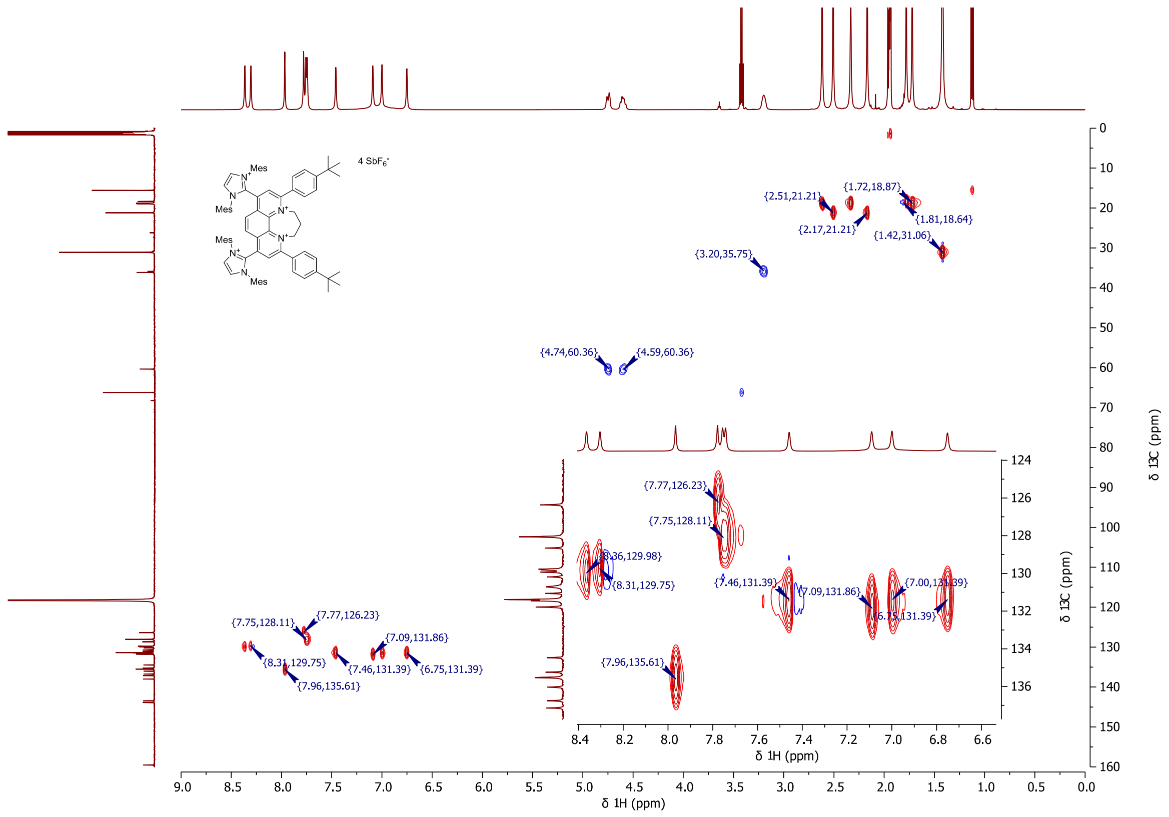

Figure S68. **^{1}H/^{13}C HSQC** (600/150 MHz, CD_3CN, 298K) of **$2c^{4+}$**.

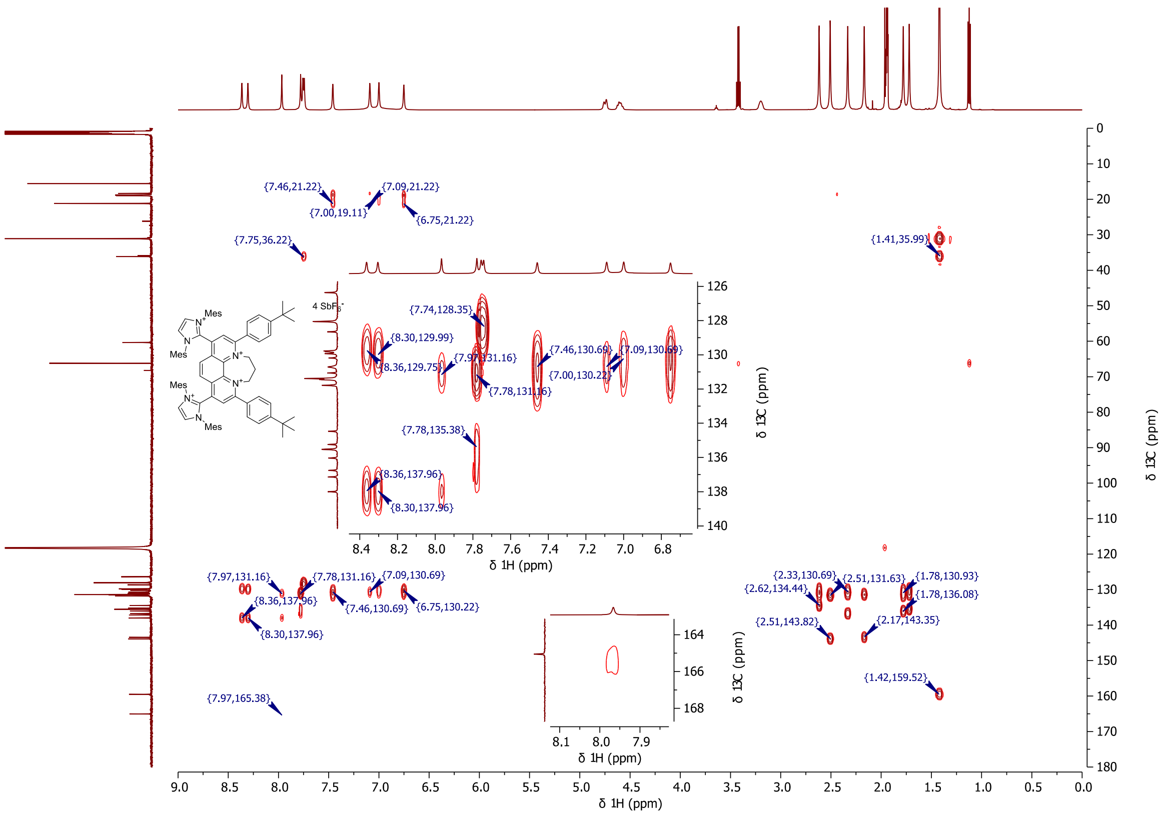

Figure S69. **^{1}H/^{13}C HMBC** (600/150 MHz, CD_3CN, 298K) of **$2c^{4+}$**.

4. Attempted preparation of radicals **2a**$^{+}$ and **2b**$^{+}$

CV measurements (see below) show only one two electron redox event for the formation of **2a**$^{2+}$ from **2a**, while SWV measurements indicate a small separation of the redox potentials for **2b** → **2b**$^{+}$ → **2b**$^{2+}$. We were curious, if this behavior is also reflected in the stoichiometric oxidation of **2a/b** with one equivalent of $AgSbF_6$.

<u>Attempted synthesis of **2a**$^{+}$</u>: The reaction was carried out in a J-Young NMR tube, utilizing CD_3CN as solvent. While **2a** is not soluble in MeCN (only residual solvents can be detected; see Fig. S70), the color changes immediately after addition of 1 eq. $AgSbF_6$ from light violet to dark blue and the formation of a dark precipitate (Ag^0) is observed. After filtration, the ^1H NMR spectrum clearly shows the formation of **2a**$^{2+}$ (Fig. S70), while ca. 50% of **2a** was filtered off. No EPR signal was detected. This finding agrees with the CV data that the preparation of **2a**$^{+}$ is not possible but leads to disproportionation.

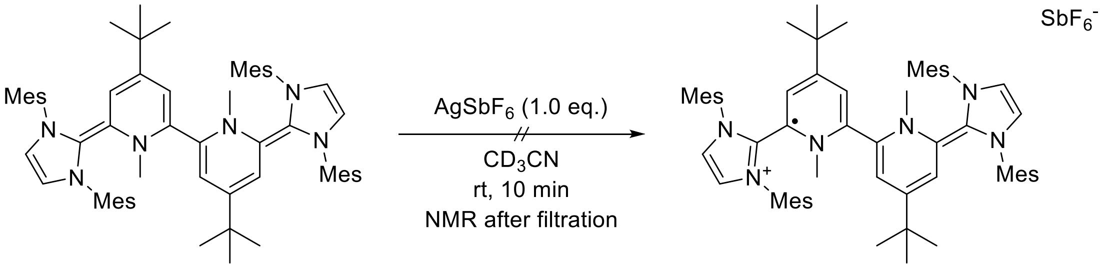

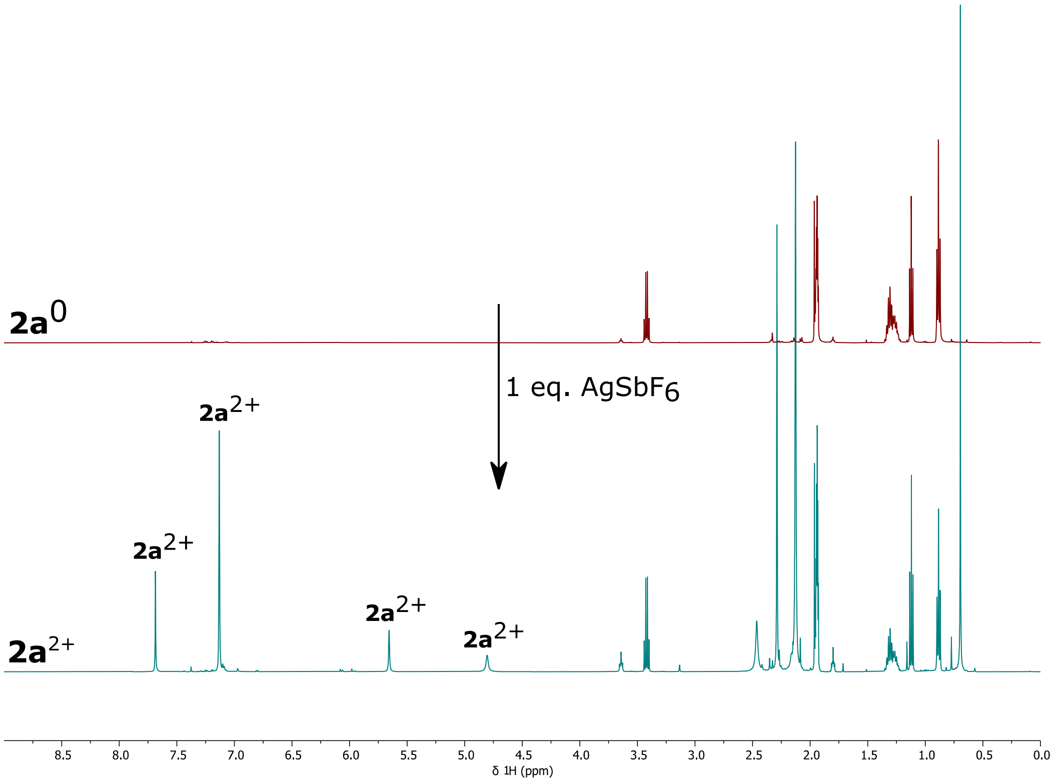

Figure S70. NMR experiment for the preparation of **2a**$^{+}$ leading to **2a** and **2a**$^{2+}$.

Synthesis of **2b**$^+$: In case of **2b** we performed additional UV-vis-spectroelectrochemical measurements (Figures S119-120). Here an intense band appears at ~900 nm (UV-vis SEC only up to 900 nm) after oxidation of **2b**, at a potential that is insufficient for the complete conversion of **2b** to **2b**$^{2+}$. This signal vanishes again, as soon as a more positive potential is applied, indicating the formation of **2b**$^+$ in an equilibrium with **2b** and **2b**$^{2+}$. As expected it is not possible to obtain the newly formed signal without an intense band of **2b** at ~470 nm or the formation of signals corresponding to **2b**$^{2+}$. After oxidation with 1 eq. of $AgSbF_6$ in thf we observed an immediate change in color from dark red to olive green. It was not possible to obtain ^{1}H NMR signals after filtration of the sample. Nevertheless, a broad wave could be detected by X-band EPR spectroscopy (Figure S71).

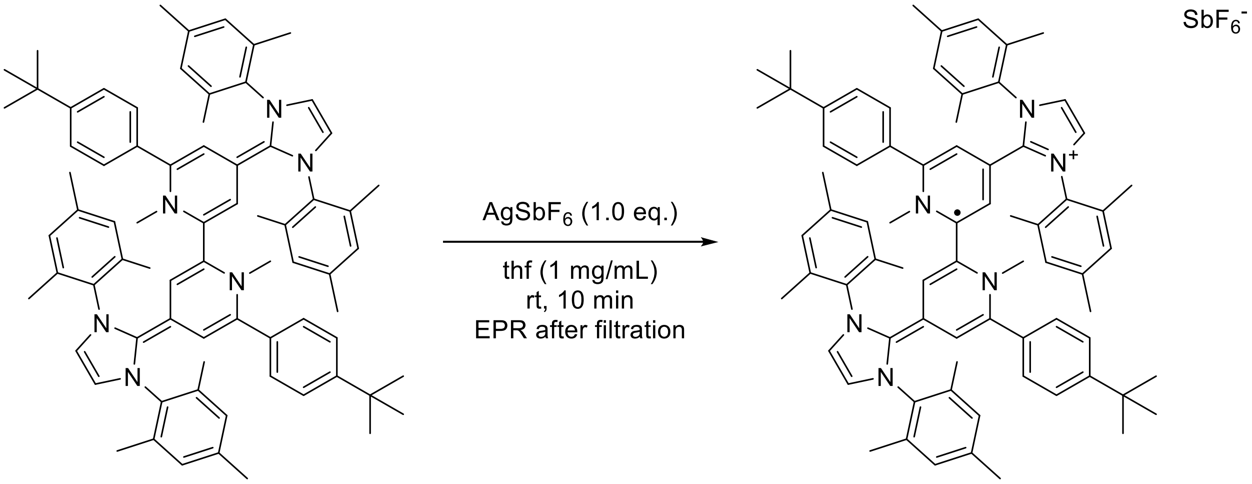

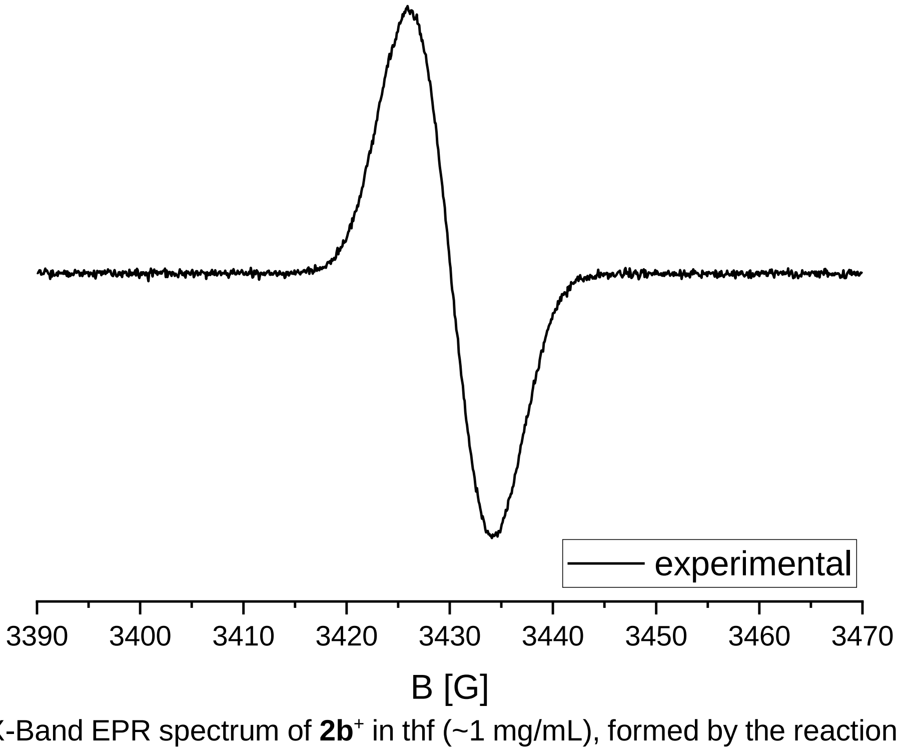

Figure S71. X-Band EPR spectrum of **2b**$^+$ in thf (~1 mg/mL), formed by the reaction of **2b** with 1 eq. $AgSbF_6$ in in THF, after filtration over a celite pad to remove precipitated Ag^0. Fitting parameter: g= 2.0031; LW 2.003; Hyperfine couplings could not be resolved.

UV-vis-NIR-spectroscopy shows the formation a new intense NIR signal (1367 nm), that neither corresponds to **2b** nor to **2b**$^{2+}$, further supporting the evidence for the formation of **2b**$^{+}$ (Figs. S72-S73). Attempts to isolate **2b**$^{+}$ by removal of the solvent under reduced pressure, precipitation by addition of an excess Et_2O to a filtered 1 mg/mL thf solution followed by crystallization at -40 °C was not successful. Only **2b** and **2b**$^{2+}$ were obtained indicating the equilibrium with the disproportionation products.

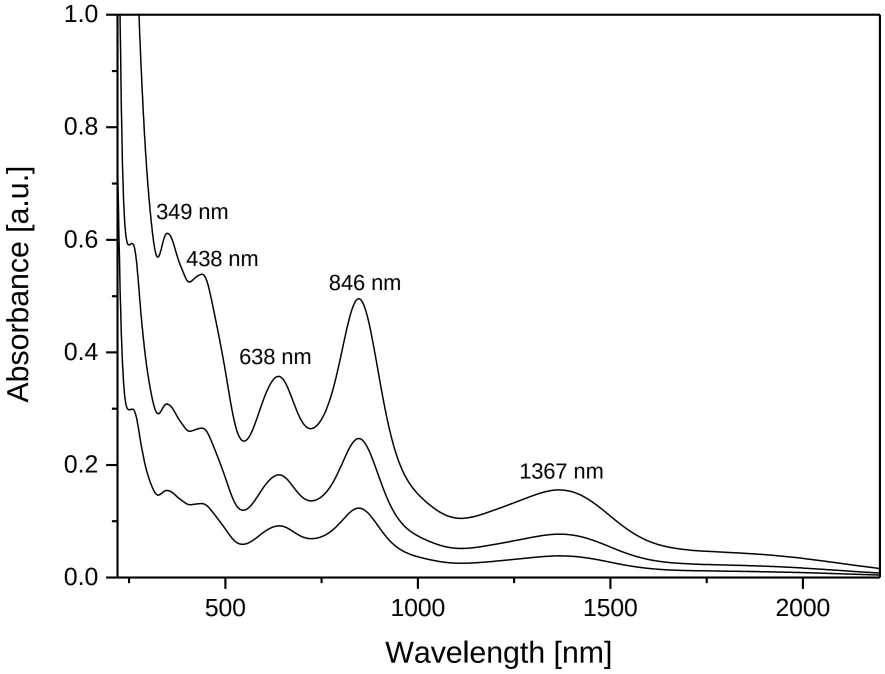

Figure S72. UV-vis-NIR spectra of *in situ* generated **2b**$^{+}$ (0.5 mg/ml, 0.25 mg/ml and 0.125 mg/ml) in thf. Measured in a 0.1 cm quartz cuvette.

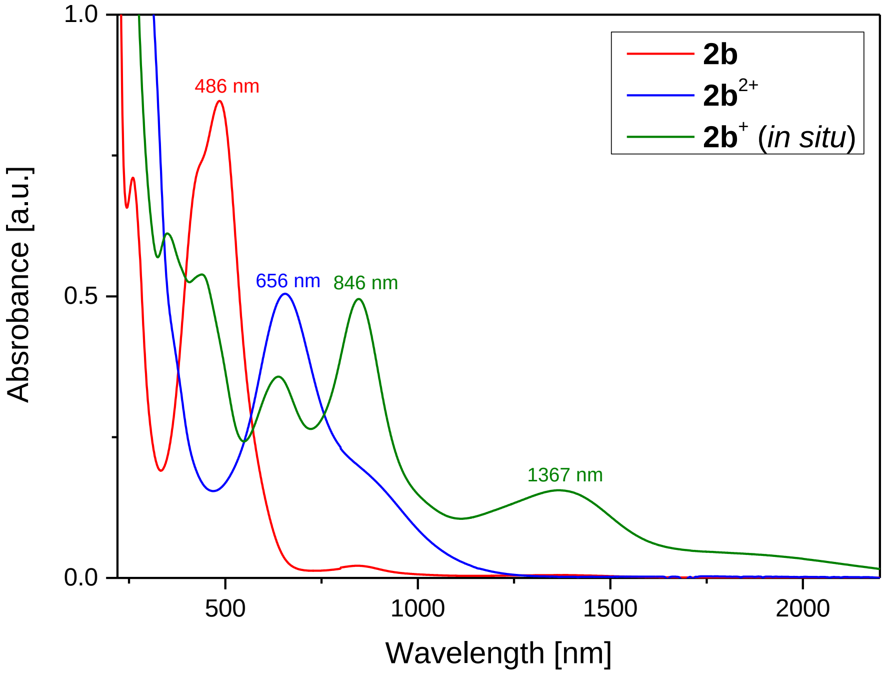

Figure S73. Comparison of the UV-vis-NIR spectra of *in situ* generated **2b**$^{+}$, **2b** and **2b**$^{2+}$ (0.5 mg/ml in thf).

5. X-ray characterization data

General part

Single crystal X-ray diffraction analysis

Data collection was performed on a *Bruker D8 Venture* four-circle-diffractometer from *Bruker AXS GmbH*; used detector: *Photon II* from *Bruker AXS GmbH; Photon III* from *Bruker AXS GmbH*; used X-ray sources: microfocus *IµS* Cu/Mo from *Incoatec GmbH* with mirror optics *HELIOS* and single-hole collimator from *Bruker AXS GmbH*.

Used programs: *APEX3 Suite* (v2017.3-0) and therein integrated programs *SAINT* (Integration) und *SADABS* (Absorption correction) from *Bruker AXS GmbH*; structure solution was done with *SHELXT*, refinement with *SHELXS*;[6] OLEX was used for data finalization.[7]

Special Utilities: *SMZ1270* stereomicroscope from *Nikon Metrology GmbH* was used for sample preparation; crystals were mounted on *MicroMounts* or *MicroLoops* from *MiTeGen*; for sensitive samples the *X-TEMP 2 System* was used for picking of crystals;[8] crystals were cooled to given temperature with *Cryostream 800* from *Oxford Cryosystems.*

	**2a**	**$2a^{2+}$**	**$2a^{3+}$**
Empirical formula	$C_{69.99}H_{95.96}N_6O_2$	$C_{62}H_{76}F_{12}N_6Sb_2$	$C_{76}H_{106}F_{18}N_6O_{3.50}Sb_3$
Formula weight	1053.23	1376.78	1866.91
Temperature [K]	100.0	99.99	100.0
Crystal system	triclinic	triclinic	monoclinic
Space group (number)	(2)	(2)	(14)
a [Å]	12.0252(7)	8.3179(3)	24.932(3)
b [Å]	12.0506(6)	12.7755(5)	16.748(2)
c [Å]	24.3702(13)	15.6451(6)	22.0658(18)
α [°]	97.693(2)	101.4570(10)	90
β [°]	101.820(2)	95.8730(10)	116.013(5)
γ [°]	108.044(2)	105.7380(10)	90
Volume [$Å^3$]	3211.9(3)	1546.67(10)	8280.6(17)
Z	2	1	4
ρ_{calc} [gcm^{-3}]	1.089	1.478	1.498
μ [mm^{-1}]	0.065	0.953	1.061
F(000)	1148	700	3788
Crystal size [mm^3]	0.561×0.467×0.318	0.37×0.326×0.148	0.294×0.194×0.112
Crystal colour	red	violet	blue
Crystal shape	block	block	block
Radiation	MoK_α (λ=0.71073 Å)	MoK_α (λ=0.71073 Å)	MoK_α (λ=0.71073 Å)
2Θ range [°]	4.19 to 57.72 (0.74 Å)	4.82 to 61.34 (0.70 Å)	4.42 to 52.87 (0.80 Å)
Index ranges	-16 ≤ h ≤ 16<br>-16 ≤ k ≤ 16<br>-33 ≤ l ≤ 33	-11 ≤ h ≤ 11<br>-18 ≤ k ≤ 18<br>-22 ≤ l ≤ 22	-31 ≤ h ≤ 31<br>-20 ≤ k ≤ 20<br>-27 ≤ l ≤ 27
Reflections collected	102942	70677	16992
Independent reflections	16767 [R_{int} = 0.0414, R_{sigma} = 0.0319]	9399<br>R_{int} = 0.0186<br>R_{sigma} = 0.0118	16992<br>R_{int} = 0.0494<br>R_{sigma} = 0.0202
Completeness to Θ = 25.242°	99.9 %	99.8 %	99.9 %
Data / Restraints / Parameters	16767/178/815	9399/0/380	16992/223/1084
Goodness-of-fit on F^2	1.050	1.093	1.026
Final R indexes [I≥2σ(I)]	R_1 = 0.0512<br>wR_2 = 0.1451	R_1 = 0.0220<br>wR_2 = 0.0568	R_1 = 0.0504<br>wR_2 = 0.1474
Final R indexes [all data]	R_1 = 0.0538<br>wR_2 = 0.1482	R_1 = 0.0227<br>wR_2 = 0.0572	R_1 = 0.0617<br>wR_2 = 0.1611
Largest peak/hole [$eÅ^{-3}$]	0.34/-0.48	0.90/-0.60	1.35/-0.95

Table 1. Summary of X-ray data.

	**2b**	**2c**	**$2c^{3+}$**
Empirical formula	$C_{49}H_{66}N_3O_3$	$C_{79}H_{89}N_6O_{0.50}$	$C_{87}H_{100}Cl_6F_9N_6O_{10}S_3$
Formula weight	745.04	1130.56	1869.60
Temperature [K]	100.0	100.0	100.00
Crystal system	monoclinic	triclinic	monoclinic
Space group (number)	(14)	(2)	(14)
a [Å]	17.0351(13)	14.6049(15)	13.0562(4)
b [Å]	14.7168(11)	15.9055(15)	30.0892(12)
c [Å]	17.1083(12)	16.2626(16)	23.8143(9)
α [°]	90	69.856(2)	90
β [°]	97.434(2)	68.453(3)	100.3160(10)
γ [°]	90	87.400(3)	90
Volume [$Å^3$]	4253.0(5)	3284.6(6)	9204.2(6)
Z	4	2	4
ρ_{calc} [gcm^{-3}]	1.164	1.143	1.349
μ [mm^{-1}]	0.072	0.067	0.332
F(000)	1620	1218	3900
Crystal size [mm^3]	0.284×0.159×0.064	0.372×0.355×0.188	0.473×0.158×0.148
Crystal colour	yellow	red	red
Crystal shape	block	block	block
Radiation	MoK_α (λ=0.71073 Å)	MoK_α (λ=0.71073 Å)	MoK_α (λ=0.71073 Å)
2Θ range [°]	4.80 to 54.37 (0.78 Å)	3.79 to 55.04 (0.77 Å)	4.48 to 54.29 (0.78 Å)
Index ranges	-21 ≤ h ≤ 21<br>-18 ≤ k ≤ 18<br>-21 ≤ l ≤ 21	-18 ≤ h ≤ 18<br>-20 ≤ k ≤ 20<br>-21 ≤ l ≤ 21	-16 ≤ h ≤ 15<br>-38 ≤ k ≤ 33<br>-30 ≤ l ≤ 30
Reflections collected	122693	30323	162457
Independent reflections	9416<br>R_{int} = 0.0927<br>R_{sigma} = 0.0386	30323<br>R_{int} = 0.0926<br>R_{sigma} = 0.0560	20348<br>R_{int} = 0.0320<br>R_{sigma} = 0.0180
Completeness to Θ = 25.242°	99.9 %	99.7 %	99.9 %
Data / Restraints / Parameters	9416/0/507	30323/78/872	20348/118/1227
Goodness-of-fit on F^2	1.036	1.056	1.017
Final R indexes [I≥2σ(I)]	R_1 = 0.0562<br>wR_2 = 0.1329	R_1 = 0.0847<br>wR_2 = 0.2175	R_1 = 0.0434<br>wR_2 = 0.1129
Final R indexes [all data]	R_1 = 0.0863<br>wR_2 = 0.1513	R_1 = 0.0992<br>wR_2 = 0.2292	R_1 = 0.0518<br>wR_2 = 0.1194
Largest peak/hole [$eÅ^{-3}$]	0.36/-0.35	0.44/-0.30	0.69/-0.49

Table 2. Summary of X-ray data.

6. Electrochemical measurements

CVs were measured with a Gamry Instruments Reference 600+ and integrated iR compensation using the positive feedback method. Samples were measured under inert atmosphere in a nitrogen glove box at room temperature in dry solution of the respective solvent containing tetrabutylammonium hexafluorophosphate (0.1 M). Electrochemical grade tetrabutylammonium hexafluorophosphate was molten under vacuum prior to use. The setup consisted of a three-neck flask with a three-electrode setup containing a glassy carbon working electrode (GC: CH Instruments, ALS Japan; A = 7.1 mm^2), a platinum wire as a counter electrode, and an Ag/$AgNO_3$ reference electrode (0.01 M $AgNO_3$ in 0.1 M nBu_4NPF_6 in CH_3CN). The reference electrode was freshly prepared by using a fritted sample holder (Vycor glass), which was activated by storing it in a CH_3CN solution for one night, followed by diluted HCl (1M) for one night, dried and stored in THF for at least one additional night. To the fritted sample holder was added a freshly prepared 0.01 M $AgNO_3$/0.1 M nBu_4NPF_6 solution in CH_3CN and a silver wire. The working electrode was cleaned before measuring a new compound by standard methods: washed with water, polished with an alox-slurry (0.05 µm), washed with millipore water, sonicated in HPLC grade EtOH for 3 minutes, rinsed with millipore water and HPLC grade EtOH, and dried. Initially a blank sample only containing electrolyte in the respective solvent (0.1M) was measured and the potential cycled for 3-5 scans until a stable and clean potential was reached. Then the 3-neck cell was emptied and a specific amount of compound dissolved in 3 mL THF added and the CV measured. The system was furthermore (doubly) referenced internally by addition of ferrocene or diacetyl ferrocene. In case of diacetylferrocene potentials were referenced against ferrocene (diacetyl ferrocene to ferrocene ΔE = 430 mV in THF).[9]

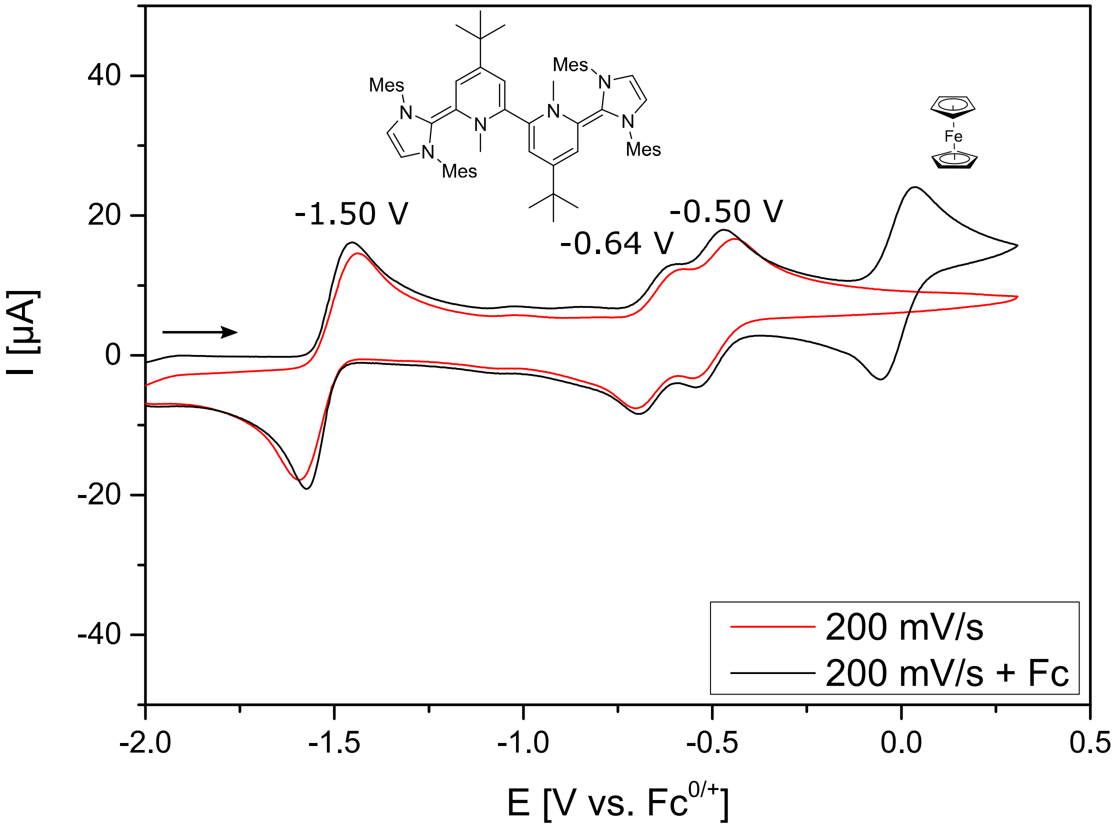

Figure S74. Cyclic voltammograms of **2a** (0.5±0.1 mg mL^{-1}) in thf (0.1 M *n*-Bu_4NPF_6) at room temperature; scan rate 200 mV s^{-1} (iR compensation = 1800 Ohm) referenced internally against ferrocene (arrows indicate scanning direction).

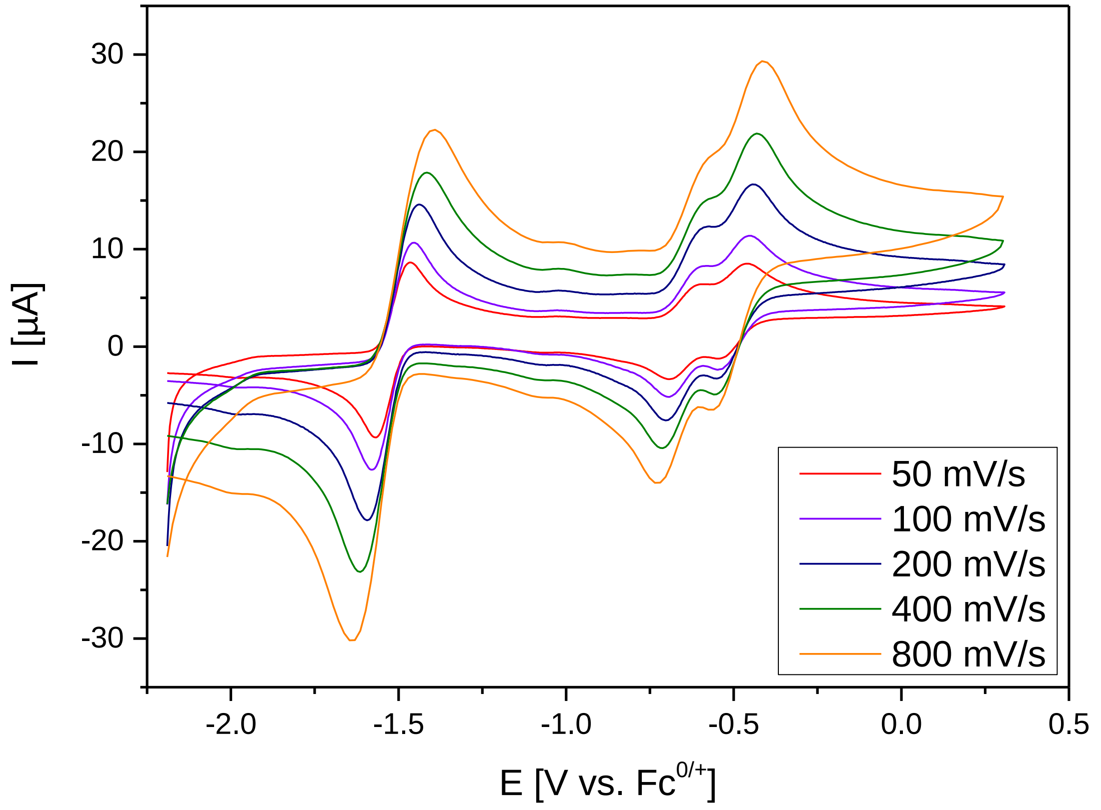

Figure S75. Cyclic voltammograms of **2a** (0.5±0.1 mg mL^{-1}) in thf (0.1 M *n*-Bu_4NPF_6) at room temperature; different scan rates (iR compensation = 1800 Ohm) referenced internally against ferrocene (arrows indicate scanning direction).

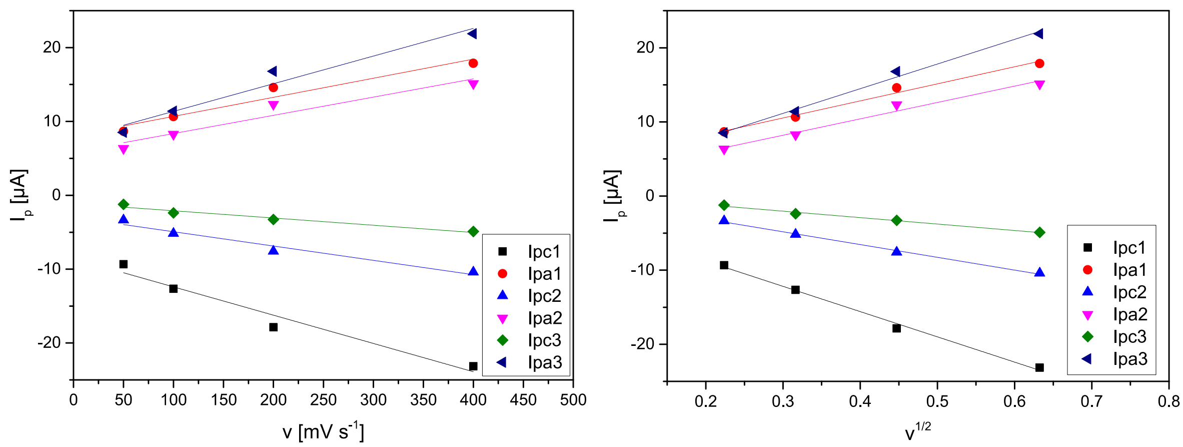

Figure S76. Exemplary plot of the peak anodic (index a) and peak cathodic (index c) current density of compound **2a** versus $v^{1/2}$ and v (v denotes scan speed). Linear response vs. $v^{1/2}$ points to freely diffusing species while linear response to v points to electrode adsorbed species. In all cases R_{fit} for the linear fit versus $v^{1/2}$ exceeds R_{fit} for the linear fit versus v.[10]

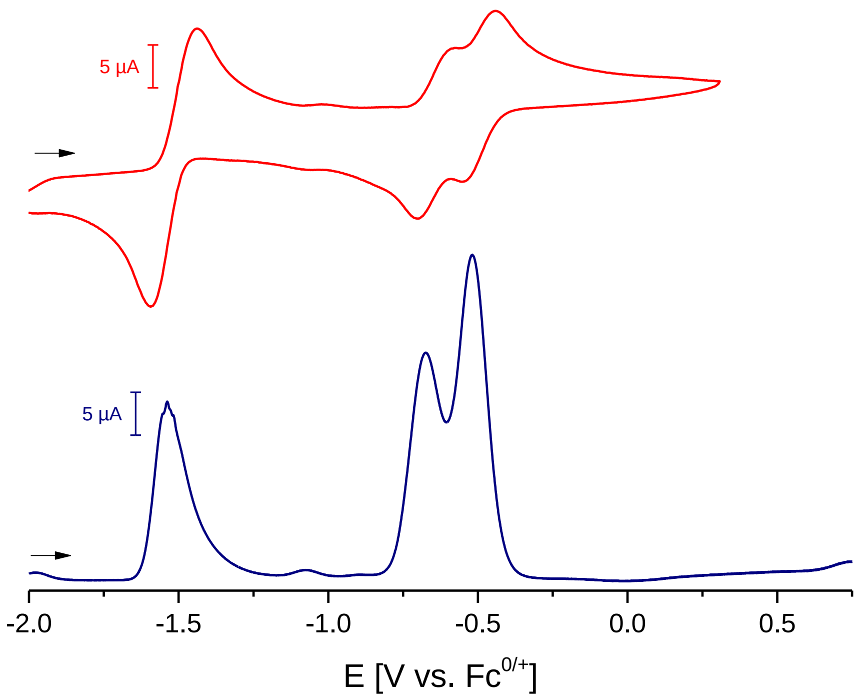

Figure S77. Top: Cyclic voltammograms of **2a** (0.5±0.1 mg mL^{-1}) in thf (0.1 M $n$$Bu_4NPF_6$) at room temperature; scan rate 200 mV*s^{-1} (iR compensation = 1800 Ohm) referenced internally against ferrocene (arrows indicate scanning direction). Bottom: Square wave voltammetry of **2a** (1.6±0.1 mg mL^{-1}) in THF (0.1 M n-Bu_4NPF_6) at room temperature; Experimental parameters: iR compensation = 2100 Ohm, step size: 1 mV, frequency: 15 Hz, pulse size: 25 mV. Referenced internally against ferrocene (arrows indicate scanning direction).

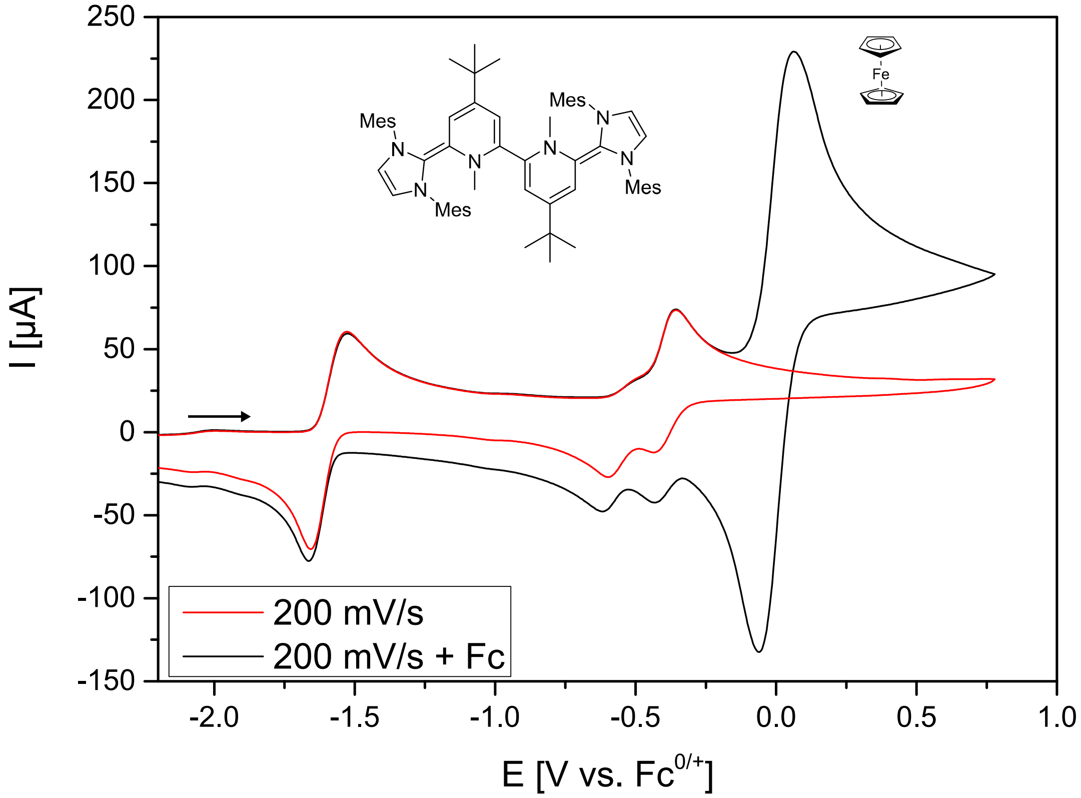

Figure S78. Cyclic voltammograms of **2a** (2.0±0.1 mg mL^{-1}) in CH_2Cl_2 (0.1 M *n*-Bu_4NPF_6) at room temperature; scan rate 200 mV*s^{-1} (iR Compensation = 780 Ohm) referenced internally against ferrocene (arrows indicate scanning direction).

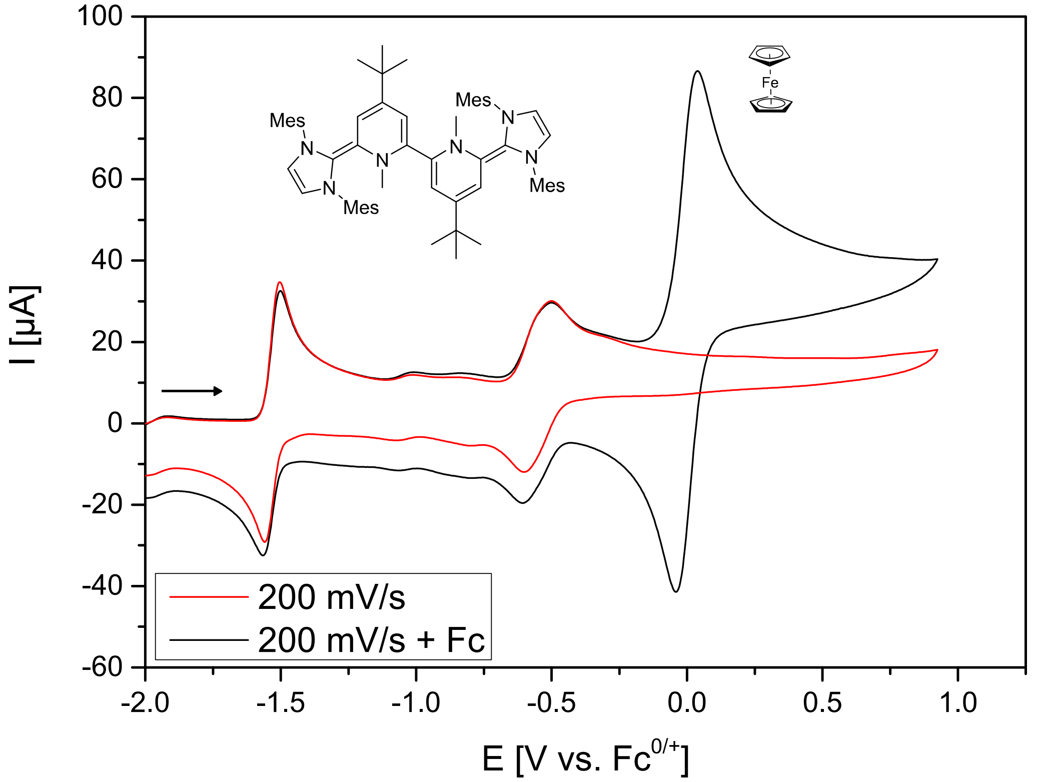

Figure S79. Cyclic voltammograms of **2a** (0.5±0.1 mg mL^{-1}, saturated solution, filtered through a syringe filter) in dmf (0.1 M *n*-Bu_4NPF_6) at room temperature; scan rate 200 mV*s^{-1} (iR Compensation = 215 Ohm) referenced internally against ferrocene (arrows indicate scanning direction).

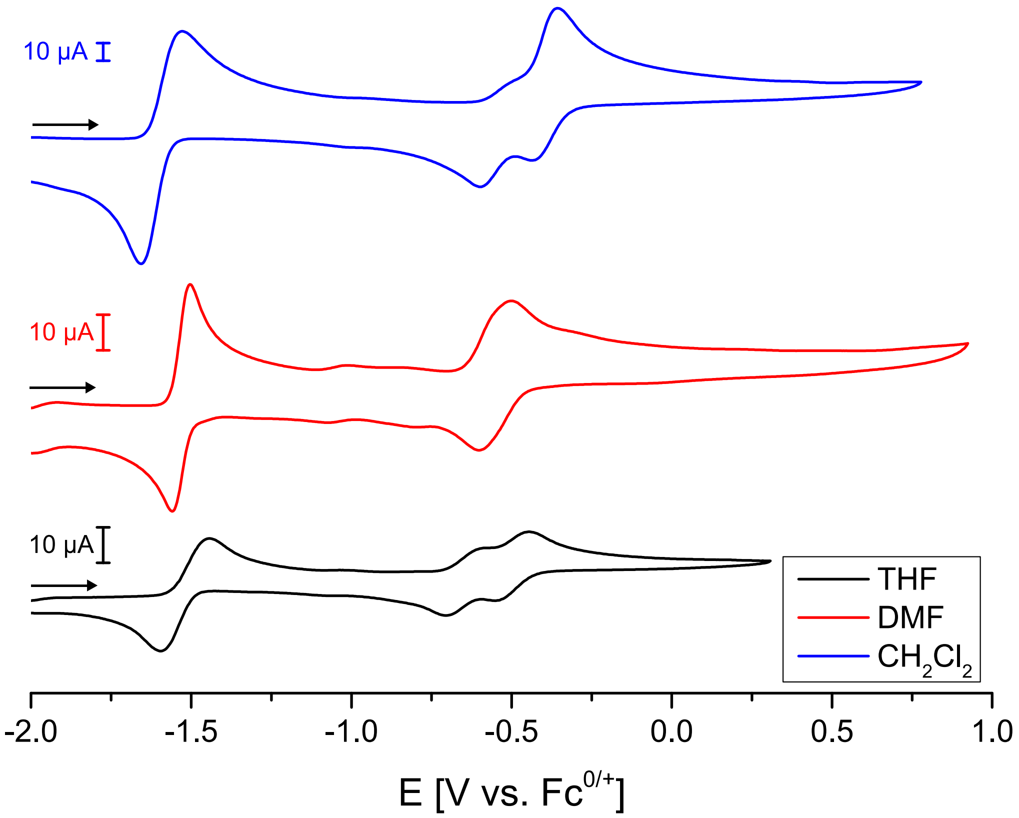

Figure S80. Comparison of cyclic voltammograms of **2a** in CH_2Cl_2 (top), dmf (middle) and thf (bottom) (0.1 M *n*-Bu_4NPF_6) at room temperature; scan rate 200 mV*s^{-1} referenced internally against ferrocene (arrows indicate scanning direction).

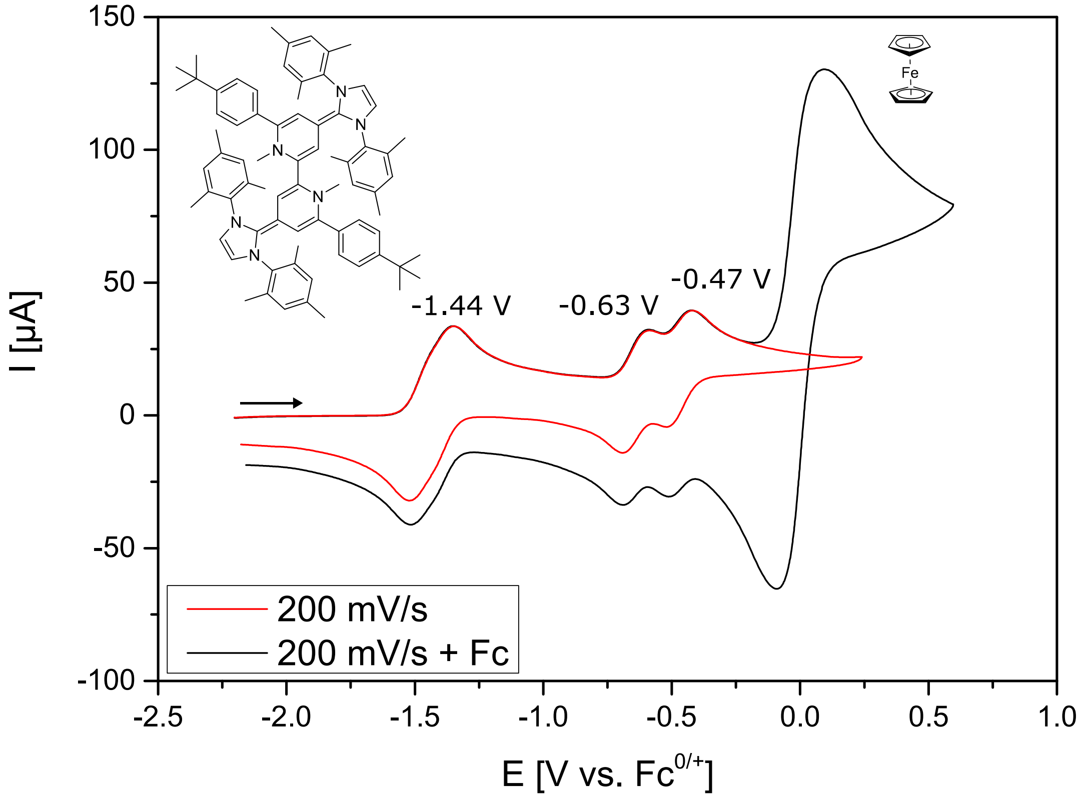

Figure S81. Cyclic voltammograms of **2b** (1.7±0.1 mg mL^{-1}) in THF (0.1 M *n*-Bu_4NPF_6) at room temperature; scan rate 200 mV s^{-1} (iR Compensation = 2200 Ohm) referenced internally against ferrocene (arrows indicate scanning direction).

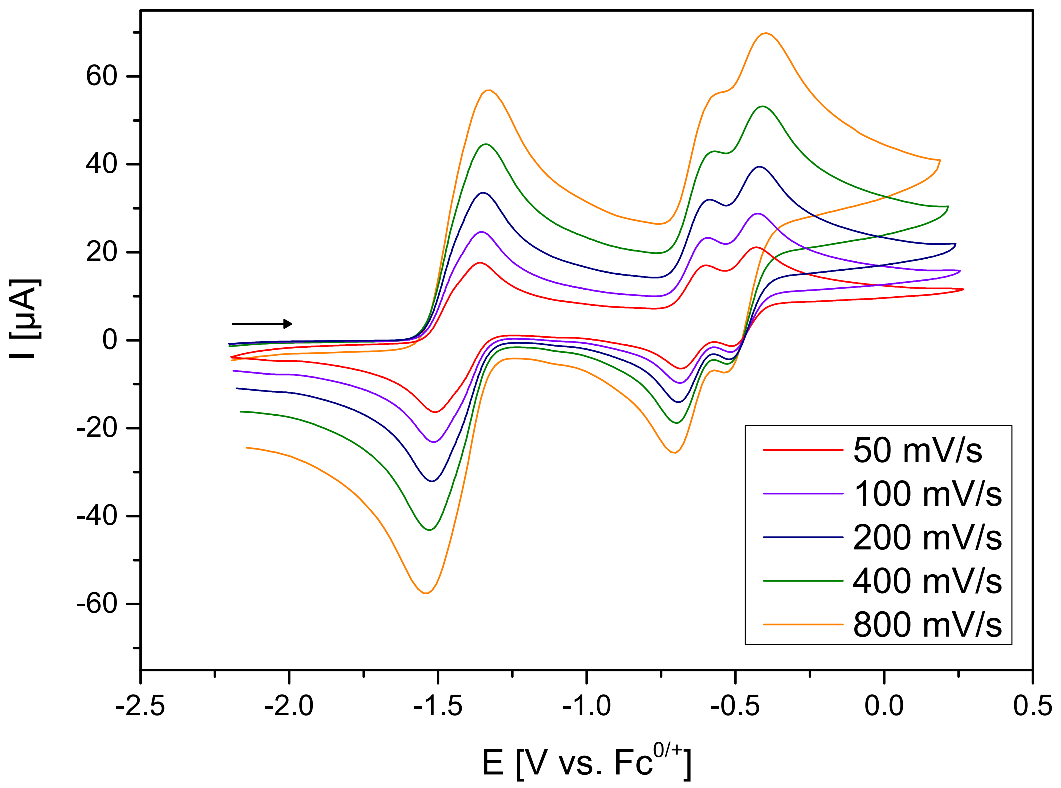

Figure S82. Cyclic voltammograms of **2b** (1.7±0.1 mg mL^{-1}) in THF (0.1 M *n*Bu_4NPF_6) at room temperature; different scan rates (iR Compensation = 2200 Ohm) referenced internally against ferrocene (arrows indicate scanning direction).

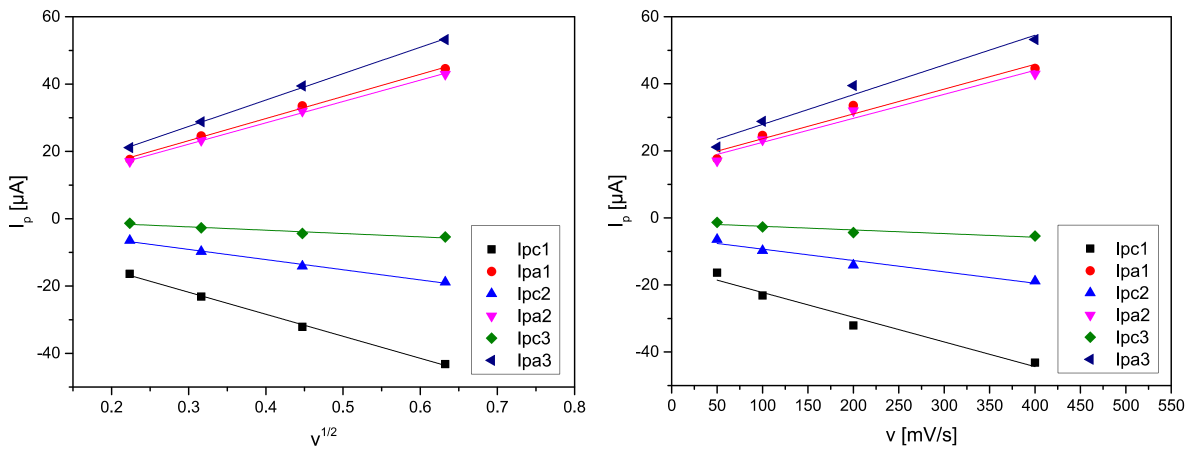

Figure S83. Exemplary plot of the peak anodic (index a) and peak cathodic (index c) current density of compound **2b** versus $v^{1/2}$ and v (v denotes scan speed). Linear response vs. $v^{1/2}$ points to freely diffusing species while linear response to v points to electrode adsorbed species. In all cases R_{fit} for the linear fit versus $v^{1/2}$ exceeds R_{fit} for the linear fit versus v.[10]

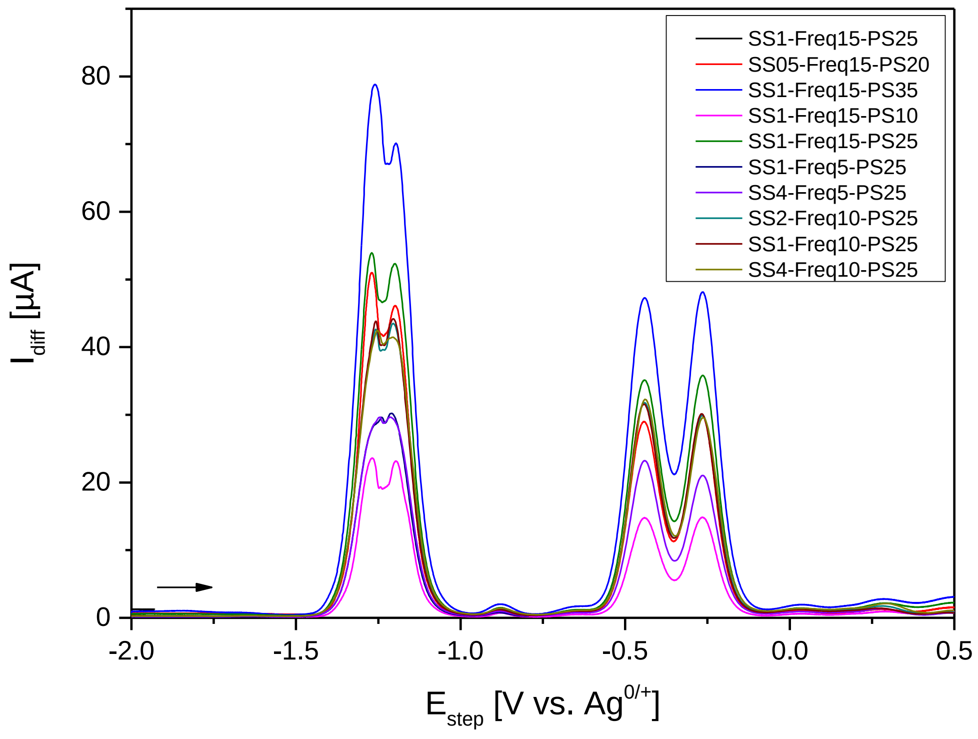

Figure S84. In case of **2b** the experimental parameters for the square wave voltammetry were optimized in thf solution to obtain maximum peak separation for the first two redox events. No internal reference was applied for the optimization. Square wave voltammetry of **2b** (1.3±0.1 mg mL^{-1}) in thf (0.1 M *n*-Bu_4NPF_6) at room temperature; SS: step size [mV]; Freq: Frequency [Hz]; PS: Pulse Size [mV].

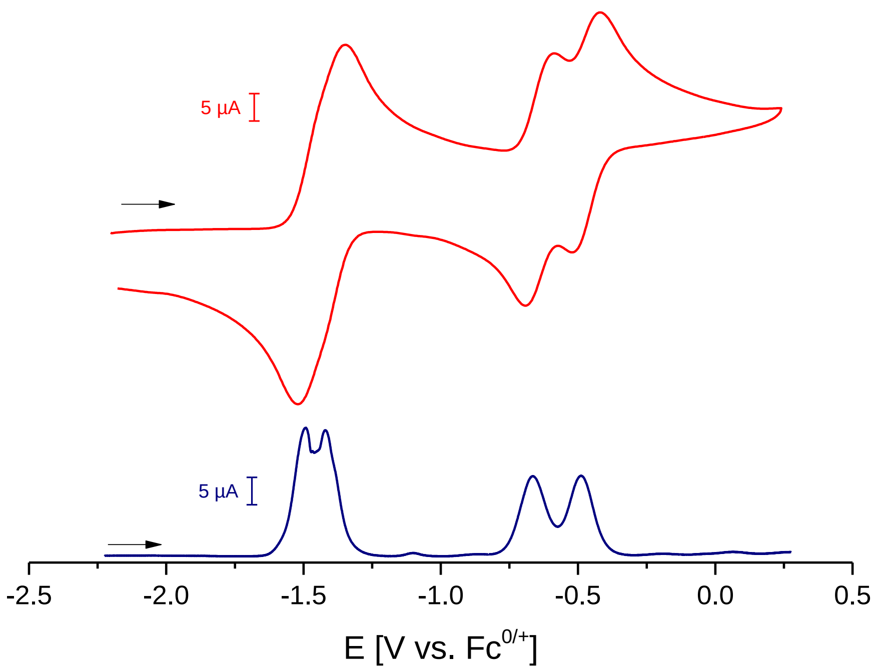

Figure S85. Top: Cyclic voltammograms of **2b** (1.7±0.1 mg mL^{-1}) in thf (0.1 M *n*-Bu_4NPF_6) at room temperature; scan rate 200 mV s^{-1} (iR Compensation = 2200 Ohm) referenced internally against ferrocene (arrows indicate scanning direction). Bottom: Square wave voltammetry of **2b** (1.3±0.1 mg mL^{-1}) in THF (0.1 M *n*-Bu_4NPF_6) at room temperature; Experimental parameters: iR Compensation = 2200 Ohm, step size: 1 mV, frequency: 15 Hz, pulse size: 25 mV. Referenced internally against ferrocene (arrows indicate scanning direction).

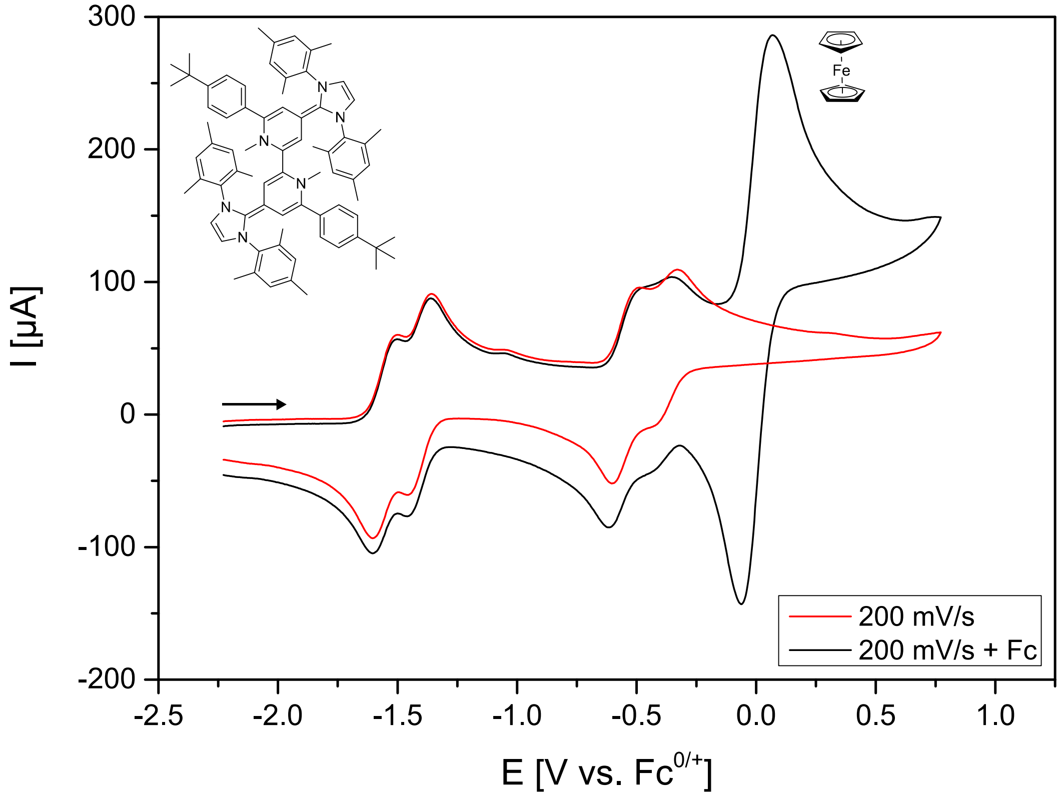

Figure S86. Cyclic voltammograms of **2b** (3.3±0.1 mg mL^{-1}) in CH_2Cl_2 (0.1 M *n*-Bu_4NPF_6) at room temperature; scan rate 200 mV s^{-1} (iR Compensation = 800 Ohm) referenced internally against ferrocene (arrows indicate scanning direction). Note: The solution changes color from red to olive green over 5 minutes at room temperature, indicating instability of **2b** in CH_2Cl_2.

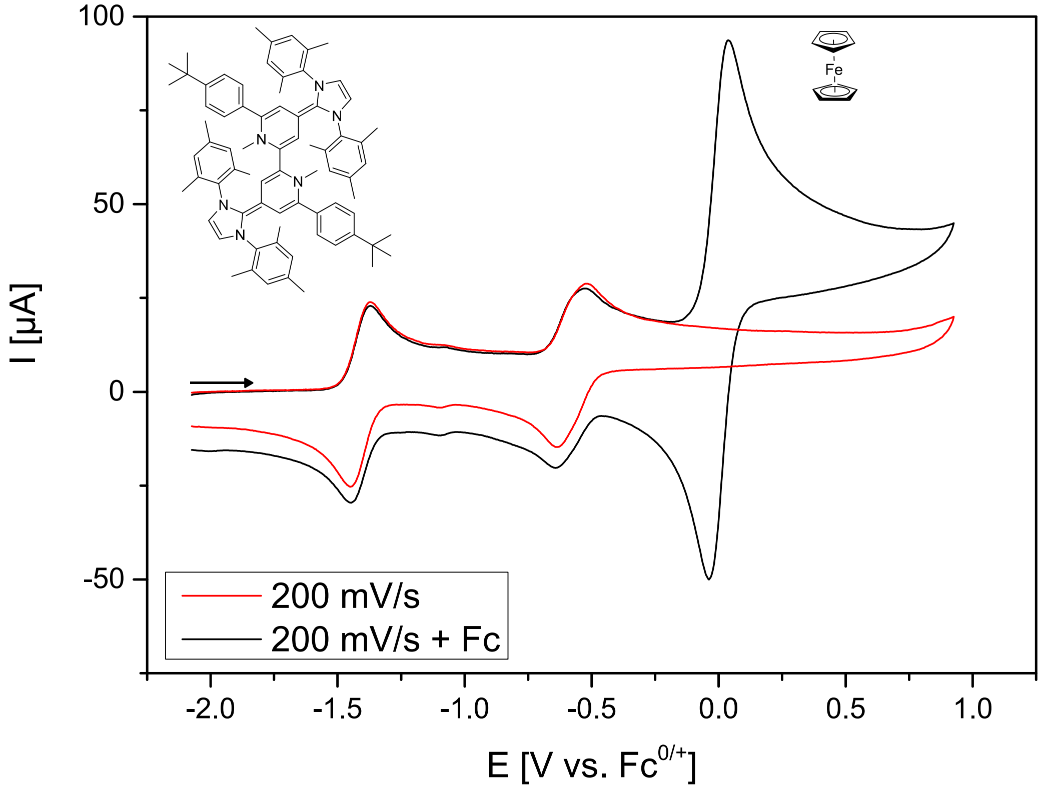

Figure S87. Cyclic voltammograms of **2b** (1.3±0.1 mg mL^{-1}) in dmf (0.1 M *n*-Bu_4NPF_6) at room temperature; scan rate 200 mV s^{-1} (iR Compensation = 320 Ohm) referenced internally against ferrocene (arrows indicate scanning direction).

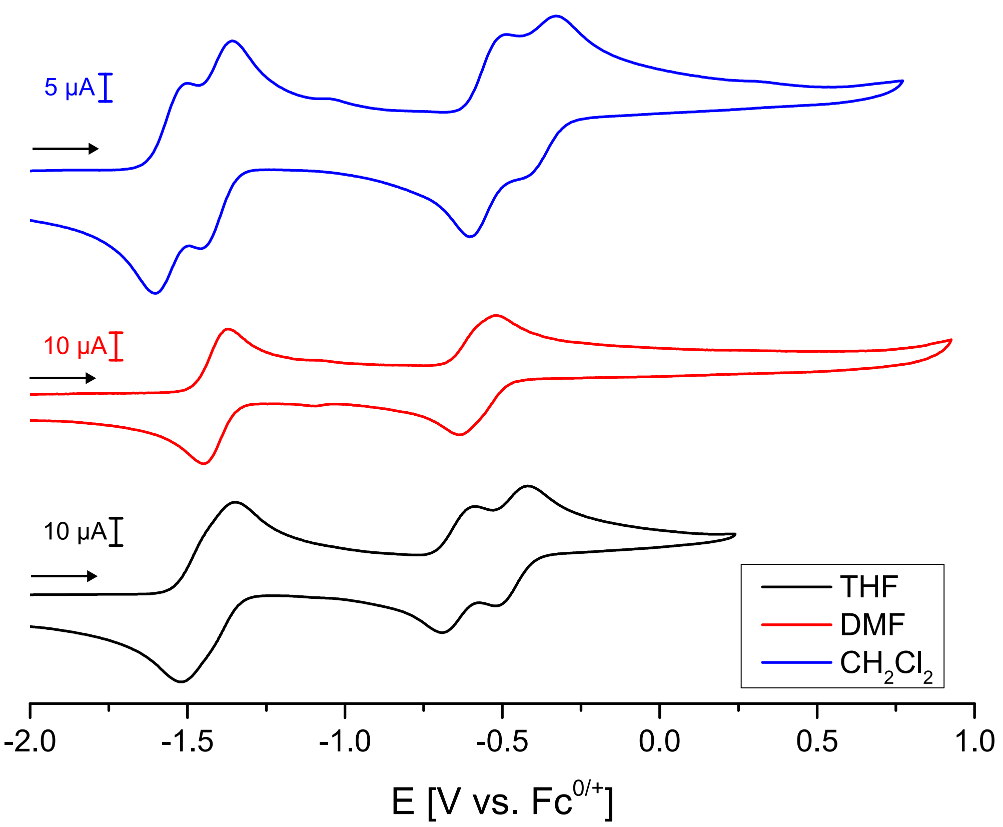

Figure S88. Comparison of cyclic voltammograms of **2b** in CH_2Cl_2 (top), dmf (middle) and thf (bottom) (0.1 M *n*-Bu_4NPF_6) at room temperature; scan rate 200 mV*s^{-1} referenced internally against ferrocene (arrows indicate scanning direction).

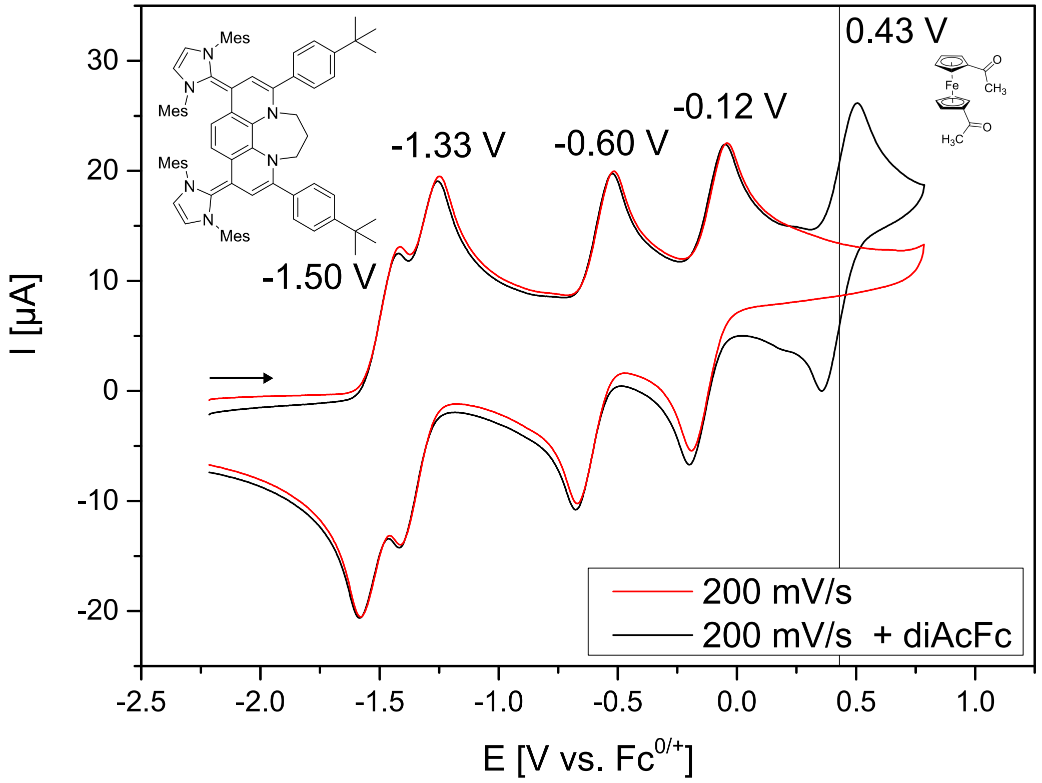

Figure S89. Cyclic voltammograms of **2c** (0.8±0.1 mg mL^{-1}) in thf (0.1 M *n*-Bu_4NPF_6) at room temperature; scan rate 200 mV s^{-1} (iR Compensation = 2000 Ohm) referenced internally against diacetylferrocene (arrows indicate scanning direction). Diacetylferrocene was used as standard since ferrocene would overlap with the fourth oxidation wave.

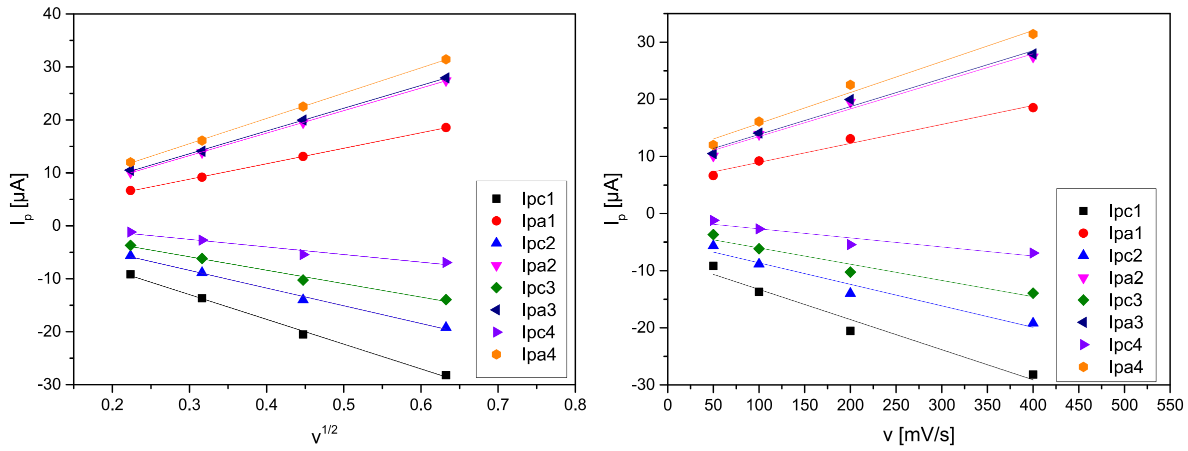

Figure S90. Exemplary plot of the peak anodic (index a) and peak cathodic (index c) current density of compound **2c** versus $v^{1/2}$ and v (v denotes scan speed). Linear response vs. $v^{1/2}$ points to freely diffusing species while linear response to v points to electrode adsorbed species. In all cases R_{fit} for the linear fit versus $v^{1/2}$ exceeds R_{fit} for the linear fit versus v.[10]

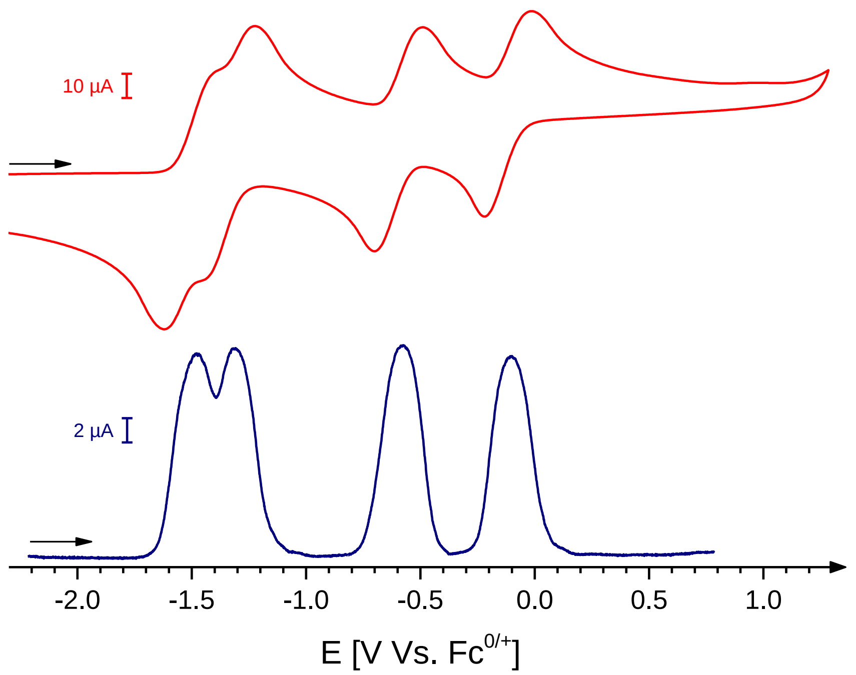

Figure S91. Top: Cyclic voltammograms of **2c** (3±0.1 mg mL^{-1}) in thf (0.1 M *n*-Bu_4NPF_6) at room temperature; scan rate 200 mV s^{-1} (iR Compensation = 2170 Ohm) referenced internally against ferrocene (arrows indicate scanning direction). Bottom: Square wave voltammetry of **2c** (3.0±0.1 mg mL^{-1}) in thf (0.1 M *n*-Bu_4NPF_6) at room temperature; Experimental parameters: iR Compensation = 2170 Ohm, step size: 1 mV, frequency: 15 Hz, pulse size: 25 mV. Referenced internally against ferrocene (arrows indicate scanning direction).

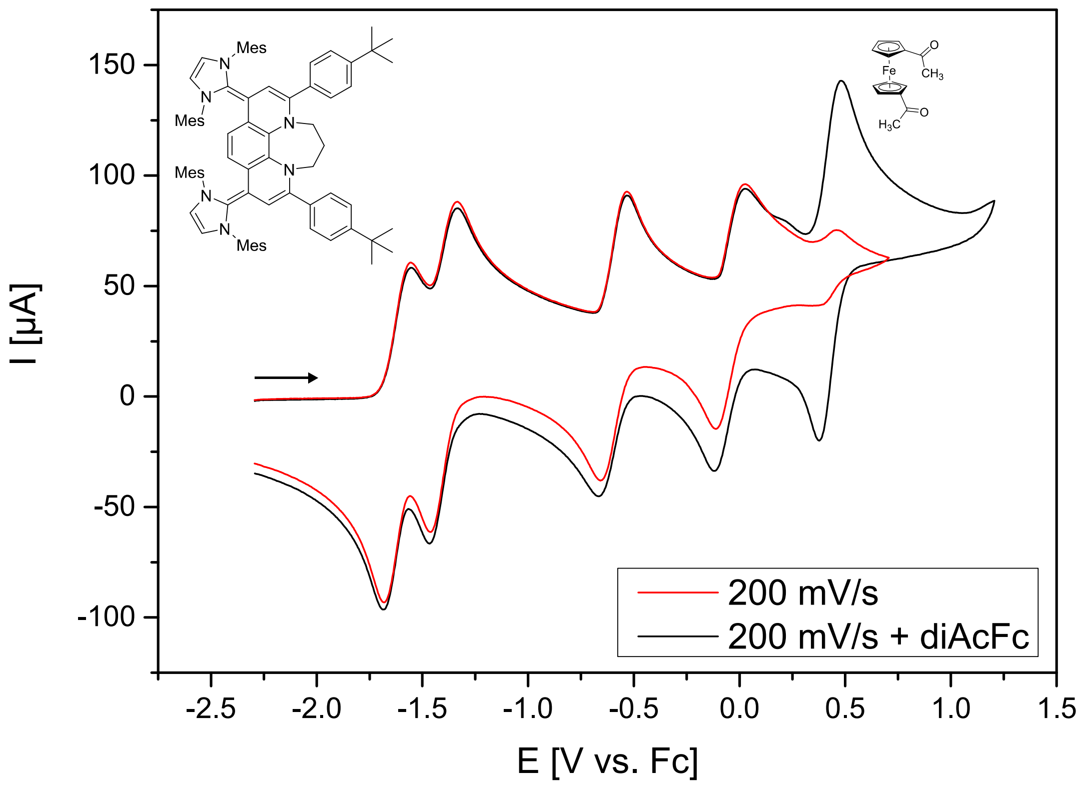

Figure S92. Cyclic voltammograms of **2c** (3.0±0.1 mg mL^{-1}) in CH_2Cl_2 (0.1 M *n*-Bu_4NPF_6) at room temperature; scan rate 200 mV s^{-1} (iR Compensation = 800 Ohm) referenced internally against ferrocene (arrows indicate scanning direction).

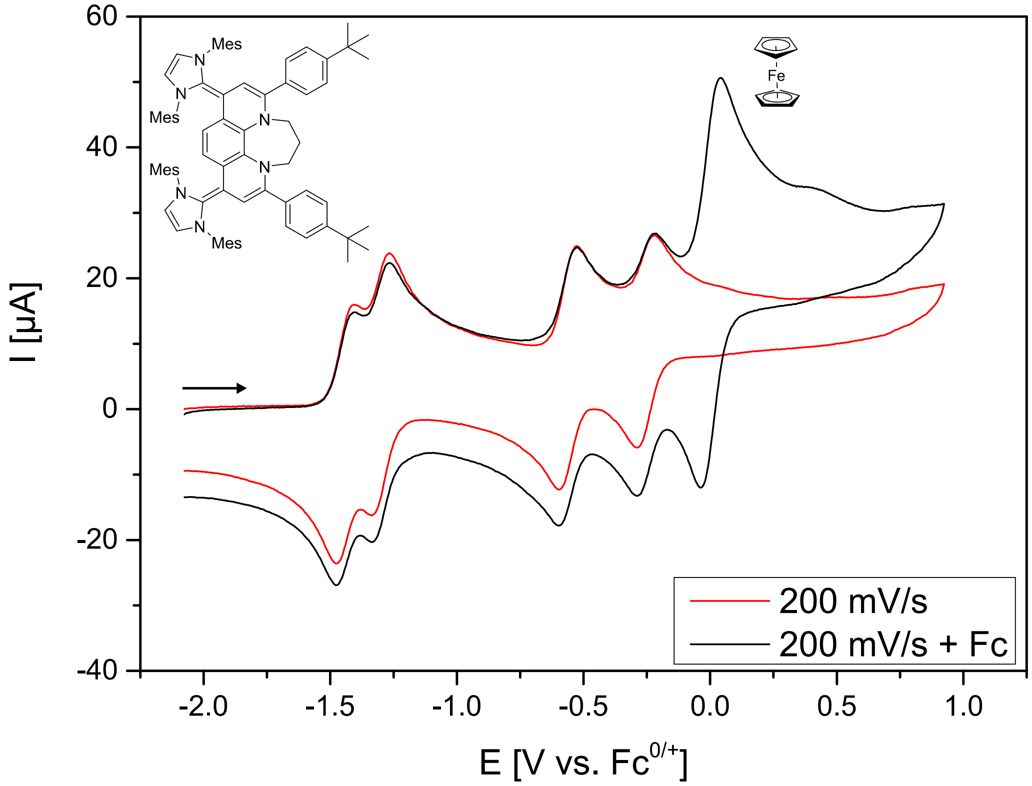

Figure S93. Cyclic voltammograms of **2c** (1.1±0.1 mg mL^{-1}) in dmf (0.1 M *n*-Bu_4NPF_6) at room temperature; scan rate 200 mV s^{-1} (iR Compensation = 250 Ohm) referenced internally against ferrocene (arrows indicate scanning direction).

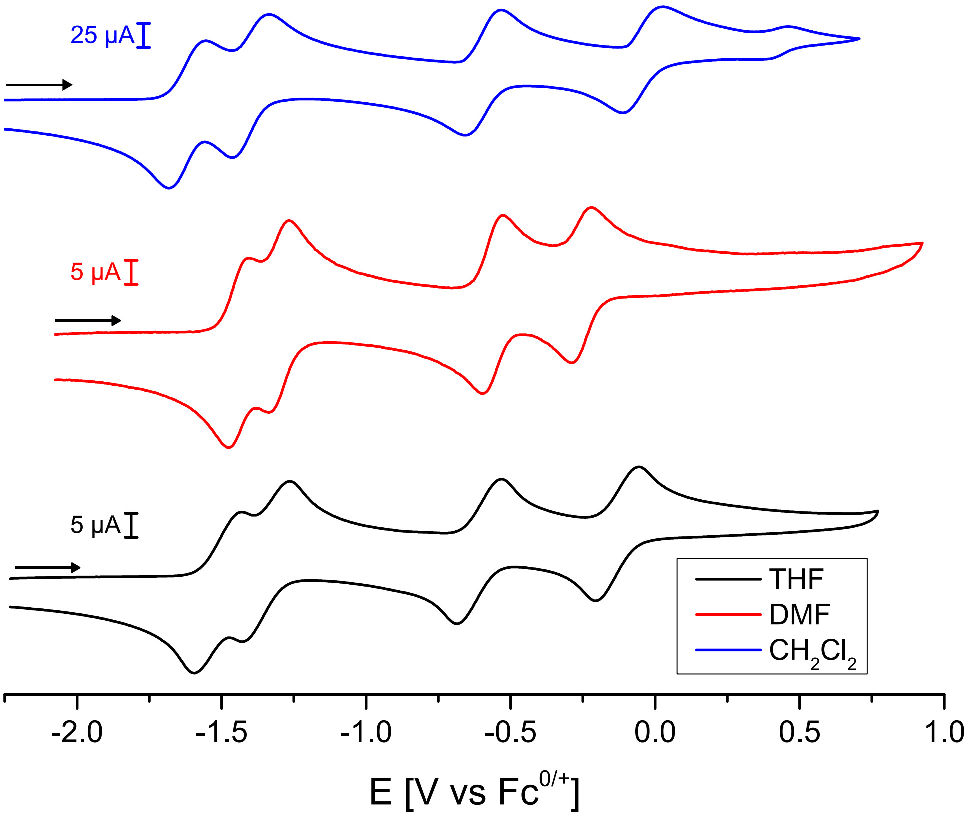

Figure S94. Comparison of cyclic voltammograms of **2c** in CH_2Cl_2 (top), dmf (middle) and thf (bottom) (0.1 M *n*-Bu_4NPF_6) at room temperature; scan rate 200 mV s^{-1} referenced internally against ferrocene (arrows indicate scanning direction).

7. EPR Spectroscopy

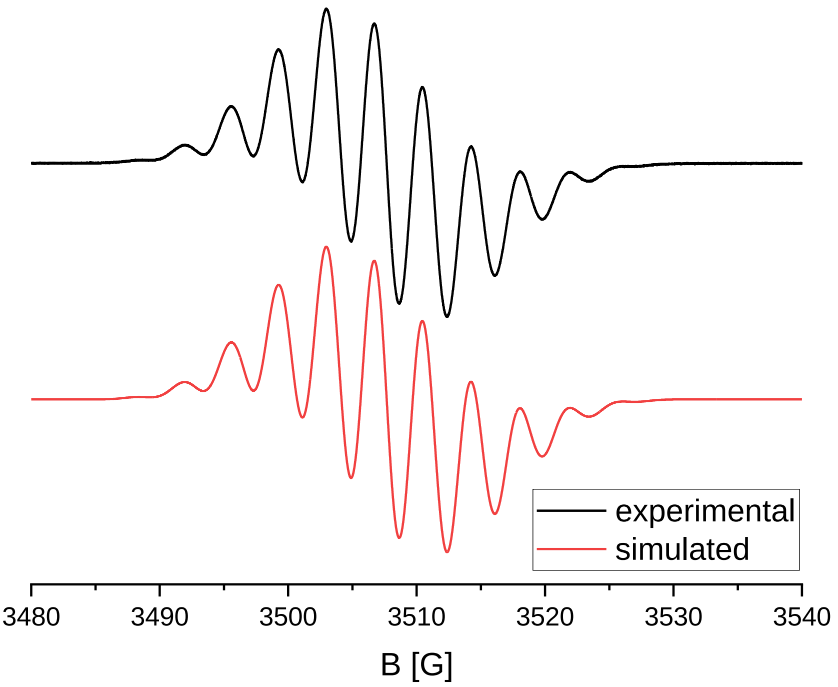

Figure S95. X-Band EPR spectrum of **2a**$^{3+}$ in THF (1 mg/mL).

Fitting parameter: g= 2.0030; LW 0.167

Hyperfine coupling: 2xN: 10.52 MHz; 4xN: 1.91 MHz; 6xH: 9.79.

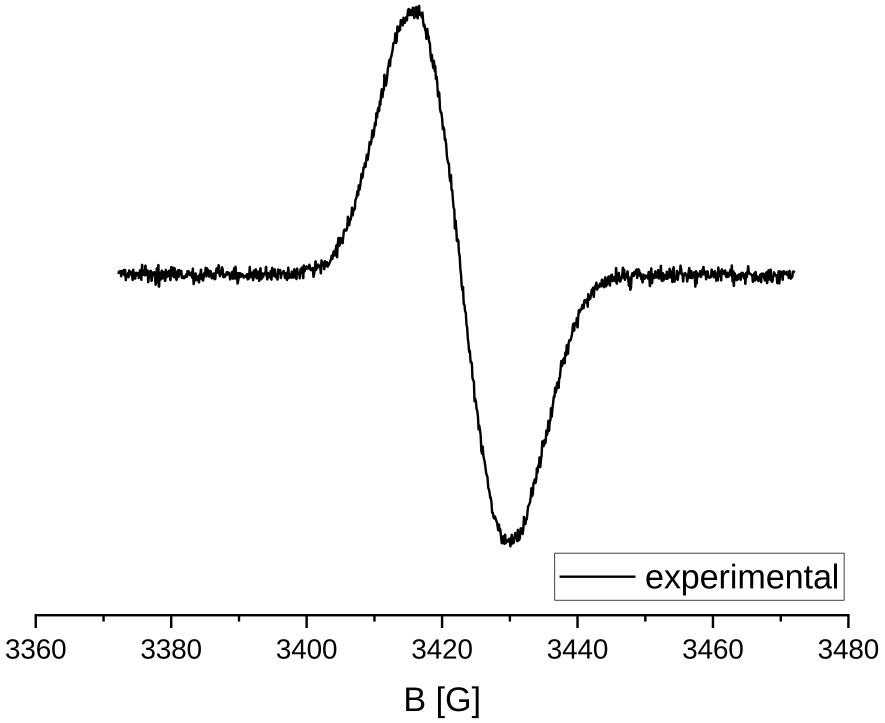

Figure S96. X-Band EPR spectrum of **2b**$^{3+}$ in THF (1 mg/mL).

Fitting parameter: g= 2.0031; LW 2.003

Hyperfine couplings could not be resolved.

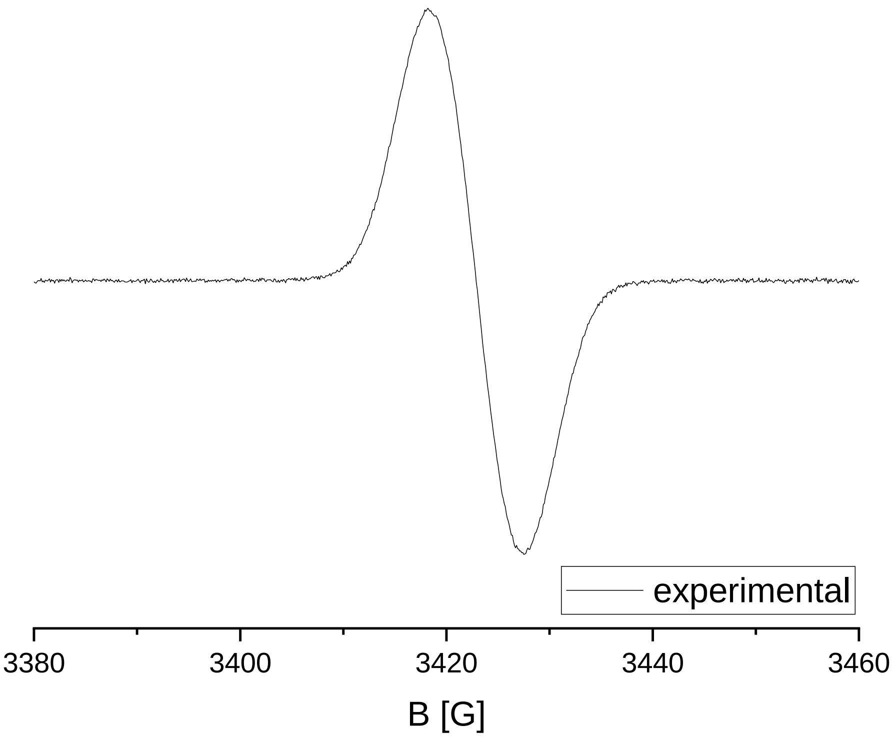

Figure S97. X-Band EPR spectrum of **2c**$^{+}$ in THF (1 mg/mL).

Fitting parameter: g= 2.0032; LW 1.032

Hyperfine couplings could not be resolved.

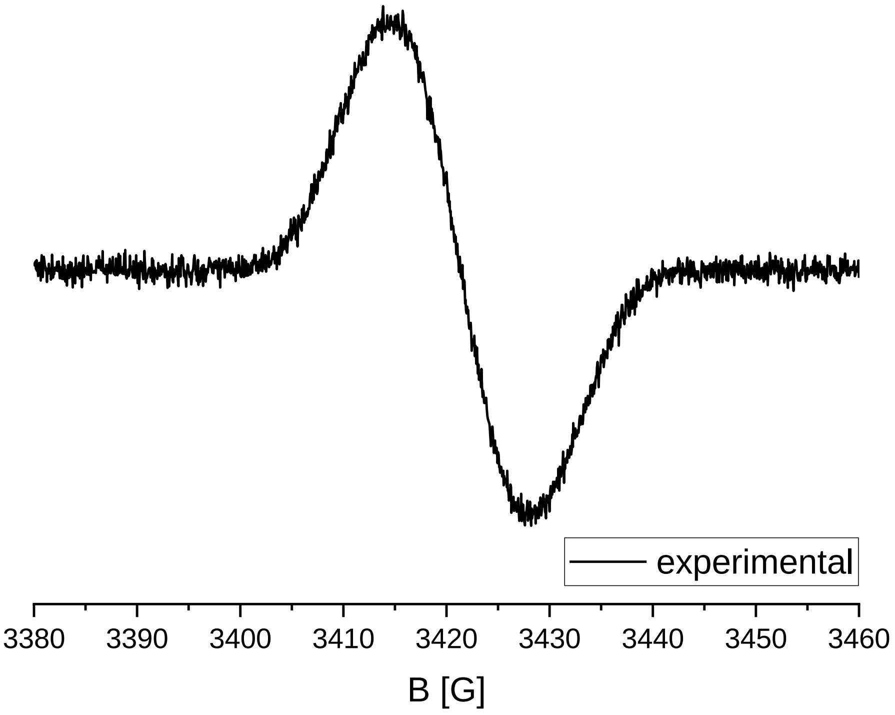

Figure S98. X-Band EPR spectrum of **2c**$^{3+}$ in THF (1 mg/mL).

Fitting parameter: g= 2.0033; LW 1.531

Hyperfine couplings could not be resolved.

8. UV-vis-NIR spectroscopy

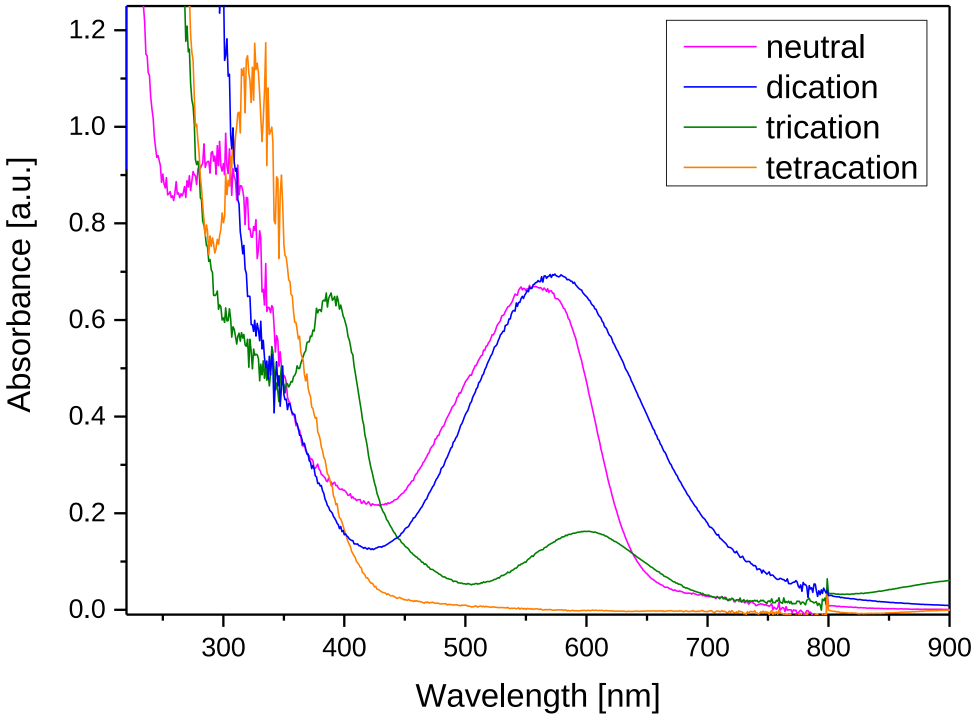

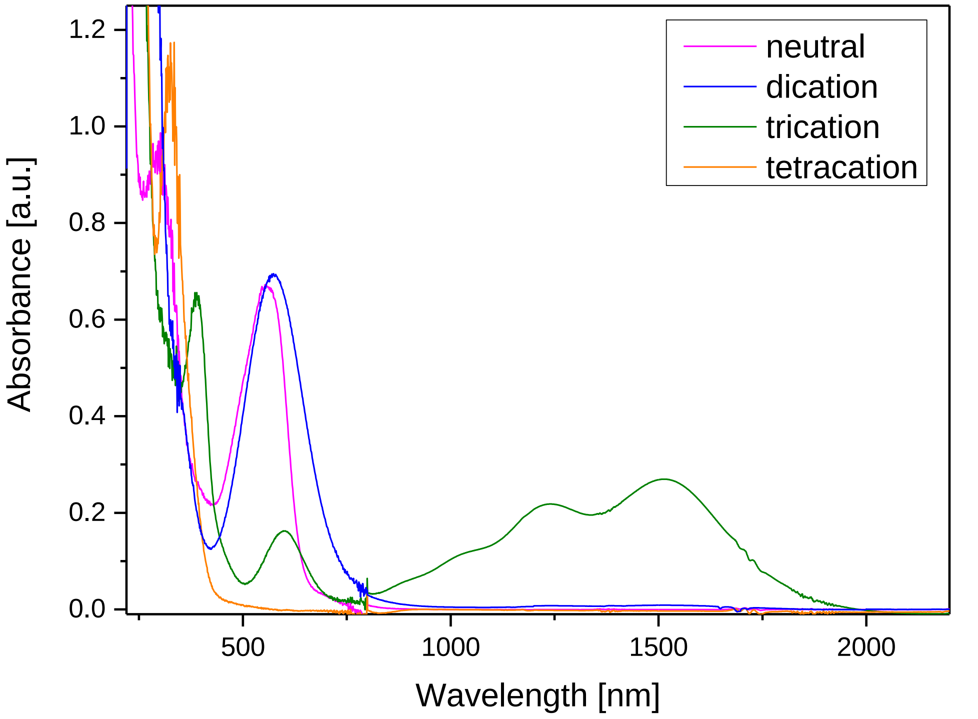

Figure S99: UV-vis-NIR spectra of **2a** (violet), **2a^{2+}** (blue), **2a^{3+}** (green), **2a^{4+}** (orange) in thf or CH_2Cl_2 (**2a^{2+}**) at c = 0.5 mg mL^{-1}, 0.1 cm quartz cuvette. Data shown as measured. Data shown in the manuscript (Figure 4) were smoothed using adjacent averaging (10 points window).

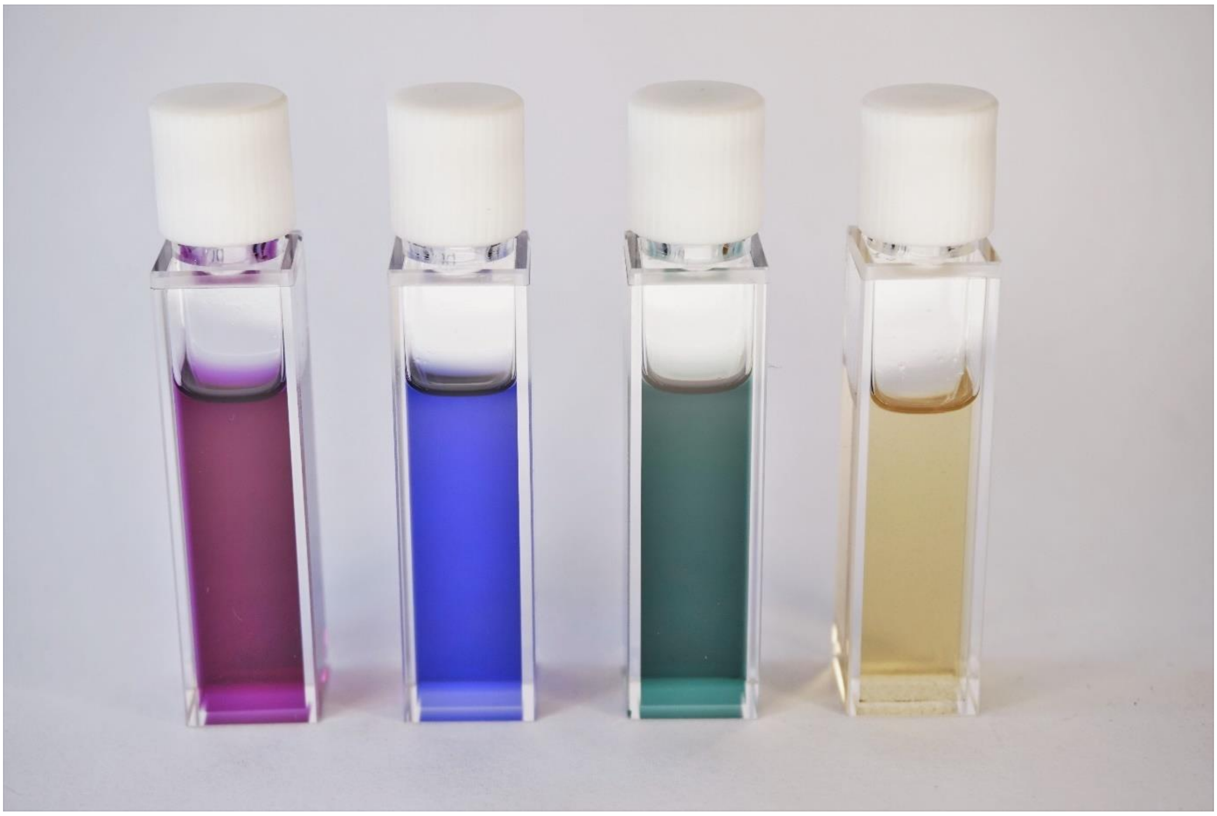

Figure S100. Photograph of the isolated oxidation states of the **2a** redox-system (increasing oxidation from left to right), in 1.0 cm quartz cuvettes.

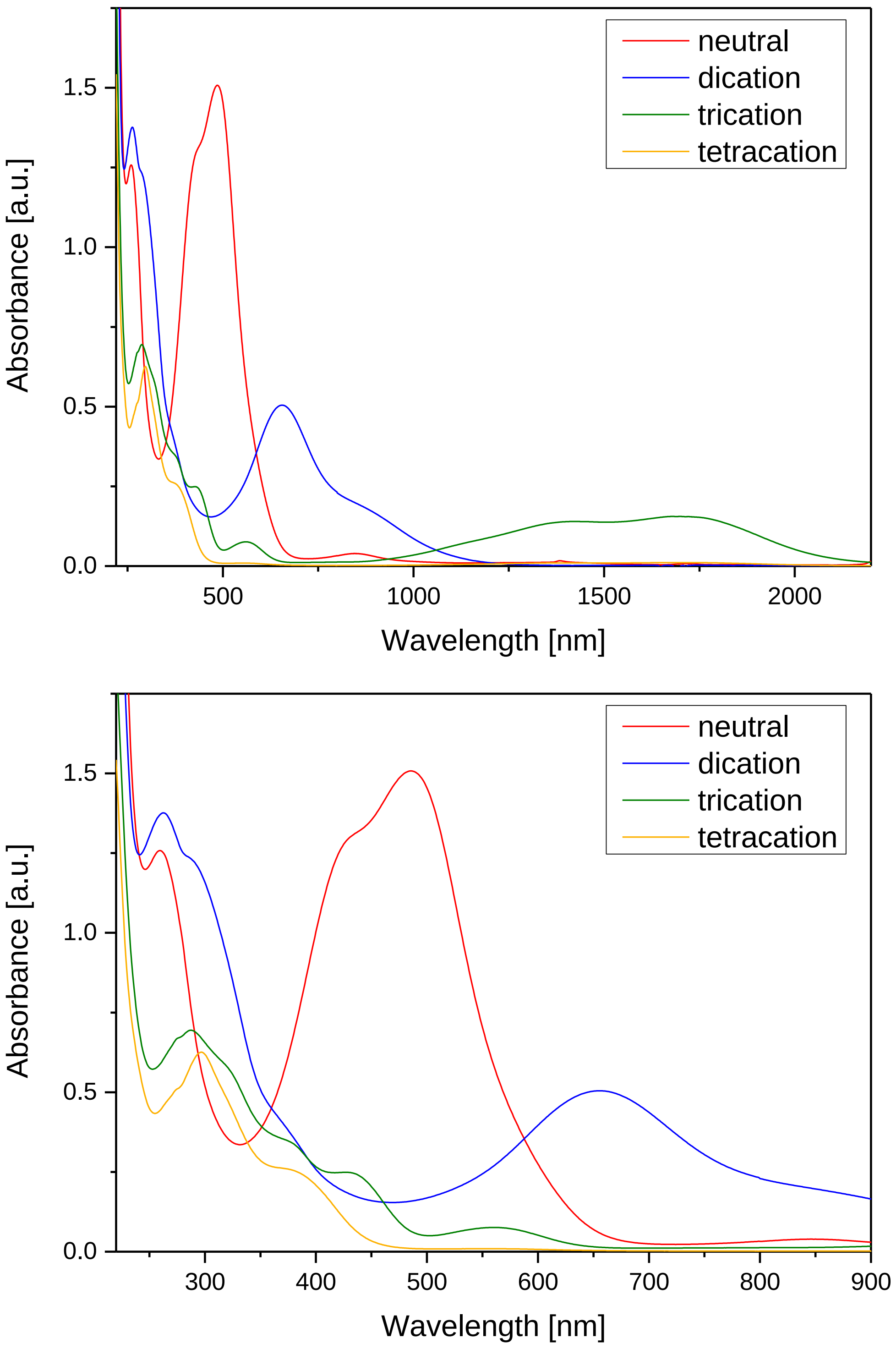

Figure S101: UV-vis-NIR spectra of **2b** (red), **2b^{2+}** (blue), **2b^{3+}** (green), **2b^{4+}** (yellow) in thf or CH_2Cl_2 (**2b^{2+}**) at c = 0.5 mg mL^{-1}, 0.1 cm quartz cuvette.

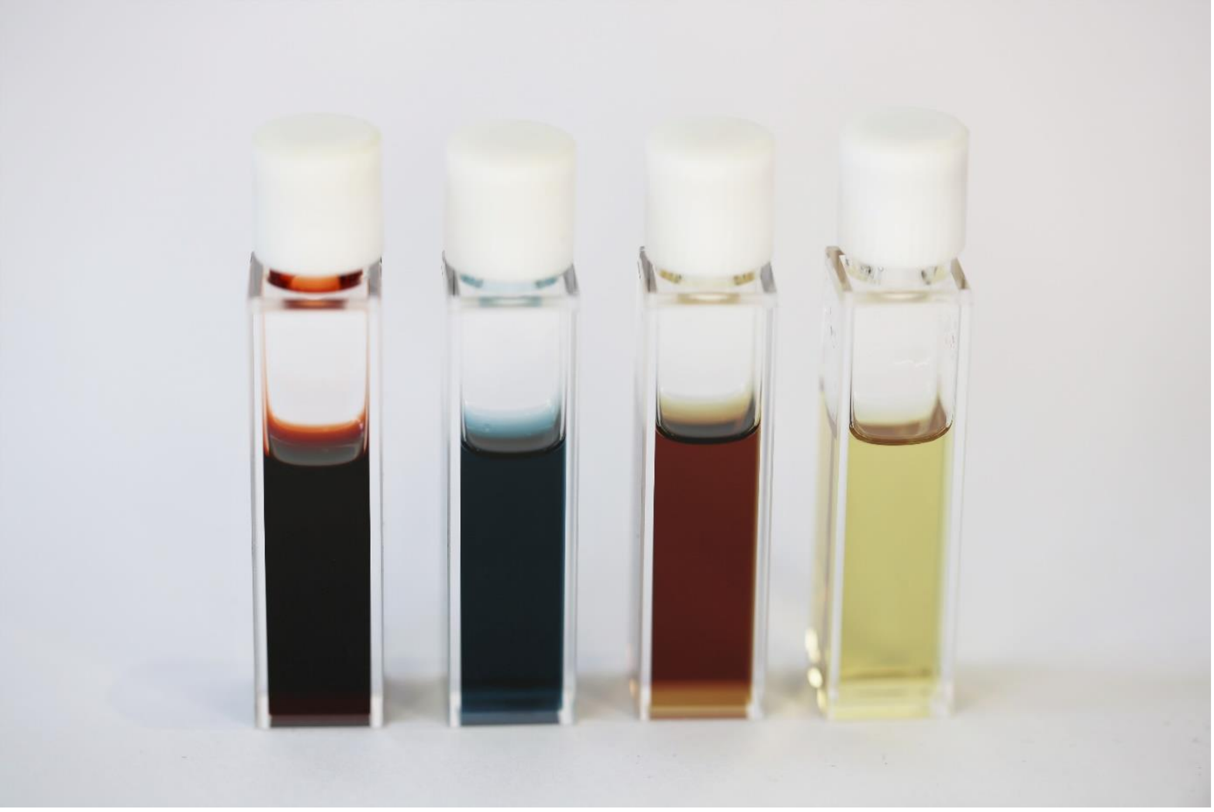

Figure S102. Photograph of the isolated oxidation states of the **2b** redox-system (increasing oxidation from left to right), in 1.0 cm quartz cuvettes.

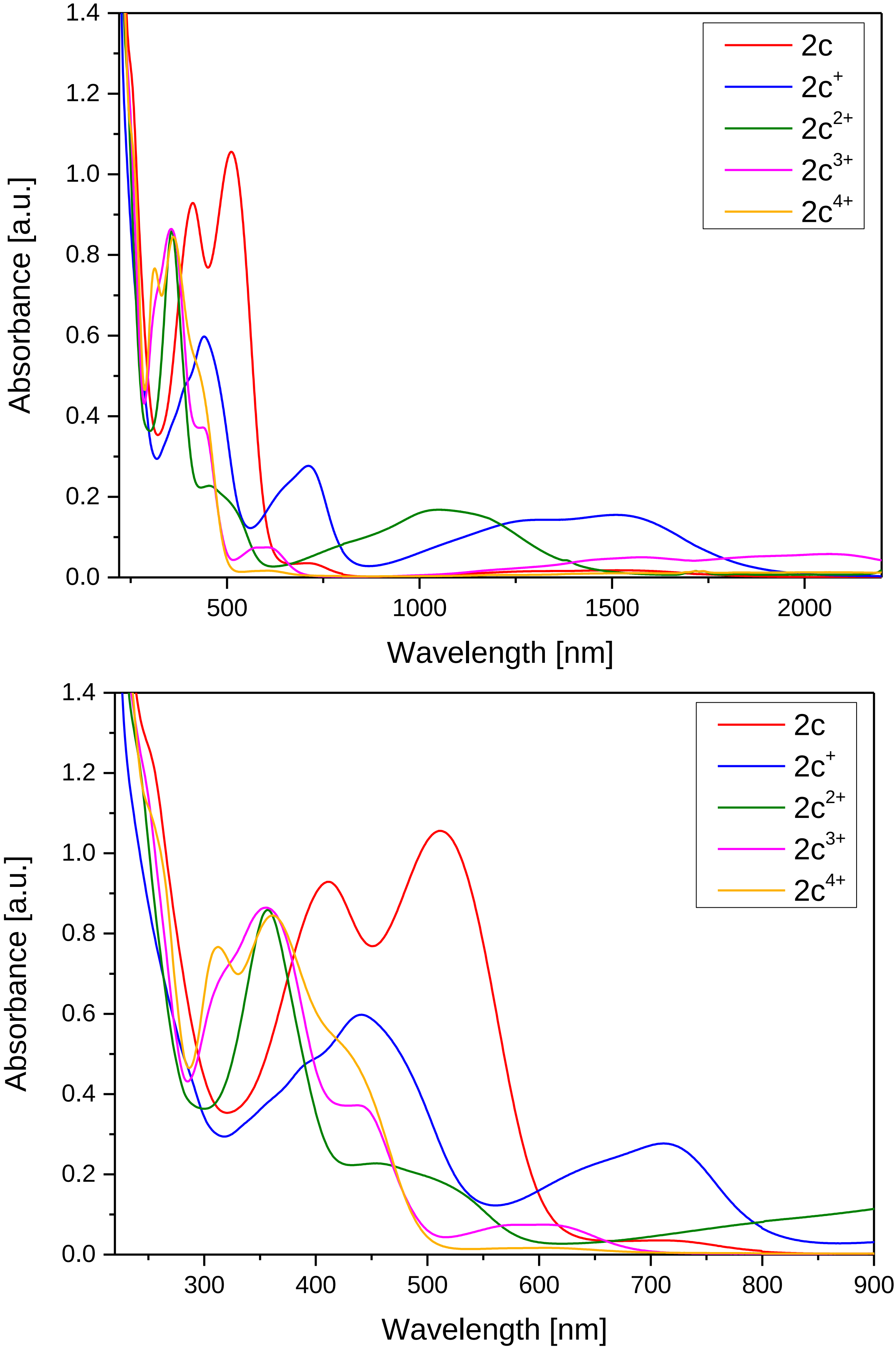

Figure S103: UV-vis-NIR spectra of **2c** (red), **2c^{+}** (blue), **2c^{2+}** (green), **2c^{3+}** (magenta), **2c^{4+}** (yellow) in thf, 0.1 cm quartz cuvette.

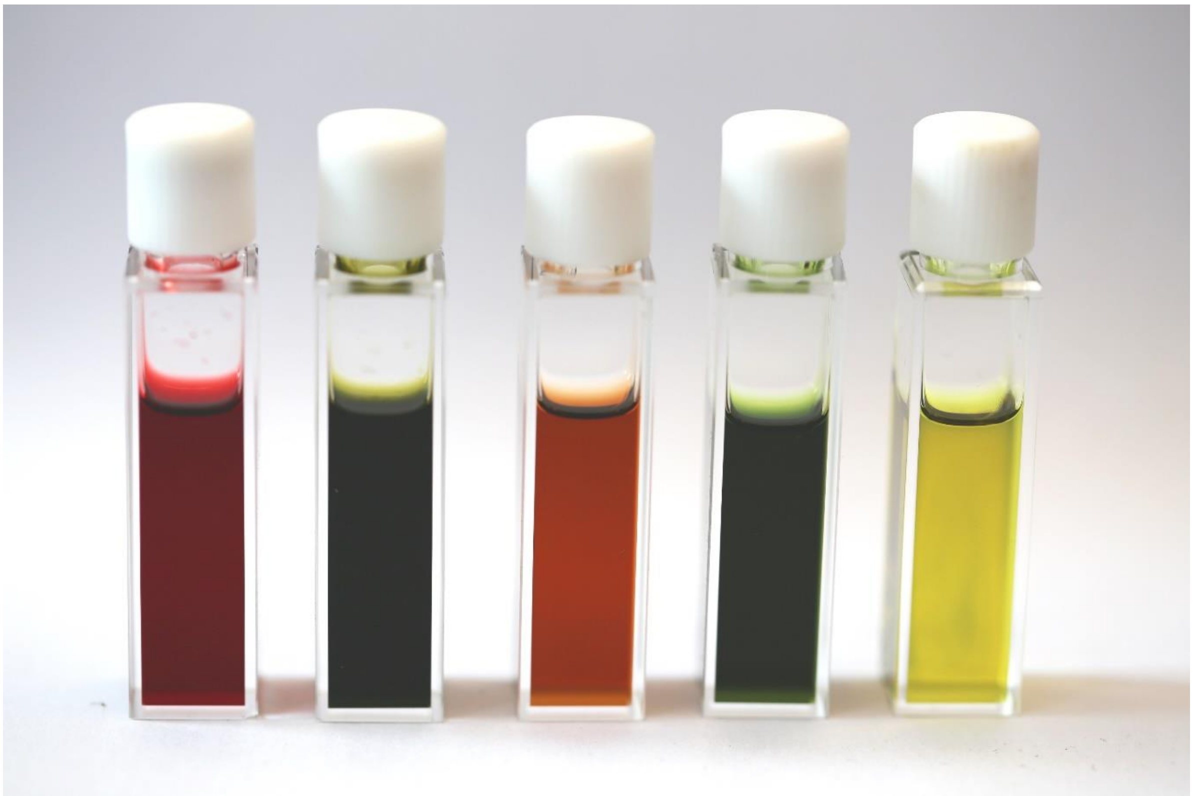

Figure S104: Photograph of the isolated oxidation states of the **2c** redox-system (increasing oxidation from left to right), in 1.0 cm quartz cuvettes.

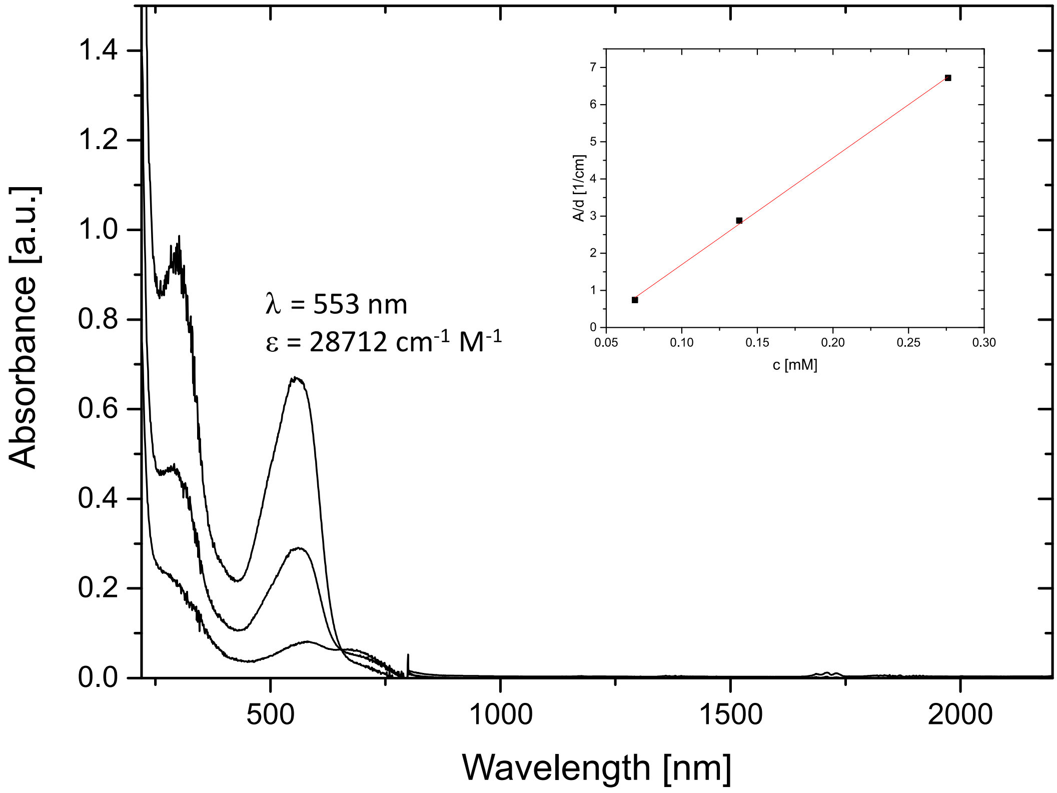

Figure S105: UV-vis-NIR spectra of **2a** in thf, measured 0.1 cm quartz cuvettes.

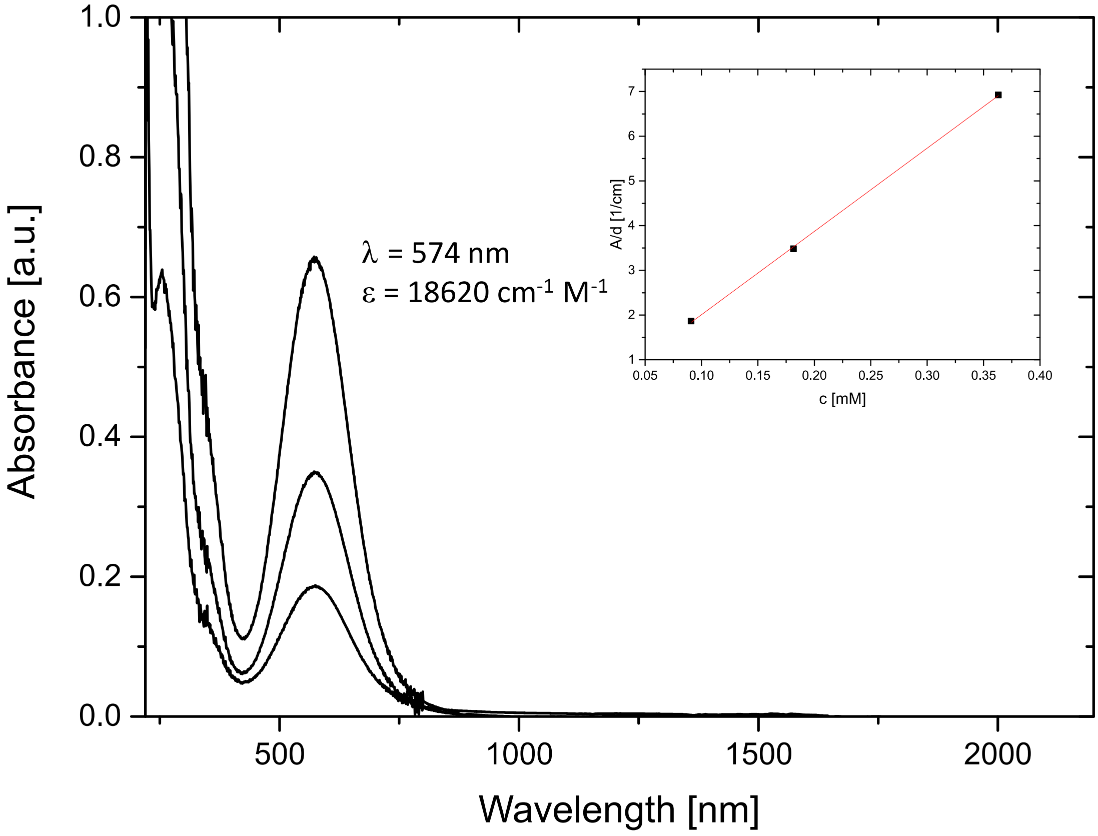

Figure S106: UV-vis-NIR spectra of **2a**$^{2+}$ in CH_2Cl_2, measured 0.1 cm quartz cuvettes.

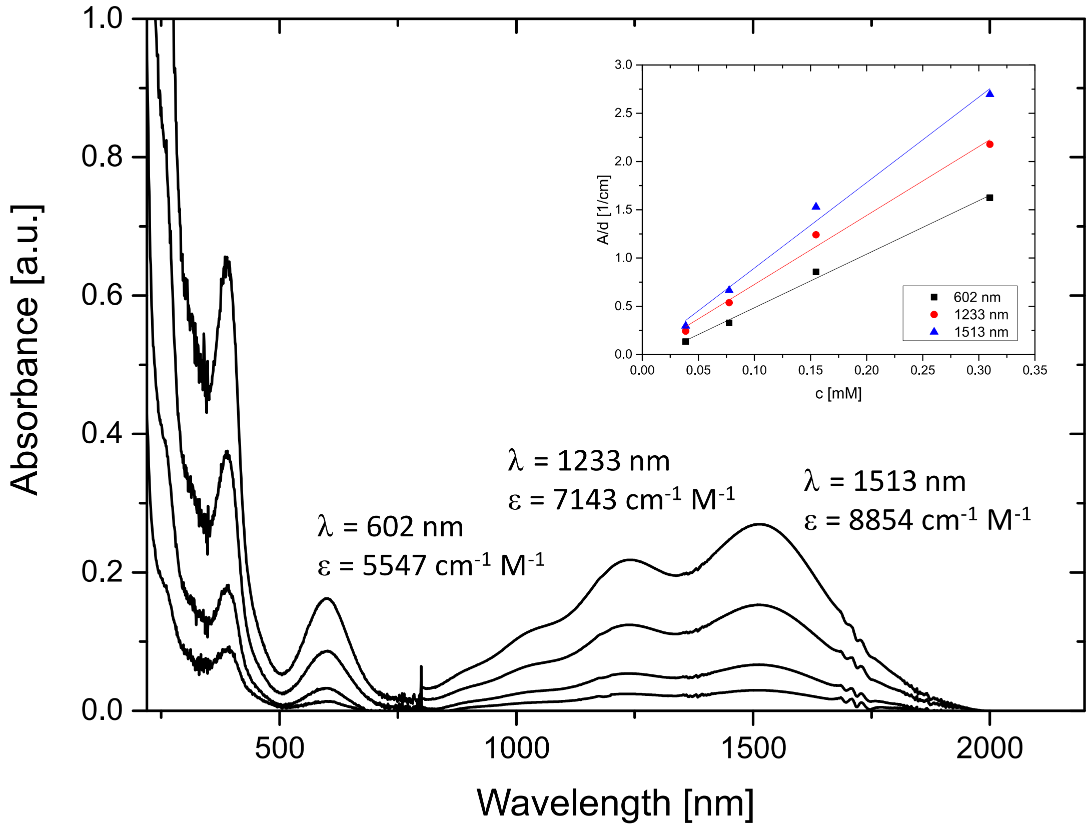

Figure S107: UV-vis-NIR spectra of **2a**$^{3+}$ in thf, measured 0.1 cm quartz cuvettes.

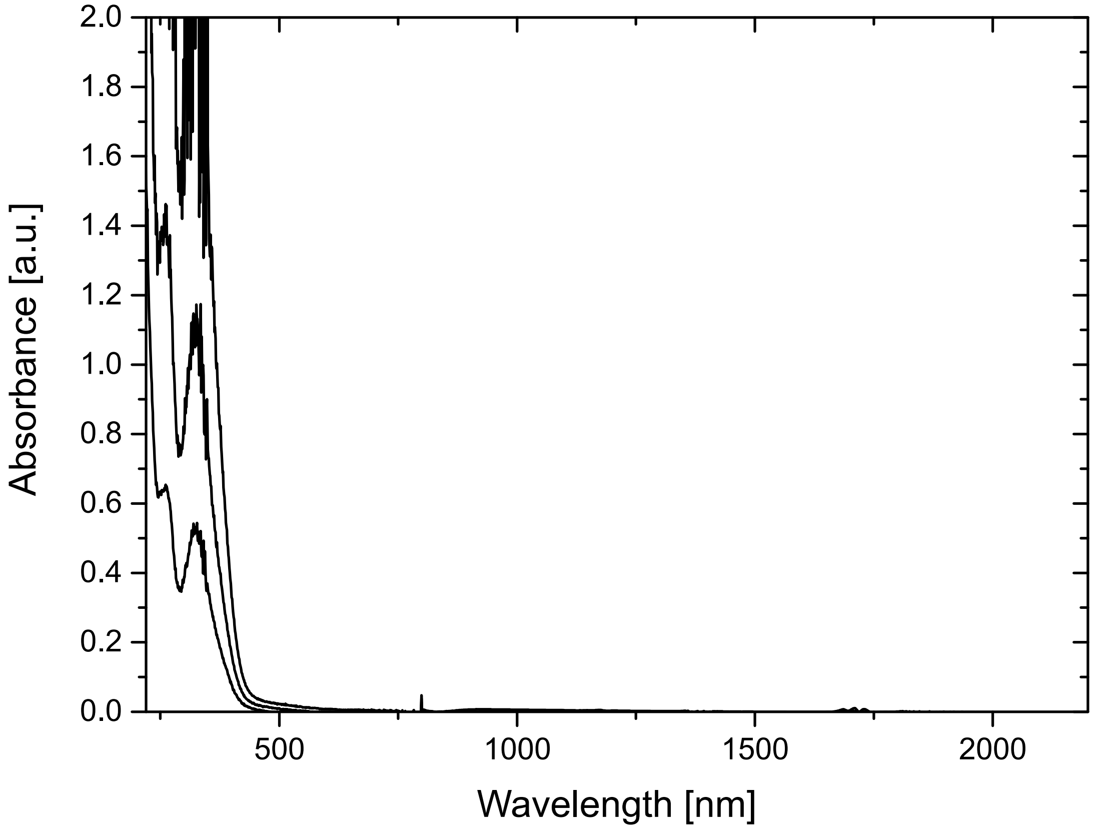

Figure S108: UV-vis-NIR spectra of **2a**$^{4+}$ in thf, measured 0.1 cm quartz cuvettes.

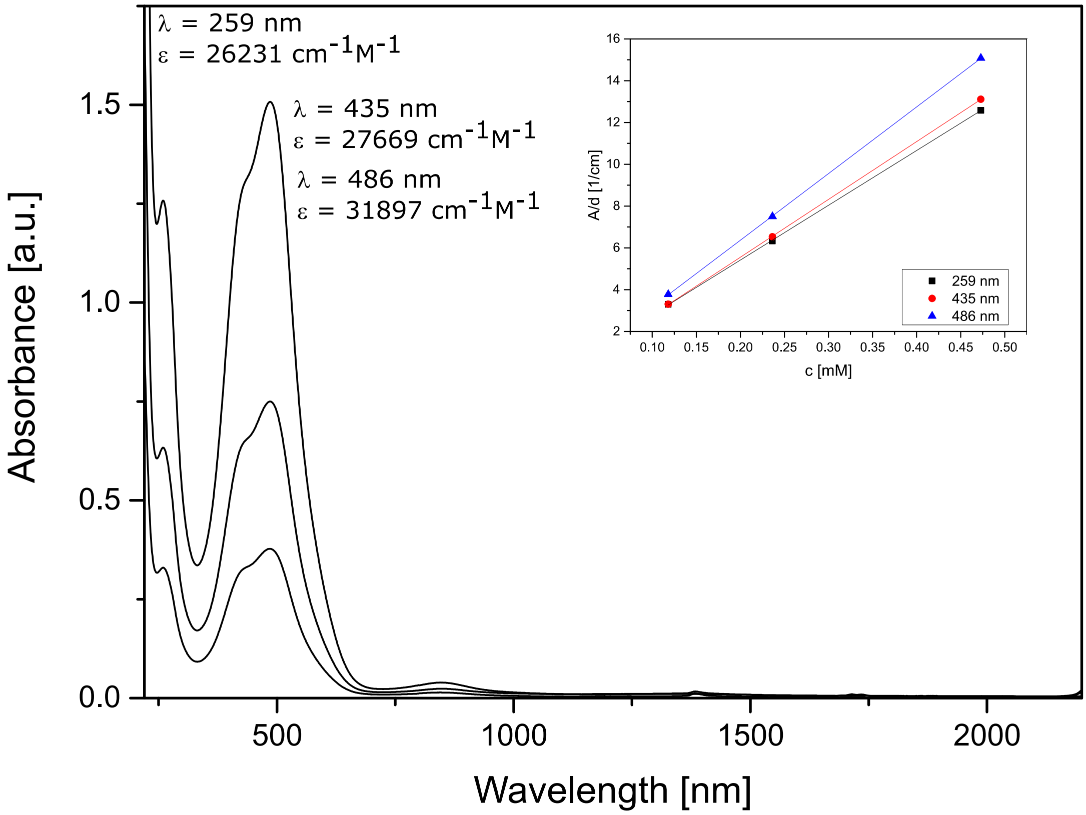

Figure S109: UV-vis-NIR spectra of **2b** in thf, measured 0.1 cm quartz cuvettes.

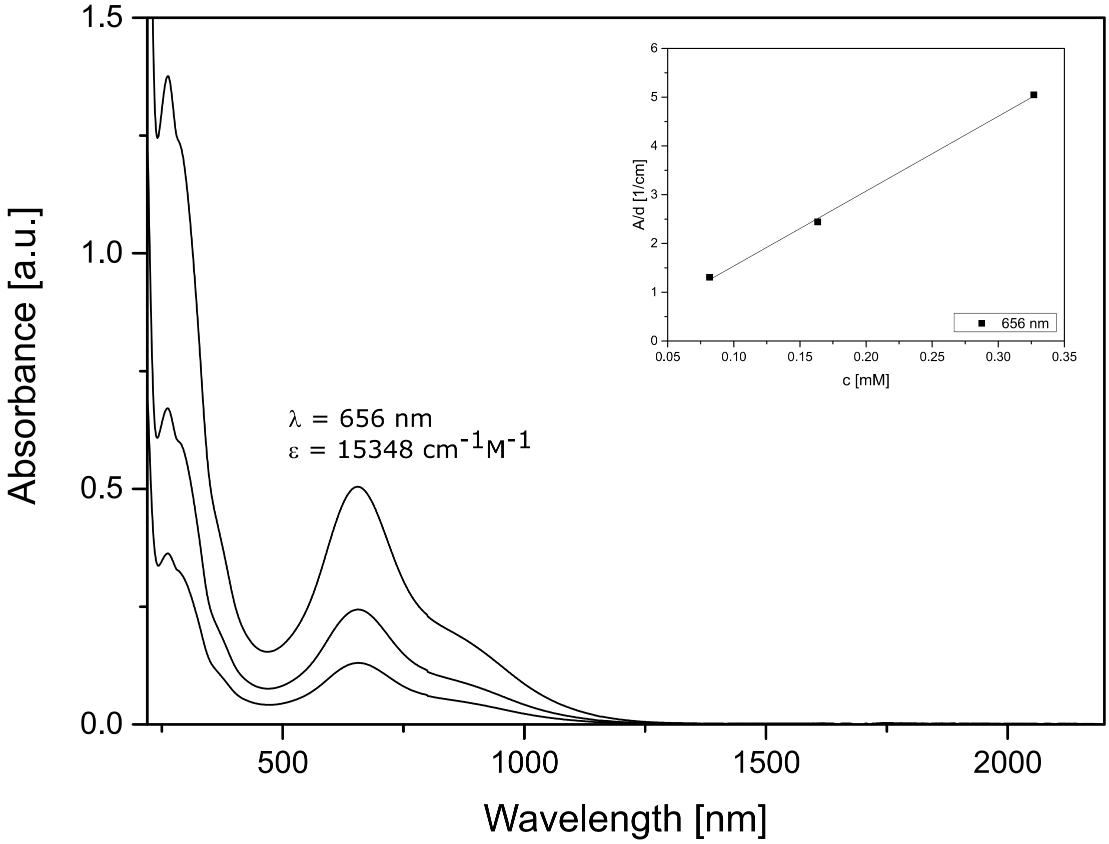

Figure S110: UV-vis-NIR spectra of **2b**$^{2+}$ in thf, measured 0.1 cm quartz cuvettes.

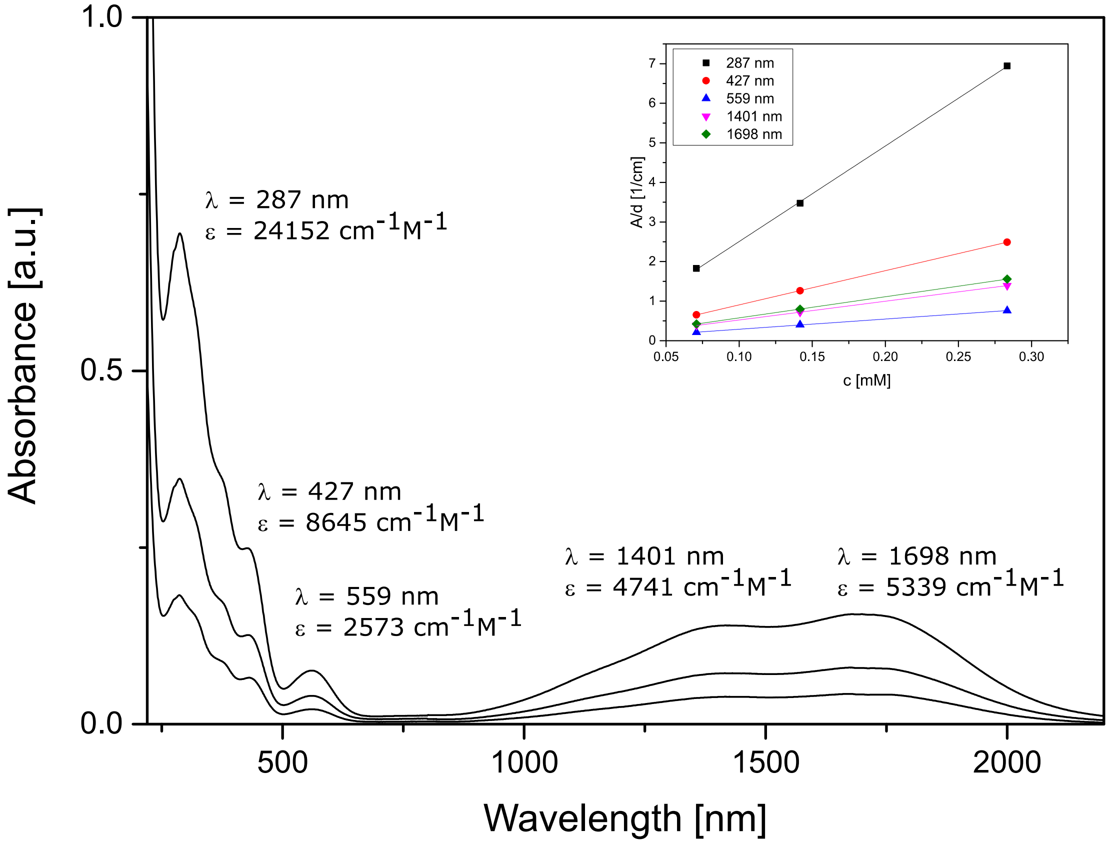

Figure S111: UV-vis-NIR spectra of **2b**$^{3+}$ in thf, measured 0.1 cm quartz cuvettes.

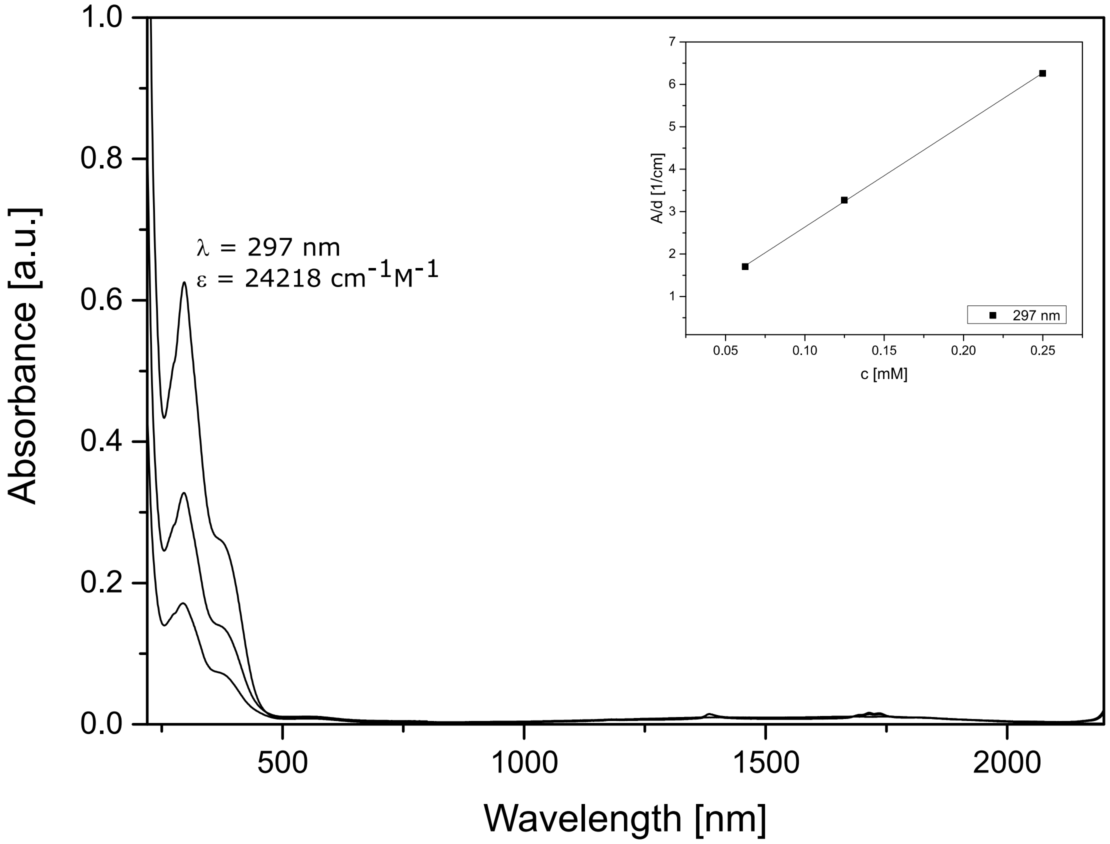

Figure S112: UV-vis-NIR spectra of **2b**$^{4+}$ in thf, measured 0.1 cm quartz cuvettes.

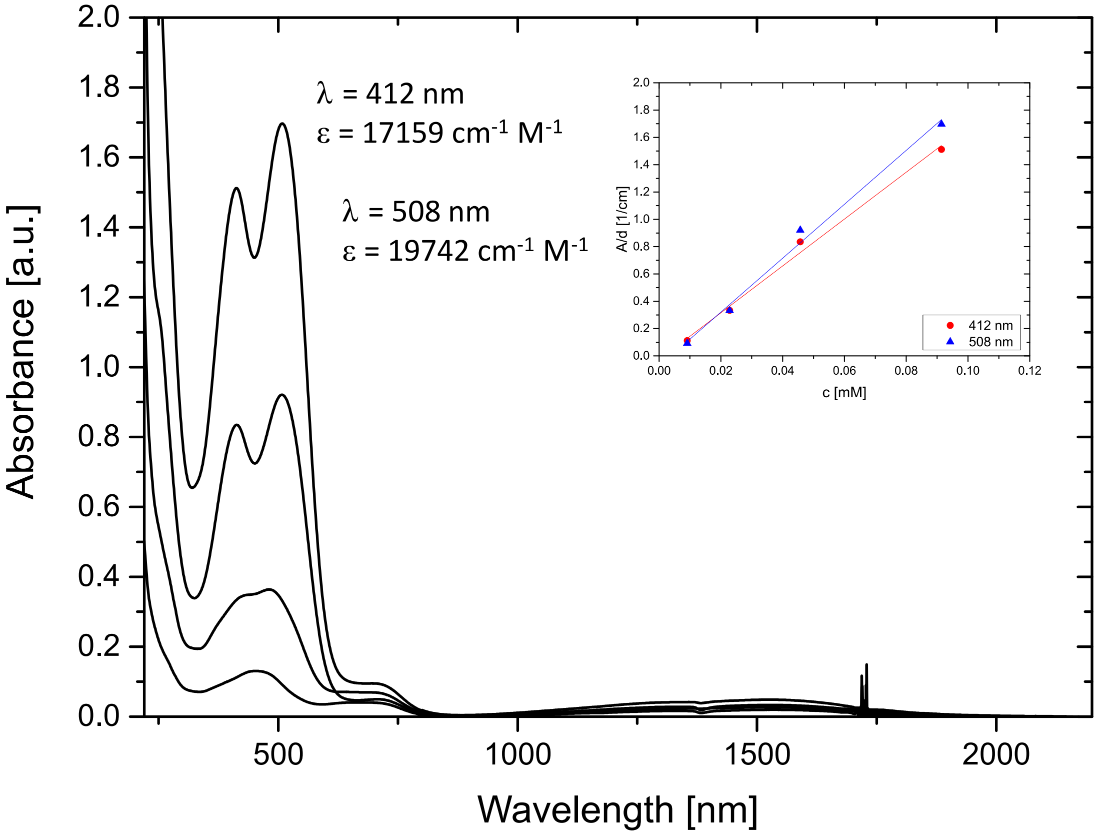

Figure S113: UV-vis-NIR spectra of **2c** in thf, measured 1.0 cm quartz cuvettes.

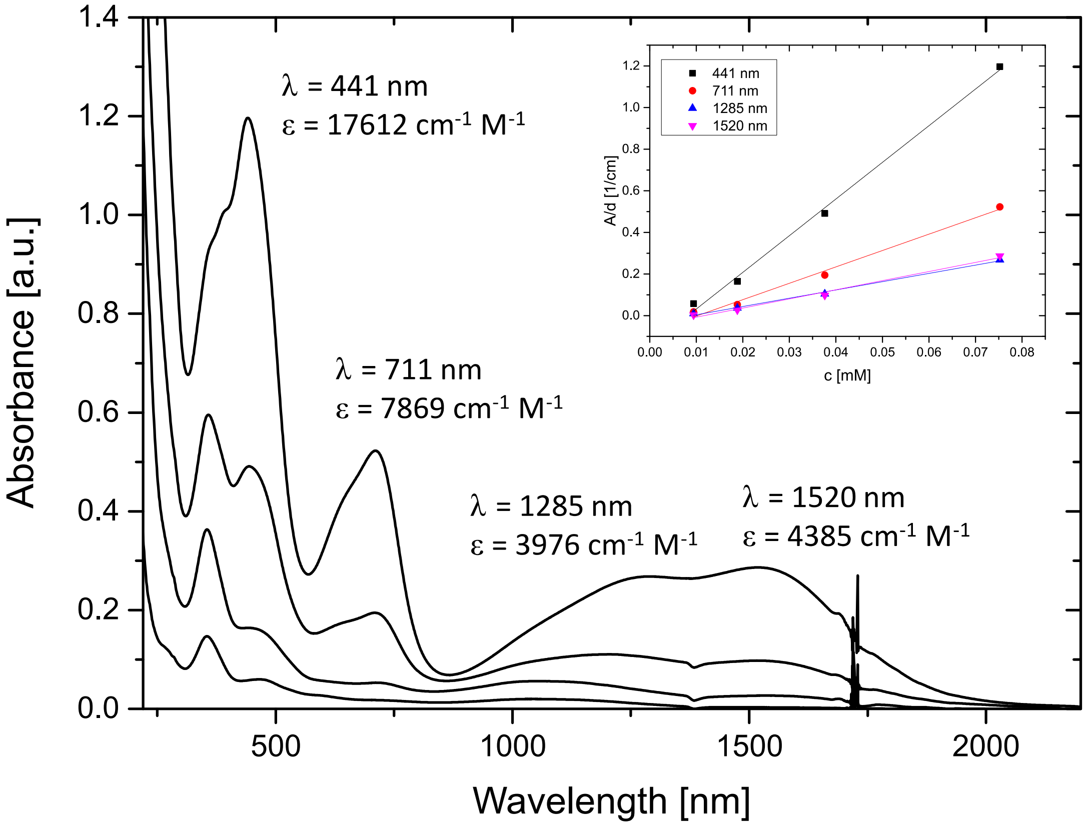

Figure S114: UV-vis-NIR spectra of **2c⁺** in thf, measured 1.0 cm quartz cuvettes.

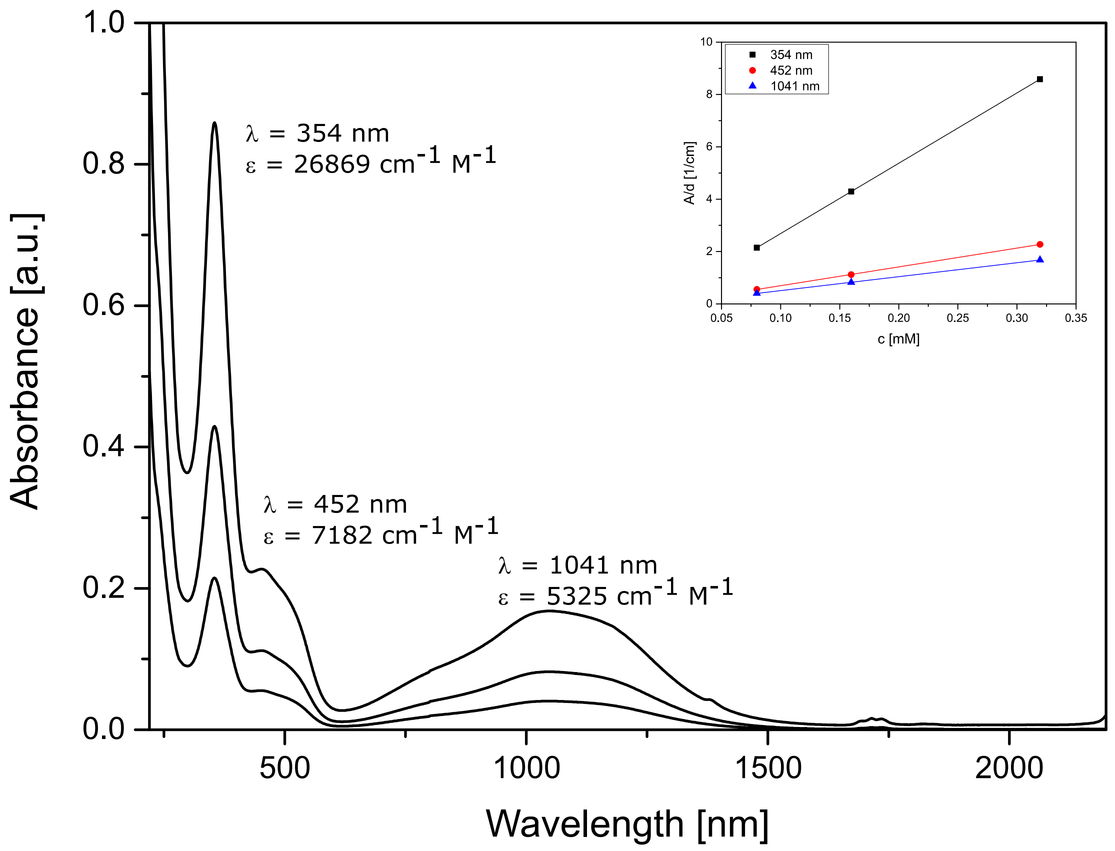

Figure S115: UV-vis-NIR spectra of **$2c^{2+}$** in thf, measured 0.1 cm quartz cuvettes.

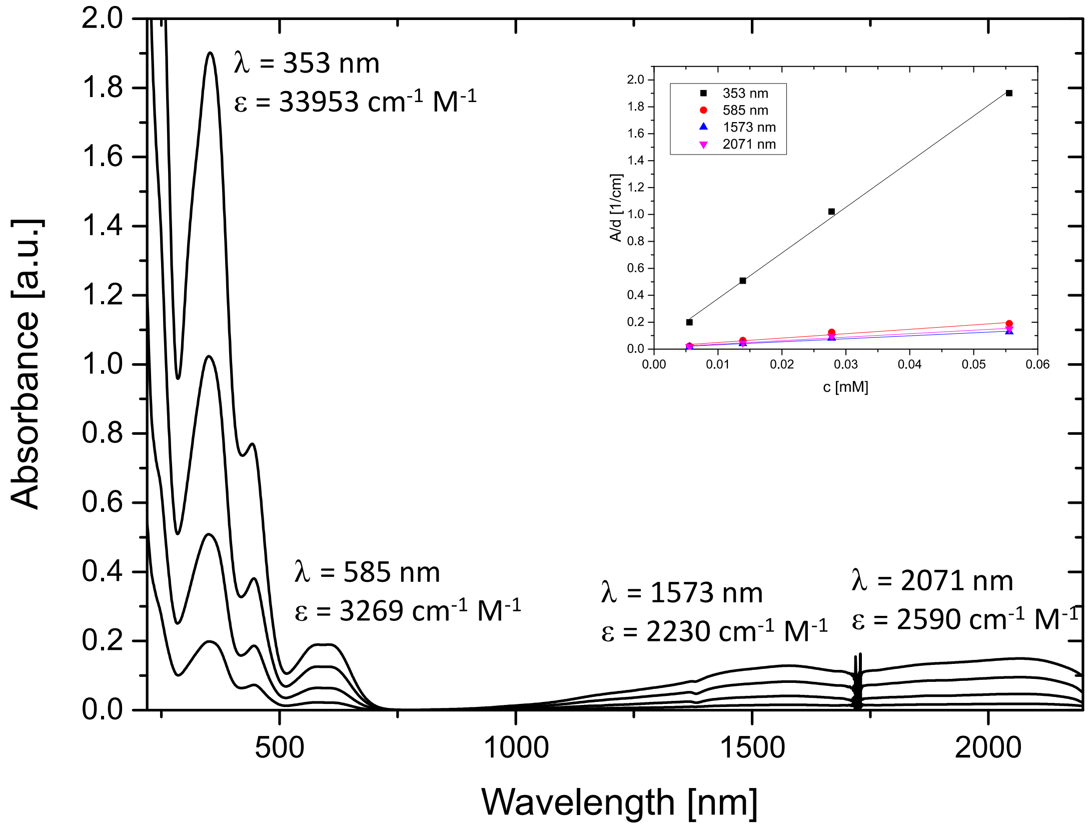

Figure S116: UV-vis-NIR spectra of **$2c^{3+}$** in thf, measured 1.0 cm quartz cuvettes.

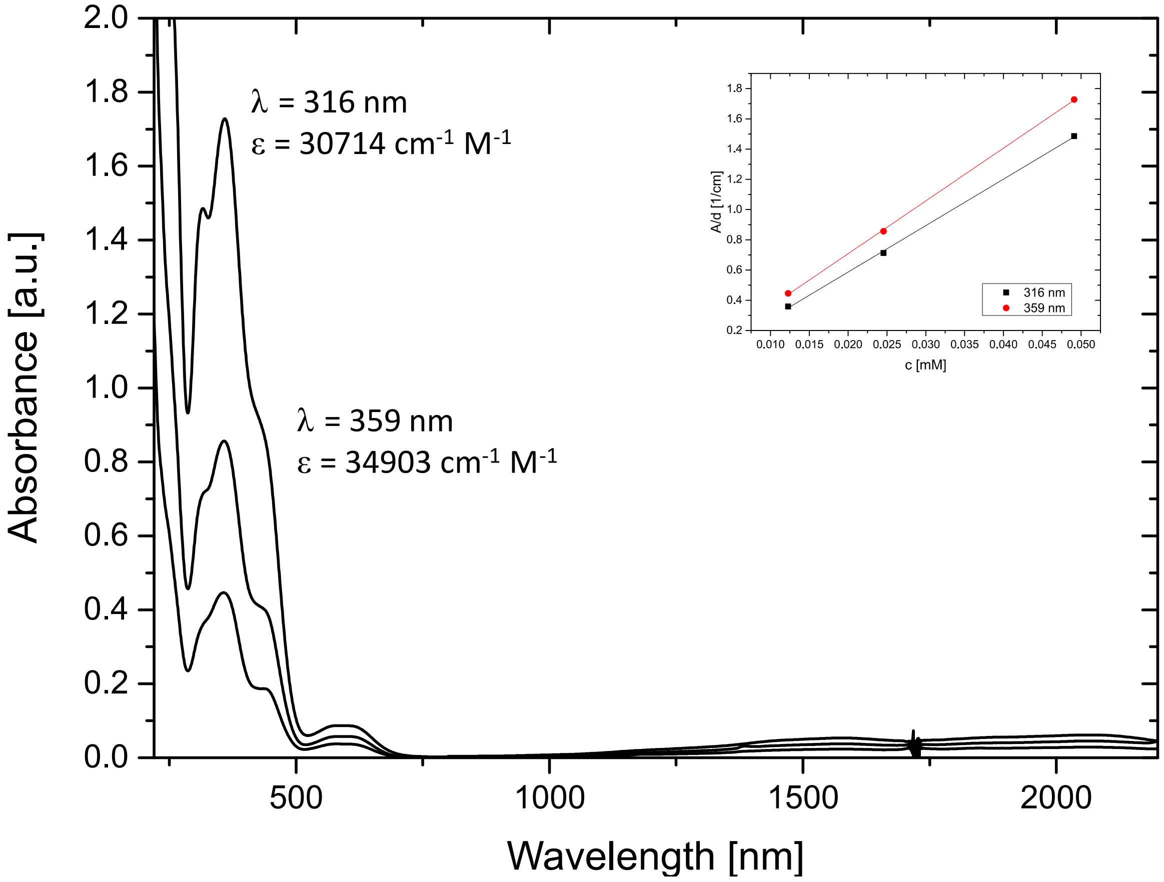

Figure S117: UV-vis-NIR spectra of **2c**$^{4+}$ in thf, measured 1.0 cm quartz cuvettes. This oxidation state decomposes rather quickly in thf (polymerization).

9. UV-vis-NIR spectroelectrochemistry

Electrochemical measurements were recorded with a Gamry Instruments Reference 600+. The samples were measured starting from the neutral compounds, containing a three electrode setup (platinum wire, platinum net and Ag/$AgNO_3$ reference electrode) in a UV-vis cell (1 mm diameter from ALS Co., Ltd; SEC-C) under nitrogen (Fig. S118).

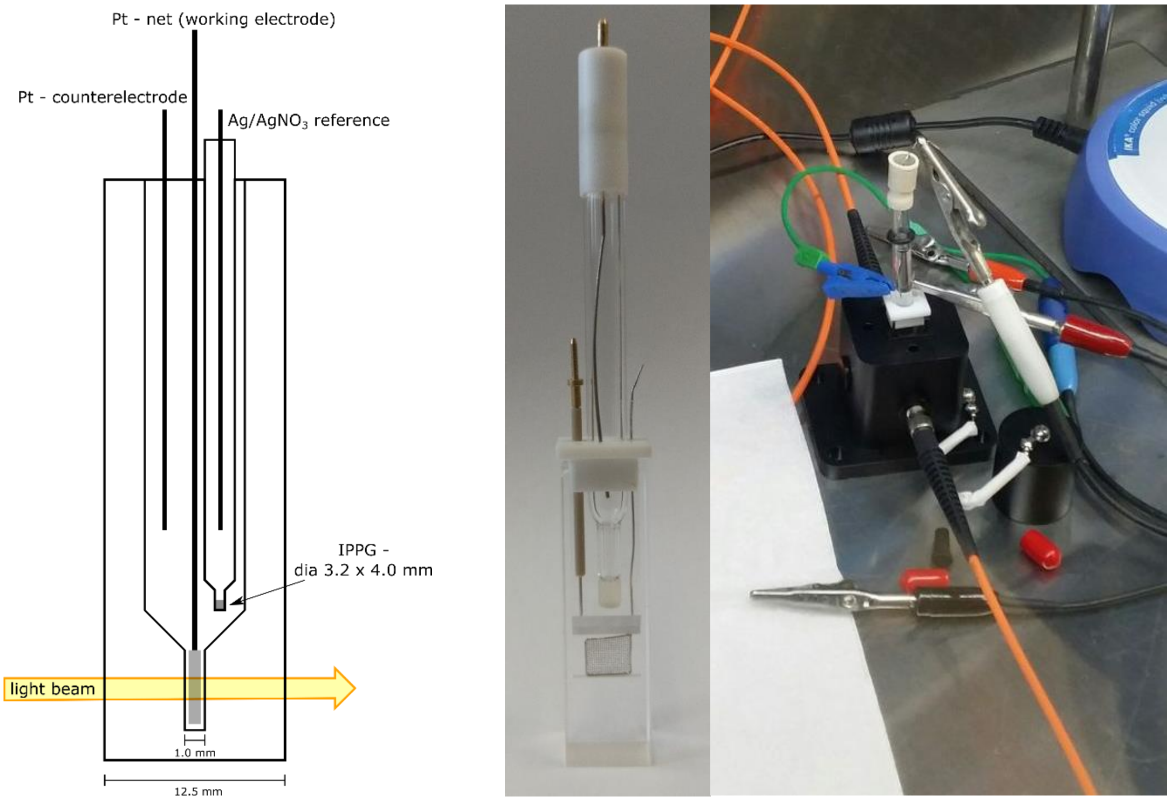

Figure S118. Experimental setup for the spectroelectrochemistry. Schematic representation of the UV-Vis cell from the side showing the light beam transmission (left); photograph of the assembled UV-Vis cell, front view (middle) and connected cell in the glovebox (right).

In order to guarantee a clean oxygen free setup, the measurements were performed in a nitrogen filled glove box. The reference electrode was freshly prepared by using a fritted sample holder, which was activated as described for the sample holder preparation for cyclic voltammetry. To the fritted sample holder was added a freshly prepared 0.01 M $AgNO_3$/ 0.1 M *n*-Bu_4NPF_6 solution in CH_3CN and a silver wire. The light source was a deuterium/tungsten light source and light detected with a BWTEK ExemplarLS. A blank spectrum and a reference spectrum with just solvent was taken in advance and was subtracted from the measured data.

The following two procedures were used:

Procedure 1 (for **2a** and **2b**): A UV-VIS spectrum was taken every 10 seconds, while the potential was modified using multiple step controlled potential coulometry for the given potentials and times: (The respective redox potentials were determined beforehand by cyclic voltammetry):

For **2a:** Starting potential (vs. Ag^0/Ag^+): -2.0 V (20 s), first step: -0.75V (300s), second step: -0.34V (600s), third step +0.5V (600s). (Fig. S119)

For **2b:** Starting potential (vs. Ag^0/Ag^+): -2.0 V (20 s), first step: -1.25 V (800 s), second step -0.7 V (800 s). (Fig. S121)

Procedure 2 [for **2b** (Fig. S120) and **2c** (Fig. S122)]: A UV-vis spectrum was measured every 10 seconds, while the potential was scanned from negative potentials (-2.0 V) to positive potentials (+ 1.0 V) by a step size of 2 - 2.5 mV/s.

For the visualization of the data not every spectrum but selected are shown in order to reduce the data amount.

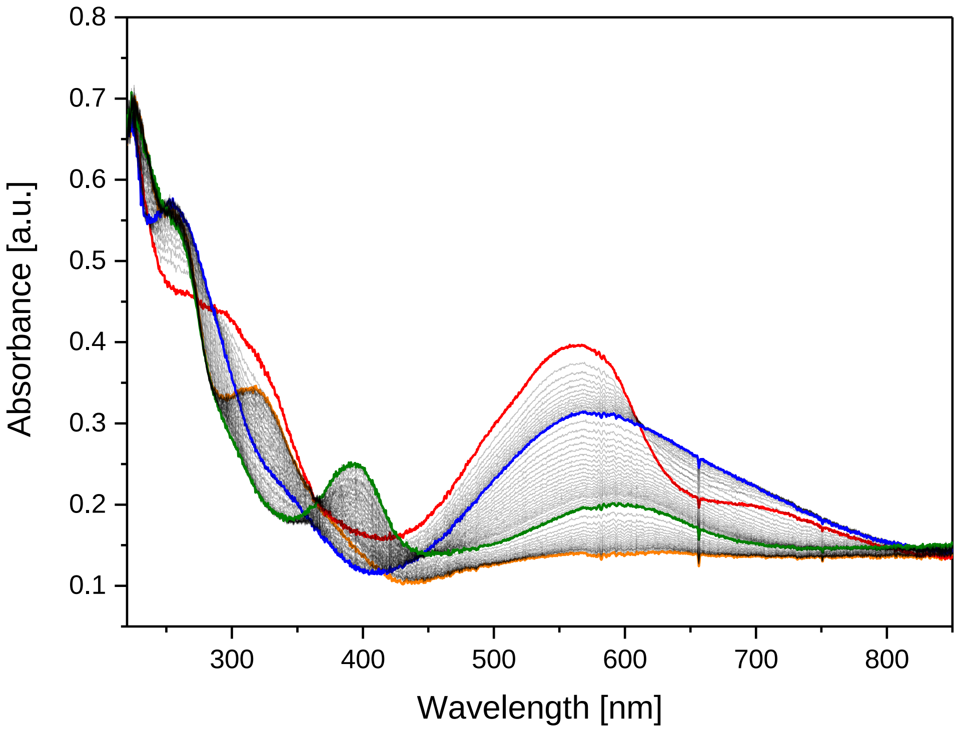

Figure S119. UV-vis-SEC of **2a** (procedure 1). Color coding: **2a** (red), **2a^{2+}** (blue), **2a^{3+}** (green), **2a^{4+}** (yellow).

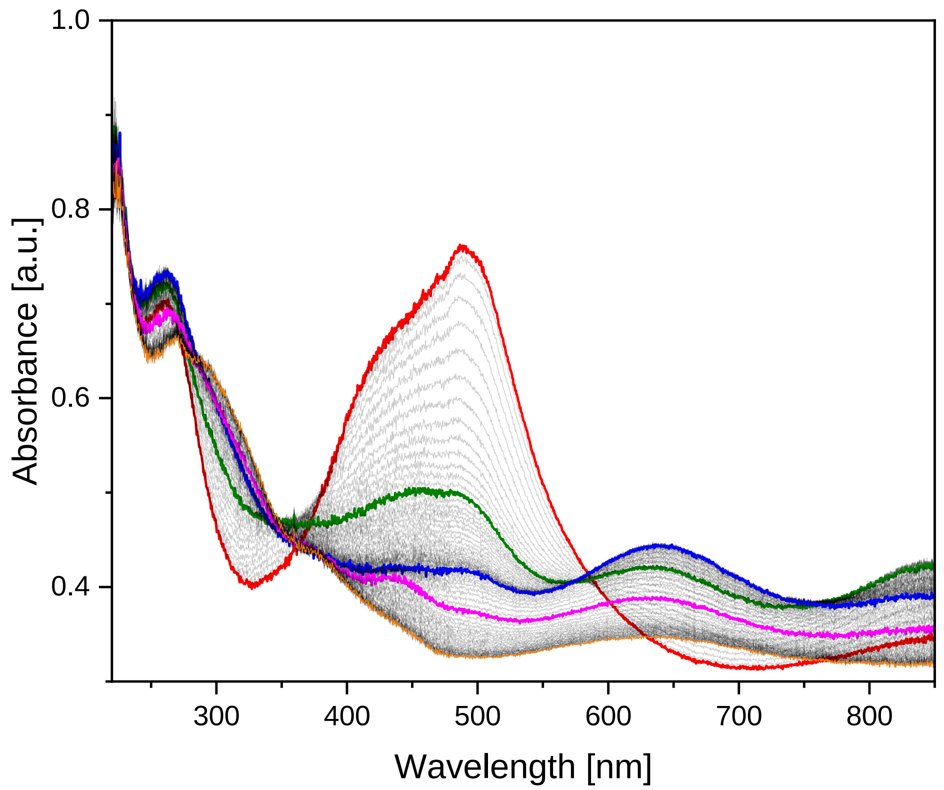

Figure S120. UV-vis-SEC of **2b** (procedure 2). Color coding: **2b** (red), mixture of $\mathbf{2b^{+}}$ and $\mathbf{2b^{2+}}$ (green), dication $\mathbf{2b^{2+}}$ (blue), $\mathbf{2b^{3+}}$ (magenta) and $\mathbf{2b^{4+}}$ orange.

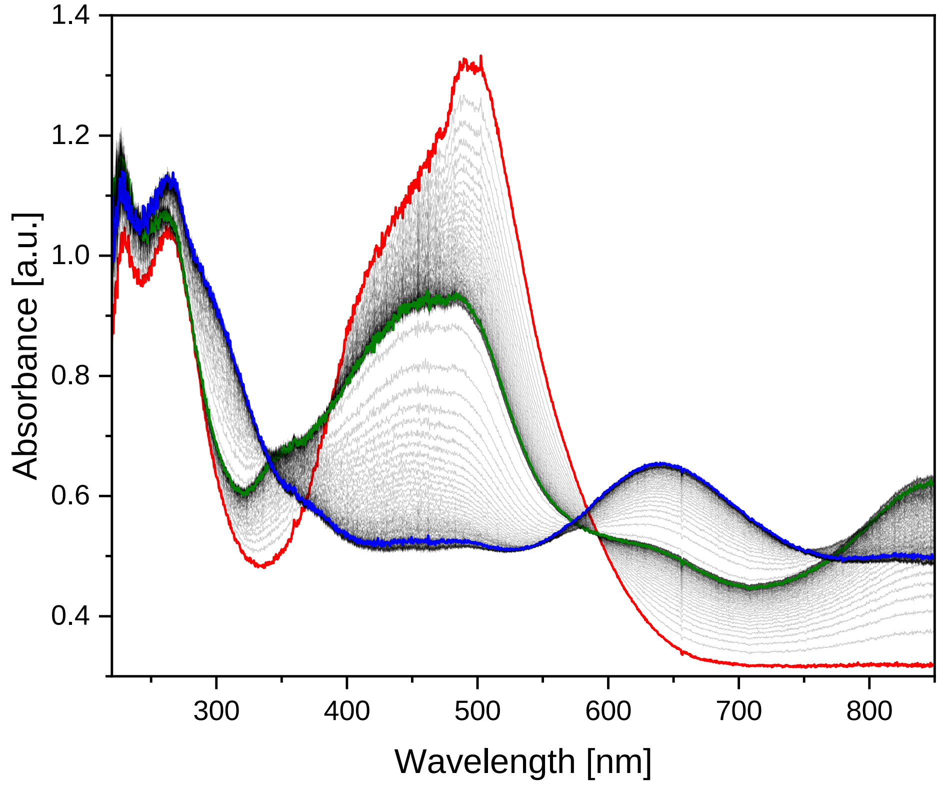

Figure S121. UV-vis-SEC of **2b** (procedure 1: two potential steps: -2 V to -1.25 V to -0.7 V). Color coding: **2b** (red), mixture of $\mathbf{2b^{+}}$ and $\mathbf{2b^{2+}}$ (green), dication $\mathbf{2b^{2+}}$ (blue). The strongly increased absorption at ~ 900 nm points to the formation of the monocation in equilibrium with the neutral and dicationic oxidation states.

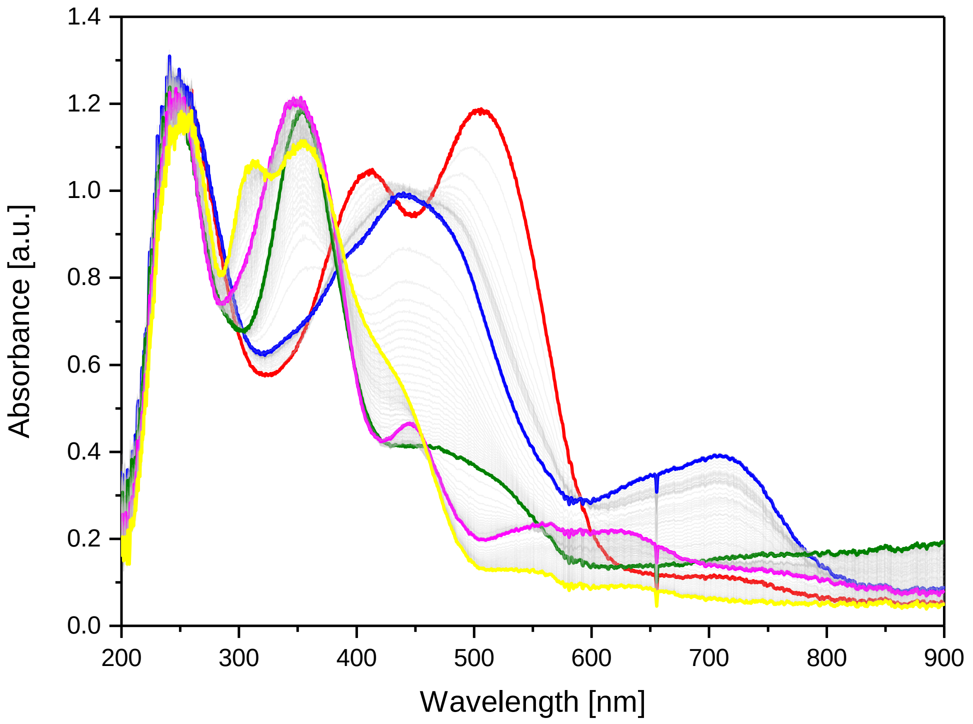

Figure S122: UV-vis-SEC of **2c** (procedure 2). Color coding: **2c** (red), **2c^{+}** (blue), **2c^{2+}** (green), **2c^{3+}** (magenta), **2c^{4+}** (yellow).

10. Solubility and stability measurements

Solubility Measurements

To estimate the solubilities of **2a^0** - **2a^{4+}** as well as **2c^0**, **2c$^+$** and **2c^{2+}**, 2 mL solvent were added to 40 mg substance. The mixture was vigorously stirred for 5 min and filtrated through a PFTE syringe filter. 1 mL was transferred in a tared vial. After carefully evaporating the solvent under reduced pressure the mass of the vial was determined again. This procedure was repeated to validate the results.

Table S3. Solubility of the oxidation states of **2a** in MeCN.

compound	solubility in MeCN [m]	solubility in MeCN [c]
2a	Below detection limit	-/-
2a^{2+} *2 SbF_6^-*	> 19.9 mg	> 15 mM
2a^{3+} *3 SbF_6^-*	> 19.9 mg	> 12 mM
2a^{4+} *4 SbF_6^-*	> 19.9 mg	> 11 mM

Table S4. Solubility of the oxidation states of **2c** in MeCN.

compound	solubility in MeCN [m]	solubility in MeCN [c]
2c	3.2 mg	2.9 mM
2c$^+$ *SbF_6^-*	> 19.9 mg	> 15 mM
2c^{2+} *2 SbF_6^-*	> 18.8 mg	> 12 mM

While being insoluble in MeCN the solubility of **2a** in dmf (0.8 mg/mL, 0.9 mM) and thf (> 19.9 mg/mL, > 22 mM) is significantly higher.

Stability measurements

To estimate the stability of closed shell compounds a solution in deuterated solvent was prepared, a ^{1}H NMR spectrum measured in a J-Young NMR tube, the compound transferred into a screw cap vial inside a nitrogen filled glove box at room temperature and stored over a variable time period before a second ^{1}H NMR spectrum of the same sample was measured.

For open shell compounds a solution of 1 mg/mL in MeCN was prepared and stored in a brown glass vial in a nitrogen filled glove box at 25 °C. UV-Vis spectra of aliquots of the solution were measured in the indicated time intervals.

Data for **2c$^+$** and **2c^{3+}** are shown in the manuscript.

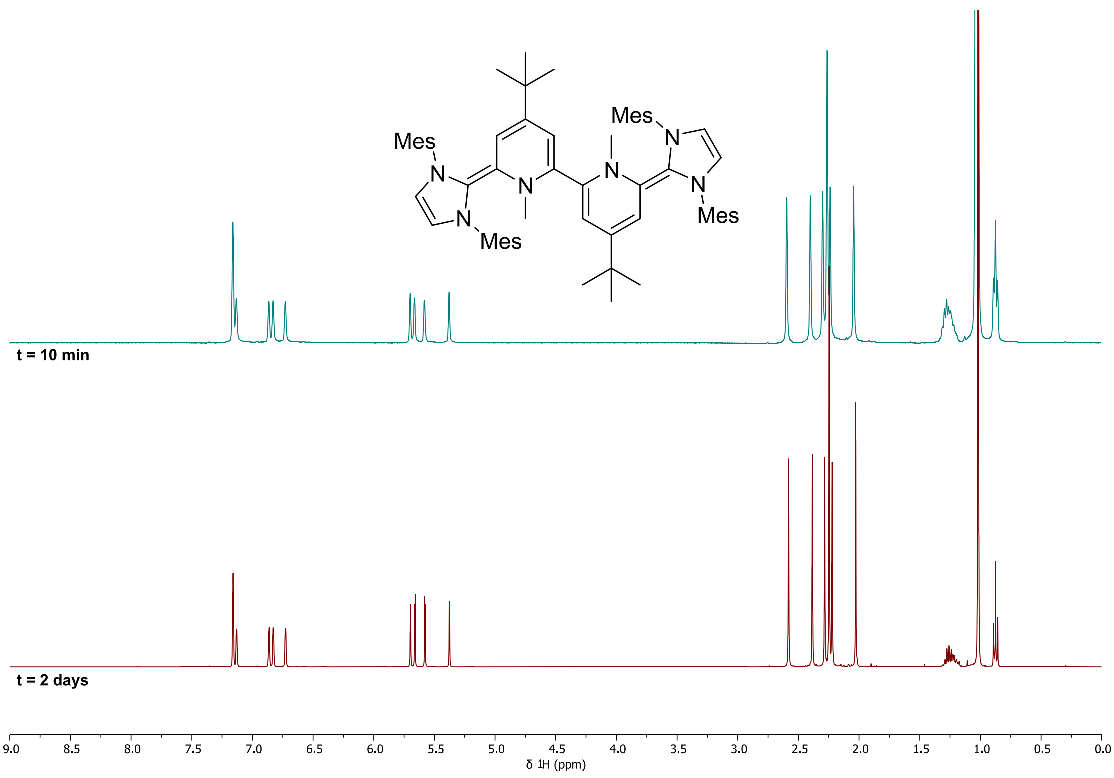

Figure S123. Comparison of ^{1}H NMR spectra of **2a** after 10 min (top, 400 MHz, C_6D_6) and of the same sample after 2 days in solution at room temperature under N_2-atmosphere (bottom, 400 MHz, C_6D_6).

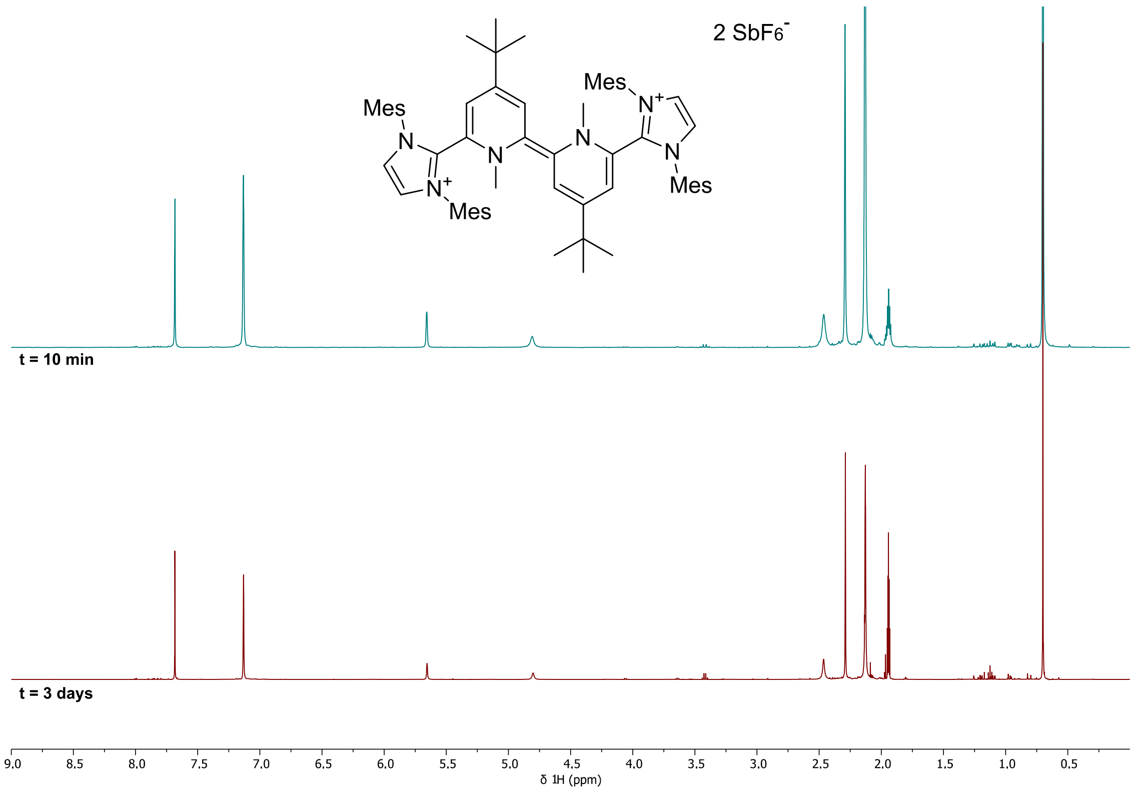

Figure S124. Comparison of ^{1}H NMR spectra of **2a^{2+}** after 10 min (top, 300 MHz, CD_3CN) and of the same sample after 3 days in solution at room temperature under N_2-atmosphere (bottom, 500 MHz, CD_3CN).

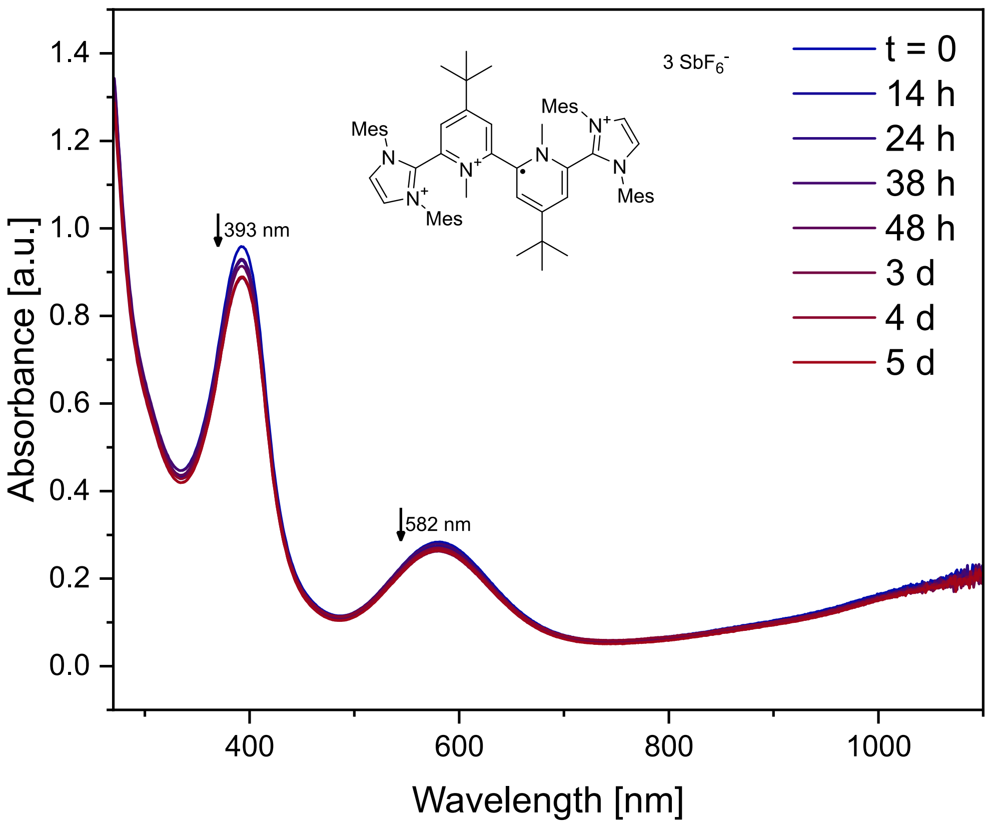

Figure S125. Comparison of UV-Vis spectra of **2a**$^{3+}$ (1 mg/ml, MeCN). The stock solution was stored in a brown glass screw cap vial under N_2-atmosphere at 25 °C.

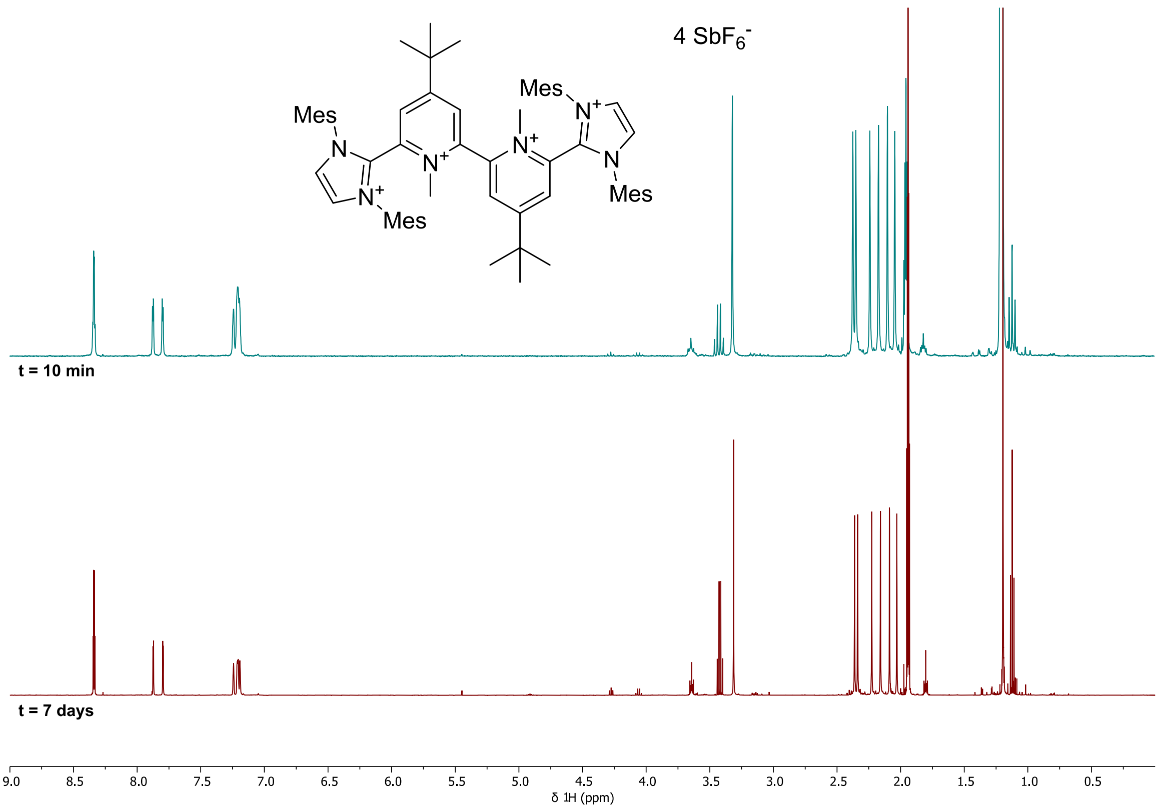

Figure S126. Comparison of ^{1}H NMR spectra of **2a**$^{4+}$ after 10 min (top, 300 MHz, CD_3CN) and of the same sample after 7 days in solution at room temperature under N_2-atmosphere (bottom, 500 MHz, CD_3CN).

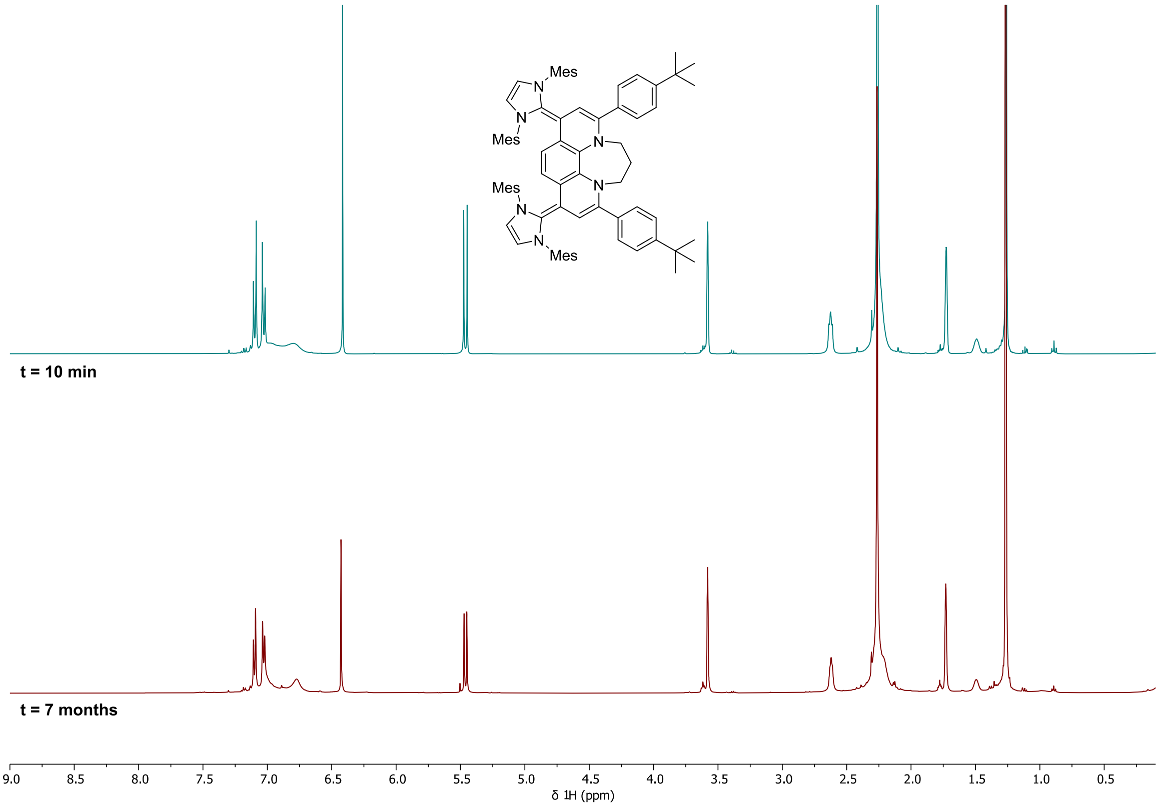

Figure S127. Comparison of ^{1}H NMR spectra of **2c** after 10 min (top, 400 MHz, d_8-THF) and of the same sample after 7 months in solution at room temperature under N_2-atmosphere (bottom, 500 MHz, d_8-THF).

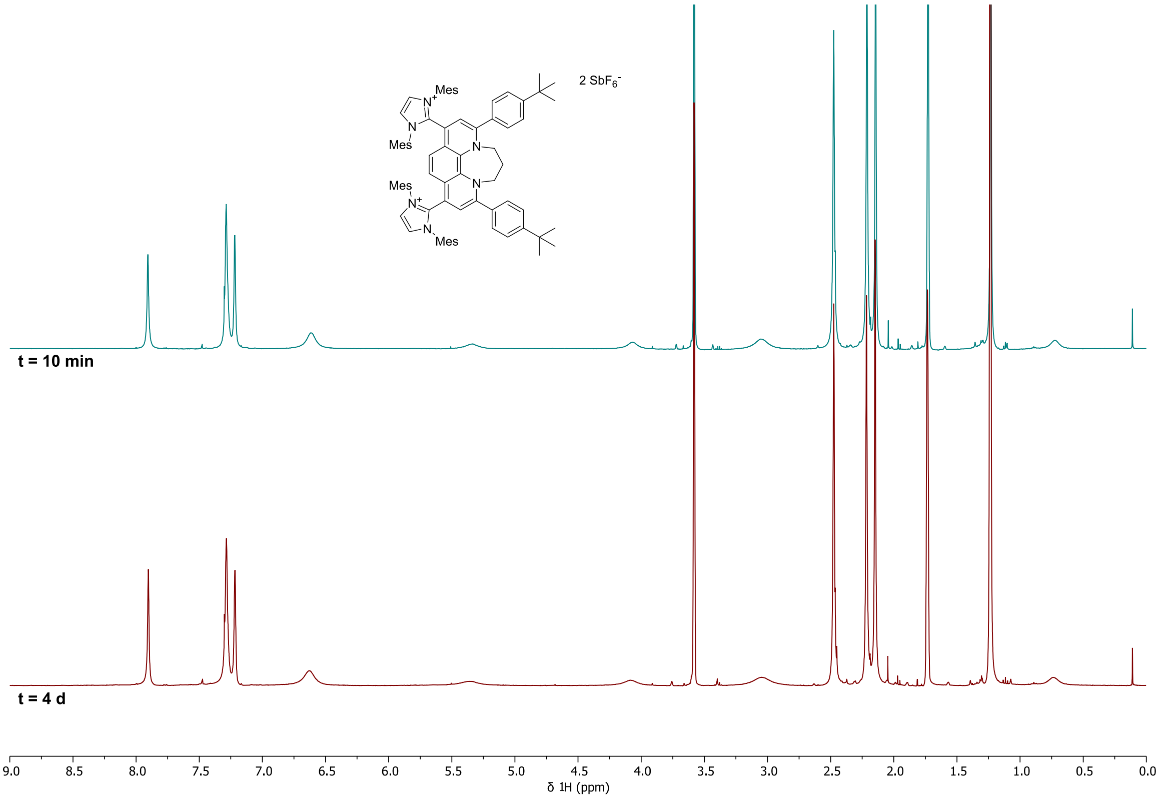

Figure S128. Comparison of ^{1}H NMR spectra of **2c^{2+}** after 10 min (top, 400 MHz, d_8-THF) and of the same sample after 4 days in solution at room temperature under N_2-atmosphere (bottom, 500 MHz, d_8-THF).

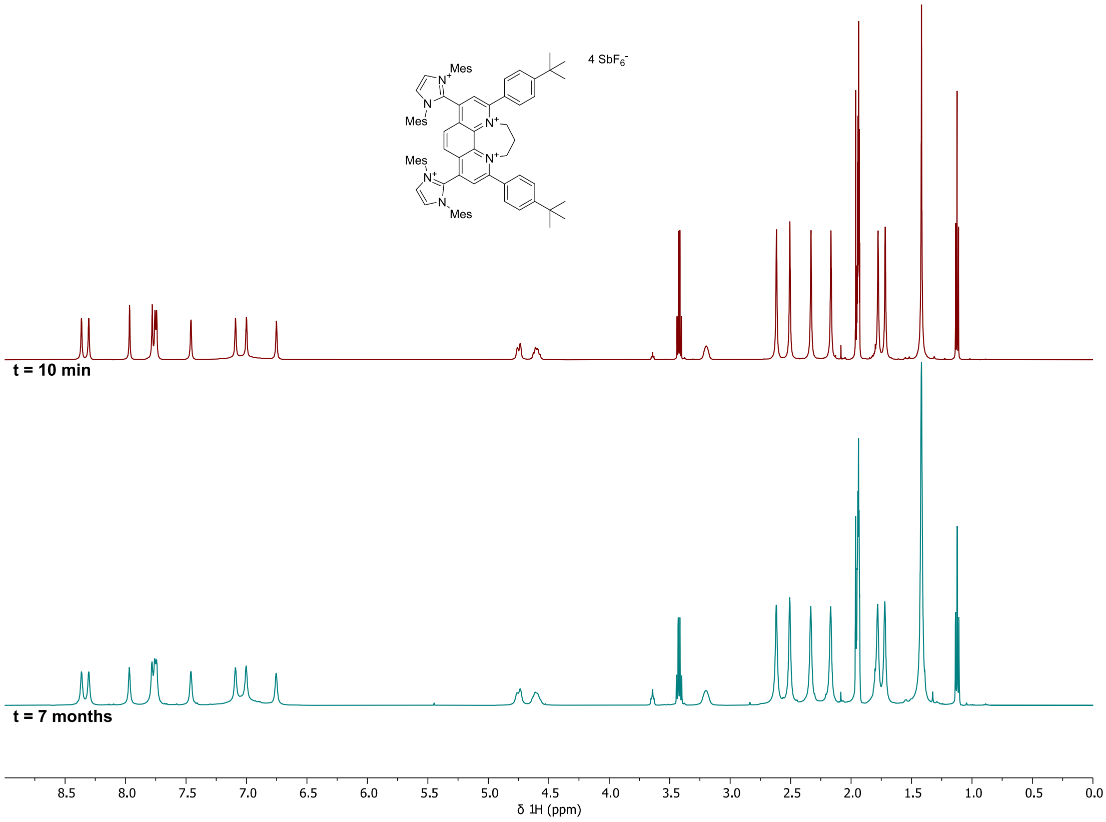

Figure S129. Comparison of ^{1}H NMR spectra of **2c^{4+}** after 10 min (top, 600 MHz, CD_3CN) and of the same sample after 7 months in solution at room temperature under N_2-atmosphere (bottom, 500 MHz, CD_3CN).

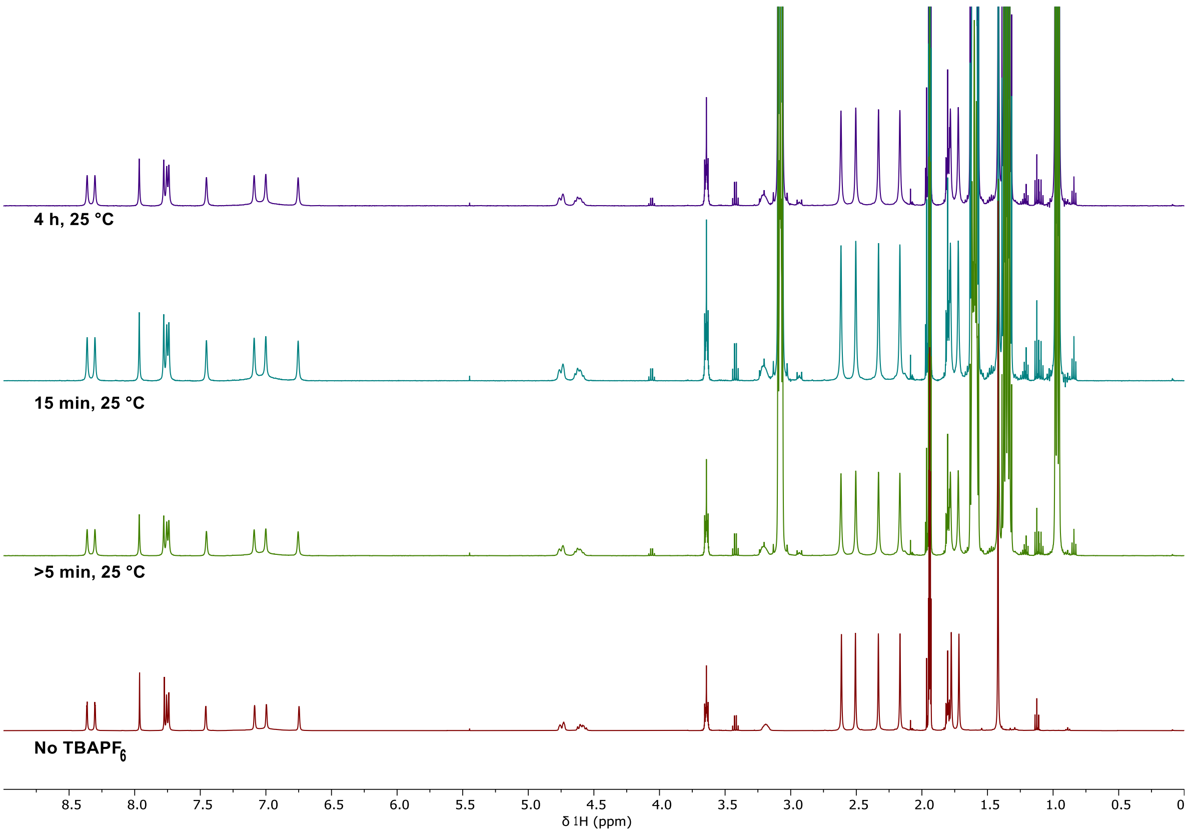

Figure S130. Influence of *n*-$BuNPF_6$ (~ 10 eq.) on the stability of **2c^{4+}** (10 mg/ml, CD_3CN).

11. H-cell charge/discharge experiments

Charge-discharge experiments were performed in analogy to reports by Sanford et al. in a custom-made H-cell in a nitrogen filled glovebox.[11] Both cell chambers were separated by a P5-frit (15 mm diameter, porosity 5 from Robu®). Reticulated vitreous carbon (RVC) electrodes (100 ppi Duocel®) were cut into rods of the dimensions 0.5 cm x 0.5 cm x 4 cm and positioned ca. 2 cm deep in solution (Fig. S131).

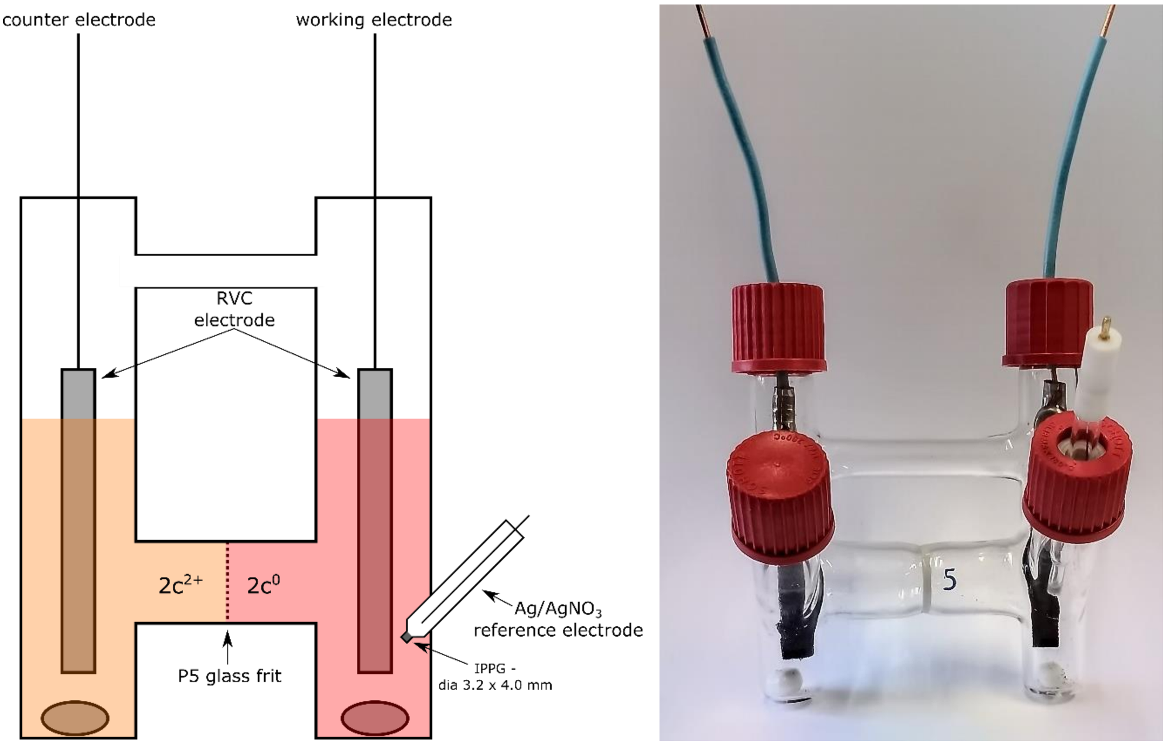

Figure S131. Experimental setup for H-cell cycling experiments. Schematic representation of the H-cell from the front (left). Photograph of the assembled H-cell, front view (right).

To rule out contamination processes the electrodes were single used and dried for 12 h under reduced pressure at room temperature. As reference an $Ag/AgNO_3$ reference electrode (0.01 M $AgNO_3$ in 0.1 M *n*-Bu_4NPF_6 in CH_3CN) was used. The reference electrode was freshly prepared by using a fritted sample holder, which was activated by storing in a CH_3CN solution for one night, followed by diluted HCl (1M) for one-night, demineralized water for one night, dried and stored in dry, degassed CH_3CN for at least one additional night. To the fritted sample holder was added a freshly prepared 0.01 M $AgNO_3$/0.1 M *n*-Bu_4NPF_6 solution in CH_3CN and a silver wire. Tetrabutylammonium hexafluorophosphate (electrochemical grade from Sigma Aldrich) was dried at 80 °C under high vacuum for 12 h prior to use. A 0.5 M tetrabutylammonium hexafluorophosphate solution in CH_3CN (anhydrous grade dried additionally over 4Å molecular sieves and filtrated over activated aluminium oxide) was freshly prepared.

H-cell cycling with constant 2C rate

Solutions (0.5 M TBAP) of neutral **2c** (2.5 mM) and dication **2c^{2+}** (2.5 mM) were separately prepared and filled into each chamber of the H-cell (each 6 mL). Then a 2C current was applied with cut-off potentials at -1.8 V and -0.8 V (against the internal Ag/$AgNO_3$ reference). During charge/discharge cycling both cells were stirred constantly at ~1000 rpm.

H-cell cycling with changing C rate

In case of different C-rates the procedure above was followed. In the beginning 10 charge/discharge cycles were measured at 1C, followed by each 5 cycles at 2C, 3C, 4C and 5C. Afterwards 20 cycles with 1C were measured (Fig. S132).

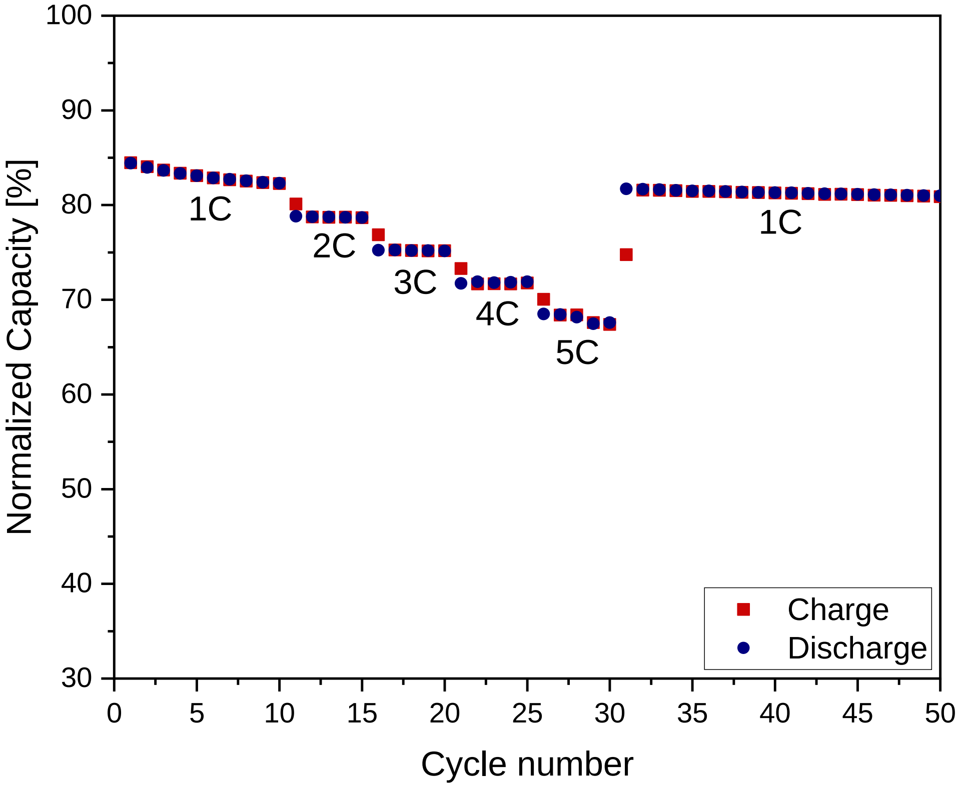

Figure S132. Capacity as a function of C-rate and cycle number for the symmetrical H-cell for redox-system **2c**/**2c^{2+}**.

12. Computational data

All DFT[12] calculations were performed with the Gaussian16 program package[13] (version g16, rev.C01). All calculations were performed with the B3LYP functionals employing Ahlrich's def2-SVP or def2-TZVP(P) basis sets[14] or the Pople 6-31G* basis set. Ground states were fully optimized without constraints at the corresponding level of theory and checked by a frequency calculation. Grimme's D3 dispersion correction with Becke-Johnson damping was used in order to take dispersion effects into account.[15] Isotopic hyperfine constants were calculated at the M06-2X/cc-pVDZ level of theory.[16] For the singlet open shell state of **2c**$^{2+}$ optimizations were performed within the broken symmetry approach using the guess=mix keyword.

NICS(1)/NICS(1)$_{zz}$ values were calculated by placing dummy atoms 1 Å above the geometric centre of the heterocyclic plane and performing a single-point calculation (from the (U)B3LYP-D3BJ/def2SVP optimized structure) at the B3LYP/6-31G* level of theory employing the GIAO[17] method implemented in Gaussian16. For the visualization of optimized structures CYLview,[18] and for the visualization of frontier molecular orbitals IboView[19] and GaussView 6.1[20] was used.

Results of the NICS calculation

Nuclear independent shifts (NICS) were calculated on the simplified model system (**2cS**) for the three rings as a function of the oxidation state (Table S5). In agreement with the Lewis structures the NICS(1) and NICS(1)zz values indicate for the central core decreasing aromaticity from neutral **2cS** to the dication (**2cS**$^{2+}$) and increasing aromaticity to the tetracation (**2cS**$^{4+}$), both for the middle (B) as well as outer ring systems (A and C), considering a closed shell configuration for **2c**$^{2+}$. The energetic penalty upon two electron oxidation can be compensated by the aromatization of the imidazolium heterocycles. The most positive (least aromatic) NICS values of the dicationic oxidation state (**2cS**$^{2+}$) are comparable with the recently described antiaromatic paraquat[21] also in respect to the significant high-field shifted ^{1}H NMR signal [δ(^{1}H): 4.03 ppm]. ^{1}H NMR data [δ(^{1}H) > 7 ppm] of the tetracation **2c**$^{4+}$ indicates full aromaticity of the central core in agreement with the negative NICS values for rings A-C.

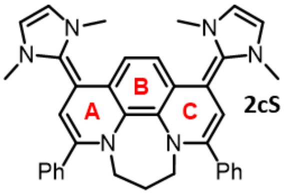

2cS

	A	B	C
**$2cS^0$**	+1.1 (+9.8)	-6.0 (-10.1)	+3.7 (+16.8)
**$2cS^{1+}$**	+6.9 (+27.0)	-4.7 (-5.4)	+7.0 (+27.4)
**$2cS^{2+}$**	+13.4 (+46.2)	-2.0 (+2.9)	+13.4 (+47.2)
**$2cS^{3+}$**	+1.5 (+10.7)	-5.2 (-6.7)	+1.5 (+10.8)
**$2cS^{4+}$**	-6.8 (-13.7)	-10.7 (-22.8)	-7.0 (-13.7)

Table S5. Calculated NICS(1) and NICS(1)zz in parenthesis for the three cycles A, B and C as a function of the oxidation states; (U)B3LYP/6-31G(d) level of theory; in case of **$2cS^{2+}$** the closed shell state was considered. The values are each averaged of the NICS(1)/NICS(1)zz values of the concave and convex sites of the core.

Calculation of the UV-VIS properties

TD-DFT calculations were performed to calculate the spectral properties of the three redox systems **2a**-**2c** for each of the oxidation states. The calculations were performed at the B3LYP/def2TZVP level of theory considering the first 30 excited states (singlets, nstates=30). As structural input an optimized simplified model (N-Me) was used (D3BJ-B3LYP/def2-SVP).

For each oxidation state for each redox system the calculations were performed with a continuum solvent model (CPCM) as well as at the CAM-B3LYP functional and solvent model (CPCM). In general, there is a very good fit between experimental and theoretical data (see Figures S133-S146). In case of the mixed-valent systems the TD-DFT calculations also predict strong NIR absorptions fairly close to the experimental data. However, the variation from experiment is not surprising since TD-DFT calculations are known not to be accurate for long-range charge-transfer excited states.[22] In case of system **2c** the syn and anti conformer of the propyl spacer were calculated. The data matches very well with the experimental data based on X-ray analysis.

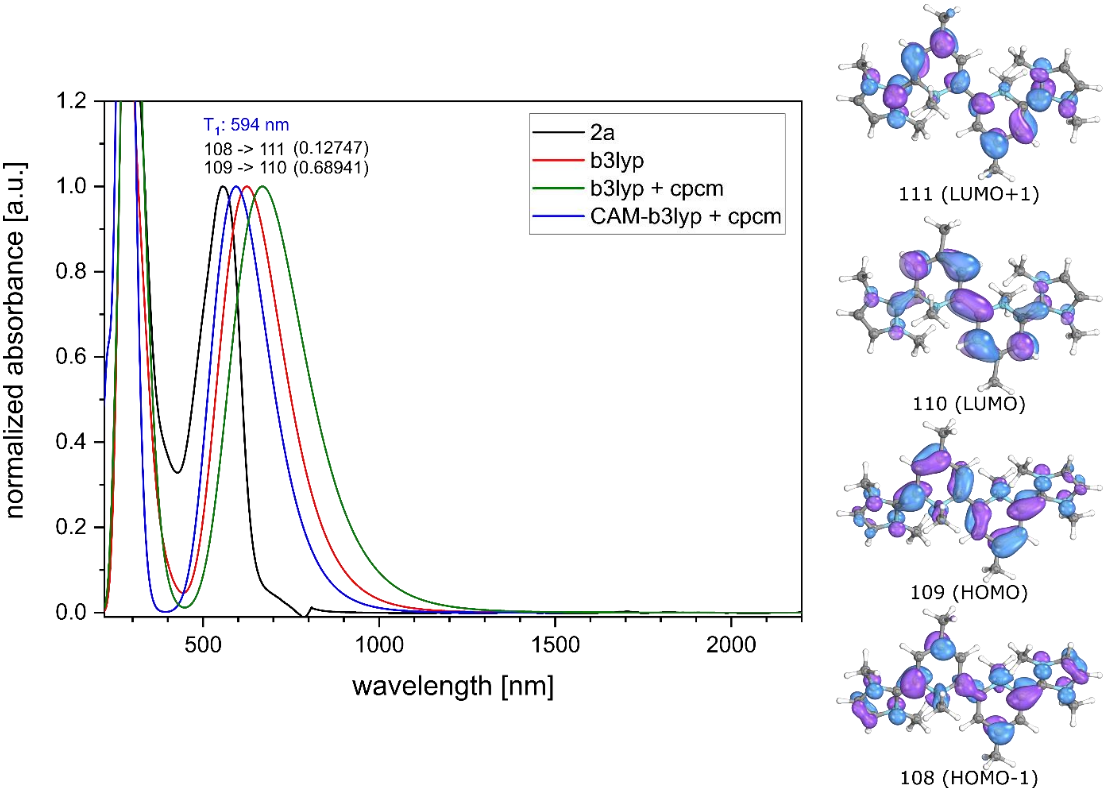

Figure S133. Left: Comparison of experimental UV-Vis-NIR spectra of **2a** (black) with TD-DFT data (red: b3lyp/def2TZVP; green b3lyp/def2TZVP+cpcm(THF); blue CAM-b3lyp/def2TZVP+cpcm(THF)). One major transition (T1) is highlighted with its wavelength and orbitals involved in the transition. Right: Depiction of the molecular orbitals of **2a** (CAM-b3lyp/def2TZVP+cpcm(THF)) with an isovalue of 0.65.

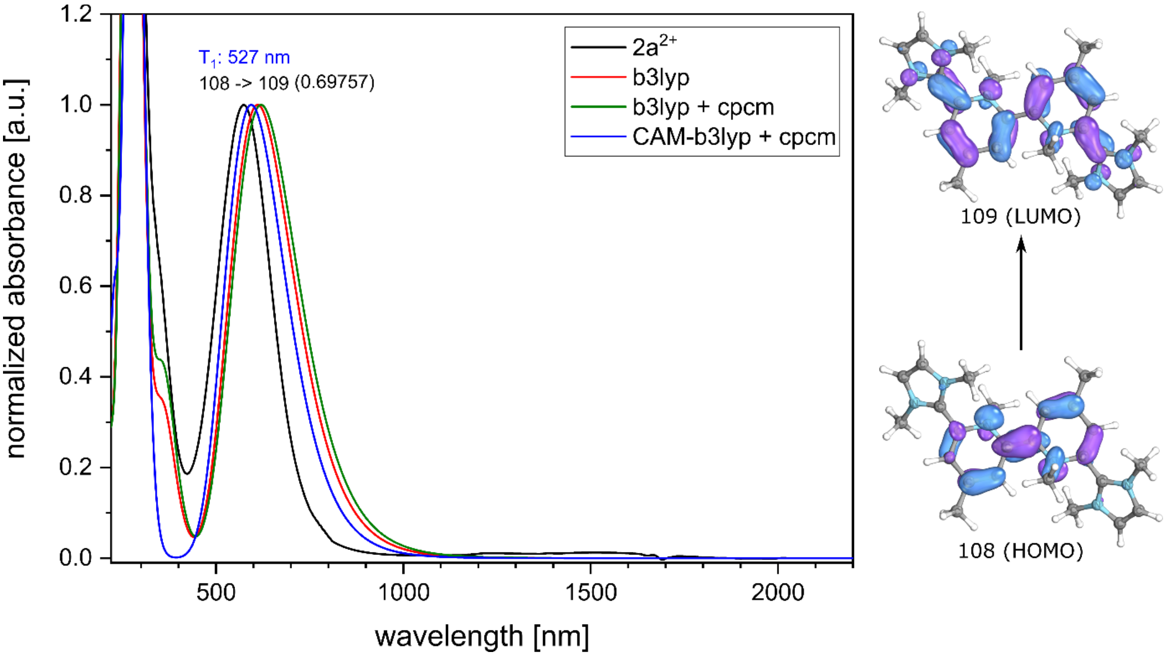

Figure S134. Left: Comparison of experimental UV-Vis-NIR spectra of $\mathbf{2a^{2+}}$ (black) with TD-DFT data (red: b3lyp/def2TZVP; green b3lyp/def2TZVP+cpcm(CH_2Cl_2); blue CAM-b3lyp/def2TZVP+cpcm(CH_2Cl_2)). One major transition (T1) is highlighted with its wavelength and orbitals involved in the transition. Right: Depiction of the molecular orbitals of $\mathbf{2a^{2+}}$ (CAM-b3lyp/def2TZVP+cpcm(THF)) with an isovalue of 0.55.

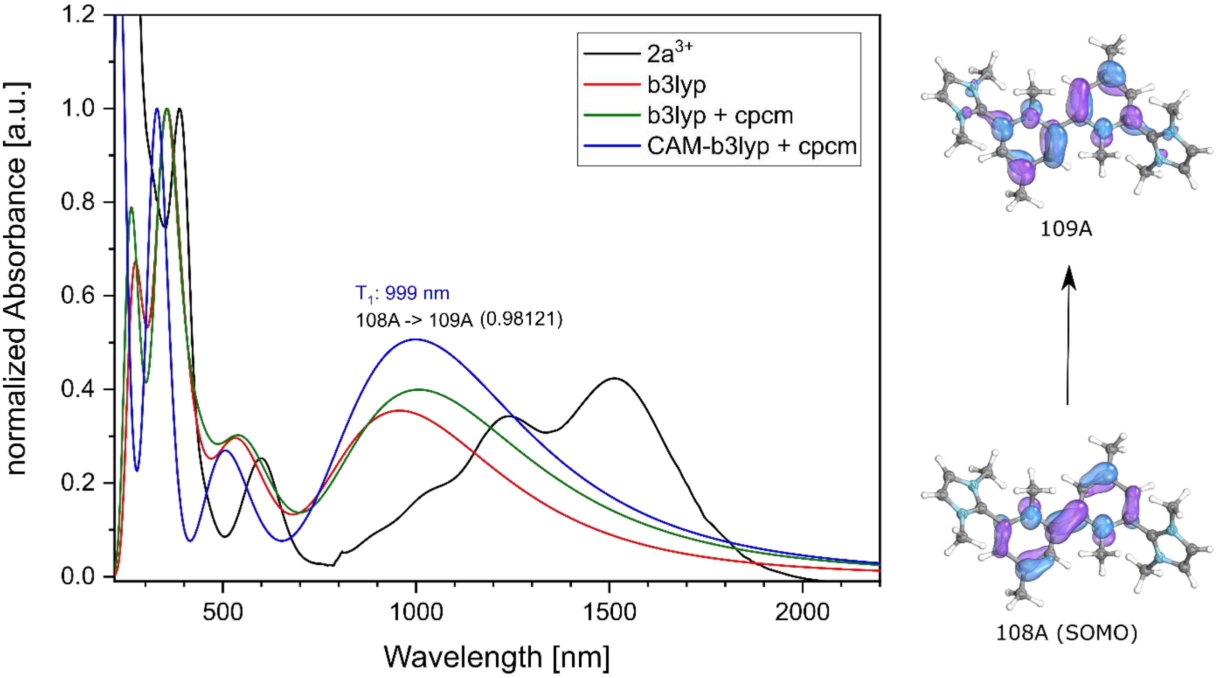

Figure S135. Left: Comparison of experimental UV-Vis-NIR spectra of **2a**$^{3+}$ (black) with TD-DFT data (red: b3lyp/def2TZVP; green b3lyp/def2TZVP+cpcm(THF); blue CAM-b3lyp/def2TZVP+cpcm(THF)). One major transition (T1) is highlighted with its wavelength and orbitals involved in the transition. Right: Depiction of the molecular orbitals of **2a**$^{3+}$ (CAM-b3lyp/def2TZVP+cpcm(THF)) with an isovalue of 0.55.

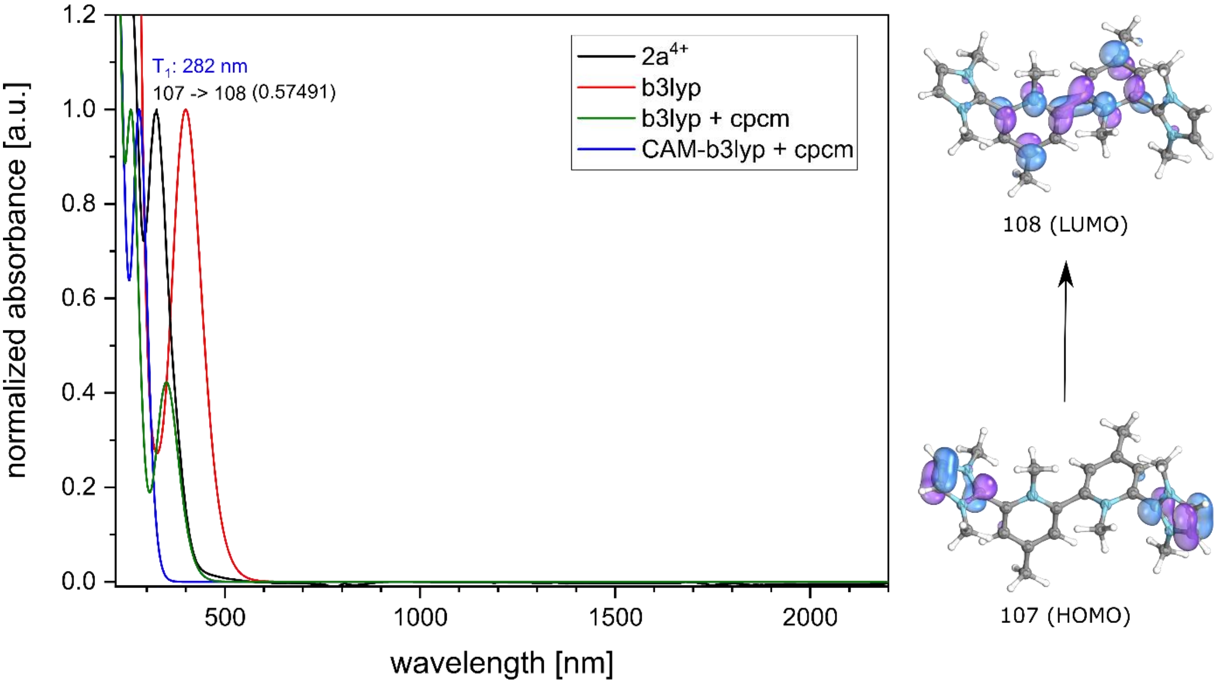

Figure S136. Left: Comparison of experimental UV-Vis-NIR spectra of **2a**$^{4+}$ (black) with TD-DFT data (red: b3lyp/def2TZVP; green b3lyp/def2TZVP+cpcm(THF); blue CAM-b3lyp/def2TZVP+cpcm(THF)). One major transition (T1) is highlighted with its wavelength and orbitals involved in the transition. Right: Depiction of the molecular orbitals of **2a**$^{4+}$ (CAM-b3lyp/def2TZVP+cpcm(THF)) with an isovalue of 0.55.

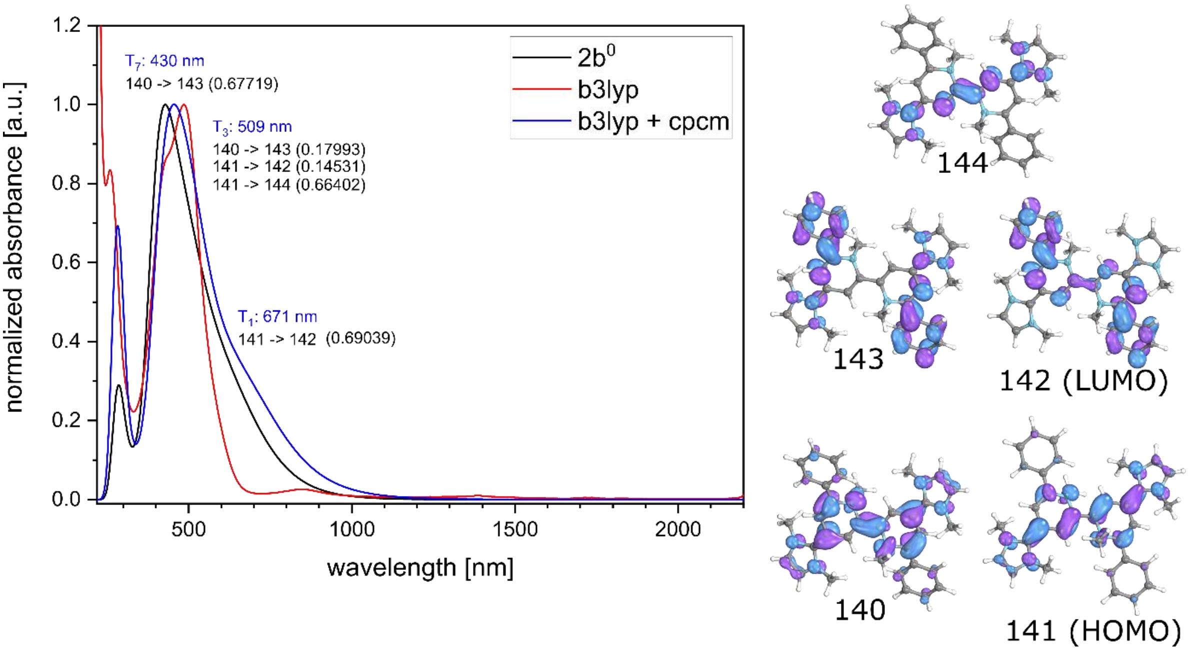

Figure S137. Left: Comparison of experimental UV-Vis-NIR spectra of **2b** (black) with TD-DFT data (red: b3lyp/def2TZVP; blue b3lyp/def2TZVP+cpcm(THF)). The major transitions (T1, T3, T7) are highlighted with their wavelengths and orbitals involved in the transition. Right: Depiction of the molecular orbitals of **2b** (b3lyp/def2TZVP+cpcm(THF)) with an isovalue of 0.55.

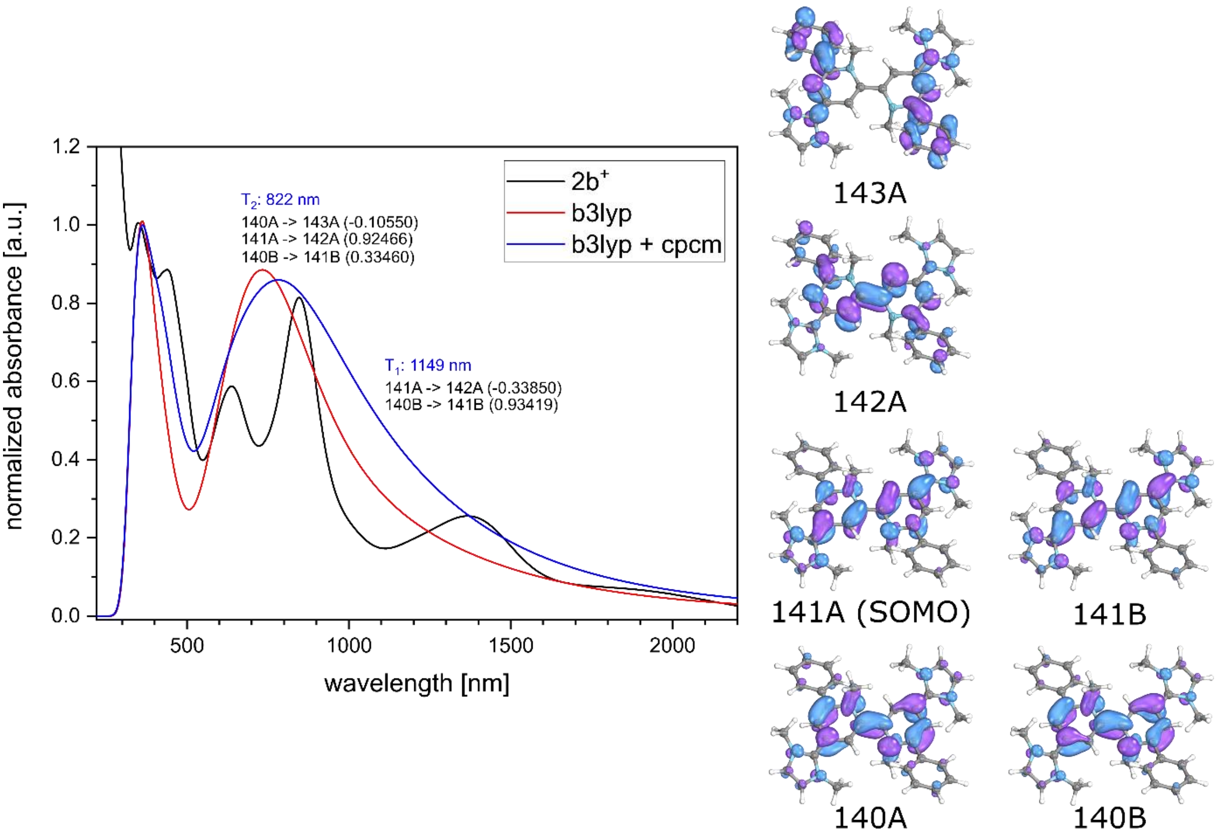

Figure S138. Left: Comparison of experimental UV-Vis-NIR spectra of **2b**$^{+}$ (black) with computational data (red: b3lyp/def2TZVP; blue b3lyp/def2TZVP+cpcm(THF)). The major two transitions (T1 and T2) are highlighted with their wavelengths and orbitals involved in the transition. Right: Depiction of the molecular orbitals of **2b**$^{+}$ (b3lyp/def2TZVP+cpcm(THF)) with an isovalue of 0.55.

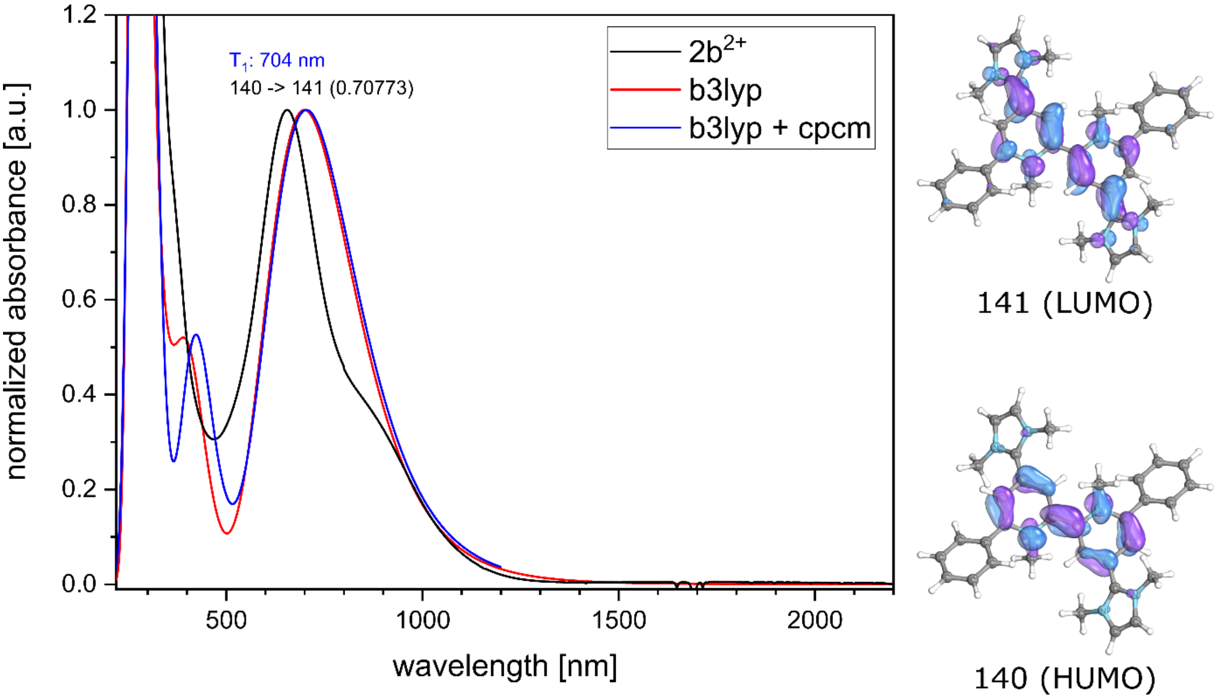

Figure S139. Left: Comparison of experimental UV-Vis-NIR spectra of **2b**$^{2+}$ (black) with computational data (red: b3lyp/def2TZVP; blue b3lyp/def2TZVP+cpcm(CH_2Cl_2)). The major transition (T1) is highlighted with its wavelength and orbitals involved in the transition. Right: Depiction of the molecular orbitals of **2b**$^{2+}$ (b3lyp/def2TZVP+cpcm(CH_2Cl_2)) with an isovalue of 0.55.

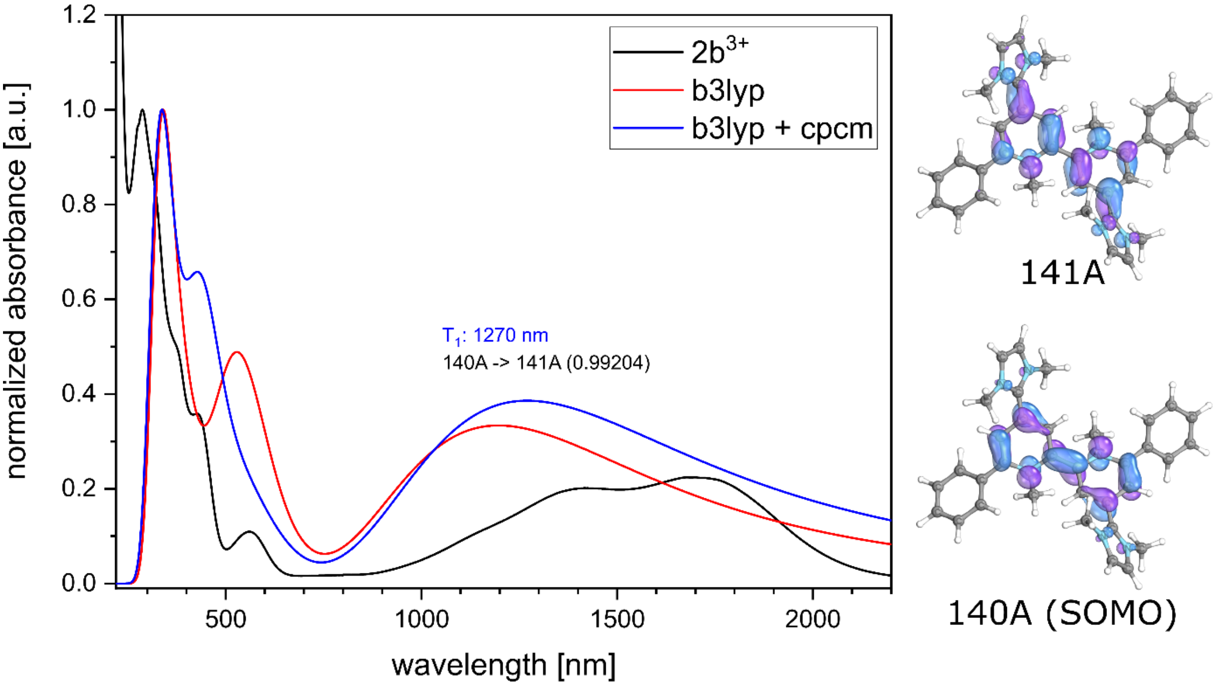

Figure S140. Left: Comparison of experimental UV-Vis-NIR spectra of **2b**$^{3+}$ (black) with computational data (red: b3lyp/def2TZVP; blue b3lyp/def2TZVP+cpcm(THF)). The major transition (T1) is highlighted with its wavelength and orbitals involved in the transition. Right: Depiction of the molecular orbitals of **2b**$^{3+}$ (b3lyp/def2TZVP+cpcm(THF)) with an isovalue of 0.55.

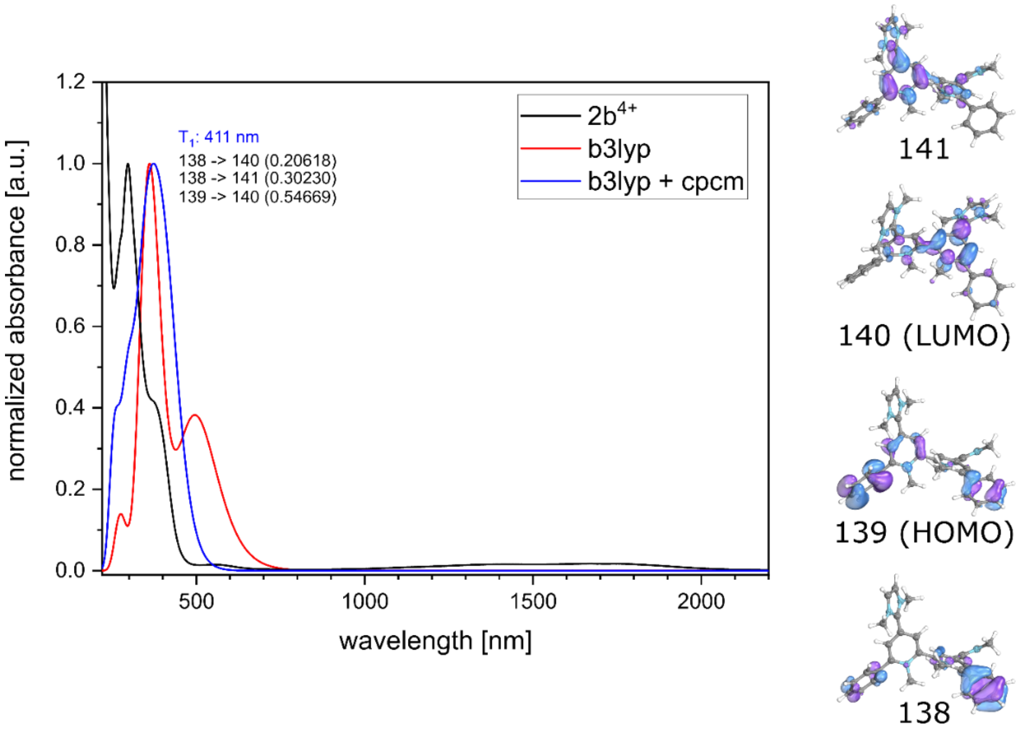

Figure S141. Left: Comparison of experimental UV-Vis-NIR spectra of $\mathbf{2b^{4+}}$ (black) with computational data (red: b3lyp/def2TZVP; blue b3lyp/def2TZVP+cpcm(THF)). The major transition (T1) is highlighted with its wavelength and orbitals involved in the transition. Right: Depiction of the molecular orbitals of $\mathbf{2b^{4+}}$ (b3lyp/def2TZVP+cpcm(THF)) with an isovalue of 0.55.

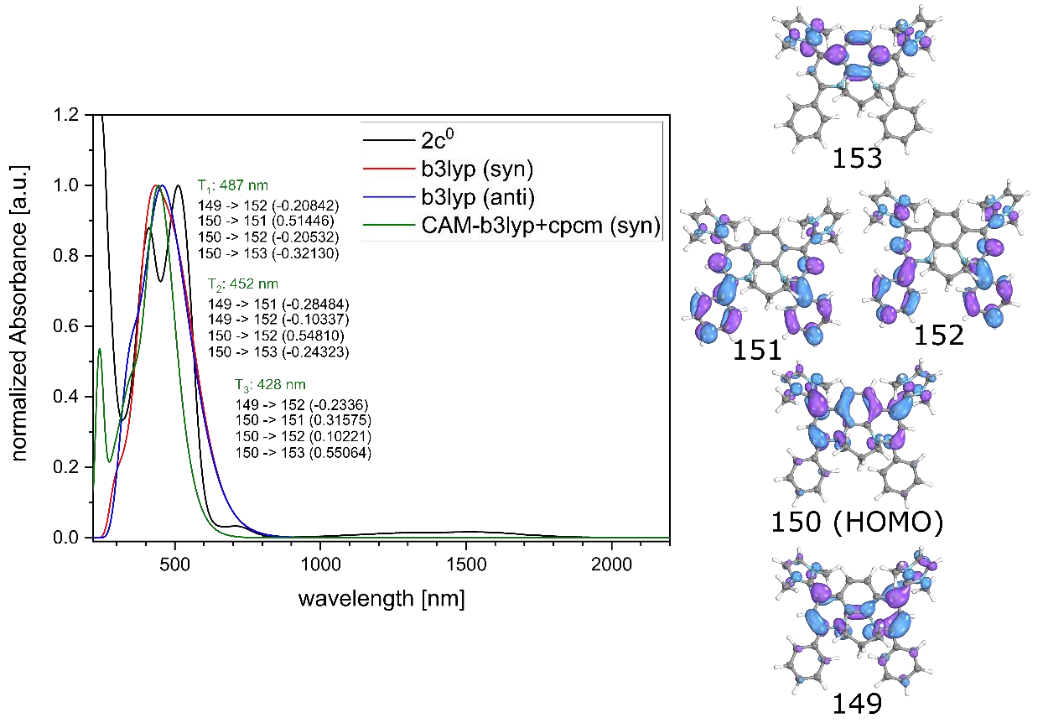

Figure S142. Left: Comparison of experimental UV-Vis-NIR spectra of **2c** (black) with computational data (red: b3lyp/def2TZVP, syn-bridged isomer; blue b3lyp/def2TZVP, anti-bridged isomer; green CAM-b3lyp/def2TZVP+cpcm(THF)). Three major transitions (T1-3) are highlighted with their wavelength and orbitals involved in the transition. Right: Depiction of the molecular orbitals of **2c** (CAM-b3lyp/def2TZVP+cpcm(THF)) with an isovalue of 0.55 of the syn-bridged isomer.

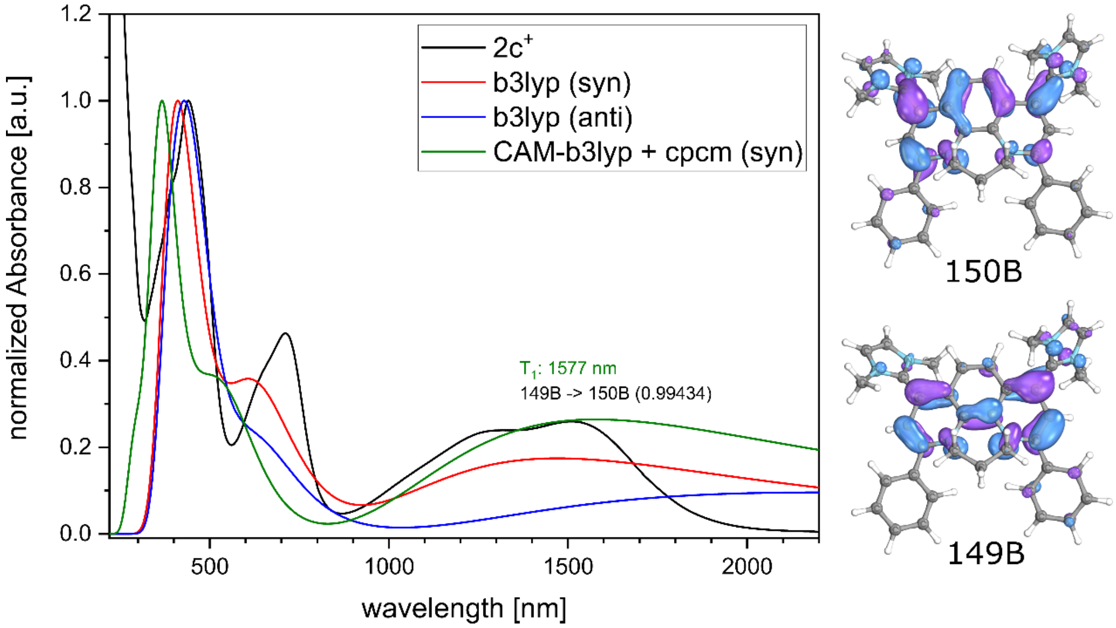

Figure S143. Left: Comparison of experimental UV-Vis-NIR spectra of **2c**$^{+}$ (black) with computational data (red: b3lyp/def2TZVP, syn-bridged isomer; blue b3lyp/def2TZVP, anti-bridged isomer; green CAM-b3lyp/def2TZVP+cpcm(THF)). One major transition (T1) is highlighted with its wavelength and orbitals involved in the transition. Right: Depiction of the molecular orbitals of **2c**$^{+}$ (CAM-b3lyp/def2TZVP+cpcm(THF)) with an isovalue of 0.55 of the syn-bridged isomer.

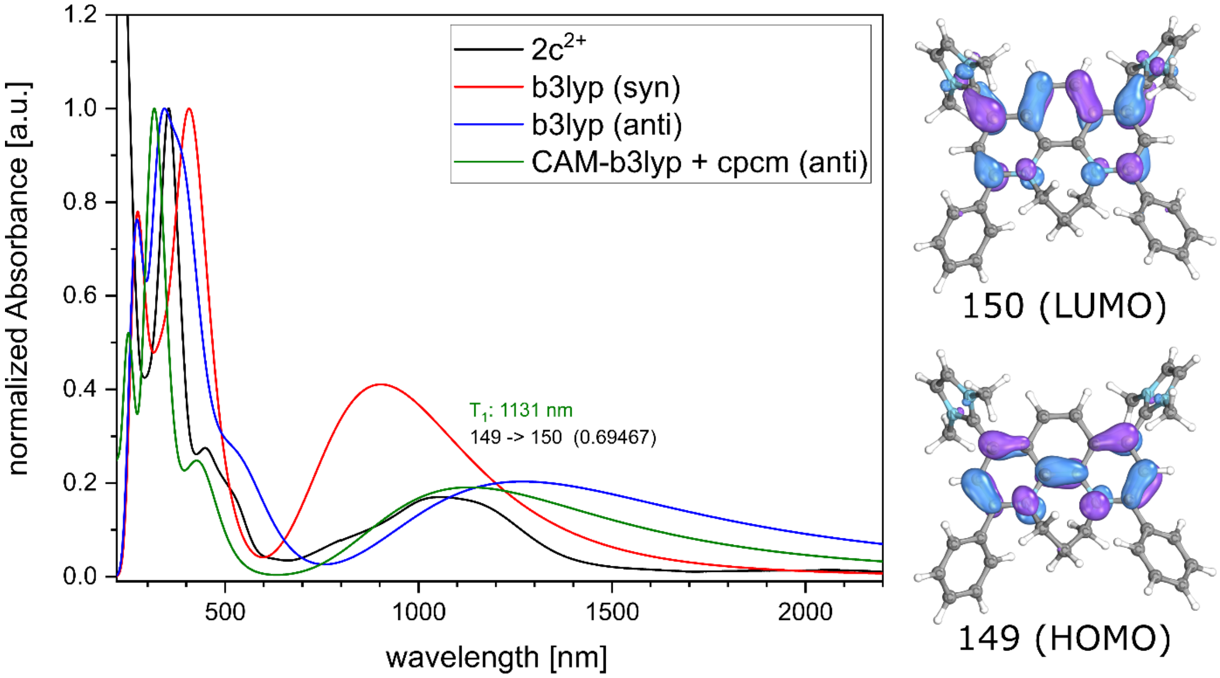

Figure S144. Left: Comparison of experimental UV-Vis-NIR spectra of **2c**$^{2+}$ (black) with computational data (red: b3lyp/def2TZVP, syn-bridged isomer; blue b3lyp/def2TZVP, anti-bridged isomer; green CAM-b3lyp/def2TZVP+cpcm(CH_2Cl_2)). One major transition (T1) is highlighted with its wavelength and orbitals involved in the transition. Right: Depiction of the molecular orbitals of **2c**$^{2+}$ (CAM-b3lyp/def2TZVP+cpcm(CH_2Cl_2)) with an isovalue of 0.55 of the anti-bridged isomer.

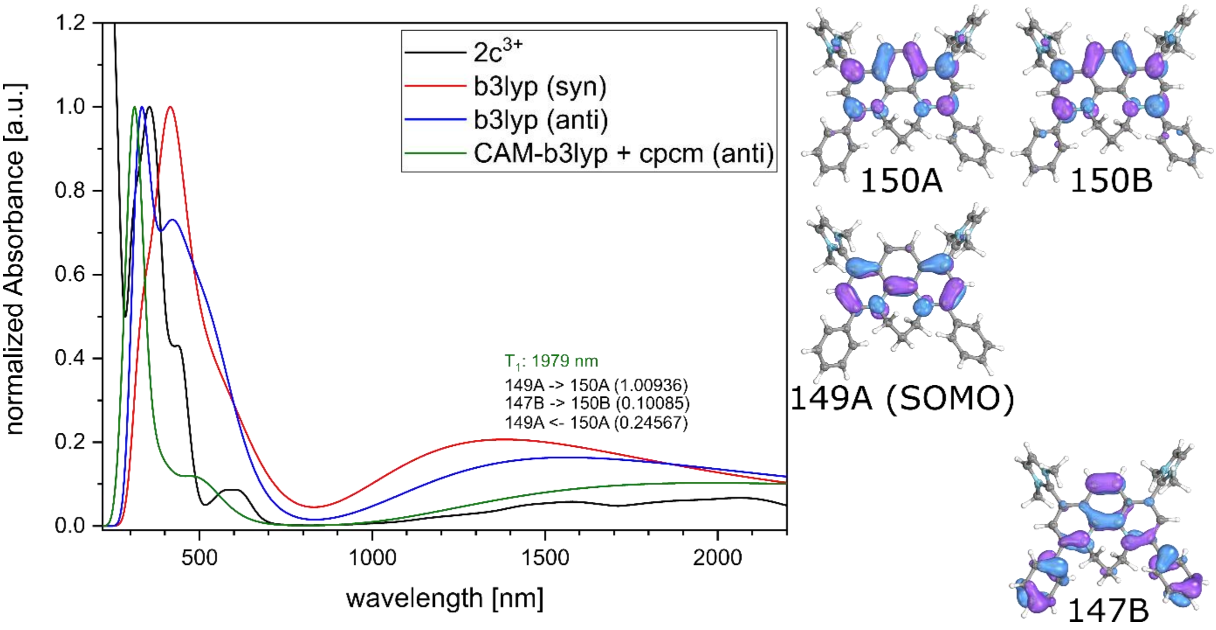

Figure S145. Left: Comparison of experimental UV-Vis-NIR spectra of **2c**$^{3+}$ (black) with computational data (red: b3lyp/def2TZVP, syn-bridged isomer; blue b3lyp/def2TZVP, anti-bridged isomer; green CAM-b3lyp/def2TZVP+cpcm(THF)). One major transition (T1) is highlighted with its wavelength and orbitals involved in the transition. Right: Depiction of the molecular orbitals of **2c**$^{3+}$ (CAM-b3lyp/def2TZVP+cpcm(THF)) with an isovalue of 0.55 of the anti-bridged isomer.

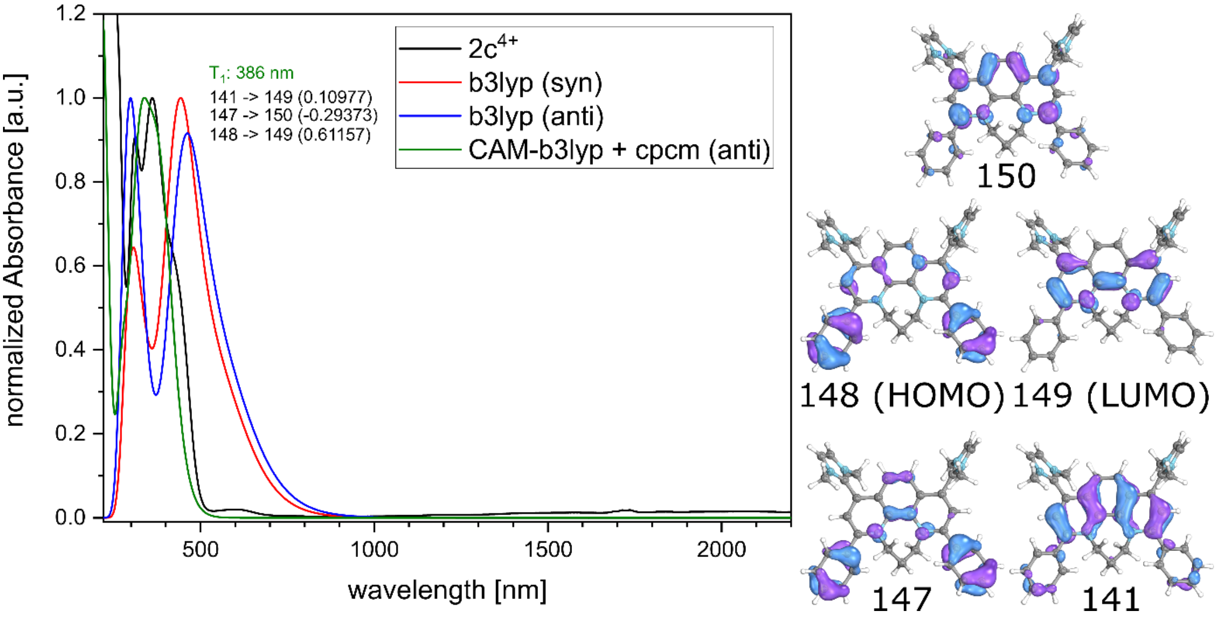

Figure S146. Left: Comparison of experimental UV-Vis-NIR spectra of **2c**$^{4+}$ (black) with computational data (red: b3lyp/def2TZVP, syn-bridged isomer; blue b3lyp/def2TZVP, anti-bridged isomer; green CAM-b3lyp/def2TZVP+cpcm(THF)). One major transition (T1) is highlighted with its wavelength and orbitals involved in the transition. Right: Depiction of the molecular orbitals of **2c**$^{4+}$ (CAM-b3lyp/def2TZVP+cpcm(THF)) with an isovalue of 0.55 of the anti-bridged isomer.

DFT optimization of $2a^{3+}$

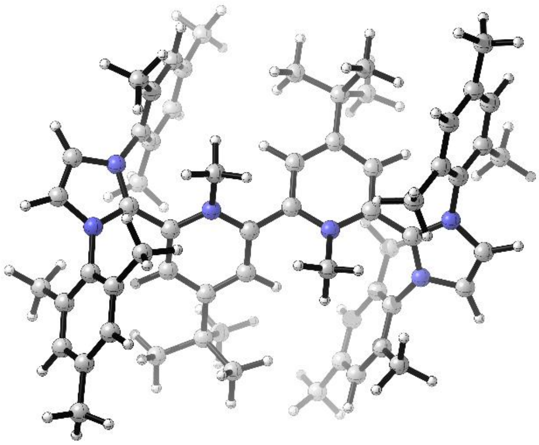

#p opt freq ub3lyp def2svp empiricaldispersion=gd3bj gfinput iop(6/7=3)

Charge = 3 Multiplicity = 2

NImag=0

Zero-point correction= 1.242502 (Hartree/Particle)

Thermal correction to Energy= 1.312804

Thermal correction to Enthalpy= 1.313748

Thermal correction to Gibbs Free Energy= 1.134994

Sum of electronic and zero-point Energies= -2733.281083

Sum of electronic and thermal Energies= -2733.210781

Sum of electronic and thermal Enthalpies= -2733.209837

Sum of electronic and thermal Free Energies= -2733.388591

Atom	X	Y	Z
N	1.65086	-0.70641	0.33845
N	3.96967	-2.81477	-0.07389
N	5.20267	-1.29739	0.90741
N	-1.65996	0.76611	0.3398
N	-5.19874	1.20118	1.0317
N	-4.08651	2.80252	0.03947
C	3.99765	-1.51464	0.31921
C	2.99634	-0.47213	0.07648
C	3.40772	0.74251	-0.41656
H	4.46455	0.88378	-0.61979
C	2.49348	1.78945	-0.70662
C	1.14696	1.48171	-0.55932
H	0.3883	2.19355	-0.87945
C	0.70679	0.23241	-0.08697
C	-0.69364	-0.14039	-0.10589
C	-1.10742	-1.37671	-0.62439
H	-0.33134	-2.06173	-0.96667
C	-2.44818	-1.71089	-0.79193
C	-3.38475	-0.70585	-0.45274
H	-4.44004	-0.86509	-0.64417
C	-2.99811	0.5026	0.08468
C	-4.03333	1.49359	0.39771
C	3.02391	3.13257	-1.19286
C	1.88405	4.10245	-1.52316
H	1.24452	3.72184	-2.33354
H	2.30099	5.06243	-1.85762
H	1.24717	4.30795	-0.64891
C	3.88463	2.91873	-2.45805
H	3.29816	2.45912	-3.26858
H	4.76253	2.28424	-2.26424
H	4.26014	3.88755	-2.81894

C	3.89652	3.7296	-0.06319
H	3.30465	3.89833	0.84958
H	4.30932	4.6972	-0.38576
H	4.7421	3.07587	0.19763
C	1.25636	-1.76314	1.27896
H	0.4206	-1.39676	1.88859
H	2.10035	-1.98267	1.94236
H	0.95152	-2.68485	0.77148
C	5.16533	-3.41707	0.27858
H	5.36874	-4.45703	0.04136
C	5.93159	-2.47012	0.89455
H	6.92688	-2.52479	1.32698
C	2.94274	-3.48403	-0.83865
C	2.30619	-4.60217	-0.25899
C	1.37155	-5.2872	-1.04032
H	0.87374	-6.16133	-0.61314
C	1.07938	-4.90854	-2.35994
C	1.73158	-3.78667	-2.88713
H	1.53233	-3.49131	-3.9202
C	2.68343	-3.06186	-2.15626
C	3.41179	-1.9154	-2.81106
H	3.49374	-2.08616	-3.89305
H	4.43071	-1.7867	-2.41852
H	2.87924	-0.9602	-2.67297
C	0.1394	-5.73023	-3.1979
H	-0.20454	-5.18454	-4.08673
H	-0.73766	-6.05584	-2.61982
H	0.64879	-6.64375	-3.54788
C	2.61879	-5.08758	1.13479
H	3.44328	-5.81967	1.12473
H	1.75052	-5.60126	1.56995
H	2.91657	-4.27849	1.81644
C	5.69967	-0.04661	1.44093
C	6.69721	0.62873	0.71195
C	7.16963	1.83357	1.2407
H	7.94307	2.37847	0.6939
C	6.68779	2.35696	2.45108
C	5.69251	1.64441	3.1348
H	5.31328	2.03507	4.08218
C	5.18042	0.43465	2.65305
C	4.10479	-0.30124	3.40902
H	3.1035	-0.06371	3.00865
H	4.10462	-0.01044	4.46796
H	4.2366	-1.39317	3.36415
C	7.2534	3.63177	3.01272
H	8.18483	3.42253	3.56597
H	6.55744	4.11414	3.7131
H	7.50621	4.34727	2.21671
C	7.24217	0.08938	-0.58682
H	7.97544	-0.71605	-0.41624
H	7.75911	0.87777	-1.14953
H	6.45478	-0.32975	-1.23451
C	-1.2818	1.84409	1.26097
H	-0.41846	1.51402	1.8522
H	-2.11732	2.04052	1.94141
H	-1.02442	2.77035	0.73511
C	-5.98341	2.3358	1.08364
H	-6.9593	2.33025	1.56137
C	-5.29235	3.33514	0.46054
H	-5.55628	4.36948	0.26105
C	-5.60493	-0.08568	1.55591
C	-5.001	-0.56849	2.72797
C	-5.42618	-1.81401	3.20227
H	-4.98096	-2.20653	4.11978
C	-6.41913	-2.56004	2.55073
C	-6.98826	-2.03273	1.38103
H	-7.76081	-2.60283	0.85928
C	-6.60403	-0.79313	0.86113
C	-7.24098	-0.25327	-0.39458
H	-8.01014	0.50311	-0.16701
H	-7.73797	-1.05602	-0.95488
H	-6.51124	0.22797	-1.06605
C	-6.89372	-3.87278	3.10974
H	-6.12884	-4.34988	3.73841
H	-7.18268	-4.57237	2.31206
H	-7.7855	-3.71595	3.74036
C	-3.92783	0.20349	3.45099
H	-2.935	0.02094	3.00352
H	-3.86762	-0.10621	4.50288
H	-4.11177	1.28861	3.43474
C	-3.12919	3.53975	-0.75296
C	-2.51532	4.66894	-0.17263
C	-1.64996	5.41599	-0.97819
H	-1.16842	6.29855	-0.54981
C	-1.40871	5.08744	-2.32022
C	-2.03997	3.95337	-2.84916
H	-1.87973	3.6957	-3.89912
C	-2.91967	3.16685	-2.09514
C	-3.6304	2.00715	-2.74647
H	-3.0488	1.07418	-2.66591
H	-3.77705	2.20483	-3.817
H	-4.62074	1.82074	-2.30645
C	-0.55104	5.96766	-3.18666
H	-0.03496	5.39471	-3.97022
H	0.1958	6.51756	-2.5974
H	-1.17924	6.71831	-3.69588
C	-2.78261	5.10484	1.24677
H	-3.63303	5.8053	1.29283
H	-1.91558	5.63985	1.65816
H	-3.02031	4.26792	1.91843
C	-2.8705	-3.05494	-1.38157
C	-2.34633	-3.13965	-2.83238
C	-2.25225	-4.18134	-0.52501
C	-4.3986	-3.21787	-1.38591
H	-2.76851	-2.33477	-3.45334
H	-1.24947	-3.06886	-2.87537
H	-2.63929	-4.10018	-3.2808
H	-2.60281	-4.12353	0.51693
H	-2.55142	-5.16093	-0.92643
H	-1.15279	-4.1457	-0.526
H	-4.66008	-4.212	-1.77483
H	-4.82547	-3.13341	-0.37472
H	-4.89146	-2.47946	-2.03722

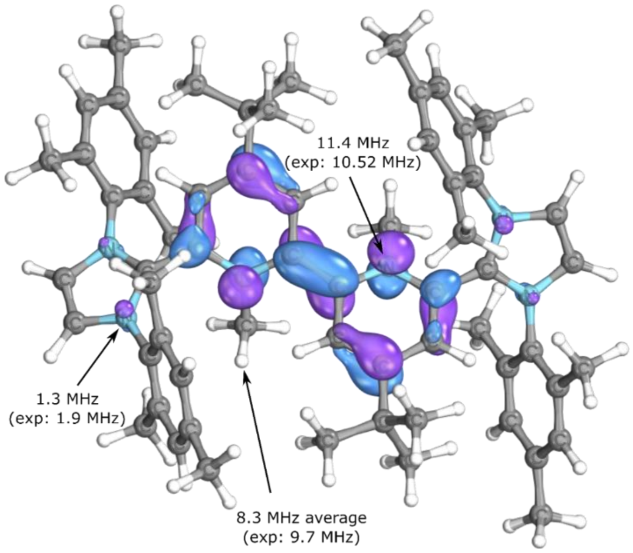

Figure S147. Visualized SOMO (B3LYP/def2-TZVP-gd3bj) and calculated hyperfine coupling constants at the M06-2X/cc-pVDZ//B3LYP/def2-SVP-gd3bj level of theory.

<u>Comparison of linker orientations</u>

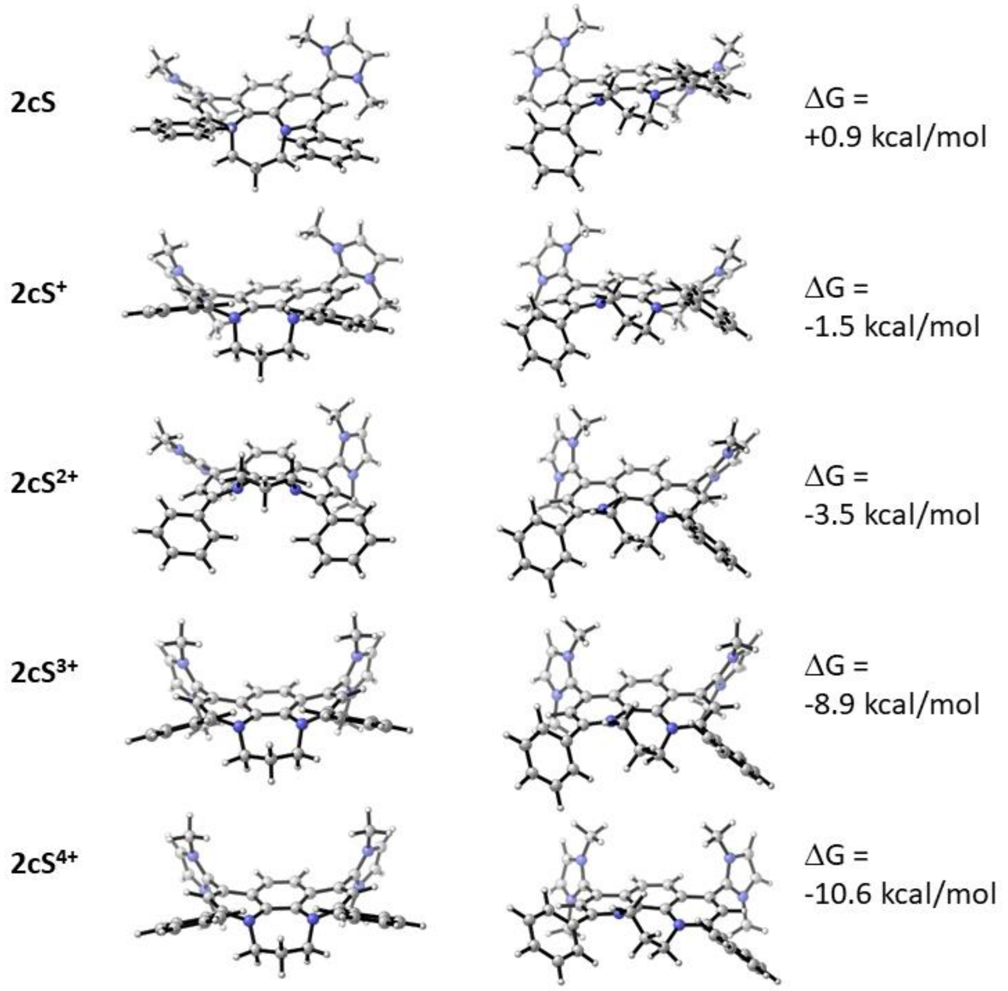

Fig. S148. Optimized structures at the B3LYP-D3BJ/def2SVP level of theory for the five different oxidation states. Left: N-propyl-N *syn* conformation, right: N-propyl-N *trans* conformation. Energy difference in between *trans*/*syn* conformations showing the stabilization of the trans form with increasing oxidation state.

X, Y, Z- coordinates

2a (simplified)

62

scf done: -1261.424790

C	-4.082652	0.196729	0.054833
C	-2.966557	-0.617254	-0.146274
C	-2.895284	-2.027685	0.099992
C	-1.671063	-2.627524	0.290010
C	-0.511082	-1.776067	0.340322
C	-0.557796	-0.463531	-0.055843
N	-1.771757	0.015379	-0.661400
N	-5.426885	-0.169719	0.028119
C	-6.220409	0.955107	0.259320
C	-5.395277	2.015361	0.418825
N	-4.084508	1.565843	0.294159
C	0.557684	0.463402	0.055172
N	1.771565	-0.015486	0.660882
C	2.966460	0.617233	0.146076
C	2.895148	2.027647	-0.100315
C	1.670952	2.627409	-0.290687
C	0.511008	1.775901	-0.341133
C	4.082725	-0.196631	-0.054535
N	5.426912	0.169989	-0.027414
C	6.220650	-0.954763	-0.258266
C	5.395707	-2.015140	-0.417898
N	4.084838	-1.565793	-0.293641
C	5.932122	1.435721	0.441690
C	2.918789	-2.340694	-0.655825
C	1.716197	0.026319	2.126314
C	1.526646	4.098587	-0.572021
C	-1.716691	-0.026700	-2.126834
C	-1.526759	-4.098742	0.571146
C	-2.918240	2.340581	0.655998
C	-5.932382	-1.435383	-0.440866
H	0.411709	-2.162017	0.772806
H	-3.796508	-2.625258	0.252674
H	-1.696344	-1.063901	-2.517564
H	-0.814906	0.493781	-2.480191
H	-2.600655	0.483372	-2.541383
H	-5.620608	3.057712	0.623265
H	-7.304266	0.894031	0.273597
H	-0.411727	2.161784	-0.773797
H	3.796381	2.625243	-0.252825
H	1.695678	1.063448	2.517224
H	0.814391	-0.494315	2.479388
H	2.600127	-0.483745	2.540942
H	5.621237	-3.057487	-0.622139
H	7.304503	-0.893551	-0.272193
H	3.253820	-3.256872	-1.162354
H	2.315215	-2.603804	0.222499
H	2.272179	-1.762683	-1.332551
H	6.980813	1.312068	0.745176
H	5.882426	2.222383	-0.330164
H	5.345430	1.773622	1.310792
H	-6.981199	-1.311636	-0.743876
H	-5.346091	-1.773252	-1.310251
H	-5.882391	-2.222102	0.330908
H	-3.252977	3.256622	1.162971
H	-2.271334	1.762331	1.332230
H	-2.315074	2.603926	-0.222537
H	-0.949386	-4.279652	1.495796
H	-2.507544	-4.586704	0.685884
H	-0.990384	-4.625478	-0.239776
H	2.507435	4.586607	-0.686473
H	0.949565	4.279373	-1.496880
H	0.989956	4.625363	0.238668

$2a^{2+}$ (simplified)

62

scf done: -1261.033538

C	-4.208097	0.149052	0.131032
C	-2.945935	-0.588749	0.089106
C	-2.850484	-1.882059	0.511419
C	-1.593116	-2.591563	0.418608
C	-0.478814	-1.844249	0.154516
C	-0.545402	-0.421492	-0.056631
N	-1.829178	0.108642	-0.408670
N	-5.419326	-0.277530	-0.303858
C	-6.354530	0.716883	-0.091387
C	-5.699717	1.773614	0.471777

N	-4.374891	1.408758	0.604383
C	0.545425	0.421605	0.056784
N	1.829199	-0.108653	0.408783
C	2.945965	0.588708	-0.089149
C	2.850531	1.882034	-0.511385
C	1.593215	2.591628	-0.418368
C	0.478884	1.844384	-0.154217
C	4.208051	-0.149186	-0.131207
N	5.419426	0.277460	0.303224
C	6.354525	-0.717058	0.090762
C	5.699500	-1.773913	-0.471917
N	4.374645	-1.409048	-0.604212
C	5.723728	1.565445	0.928403
C	3.340047	-2.245444	-1.210391
C	1.990752	-0.618786	1.783397
C	1.562832	4.072685	-0.657895
C	-1.990771	0.619618	-1.782948
C	-1.562583	-4.072617	0.658137
C	-3.340500	2.245004	1.211128
C	-5.723417	-1.565406	-0.929366
H	0.504889	-2.317275	0.129840
H	-3.715132	-2.378749	0.955077
H	-2.130649	-0.204928	-2.503999
H	-1.105421	1.194734	-2.074185
H	-2.863579	1.287561	-1.840308
H	-6.068675	2.743573	0.794455
H	-7.400450	0.593757	-0.359651
H	-0.504757	2.317533	-0.129327
H	3.715130	2.378730	-0.955129
H	2.129302	0.206259	2.504138
H	1.106000	-1.194904	2.074504
H	2.864385	-1.285594	1.841399
H	6.068314	-2.743986	-0.794417
H	7.400526	-0.593905	0.358696
H	3.821455	-2.938316	-1.911034
H	2.808552	-2.817503	-0.437277
H	2.626072	-1.614432	-1.751171
H	6.530849	1.418071	1.656662
H	6.046479	2.294874	0.172157
H	4.830995	1.941354	1.441294
H	-6.530184	-1.417879	-1.657987
H	-4.830466	-1.941299	-1.441883
H	-6.046565	-2.294912	-0.173364
H	-3.822118	2.937561	1.911937
H	-2.626596	1.613838	1.751823
H	-2.808884	2.817420	0.438360
H	-0.539437	-4.469992	0.612200
H	-1.982986	-4.320697	1.647048
H	-2.169966	-4.609750	-0.089549
H	1.983102	4.320645	-1.646903
H	0.539745	4.470205	-0.611857
H	2.170412	4.609764	0.089658

$2a^{3+}$ (simplified)

62

scf done: -1260.653412

C	-4.296827	0.090507	0.218116
C	-3.002347	-0.559596	-0.068799
C	-2.976300	-1.829376	-0.586559
C	-1.741174	-2.473850	-0.900108
C	-0.589402	-1.729241	-0.701185
C	-0.599221	-0.407971	-0.202831
N	-1.826088	0.139848	0.191474
N	-4.829274	1.145936	-0.440973
C	-6.080251	1.413783	0.072767
C	-6.314437	0.497840	1.061501
N	-5.202016	-0.313685	1.138485
C	0.599192	0.408028	-0.202844
N	1.826052	-0.139835	0.191492
C	3.002324	0.559565	-0.068778
C	2.976340	1.829347	-0.586558
C	1.741248	2.473852	-0.900120
C	0.589436	1.729285	-0.701187
C	4.296794	-0.090560	0.218129
N	5.202164	0.313912	1.138196
C	6.314566	-0.497652	1.061241
C	6.080167	-1.413913	0.072852
N	4.829092	-1.146213	-0.440723
C	5.070206	1.437468	2.074794
C	4.226045	-1.868184	-1.564020
C	1.887823	-1.333097	1.049660
C	1.732771	3.853436	-1.478819
C	-1.887903	1.333204	1.049504

C	-1.732682	-3.853442	-1.478790
C	-5.069920	-1.437042	2.075291
C	-4.226373	1.867643	-1.564525
H	0.365357	-2.148084	-1.018058
H	-3.921148	-2.335791	-0.792546
H	-2.678013	1.201994	1.799684
H	-2.089163	2.250901	0.479150
H	-0.931601	1.449852	1.571904
H	-7.178102	0.361802	1.709342
H	-6.705060	2.219424	-0.307619
H	-0.365294	2.148179	-1.018074
H	3.921210	2.335702	-0.792578
H	2.677847	-1.201759	1.799918
H	0.931462	-1.449746	1.571961
H	2.089185	-2.250840	0.479428
H	6.704875	-2.219713	-0.307365
H	7.178348	-0.361416	1.708881
H	4.950529	-1.923524	-2.387597
H	3.955101	-2.887216	-1.253380
H	3.338396	-1.326493	-1.910443
H	5.445565	1.125855	3.058053
H	5.658069	2.294093	1.715648
H	4.015918	1.723002	2.159241
H	-3.954820	2.886548	-1.253996
H	-3.339145	1.325520	-1.911343
H	-4.951203	1.923334	-2.387772
H	-5.658167	-2.293600	1.716611
H	-4.015669	-1.722843	2.159300
H	-5.444733	-1.125114	3.058660
H	-0.715279	-4.205305	-1.693454
H	-2.207183	-4.567591	-0.784954
H	-2.316132	-3.888595	-2.414085
H	2.316470	3.888653	-2.413954
H	0.715392	4.205205	-1.693732
H	2.207030	4.567634	-0.784860

$2a^{4+}$ (simplified)

62

scf done: -1260.146171

C	-4.175271	0.147197	0.517370
C	-2.983114	-0.435977	-0.146868
C	-3.122962	-1.587795	-0.909038
C	-2.026871	-2.175202	-1.576603
C	-0.789423	-1.516742	-1.426878
C	-0.659261	-0.366565	-0.657955
N	-1.745387	0.160164	-0.007396
N	-4.852051	1.256383	0.128595
C	-5.973694	1.391232	0.912061
C	-5.983698	0.338860	1.792750
N	-4.871458	-0.425365	1.529683
C	0.659282	0.366636	-0.657906
N	1.745445	-0.160231	-0.007492
C	2.983151	0.435951	-0.146897
C	3.122936	1.587982	-0.908776
C	2.026822	2.175540	-1.576134
C	0.789386	1.516991	-1.426545
C	4.175331	-0.147281	0.517229
N	4.871546	0.425276	1.529526
C	5.983634	-0.339104	1.792760
C	5.973483	-1.391604	0.912214
N	4.851913	-1.256665	0.128671
C	4.551234	1.668666	2.250342
C	4.518554	-2.159083	-0.982151
C	1.603457	-1.356920	0.858703
C	2.189746	3.388888	-2.421731
C	-1.603401	1.356747	0.858943
C	-2.189915	-3.388502	-2.422244
C	-4.550791	-1.668265	2.251170
C	-4.518587	2.158806	-0.982192
H	0.088050	-1.900257	-1.951715
H	-4.117724	-2.029652	-1.006459
H	-2.310403	1.283286	1.693132
H	-1.806197	2.272006	0.286078
H	-0.587198	1.398014	1.263410
H	-6.695176	0.082486	2.577640
H	-6.682621	2.208719	0.781500
H	-0.088137	1.900620	-1.951229
H	4.117677	2.029891	-1.006121
H	2.310353	-1.283487	1.692989
H	0.587211	-1.398289	1.263054
H	1.806365	-2.272107	0.285772
H	6.682261	-2.209250	0.781830
H	6.695102	-0.082756	2.577664

H	5.409734	-2.300202	-1.609350
H	4.200024	-3.135278	-0.588953
H	3.728300	-1.711384	-1.596695
H	4.628288	1.486149	3.330676
H	5.266081	2.453801	1.964370
H	3.531392	1.986549	2.006379
H	-4.198612	3.134521	-0.588959
H	-3.729415	1.710373	-1.597594
H	-5.410150	2.301178	-1.608551
H	-4.623483	-1.484110	3.331542
H	-5.268170	-2.452418	1.968902
H	-3.532529	-1.988523	2.003756
H	-1.229834	-3.819510	-2.733380
H	-2.786603	-4.156839	-1.903223
H	-2.759045	-3.128018	-3.335524
H	2.791830	4.154899	-1.905663
H	2.752893	3.126685	-3.338279
H	1.229512	3.823211	-2.727830

2b (simplified)

76

scf done: -1644.685868

C	2.925831	-1.080071	-0.017463
C	3.478737	0.260526	-0.103737
C	2.705909	1.370047	0.052964
N	1.341934	1.233757	0.474508
C	0.721069	-0.007988	0.096608
C	1.478594	-1.134778	-0.040274
C	-0.721113	0.007962	-0.096547
C	-1.478652	1.134760	0.040199
C	-2.925885	1.080044	0.017312
C	-3.478792	-0.260544	0.103711
C	-2.705954	-1.370078	-0.052847
N	-1.341956	-1.233824	-0.474337
C	-3.751716	2.208681	0.050013
N	-5.082955	2.269800	-0.360883
C	-5.574047	3.558849	-0.163085
C	-4.572425	4.309895	0.348690
N	-3.449944	3.497136	0.485193
C	-5.754252	1.282493	-1.180448
C	-2.274784	3.883536	1.235484
C	-1.181699	-1.546687	-1.899081
C	-3.177668	-2.745155	0.177015
C	-4.544892	-3.092749	0.129092
C	-4.965550	-4.400622	0.360796
C	-4.034097	-5.408720	0.636404
C	-2.674497	-5.084342	0.674034
C	-2.251582	-3.775470	0.443946
C	1.181789	1.546438	1.899306
C	3.177623	2.745148	-0.176758
C	4.544851	3.092729	-0.128841
C	4.965507	4.400627	-0.360411
C	4.034049	5.408760	-0.635872
C	2.674446	5.084394	-0.673492
C	2.251532	3.775497	-0.443538
C	3.751696	-2.208680	-0.050284
N	3.450024	-3.497129	-0.485568
C	4.572562	-4.309816	-0.349090
C	5.574133	-3.558723	0.162714
N	5.082952	-2.269716	0.360556
C	2.274926	-3.883588	-1.235926
C	5.754191	-1.282361	1.180109
H	4.365184	6.435165	-0.810936
H	-4.365233	-6.435106	0.811573
H	0.945034	-2.063225	-0.216621
H	4.498839	0.384391	-0.467738
H	5.285855	2.328127	0.114273
H	6.031286	4.640337	-0.313975
H	1.934760	5.860566	-0.888978
H	1.193279	3.512751	-0.482309
H	4.556559	-5.354206	-0.645677
H	6.593884	-3.826850	0.421421
H	1.741520	0.839362	2.543773
H	0.120867	1.495860	2.180101
H	1.552566	2.563838	2.097529
H	-0.945116	2.063238	0.216461
H	-4.498900	-0.384379	0.467707
H	-5.285892	-2.328178	-0.114135
H	-6.031326	-4.640343	0.314350
H	-1.934814	-5.860487	0.889634
H	-1.193331	-3.512715	0.482722
H	-4.556345	5.354301	0.645220
H	-6.593774	3.827041	-0.421819

H	-1.741373	-0.839690	-2.543685
H	-0.120751	-1.496147	-2.179789
H	-1.552461	-2.564111	-2.097206
H	-2.488208	4.823276	1.762118
H	-2.025086	3.110081	1.977110
H	-1.395898	4.037110	0.589673
H	6.453553	-1.794180	1.857421
H	6.319102	-0.548066	0.583583
H	5.009748	-0.737586	1.777626
H	2.488461	-4.823274	-1.762612
H	1.396032	-4.037292	-0.590158
H	2.025181	-3.110112	-1.977513
H	-6.453551	1.794368	-1.857783
H	-5.009840	0.737652	-1.777942
H	-6.319244	0.548252	-0.583935

2b$^+$ (simplified)

76

scf done: -1644.542110

C	2.866371	-1.141927	-0.048133
C	3.464787	0.174555	-0.188891
C	2.731946	1.307201	-0.005128
N	1.373759	1.213612	0.407074
C	0.706820	-0.012456	0.091307
C	1.455054	-1.182777	0.007918
C	-0.706882	0.012442	-0.091366
C	-1.455128	1.182757	-0.008014
C	-2.866442	1.141896	0.048026
C	-3.464843	-0.174586	0.188869
C	-2.731994	-1.307231	0.005141
N	-1.373812	-1.213644	-0.407082
C	-3.673709	2.318058	0.059798
N	-4.948273	2.432032	-0.441737
C	-5.416510	3.721196	-0.236641
C	-4.430406	4.419454	0.381705
N	-3.361489	3.555443	0.571013
C	-5.658169	1.431056	-1.224744
C	-2.176060	3.895906	1.338933
C	-1.168977	-1.690744	-1.785468
C	-3.265612	-2.668611	0.201570
C	-4.625962	-2.965577	-0.005995
C	-5.117194	-4.252827	0.207394
C	-4.258240	-5.275479	0.622887
C	-2.902446	-4.997286	0.821991
C	-2.410392	-3.709670	0.611182
C	1.168926	1.690661	1.785479
C	3.265589	2.668583	-0.201465
C	4.625937	2.965518	0.006151
C	5.117194	4.252773	-0.207152
C	4.258264	5.275462	-0.622606
C	2.902471	4.997300	-0.821757
C	2.410392	3.709680	-0.611034
C	3.673674	-2.318067	-0.059896
N	3.361594	-3.555476	-0.571157
C	4.430546	-4.419414	-0.381713
C	5.416568	-3.721065	0.236658
N	4.948243	-2.431917	0.441639
C	2.176287	-3.896051	-1.339217
C	5.658046	-1.430811	1.224563
H	4.643305	6.284525	-0.784912
H	-4.643262	-6.284539	0.785262
H	0.902214	-2.119047	-0.022501
H	4.480895	0.264540	-0.571037
H	5.299937	2.183632	0.362509
H	6.175311	4.464031	-0.036140
H	2.226340	5.789837	-1.151737
H	1.356506	3.482089	-0.781104
H	4.398328	-5.452519	-0.714337
H	6.405491	-4.031157	0.560500
H	1.734428	1.077307	2.511939
H	0.105796	1.636357	2.048450
H	1.507058	2.732625	1.872058
H	-0.902305	2.119040	0.022394
H	-4.480931	-0.264566	0.571066
H	-5.299981	-2.183722	-0.362382
H	-6.175312	-4.464111	0.036419
H	-2.226296	-5.789794	1.152003
H	-1.356505	-3.482054	0.781215
H	-4.398094	5.452545	0.714361
H	-6.405425	4.031372	-0.560424
H	-1.734492	-1.077427	-2.511950
H	-0.105849	-1.636429	-2.048443
H	-1.507090	-2.732717	-1.872005

H	-2.400268	4.774709	1.956356
H	-1.906151	3.057058	1.994024
H	-1.320014	4.128202	0.687599
H	6.312174	-1.944538	1.941170
H	6.270874	-0.776660	0.586894
H	4.934972	-0.815047	1.773255
H	2.400645	-4.774854	-1.956586
H	1.320179	-4.128400	-0.687985
H	1.906394	-3.057246	-1.994370
H	-6.312272	1.944906	-1.941286
H	-4.935160	0.815288	-1.773514
H	-6.271038	0.776894	-0.587126

$2b^{2+}$ (simplified)

76

scf done: -1644.296860

C	2.828770	-1.166054	-0.103863
C	3.454469	0.114465	-0.341580
C	2.751780	1.264048	-0.102283
N	1.422209	1.190439	0.318003
C	0.690954	-0.011981	0.087151
C	1.466020	-1.218857	0.081526
C	-0.690940	0.011893	-0.087057
C	-1.465969	1.218790	-0.081426
C	-2.828730	1.166036	0.103901
C	-3.454479	-0.114463	0.341579
C	-2.751811	-1.264066	0.102316
N	-1.422224	-1.190503	-0.317914
C	-3.655452	2.371003	0.065479
N	-4.805841	2.519464	-0.636272
C	-5.324871	3.779270	-0.407559
C	-4.476269	4.414223	0.449997
N	-3.452543	3.530416	0.738552
C	-5.402712	1.534945	-1.539907
C	-2.367094	3.813247	1.674042
C	-1.034454	-2.055574	-1.447391
C	-3.347222	-2.607223	0.243899
C	-4.669439	-2.857726	-0.163369
C	-5.234676	-4.121767	0.005512
C	-4.487636	-5.151764	0.584770
C	-3.171311	-4.913202	0.994917
C	-2.602879	-3.652544	0.822188
C	1.034490	2.055421	1.447567
C	3.347132	2.607226	-0.243902
C	4.669364	2.857780	0.163286
C	5.234541	4.121845	-0.005621
C	4.487426	5.151816	-0.584828
C	3.171086	4.913204	-0.994899
C	2.602716	3.652522	-0.822144
C	3.655536	-2.370993	-0.065489
N	3.452664	-3.530381	-0.738616
C	4.476422	-4.414166	-0.450107
C	5.325016	-3.779215	0.407461
N	4.805939	-2.519439	0.636238
C	2.367179	-3.813203	-1.674065
C	5.402783	-1.534922	1.539893
H	4.930944	6.140793	-0.717637
H	-4.931202	-6.140722	0.717560
H	0.951678	-2.164263	0.254016
H	4.467268	0.176332	-0.736122
H	5.248223	2.062259	0.637116
H	6.259501	4.307540	0.321888
H	2.592053	5.713173	-1.461330
H	1.584645	3.457322	-1.166510
H	4.519735	-5.410259	-0.882044
H	6.245404	-4.119385	0.874451
H	1.909068	2.260018	2.081565
H	0.283620	1.524808	2.047877
H	0.623443	3.021758	1.124665
H	-0.951578	2.164173	-0.253890
H	-4.467301	-0.176308	0.736063
H	-5.248237	-2.062182	-0.637237
H	-6.259624	-4.307423	-0.322056
H	-2.592336	-5.713191	1.461386
H	-1.584819	-3.457385	1.166610
H	-4.519550	5.410338	0.881887
H	-6.245235	4.119457	-0.874585
H	-1.909016	-2.260254	-2.081383
H	-0.283593	-1.524994	-2.047739
H	-0.623382	-3.021869	-1.124394
H	-2.752070	4.453472	2.477666
H	-2.004182	2.871936	2.101410
H	-1.541480	4.329868	1.164352

H	5.893868	-2.068089	2.363395
H	6.144463	-0.923838	1.006752
H	4.617238	-0.886168	1.942112
H	2.752089	-4.453539	-2.477631
H	1.541523	-4.329701	-1.164319
H	2.004346	-2.871899	-2.101522
H	-5.893693	2.068105	-2.363476
H	-4.617200	0.886094	-1.942032
H	-6.144486	0.923963	-1.006778

$2b^{3+}$ (simplified)

76

scf done: -1643.933447

C	2.765763	-1.256050	0.313739
C	3.495882	-0.107643	-0.083247
C	2.857619	0.999211	-0.621898
N	1.483511	0.969826	-0.806467
C	0.716803	-0.088841	-0.303507
C	1.373697	-1.217692	0.206482
C	-0.732848	0.042258	-0.244685
C	-1.341728	1.204242	0.252351
C	-2.724741	1.278687	0.425256
C	-3.496205	0.126206	0.125556
C	-2.905818	-1.021545	-0.377383
N	-1.543618	-1.027088	-0.641819
C	-3.379018	2.479411	0.959181
N	-4.466764	3.089246	0.428316
C	-4.812123	4.168001	1.215261
C	-3.920103	4.216735	2.248645
N	-3.041207	3.166085	2.078398
C	-5.177915	2.712779	-0.798266
C	-1.966340	2.840769	3.018021
C	-1.000960	-2.045828	-1.560134
C	-3.707494	-2.230679	-0.628089
C	-4.920675	-2.129735	-1.336274
C	-5.713182	-3.259778	-1.535168
C	-5.315491	-4.494548	-1.012691
C	-4.119025	-4.600624	-0.292579
C	-3.313908	-3.479891	-0.106219
C	0.869371	1.918133	-1.757746
C	3.628811	2.189972	-1.015634
C	3.246729	3.476410	-0.582440
C	4.023611	4.584496	-0.909899
C	5.180109	4.427750	-1.684158
C	5.566309	3.156048	-2.119360
C	4.802808	2.039522	-1.780058
C	3.466039	-2.415751	0.880149
N	3.324683	-3.707826	0.493879
C	4.179013	-4.499600	1.233838
C	4.855268	-3.674326	2.086834
N	4.405573	-2.390868	1.856548
C	2.461030	-4.215837	-0.573604
C	4.876933	-1.218243	2.602022
H	5.782858	5.300044	-1.945596
H	-5.941431	-5.376832	-1.162024
H	0.768162	-2.028951	0.608578
H	4.577543	-0.060935	0.025845
H	2.361575	3.605165	0.044447
H	3.735281	5.575934	-0.555070
H	6.464213	3.034771	-2.728429
H	5.100183	1.051144	-2.137467
H	4.245182	-5.575603	1.090170
H	5.611278	-3.898379	2.836041
H	1.622374	2.211281	-2.497483
H	0.497246	2.827409	-1.269712
H	0.035381	1.414091	-2.261142
H	-0.701993	2.038300	0.535903
H	-4.560136	0.089176	0.353236
H	-5.228346	-1.170382	-1.758220
H	-6.643460	-3.178698	-2.100740
H	-3.822978	-5.561178	0.133698
H	-2.398634	-3.564554	0.484126
H	-3.848627	4.903534	3.088831
H	-5.655306	4.812878	0.978150
H	-1.802856	-2.384387	-2.225144
H	-0.199170	-1.588638	-2.153243
H	-0.604928	-2.921313	-1.031020
H	-2.288786	3.118350	4.029367
H	-1.769197	1.762725	2.991506
H	-1.053030	3.399301	2.767477
H	5.095849	-1.524722	3.632418
H	5.791543	-0.816294	2.143463
H	4.096234	-0.449748	2.614132

H	2.971351	-5.048729	-1.073627
H	1.509409	-4.577006	-0.157774
H	2.277396	-3.421274	-1.305958
H	-5.545975	3.625239	-1.283600
H	-4.491824	2.196754	-1.479135
H	-6.030953	2.061785	-0.559776

2b^{4+} (simplified)

76

scf done: -1643.452391

C	2.842164	1.017582	-0.821434
C	3.505702	0.089753	-0.027990
C	2.820015	-0.812539	0.817534
N	1.441074	-0.825540	0.767365
C	0.749557	0.084163	-0.008294
C	1.419075	1.017363	-0.775200
C	-0.756784	0.033693	-0.015642
C	-1.486443	0.882454	0.790868
C	-2.910860	0.863738	0.745920
C	-3.511276	-0.065969	-0.093020
C	-2.762543	-0.969199	-0.883377
N	-1.387858	-0.849250	-0.873310
C	-3.735877	1.764982	1.568254
N	-4.817077	2.468131	1.141385
C	-5.344887	3.167705	2.199207
C	-4.580052	2.880413	3.300601
N	-3.589559	2.018765	2.894757
C	-5.369672	2.535155	-0.220334
C	-2.597940	1.439505	3.809213
C	-0.569016	-1.606963	-1.850481
C	-3.452554	-2.000115	-1.648502
C	-4.639472	-1.657519	-2.344778
C	-5.351964	-2.629092	-3.037817
C	-4.922031	-3.963752	-3.013854
C	-3.767545	-4.323259	-2.302049
C	-3.028209	-3.353486	-1.636030
C	0.683761	-1.849947	1.524040
C	3.584779	-1.669498	1.718534
C	4.745409	-2.311927	1.218265
C	5.530951	-3.095125	2.056251
C	5.202491	-3.215058	3.414089
C	4.076700	-2.556583	3.930077
C	3.263929	-1.800904	3.093007
C	3.600977	1.957624	-1.663333
N	3.403298	3.297342	-1.769392
C	4.335732	3.823744	-2.630161
C	5.116431	2.784758	-3.068235
N	4.656452	1.642908	-2.459229
C	2.413693	4.111571	-1.053568
C	5.242411	0.315061	-2.699382
H	5.831203	-3.816594	4.075063
H	-5.493541	-4.729059	-3.544695
H	0.835841	1.709181	-1.382519
H	4.592481	0.089479	0.040351
H	5.003762	-2.237380	0.159429
H	6.404103	-3.615058	1.656796
H	3.844160	-2.627982	4.994522
H	2.419009	-1.261794	3.523706
H	4.386046	4.887152	-2.859136
H	5.953087	2.778051	-3.765464
H	1.330402	-2.718048	1.688623
H	-0.194784	-2.156569	0.949710
H	0.361944	-1.455295	2.496208
H	-0.952151	1.577293	1.438092
H	-4.593166	-0.189869	-0.105000
H	-4.975582	-0.618900	-2.383734
H	-6.246668	-2.353091	-3.599574
H	-3.455855	-5.368932	-2.261365
H	-2.157813	-3.660752	-1.053984
H	-4.677979	3.214675	4.332371
H	-6.214107	3.815593	2.095121
H	-1.206823	-1.904624	-2.688711
H	0.234264	-0.960251	-2.216637
H	-0.140327	-2.509521	-1.395168
H	-3.052993	1.346049	4.803490
H	-2.313788	0.439209	3.461925
H	-1.717076	2.093552	3.881513
H	5.612095	0.280401	-3.731947
H	6.084122	0.143529	-2.013105
H	4.478119	-0.459780	-2.571525
H	2.843223	5.105712	-0.876013
H	1.501623	4.220619	-1.657828
H	2.186776	3.652461	-0.084274

H	-5.781385	3.539821	-0.379118
H	-4.576378	2.357324	-0.955127
H	-6.176050	1.796934	-0.337313

***syn*-2cS** (B3LYP-D3BJ)/def2SVP)

79

scf done: -1758.978507

C	4.857514	4.651899	0.042111
C	3.482674	4.552704	-0.196428
C	2.831472	3.324950	-0.085815
C	3.538011	2.154662	0.263245
C	4.921191	2.274107	0.513947
C	5.571335	3.501725	0.397061
C	2.842805	0.860312	0.352323
N	1.465149	0.901439	0.715536
C	0.655740	-0.213077	0.363580
C	1.250502	-1.483407	0.088254
C	2.700248	-1.555267	-0.107237
C	3.434868	-0.318137	0.024334
C	0.394178	-2.594804	0.022166
C	-0.992427	-2.460874	0.072363
C	-1.596673	-1.204044	0.222559
C	-0.743946	-0.073969	0.367535
N	-1.336384	1.214862	0.504369
C	-2.582252	1.403552	-0.169147
C	-3.423442	0.350404	-0.342157
C	-3.046153	-0.981553	0.090002
C	-1.278128	1.826518	1.837007
C	0.076827	2.435601	2.157399
C	1.227111	1.444218	2.065294
C	-3.983330	-1.995949	0.308483
N	-5.202099	-2.198034	-0.332233
C	-5.846475	-3.299917	0.226256
C	-5.047784	-3.799179	1.198484
N	-3.904994	-3.005282	1.265272
C	-5.599109	-1.564260	-1.567468
C	-3.011698	-2.965971	2.403936
C	3.368005	-2.688775	-0.594309
N	2.946339	-3.607928	-1.549676
C	3.954178	-4.548076	-1.759872
C	5.004521	-4.220390	-0.969808
N	4.654001	-3.084677	-0.246563
C	1.905461	-3.328147	-2.523134
C	5.394171	-2.567099	0.882709
C	-2.857088	2.772525	-0.638639
C	-4.168717	3.252063	-0.840936
C	-4.399751	4.548426	-1.296909
C	-3.329023	5.412667	-1.554856
C	-2.023552	4.957241	-1.347075
C	-1.789844	3.659510	-0.892781
H	-3.512422	6.430985	-1.905921
H	5.366063	5.615538	-0.038831
H	-6.810912	-3.641350	-0.137563
H	-5.194854	-4.644430	1.864037
H	4.460469	-0.284013	-0.344139
H	1.762759	3.244406	-0.287054
H	2.911569	5.441664	-0.477617
H	6.643641	3.565990	0.601018
H	5.483781	1.391700	0.827650
H	0.825181	-3.592474	-0.072361
H	-1.616971	-3.348070	-0.053344
H	-4.376359	0.529738	-0.838294
H	-5.018743	2.604190	-0.615338
H	-5.427074	4.894346	-1.440337

H	-1.175981	5.619188	-1.544819
H	-0.773201	3.296050	-0.738876
H	-1.545331	1.076337	2.610879
H	-2.048041	2.609933	1.876622
H	0.270002	3.279244	1.477390
H	0.048333	2.842007	3.183865
H	2.153553	1.940626	2.384059
H	1.055603	0.605488	2.770626
H	3.836446	-5.361338	-2.469821
H	5.964732	-4.709721	-0.836550
H	-4.710101	-1.389521	-2.192427
H	-6.291619	-2.226077	-2.105860
H	-6.098954	-0.595279	-1.402022
H	-2.870446	-1.919628	2.716499
H	-3.460420	-3.536597	3.228443
H	-2.016990	-3.374457	2.169236
H	5.999902	-3.373245	1.321682
H	6.060644	-1.734315	0.605485
H	4.680203	-2.194630	1.631792
H	2.204122	-3.744689	-3.495241
H	0.930586	-3.742674	-2.229454
H	1.788230	-2.238744	-2.615204

***trans*-2cS** (B3LYP-D3BJ)/def2SVP)

79

scf done: -1758.977074

C	4.660960	4.639709	-1.929791
C	5.307261	3.914921	-0.923635
C	4.704957	2.783513	-0.371496
C	3.445395	2.339711	-0.819059
C	2.807959	3.079560	-1.834910
C	3.407547	4.213033	-2.382775
C	2.826941	1.115674	-0.266793
N	1.448620	1.163796	0.053472
C	0.705654	-0.044573	0.065396
C	1.416539	-1.273072	0.181469
C	2.883506	-1.252617	0.329511
C	3.529481	-0.037037	-0.128001
C	0.691824	-2.469116	0.078254
C	-0.691784	-2.469146	-0.077821
C	-1.416534	-1.273148	-0.181245
C	-0.705690	-0.044603	-0.065374
N	-1.448728	1.163703	-0.053605
C	-2.827009	1.115553	0.266792
C	-3.529515	-0.037192	0.128156
C	-2.883500	-1.252777	-0.329347
C	0.949200	2.312337	0.812954
C	-0.000096	3.193380	0.000057
C	-0.949321	2.312340	-0.812930
C	-3.601204	-2.324860	-0.861605
N	-3.192156	-3.191642	-1.876981
C	-4.216617	-4.095241	-2.154494
C	-5.260874	-3.809278	-1.343184
N	-4.897043	-2.729242	-0.539792
C	-2.111750	-2.894045	-2.793275
C	-5.609017	-2.328278	0.649789
C	-3.445483	2.339595	0.819048
C	-4.705013	2.783400	0.371394
C	-5.307359	3.914795	0.923506
C	-4.661130	4.639565	1.929728
C	-3.407756	4.212884	2.382799
C	-2.808126	3.079416	1.834957
C	3.601314	-2.324716	0.861645

N	4.897053	-2.729141	0.539527
C	5.261063	-3.809153	1.342860
C	4.216973	-4.095095	2.154405
N	3.192514	-3.191431	1.877159
C	5.608745	-2.328200	-0.650238
C	2.112178	-2.893931	2.793562
H	-5.128123	5.530831	2.355596
H	5.127914	5.530985	-2.355680
H	1.810677	2.894956	1.169633
H	0.427569	1.934062	1.708833
H	-0.565403	3.847774	0.680106
H	0.565169	3.847855	-0.679950
H	-1.810815	2.894943	-1.169589
H	-0.427636	1.934177	-1.708822
H	1.228327	-3.419242	0.116158
H	-1.228267	-3.419289	-0.115608
H	-4.584196	-0.015248	0.399096
H	4.584202	-0.015066	-0.398790
H	6.228708	-4.291984	1.244349
H	4.111787	-4.857205	2.920806
H	-4.111275	-4.857355	-2.920869
H	-6.228553	-4.292086	-1.244888
H	-5.200420	2.236702	-0.434368
H	-1.844672	2.733200	2.211536
H	-6.283091	4.242386	0.554876
H	-2.897576	4.763696	3.177532
H	5.200433	2.236816	0.434226
H	1.844477	2.733343	-2.211413
H	6.283019	4.242498	-0.555061
H	2.897315	4.763867	-3.177459
H	-6.299953	-3.130236	0.945620
H	-4.890502	-2.155002	1.465975
H	-6.191403	-1.402792	0.504524
H	-2.299008	-3.409966	-3.744949
H	-2.072071	-1.806951	-2.968639
H	-1.131467	-3.196706	-2.395437
H	6.299485	-3.130235	-0.946324
H	4.890010	-2.154767	-1.466191
H	6.191300	-1.402805	-0.505076
H	2.299877	-3.409344	3.745426
H	2.072086	-1.806780	2.968438
H	1.131932	-3.197181	2.396077

***syn*-2cS$^+$** (B3LYP-D3BJ)/def2SVP)

79

scf done: -1758.832233

C	4.807414	4.706742	-0.307357
C	3.436045	4.583627	-0.553270
C	2.796438	3.358317	-0.372209
C	3.517279	2.226493	0.056676
C	4.895578	2.365447	0.306728
C	5.533916	3.591655	0.122309
C	2.835776	0.928485	0.214115
N	1.503127	0.956469	0.679023
C	0.678112	-0.172454	0.427675
C	1.291241	-1.452679	0.199726
C	2.697097	-1.498195	-0.080071
C	3.422804	-0.258463	-0.116588
C	0.451561	-2.592627	0.231143
C	-0.923490	-2.475234	0.286282
C	-1.553640	-1.207755	0.335999
C	-0.713834	-0.050262	0.446365
N	-1.329775	1.227880	0.567726

C	-2.592783	1.388969	-0.054272
C	-3.396460	0.306363	-0.252870
C	-2.964870	-1.017710	0.121804
C	-1.187153	1.936938	1.850935
C	0.156800	2.616809	2.024351
C	1.318651	1.642462	1.975272
C	-3.894857	-2.096647	0.190729
N	-4.917571	-2.379890	-0.679184
C	-5.624271	-3.483413	-0.224854
C	-5.032642	-3.901565	0.924703
N	-3.976401	-3.040577	1.185432
C	-5.143287	-1.728215	-1.958804
C	-3.229248	-3.005651	2.431855
C	3.374353	-2.695759	-0.480270
N	3.005080	-3.601745	-1.440071
C	3.977502	-4.584574	-1.548228
C	4.968689	-4.275607	-0.669824
N	4.589978	-3.116567	-0.012397
C	1.909773	-3.427182	-2.383167
C	5.324486	-2.500241	1.081912
C	-2.960821	2.762474	-0.444675
C	-4.298664	3.202168	-0.439260
C	-4.625784	4.497389	-0.838729
C	-3.623929	5.385919	-1.243204
C	-2.290271	4.965568	-1.243190
C	-1.960796	3.670401	-0.845826
H	-3.881357	6.402059	-1.549855
H	5.306998	5.668079	-0.445344
H	-6.477435	-3.882213	-0.765564
H	-5.280394	-4.725536	1.587121
H	4.422824	-0.238079	-0.549840
H	1.730822	3.254950	-0.581213
H	2.862820	5.448394	-0.895781
H	6.602994	3.682353	0.328173
H	5.463399	1.507729	0.674210
H	0.898528	-3.586340	0.179800
H	-1.533443	-3.377406	0.215295
H	-4.372999	0.464306	-0.707386
H	-5.085976	2.529803	-0.091321
H	-5.668847	4.821998	-0.820633
H	-1.501498	5.651804	-1.560868
H	-0.923481	3.333014	-0.856999
H	-1.355275	1.221506	2.680922
H	-1.989814	2.683425	1.905047
H	0.286198	3.392612	1.255187
H	0.171438	3.122201	3.004659
H	2.253023	2.172995	2.195461
H	1.192140	0.871861	2.760527
H	3.887755	-5.408003	-2.250563
H	5.901070	-4.786311	-0.447971
H	-4.183436	-1.377705	-2.359746
H	-5.584595	-2.453615	-2.654616
H	-5.823049	-0.868310	-1.860931
H	-3.000580	-1.960242	2.678521
H	-3.845373	-3.444955	3.226944
H	-2.279900	-3.555526	2.355133
H	5.870677	-3.279198	1.629909
H	6.038388	-1.749454	0.712105
H	4.611833	-2.006554	1.754525
H	2.237561	-3.769264	-3.373754
H	1.016890	-3.988920	-2.075955
H	1.650765	-2.362405	-2.430376

***trans*-2cS**+ (B3LYP-D3BJ)/def2SVP)

79

scf done: -1758.834249

C	4.809938	4.851156	-1.046343
C	5.283682	4.050858	-0.003428
C	4.630731	2.859450	0.320043
C	3.493413	2.448268	-0.396162
C	3.029780	3.259218	-1.448670
C	3.682533	4.448874	-1.770598
C	2.827519	1.161822	-0.085772
N	1.447881	1.172473	0.168024
C	0.701553	-0.031091	0.074401
C	1.423636	-1.271156	0.137702
C	2.862145	-1.257278	0.206424
C	3.526085	-0.006276	-0.061363
C	0.687419	-2.475040	0.052149
C	-0.687471	-2.475038	-0.051921
C	-1.423679	-1.271163	-0.137681
C	-0.701592	-0.031091	-0.074541
N	-1.447939	1.172421	-0.168293
C	-2.827533	1.161787	0.085763
C	-3.526099	-0.006301	0.061536
C	-2.862198	-1.257326	-0.206356
C	0.870170	2.311143	0.892868
C	-0.000073	3.190831	0.000008
C	-0.870245	2.311185	-0.892960
C	-3.622891	-2.426529	-0.500110
N	-3.397709	-3.328022	-1.514060
C	-4.388610	-4.299320	-1.500898
C	-5.246357	-3.993952	-0.492414
N	-4.770187	-2.848050	0.126989
C	-2.427914	-3.155576	-2.582144
C	-5.319194	-2.282022	1.347542
C	-3.493402	2.448254	0.396195
C	-4.630565	2.859569	-0.320180
C	-5.283516	4.050977	0.003292
C	-4.809922	4.851136	1.046380
C	-3.682675	4.448717	1.770805
C	-3.029924	3.259057	1.448874
C	3.622873	-2.426519	0.500089
N	4.770034	-2.848064	-0.127185
C	5.246463	-3.993786	0.492329
C	4.388923	-4.299072	1.501027
N	3.397948	-3.327863	1.514204
C	5.318905	-2.282086	-1.347834
C	2.428100	-3.155577	2.582269
H	-5.318163	5.784907	1.296728
H	5.318187	5.784927	-1.296673
H	1.689625	2.891219	1.336812
H	0.273468	1.908521	1.727665
H	-0.624202	3.844598	0.625420
H	0.624012	3.844708	-0.625335
H	-1.689707	2.891264	-1.336885
H	-0.273483	1.908686	-1.727771
H	1.221408	-3.426188	0.054687
H	-1.221487	-3.426174	-0.054278
H	-4.598931	0.039001	0.239422
H	4.598954	0.038972	-0.239095
H	6.145136	-4.499858	0.152408
H	4.406080	-5.112839	2.219785
H	-4.405567	-5.113207	-2.219524
H	-6.145053	-4.500046	-0.152588

H -4.989175 2.244028 -1.148299

H -2.170760 2.930603 2.036406

H -6.161442 4.360582 -0.568449

H -3.317373 5.061305 2.598423

H 4.989463 2.243827 1.148048

H 2.170491 2.930862 -2.036070

H 6.161726 4.360360 0.568186

H 3.317104 5.061569 -2.598081

H -5.810044 -3.079176 1.921040

H -4.503423 -1.854346 1.944838

H -6.054127 -1.491068 1.133902

H -2.824915 -3.610676 -3.498809

H -2.267029 -2.081358 -2.745145

H -1.459108 -3.612465 -2.333059

H 5.808144 -3.079576 -1.922261

H 4.503267 -1.852904 -1.944192

H 6.055131 -1.492316 -1.134222

H 2.825701 -3.609470 3.499277

H 2.266081 -2.081407 2.744369

H 1.459770 -3.613745 2.333665

***syn*-2cS**$^{2+}$ (B3LYP-D3BJ)/def2SVP)

79

scf done: -1758.581393

C 4.694766 4.827125 -0.673244

C 3.322812 4.673891 -0.901357

C 2.703405 3.452312 -0.646037

C 3.451231 2.359730 -0.164627

C 4.830743 2.524178 0.058554

C 5.446231 3.749846 -0.192962

C 2.794986 1.059638 0.059381

N 1.514481 1.064423 0.619034

C 0.697360 -0.091662 0.464875

C 1.362545 -1.367301 0.296653

C 2.718286 -1.372001 -0.055809

C 3.395442 -0.129930 -0.269352

C 0.569221 -2.557086 0.425145

C -0.791928 -2.493874 0.467395

C -1.478566 -1.234473 0.398498

C -0.687282 -0.026494 0.494225

N -1.386010 1.210281 0.620213

C -2.656748 1.307378 0.044533

C -3.380302 0.170975 -0.215724

C -2.845455 -1.118867 0.102187

C -1.163031 2.031247 1.830595

C 0.139923 2.801165 1.836383

C 1.346741 1.886899 1.839534

C -3.724108 -2.280896 0.026620

N -4.468339 -2.661327 -1.040822

C -5.224040 -3.769270 -0.704167

C -4.932996 -4.075883 0.592230

N -4.010809 -3.143894 1.033784

C -4.461639 -2.030976 -2.360334

C -3.500386 -3.065799 2.400723

C 3.454281 -2.607956 -0.313560

N 3.210780 -3.528049 -1.279548

C 4.179283 -4.514301 -1.228916

C 5.038126 -4.181929 -0.222313

N 4.577202 -3.003426 0.332458

C 2.153326 -3.453406 -2.284334

C 5.205477 -2.300441 1.450442

C -3.175587 2.656916 -0.239349

C -4.534381 2.970979 -0.050293

C	-5.014494	4.243443	-0.358269
C	-4.147297	5.219179	-0.860238
C	-2.794563	4.917490	-1.052320
C	-2.309787	3.649012	-0.740801
H	-4.525053	6.215429	-1.099408
H	5.178131	5.786495	-0.869090
H	-5.898000	-4.246358	-1.410642
H	-5.310532	-4.866189	1.235379
H	4.369663	-0.113728	-0.756527
H	1.638256	3.322894	-0.844220
H	2.737189	5.510506	-1.288256
H	6.515160	3.869756	-0.005149
H	5.416862	1.694411	0.459202
H	1.064636	-3.529472	0.436242
H	-1.369737	-3.419575	0.478475
H	-4.371749	0.270192	-0.654009
H	-5.210462	2.223855	0.370823
H	-6.068039	4.480010	-0.195812
H	-2.118266	5.674963	-1.454190
H	-1.259881	3.403405	-0.908128
H	-1.200071	1.357380	2.707465
H	-2.010027	2.721041	1.915774
H	0.182420	3.492786	0.982544
H	0.173427	3.413315	2.752276
H	2.263550	2.474221	1.959864
H	1.285150	1.195095	2.700448
H	4.183448	-5.357954	-1.913736
H	5.928938	-4.685445	0.143693
H	-3.515728	-1.495294	-2.499394
H	-4.558878	-2.810600	-3.126435
H	-5.297928	-1.323662	-2.454018
H	-3.154652	-2.043786	2.594053
H	-4.310064	-3.317303	3.097491
H	-2.664062	-3.764464	2.544673
H	5.641292	-3.038747	2.135082
H	5.994933	-1.628575	1.084931
H	4.444961	-1.713426	1.977824
H	2.565164	-3.751334	-3.257194
H	1.319593	-4.117866	-2.018561
H	1.789644	-2.421184	-2.341100

***trans*-2cS**$^{2+}$ (B3LYP-D3BJ)/def2SVP)

79

scf done: -1758.586909

C	4.930701	4.903433	-0.255011
C	5.262770	3.994632	0.753535
C	4.572023	2.786110	0.863071
C	3.531045	2.481815	-0.030076
C	3.208655	3.398102	-1.047739
C	3.907761	4.599748	-1.159683
C	2.825332	1.182484	0.059692
N	1.455286	1.175661	0.263337
C	0.695161	-0.016183	0.078333
C	1.428853	-1.263862	0.081444
C	2.830680	-1.246916	0.073717
C	3.519348	0.004815	-0.037245
C	0.679339	-2.485984	0.026849
C	-0.679223	-2.486019	-0.026658
C	-1.428801	-1.263940	-0.081336
C	-0.695171	-0.016214	-0.078264
N	-1.455367	1.175592	-0.263218
C	-2.825415	1.182326	-0.059646
C	-3.519372	0.004619	0.037275

C	-2.830626	-1.247063	-0.073646
C	0.814515	2.309254	0.950002
C	-0.000136	3.179513	0.000311
C	-0.814608	2.309359	-0.949616
C	-3.623750	-2.469133	-0.138009
N	-3.642047	-3.377681	-1.146755
C	-4.578958	-4.356681	-0.866561
C	-5.153824	-4.032386	0.326626
N	-4.553555	-2.866934	0.765570
C	-2.866723	-3.299488	-2.381732
C	-4.861913	-2.196226	2.027771
C	-3.531242	2.481612	0.030048
C	-4.571972	2.785929	-0.863373
C	-5.262820	3.994404	-0.753918
C	-4.931100	4.903120	0.254817
C	-3.908410	4.599403	1.159762
C	-3.209202	3.397808	1.047903
C	3.623913	-2.468922	0.138001
N	4.553662	-2.866608	-0.765701
C	5.154082	-4.032026	-0.326880
C	4.579355	-4.356452	0.866333
N	3.642386	-3.377550	1.146676
C	4.861934	-2.195816	-2.027876
C	2.867292	-3.299533	2.381809
H	-5.474023	5.846632	0.340959
H	5.473545	5.846985	-0.341215
H	1.594156	2.895297	1.450708
H	0.171495	1.885380	1.737677
H	-0.661346	3.833749	0.584643
H	0.660936	3.834038	-0.583853
H	-1.594243	2.895426	-1.450298
H	-0.171474	1.885678	-1.737298
H	1.215378	-3.436002	0.018610
H	-1.215214	-3.436062	-0.018309
H	-4.598047	0.046502	0.174939
H	4.598009	0.046753	-0.174996
H	5.931189	-4.532976	-0.897913
H	4.765507	-5.189191	1.539042
H	-4.765029	-5.189359	-1.539369
H	-5.930982	-4.533406	0.897527
H	-4.824308	2.079647	-1.657411
H	-2.435997	3.146837	1.776499
H	-6.061703	4.228569	-1.460571
H	-3.662755	5.298244	1.962190
H	4.824619	2.079772	1.656978
H	2.435260	3.147157	-1.776142
H	6.061843	4.228775	1.459979
H	3.661828	5.298655	-1.961970
H	-5.079708	-2.955571	2.789380
H	-3.996253	-1.601140	2.340123
H	-5.733613	-1.536711	1.910973
H	-3.493579	-3.636431	-3.217131
H	-2.564227	-2.258814	-2.547138
H	-1.969117	-3.931009	-2.319869
H	5.079147	-2.955117	-2.789698
H	3.996452	-1.600280	-2.339855
H	5.733974	-1.536725	-1.911210
H	3.494778	-3.635137	3.217282
H	2.563532	-2.259137	2.546609
H	1.970478	-3.932234	2.320573

***syn*-2cS**$^{3+}$ (B3LYP-D3BJ)/def2SVP)

79

scf done: -1758.208576

C	4.625985	4.973830	-0.611222
C	3.261514	4.803742	-0.880461
C	2.648258	3.581294	-0.626412
C	3.399545	2.500613	-0.111211
C	4.776374	2.679698	0.143189
C	5.379774	3.911375	-0.098140
C	2.761719	1.198917	0.094668
N	1.494494	1.143579	0.650704
C	0.710837	-0.028274	0.514649
C	1.414790	-1.262986	0.286082
C	2.804608	-1.225405	-0.009124
C	3.422298	0.013689	-0.219540
C	0.681702	-2.485653	0.222626
C	-0.681678	-2.485642	0.222632
C	-1.414756	-1.262968	0.286098
C	-0.710788	-0.028266	0.514658
N	-1.494437	1.143601	0.650690
C	-2.761640	1.198938	0.094639
C	-3.422235	0.013716	-0.219578
C	-2.804580	-1.225387	-0.009117
C	-1.248207	2.073589	1.789632
C	0.000041	2.914745	1.689244
C	1.248259	2.073546	1.789650
C	-3.596836	-2.451488	-0.201317
N	-4.235312	-2.823985	-1.334443
C	-4.932760	-3.992300	-1.099786
C	-4.714426	-4.337522	0.203933
N	-3.888201	-3.373226	0.746654
C	-4.210663	-2.124123	-2.623000
C	-3.451777	-3.348251	2.144587
C	3.596830	-2.451541	-0.201346
N	4.235210	-2.824157	-1.334473
C	4.932533	-3.992552	-1.099802
C	4.714201	-4.337692	0.203936
N	3.888112	-3.373280	0.746655
C	4.210679	-2.124304	-2.623033
C	3.451727	-3.348088	2.144595
C	-3.399484	2.500632	-0.111219
C	-4.776313	2.679702	0.143187
C	-5.379726	3.911372	-0.098138
C	-4.625950	4.973839	-0.611216
C	-3.261477	4.803768	-0.880453
C	-2.648207	3.581325	-0.626407
H	-5.103764	5.936403	-0.805863
H	5.103786	5.936400	-0.805869
H	-5.522392	-4.481022	-1.872118
H	-5.086195	-5.177991	0.785548
H	4.437576	0.078798	-0.608059
H	1.593805	3.438449	-0.868727
H	2.682539	5.627924	-1.301748
H	6.440634	4.048985	0.119639
H	5.366304	1.865702	0.569655
H	1.220127	-3.424077	0.084673
H	-1.220116	-3.424058	0.084689
H	-4.437508	0.078864	-0.608099
H	-5.366235	1.865699	0.569651
H	-6.440589	4.048969	0.119640
H	-2.682509	5.627957	-1.301737
H	-1.593754	3.438495	-0.868727
H	-1.204578	1.444919	2.695803
H	-2.134965	2.707878	1.874033

H	0.000062	3.524165	0.774946
H	0.000045	3.615289	2.539555
H	2.135029	2.707812	1.874097
H	1.204580	1.444875	2.695820
H	5.522127	-4.481333	-1.872126
H	5.085873	-5.178199	0.785559
H	-3.322782	-1.484235	-2.674664
H	-4.170583	-2.867810	-3.428878
H	-5.116746	-1.512708	-2.739839
H	-3.165323	-2.326158	2.416999
H	-4.284262	-3.671065	2.782673
H	-2.598101	-4.025045	2.290915
H	2.597618	-4.024336	2.290925
H	4.284015	-3.671473	2.782648
H	3.165943	-2.325817	2.417051
H	5.116741	-1.512830	-2.739755
H	4.170749	-2.867999	-3.428909
H	3.322770	-1.484467	-2.674808

***trans*-2cS^{3+}** (B3LYP-D3BJ)/def2SVP)

79

scf done: -1758.222536

C	5.044006	4.855720	0.040776
C	5.354280	3.863241	0.976997
C	4.629244	2.672211	0.999918
C	3.563569	2.477575	0.099628
C	3.263020	3.478333	-0.847077
C	4.006155	4.656804	-0.877304
C	2.830735	1.199863	0.095145
N	1.464267	1.184544	0.259677
C	0.709691	0.009183	0.059435
C	1.424481	-1.239971	0.018140
C	2.838508	-1.229089	-0.000716
C	3.518976	-0.000674	-0.037519
C	0.680463	-2.461835	-0.012584
C	-0.680445	-2.461826	0.012712
C	-1.424449	-1.239953	-0.017963
C	-0.709648	0.009200	-0.059190
N	-1.464227	1.184568	-0.259377
C	-2.830697	1.199855	-0.095019
C	-3.518957	-0.000689	0.037567
C	-2.838463	-1.229090	0.000838
C	0.812633	2.332394	0.942762
C	-0.000050	3.203029	0.000546
C	-0.812513	2.332618	-0.942051
C	-3.636940	-2.468666	0.016926
N	-3.763752	-3.360729	-0.992951
C	-4.663321	-4.341274	-0.622008
C	-5.095891	-4.034231	0.636898
N	-4.452627	-2.872887	1.016644
C	-3.105722	-3.293082	-2.299514
C	-4.652966	-2.200695	2.304697
C	-3.563597	2.477551	-0.099631
C	-4.628916	2.672224	-1.000324
C	-5.354021	3.863221	-0.977560
C	-5.044166	4.855609	-0.041109
C	-4.006668	4.656645	0.877360
C	-3.263460	3.478212	0.847299
C	3.636994	-2.468643	-0.016954
N	4.452708	-2.872624	-1.016749
C	5.095902	-4.034103	-0.637298
C	4.663220	-4.341511	0.621478
N	3.763663	-3.361036	0.992650

C	4.653232	-2.199911	-2.304502
C	3.105695	-3.293736	2.299263
H	-5.620341	5.783032	-0.019144
H	5.620129	5.783173	0.018687
H	1.597771	2.908208	1.444701
H	0.169918	1.895814	1.722159
H	-0.651471	3.857655	0.594007
H	0.651204	3.858074	-0.592635
H	-1.597586	2.908526	-1.443981
H	-0.169638	1.896269	-1.721442
H	1.220178	-3.408958	-0.042475
H	-1.220187	-3.408936	0.042570
H	-4.602618	0.042826	0.139403
H	4.602624	0.042847	-0.139477
H	5.802861	-4.544651	-1.287409
H	4.928323	-5.165541	1.279958
H	-4.928527	-5.165091	-1.280713
H	-5.802847	-4.544922	1.286899
H	-4.868656	1.902867	-1.737855
H	-2.488644	3.307166	1.596857
H	-6.165106	4.018643	-1.691678
H	-3.787765	5.418650	1.628117
H	4.869296	1.902799	1.737289
H	2.487937	3.307320	-1.596366
H	6.165637	4.018630	1.690814
H	3.786923	5.418878	-1.627897
H	-4.727090	-2.959676	3.093808
H	-3.799978	-1.544542	2.510268
H	-5.579147	-1.608986	2.281776
H	-3.838965	-3.529876	-3.081320
H	-2.722916	-2.279016	-2.460179
H	-2.278061	-4.014713	-2.347071
H	4.729919	-2.958716	-3.093528
H	3.799093	-1.545574	-2.511130
H	5.578140	-1.606257	-2.280464
H	3.839363	-3.529082	3.081117
H	2.721396	-2.280169	2.459478
H	2.279113	-4.016573	2.347220

***syn*-2cS**$^{4+}$ (B3LYP-D3BJ)/def2SVP)

79

scf done: -1757.719542

C	4.812689	4.900391	-0.593897
C	3.428963	4.804669	-0.824069
C	2.762785	3.617366	-0.562518
C	3.477424	2.482295	-0.088689
C	4.880631	2.589427	0.114281
C	5.532910	3.792423	-0.120816
C	2.796303	1.221120	0.112910
N	1.526077	1.169558	0.636018
C	0.727112	0.013019	0.502045
C	1.405308	-1.216399	0.231045
C	2.834138	-1.206312	-0.009278
C	3.466013	-0.000825	-0.167818
C	0.683825	-2.425376	0.072959
C	-0.683818	-2.425387	0.072967
C	-1.405315	-1.216422	0.231068
C	-0.727133	0.013002	0.502065
N	-1.526120	1.169527	0.636073
C	-2.796349	1.221077	0.112978
C	-3.466031	-0.000869	-0.167802
C	-2.834148	-1.206352	-0.009257
C	-1.244170	2.157566	1.732125

C	-0.000030	2.990546	1.573652
C	1.244149	2.157611	1.732066
C	-3.612502	-2.452174	-0.189961
N	-4.307015	-2.808356	-1.296828
C	-4.969303	-3.991017	-1.049685
C	-4.679100	-4.358018	0.237304
N	-3.837027	-3.397757	0.754111
C	-4.374795	-2.091178	-2.578905
C	-3.339597	-3.403629	2.135526
C	3.612521	-2.452123	-0.189978
N	4.306955	-2.808355	-1.296882
C	4.969366	-3.990934	-1.049695
C	4.679269	-4.357873	0.237338
N	3.837177	-3.397630	0.754142
C	4.374563	-2.091277	-2.579018
C	3.339964	-3.403387	2.135628
C	-3.477481	2.482250	-0.088630
C	-4.880689	2.589369	0.114320
C	-5.532971	3.792366	-0.120771
C	-4.812750	4.900341	-0.593834
C	-3.429022	4.804628	-0.823991
C	-2.762840	3.617326	-0.562442
H	-5.332539	5.840847	-0.793556
H	5.332475	5.840897	-0.793622
H	-5.589331	-4.478613	-1.800411
H	-5.015283	-5.213524	0.821070
H	4.508960	0.055493	-0.478015
H	1.698987	3.531566	-0.788125
H	2.885681	5.662136	-1.226272
H	6.605310	3.877732	0.066302
H	5.450155	1.747860	0.513422
H	1.224840	-3.351465	-0.123186
H	-1.224817	-3.351484	-0.123183
H	-4.508968	0.055443	-0.478036
H	-5.450214	1.747794	0.513443
H	-6.605374	3.877668	0.066336
H	-2.885741	5.662103	-1.226179
H	-1.699038	3.531532	-0.788032
H	-1.182337	1.556204	2.654007
H	-2.131454	2.789962	1.813146
H	-0.000066	3.554149	0.630619
H	-0.000022	3.736380	2.385180
H	2.131418	2.790036	1.813025
H	1.182388	1.556262	2.653962
H	5.589339	-4.478577	-1.800437
H	5.015578	-5.213303	0.821143
H	-3.512937	-1.421762	-2.675996
H	-4.354138	-2.825179	-3.394696
H	-5.309707	-1.515600	-2.638094
H	-2.950353	-2.411970	2.391266
H	-4.169864	-3.645406	2.812428
H	-2.550234	-4.160055	2.247918
H	2.551367	-4.160546	2.248442
H	4.170553	-3.644051	2.812536
H	2.949746	-2.412014	2.390996
H	5.309452	-1.515676	-2.638360
H	4.353833	-2.825334	-3.394757
H	3.512681	-1.421886	-2.676061

***trans*-2cS^{4+}** (B3LYP-D3BJ)/def2SVP)

79

scf done: -1757.734721

C	5.257797	4.748821	0.128944

C	5.579237	3.666558	0.962412
C	4.793127	2.520621	0.947507
C	3.632976	2.461022	0.132700
C	3.324356	3.559886	-0.709405
C	4.140128	4.684332	-0.717598
C	2.850337	1.234333	0.102394
N	1.485411	1.228985	0.194708
C	0.726057	0.062511	0.008145
C	1.412019	-1.189222	-0.042050
C	2.854185	-1.199294	-0.071211
C	3.531601	-0.007292	-0.014067
C	0.684041	-2.408268	0.014534
C	-0.678343	-2.406208	0.129409
C	-1.410114	-1.190063	0.071011
C	-0.726929	0.056606	-0.056968
N	-1.492655	1.210889	-0.290169
C	-2.850849	1.227971	-0.135338
C	-3.528057	-0.005494	0.070788
C	-2.851753	-1.197099	0.130221
C	0.816936	2.413135	0.821351
C	-0.000925	3.242310	-0.147226
C	-0.845305	2.342109	-1.027118
C	-3.632701	-2.449090	0.302200
N	-3.854241	-3.395723	-0.638880
C	-4.698948	-4.355096	-0.120366
C	-4.993434	-3.982463	1.162979
N	-4.329564	-2.798607	1.407181
C	-3.364283	-3.398983	-2.022487
C	-4.409124	-2.065136	2.678991
C	-3.632264	2.452651	-0.215690
C	-4.826260	2.464152	-0.982945

C	-5.607678	3.611970	-1.042211
C	-5.246446	4.746456	-0.299512
C	-4.093787	4.732318	0.501444
C	-3.283329	3.604615	0.535261
C	3.634300	-2.457067	-0.172786
N	3.719939	-3.268602	-1.252985
C	4.613070	-4.283326	-0.982700
C	5.076677	-4.085224	0.290079
N	4.466101	-2.948524	0.774487
C	3.049594	-3.093528	-2.547079
C	4.716758	-2.395310	2.113226
H	-5.875351	5.639635	-0.331020
H	5.891351	5.639246	0.125794
H	1.601109	3.008338	1.301641
H	0.177128	1.999568	1.615196
H	-0.617763	3.939931	0.433090
H	0.634341	3.860812	-0.793504
H	-1.644141	2.898535	-1.530058
H	-0.227609	1.866524	-1.803373
H	1.228487	-3.353100	0.021989
H	-1.215858	-3.347411	0.248820
H	-4.613481	0.032012	0.166437
H	4.618583	0.026027	-0.090668
H	4.857515	-5.053802	-1.712308
H	5.786103	-4.661856	0.881787
H	-5.030200	-5.214309	-0.701472
H	-5.616929	-4.464895	1.914215
H	-5.110373	1.591928	-1.576114
H	-2.430450	3.578498	1.213646
H	-6.505190	3.628772	-1.664019
H	-3.846052	5.601384	1.114554

H	5.045957	1.688769	1.608843
H	2.500459	3.491125	-1.419878
H	6.449870	3.722585	1.619105
H	3.924421	5.510260	-1.398664
H	-4.318912	-2.779346	3.507823
H	-3.591537	-1.338360	2.738317
H	-5.376958	-1.548499	2.750829
H	-2.752390	-4.294922	-2.195213
H	-4.220524	-3.408744	-2.711308
H	-2.767383	-2.498251	-2.203365
H	2.329805	-3.908331	-2.706357
H	2.534186	-2.127040	-2.566584
H	3.801599	-3.113991	-3.347742
H	4.735574	-3.217691	2.840275
H	5.687970	-1.880207	2.127059
H	3.915717	-1.697426	2.380

13. References

(1) S. Stoll, A. Schweiger, *J. Magn. Reson.* **2006**, *178*, 42.

(2) X. Bantreil, S. P. Nolan, *Nat. Protoc.* **2011**, *6*, 69.

(3) J. E. Parks, B. E. Wagner, R.-H. Holm, *J. Organomet. Chem.* **1973**, *56*, 53-66.

(4) A. Ogawa, K. Oohora, W. Gu, T. Hayashi *Chem. Commun.* **2019**, *55*, 493-496.

(5) a) D. DiMondo, M. E. Thibault, J. Britten, M. Schlaf, *Organometallics* **2013**, *32*, 6541–6554; b) T. Nagata, *Chem. Eng. News* **2000**, 78, 2.

(6) G. M. Sheldrick, *Acta Cryst*. **2008**, *A64*, 112.

(7) O. V Dolomanov, L. J. Bourhis, R. J. Gildea, J. A. K. Howard, H. Puschmann, *J. Appl. Cryst*. **2009,** *42*, 339.

(8) T. Kottke, D. Stalke, *J. Appl. Cryst*. **1993**, *26*, 615.

(9) P. W. Antoni, M. M. Hansmann, *J. Am. Chem. Soc.* **2018**, *140*, 14823.

(10) (a) M. Werr, E. Kaifer, H. Wadepohl, H.-J. Himmel, *Chem. Eur. J.* **2019**, *25*, 12981 - 12990; (b) N. Elgrishi, K. J. Rountree, B. D. McCarthy, E. S. Rountree, T. T. Eisenhart, J. L. Dempsey, *J. Chem. Educ.* **2018**, *95*, 197-206.

(11) C. S. Sevov, D. P. Hickey, M. E. Cook, S. G. Robinson, S. Barnett, S. D. Minteer, M. S. Sigman, M. S. Sanford, *J. Am. Chem. Soc.* **2017**, *139*, 2924-2927.

(12) (a) P. Hohenberg, W. Kohn, *Phys. Rev. B* **1964**, *136*, B864; (b) W. Kohn, L. Sham, *J. Phys. Rev.* **1965**, *140*, A1133.

(13) Gaussian 16, Revision C.01, M. J. Frisch, G. W. Trucks, H. B. Schlegel, G. E. Scuseria, M. A. Robb, J. R. Cheeseman, G. Scalmani, V. Barone, G. A. Petersson, H. Nakatsuji, X. Li, M. Caricato, A. V. Marenich, J. Bloino, B. G. Janesko, R. Gomperts, B. Mennucci, H. P. Hratchian, J. V. Ortiz, A. F. Izmaylov, J. L. Sonnenberg, D. Williams-Young, F. Ding, F. Lipparini, F. Egidi, J. Goings, B. Peng, A. Petrone, T. Henderson, D. Ranasinghe, V. G. Zakrzewski, J. Gao, N. Rega, G. Zheng, W. Liang, M. Hada, M. Ehara, K. Toyota, R. Fukuda, J. Hasegawa, M. Ishida, T. Nakajima, Y. Honda, O. Kitao, H. Nakai, T. Vreven, K. Throssell, J. A. Montgomery, Jr., J. E. Peralta, F. Ogliaro, M. J. Bearpark, J. J. Heyd, E. N. Brothers, K. N. Kudin, V. N. Staroverov, T. A. Keith, R. Kobayashi, J. Normand, K. Raghavachari, A. P. Rendell, J. C. Burant, S. S. Iyengar, J. Tomasi, M. Cossi, J. M. Millam, M. Klene, C. Adamo, R. Cammi, J. W. Ochterski, R. L. Martin, K. Morokuma, O. Farkas, J. B. Foresman, and D. J. Fox, Gaussian, Inc., Wallingford CT, **2016**.

(14) (a) F. Weigend, R. Ahlrichs, *Phys. Chem. Chem. Phys.* **2005**, *7*, 3297. (b) F. Weigend, *Phys. Chem. Chem. Phys.* **2006**, *8*, 1057.

(15) (a) S. Grimme, S. Ehrlich, L. Goerigk, *J. Comp. Chem*. **2011**, **32**, 1456. (b) S. Grimme, J. Antony, S. Ehrlich, H. Krieg, *J. Chem. Phys.* **2010**, *132*, 154104.

(16) Basis set / functional screening on structurally comparable systems, see: P.W. Antoni, T. Bruckhoff M. M. Hansmann, *J. Am. Chem. Soc.* **2019***, 141,* 9701.

(17) a) J. R. Cheeseman, G. W. Trucks, T. A. Keith, M. J. Frisch, *J. Chem. Phys.* **1996**, *104*, 5497; b) K. Wolinski, J. F. Hilton, P. Pulay, *J. Am. Chem. Soc.* **1990**, *112*, 8251.

(18) CYLview, 1.0b; Legault, C. Y. Université de Sherbrooke, **2009** (http://www.cylview.org).

(19) G. Knizia, *J. Chem. Theory Comput.* **2013**, *9* 4834.

(20) GaussView, Version 6.1, R. Dennington, T. A. Keith, J. M. Millam, Semichem Inc., Shawnee Mission, KS, **2016**.

(21) T. Nakazato, H. Takekoshi, T. Sakurai, H. Shinokubo, Y. Miyake, *Angew. Chem.* **2021**, *133*, 13996–14000; *Angew. Chem. Int. Ed.* **2021**, *60*, 13877–13881.

(22) A. Dreuw, M. Head-Gordon, *J. Am. Chem. Soc.* **2004**, *126*, 4007-4016.
